# Copper‐Photoredox‐Catalyzed C(sp^3^)–C(sp^3^) Reductive Cross‐Coupling of Alkyl Bromides with BCP‐Thianthrenium Reagents

**DOI:** 10.1002/anie.202506785

**Published:** 2025-05-26

**Authors:** Saikat Pandit, Tobias Ritter

**Affiliations:** ^1^ Max‐Planck‐Institut für Kohlenforschung Mülheim an der Ruhr Germany; ^2^ Institute of Organic Chemistry RWTH Aachen University Aachen Germany

**Keywords:** Bicyclopentylation, Bioisosteres, Copper catalysis, Reductive coupling, Thianthrenium salt

## Abstract

Herein, we report a reductive cross‐coupling reaction of bicyclo[1.1.1]pentyl (BCP)‐thianthrenium reagents and alkyl bromides. The reaction is catalyzed by a copper/photoredox catalyst system. The approach is the first example of a cross‐coupling between BCP‐based reagents with alkyl electrophiles.

## Graphical abstract

We show a reductive cross‐coupling reaction of bicyclo[1.1.1]pentyl (BCP)‐thianthrenium reagents and alkyl bromides, which is catalyzed by a copper/photoredox catalyst system. The reaction provides a variety of BCP‐alkylated products through the formation of a C(sp^3^)−C(sp^3^) bond.

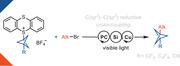

The rigid 1,3‐disubstituted bicyclo[1.1.1]pentane (BCP) is a three‐dimensional bioisostere of para‐substituted phenyl rings, providing two exit vectors in a 180° dihedral angle.^[^ ^1^, ^2^, ^3^, ^4^ ^]^ Approximately, 45% of marketed small molecule drugs contain a phenyl ring.^[^ ^5^ ^]^ Replacement of an aryl ring with 1,3‐disubstituted BCP can provide improved metabolic and pharmacokinetic properties, such as increased metabolic stability, membrane permeability, and solubility.^[^ ^6^ ^]^ The most prevalent approach to accessing BCP compounds entails the opening of the strained [1.1.1]propellane through the installation of functional groups at the BCP bridgehead positions, followed by using the functional group for functionalization.^[^ ^7^, ^8^ ^]^ Over the past two decades, several BCP reagents have been developed, such as BCP Grignard reagents,^[^ ^9^ ^]^ BCP halides,^[^ ^10^ ^]^ and BCP boronates,^[^ ^11^, ^12^, ^13^ ^]^ which can be used for C─C bond formation reactions to form C(sp^3^)─C(sp^2^) bonds.^[^ ^9^, ^13^, ^14^, ^15^ ^]^ However, to the best of our knowledge, none of the reagents have been reported to undergo cross‐coupling reactions with alkyl electrophiles to form C(sp^3^)−C(sp^3^) bonds (Figure 1b). Here, we achieved the first reductive cross‐coupling reaction between alkyl bromides and BCP‐thianthrenium reagents to prepare alkyl‐BCP compounds, including diarylmethane analogs. Diarylmethanes are important structural motifs in pharmaceutically active compounds,^[^ ^16^, ^17^ ^]^ such as in the cholesterol‐lowering drug beclobrate,^[^ ^18^ ^]^ the antispasmodic drug papaverine,^[^ ^19^ ^]^ and the antineoplastic drug and chemopotentiator tesmilifene^[^ ^20^ ^]^ (Figure 1a). Our method may be useful for preparation of the BCP analogs of diarylmethanes from BCP‐thianthrenium reagents that are more stable and more readily stored than a propellane solution.^[^ ^21^, ^22^, ^23^ ^]^

**Figure 1:**
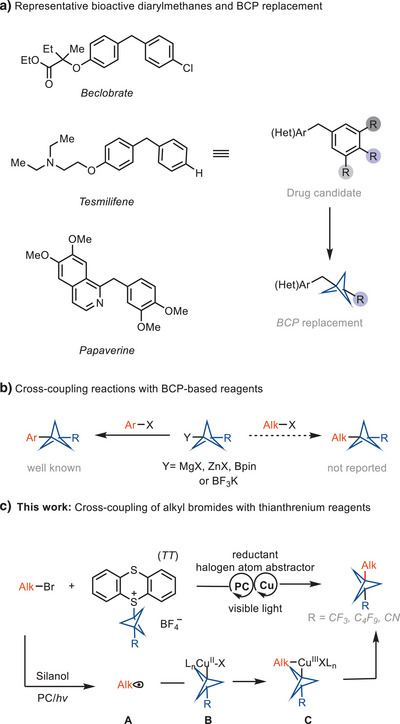
Reductive coupling of alkyl bromides with BCP‐thianthrenium salts. X, halides; PC, photocatalyst.

Traditional cross‐coupling reactions generally involve an organometallic reagent and an electrophile.^[^ ^24^, ^25^ ^]^ Cross‐electrophile coupling reactions can directly make use of two electrophilic coupling partners that are often more readily available.^[^ ^26^, ^27^ ^]^ In most cases, at least one coupling partner with C(sp^2^) hybridization at the reactive site is required to control chemoselective reductive cross‐electrophile coupling reactions.^[^ ^26^, ^28^, ^29^, ^30^, ^31^, ^32^ ^]^ The cross‐coupling reaction between two alkyl electrophiles has been achieved via nickel‐catalyzed reductive conditions and was first demonstrated by the Gong group but generally deals with the challenge of chemoselectivity.^[^ ^33^, ^34^, ^35^ ^]^ The mechanism is thought to involve the successive oxidative addition of low valent Ni‐intermediates to the two alkyl halides, which is proposed to occur through radical intermediates during the course of the coupling.

A challenge in this reaction is the propensity to form homo‐coupling by‐products.^[^ ^33^ ^]^ In 2014, the Liu group developed an efficient copper‐catalyzed system for the reductive cross‐coupling of alkyl‐OTs/OMs with alkyl bromides, and they proposed that the reaction proceeds via the initial formation of a Grignard reagent, which then reacts with the Cu catalyst to form an organocopper intermediate for subsequent S_N_2 substitution at alkyl pseudohalides.^[^ ^36^ ^]^ A nickel‐photoredox dual catalyst system was developed for the cross‐electrophilic coupling of aliphatic halides using silanol as an effective halogen atom abstractor.^[^ ^37^ ^]^ N‐alkylpyridinium salts and redox active esters were also reported to undergo cross‐electrophilic reaction with alkyl halides to form C(sp^3^)−C(sp^3^) bonds using a nickel catalyst.^[^ ^38^, ^39^ ^]^ Nickel catalysts are usually preferred due to facile oxidative addition of alkyl halides to low‐valent nickel under mild conditions^[^ ^40^ ^]^ and the ease of reduction of nickel species in solution with reductants such as Zn^[^ ^41^ ^]^ or Mn.^[^ ^42^ ^]^ To address the chemoselective oxidative addition to avoid homocoupling reactions, electrochemical approaches have been developed for cross‐electrophilic coupling of two alkyl halides or the coupling between alkyl bromides and alkyl tosylates.^[^ ^40^, ^41^, ^42^, ^43^ ^]^

To date, BCP reagents, such as BCP organometallic reagents, BCP halides, and BCP boronates, have been reported to undergo C─C bond formation reactions with aromatic halides, resulting in the formation of C(sp^3^)−C(sp^2^) bonds. However, none of these reagents are reported to undergo cross‐coupling reactions with aliphatic halides to form C(sp^3^)−C(sp^3^) bonds. Considering the importance of the BCP moiety in medicinal chemistry, we leverage the unusual redox properties of the BCP‐thianthrenium scaffold^[^ ^21^ ^]^ to reductive cross‐electrophilic‐coupling reactions with alkyl halides. We have demonstrated that BCP‐thianthrenium reagents can engage in transition metal‐mediated functionalization of phenols, alcohols, various N‐nucleophiles, and (het)aryl bromides, but aliphatic carbon couplings were out of scope.^[^ ^21^, ^22^ ^]^ The high reduction potential (E_1/2_ BCP − TT^+^ = −1.40 V vs. SCE in MeCN) and the low BDE facilitate mesolytic cleavage of the C─S bond to form a BCP radical after single electron reduction, which can engage in transition metal‐catalyzed bond formation.

We sought to use a dual copper/photoredox catalyst for the reductive cross‐coupling of BCP‐thianthrenium reagents and alkyl bromides because both structures are more readily available than the corresponding organometallic reagents and would provide access to synthetically useful motifs that are currently challenging to access. Free radical pathways can give rise to homocoupling by‐products and a lack of cross‐selectivity.^[^ ^44^ ^]^ The dual system, combined with the distinct redox properties of thianthrenium salts, which differ from the corresponding halides, may address the homocoupling problem. We envisioned that a silyl radical/halogen abstraction/copper capture mechanism^[^ ^45^, ^46^ ^]^ could achieve cross‐coupling while circumventing the homocoupling by‐products. A halogen atom abstraction followed by radical oxidative ligation to a copper catalyst would circumvent a potentially slow oxidative addition of the alkyl bromide.^[^ ^45^, ^46^ ^]^ If the various rates were appropriate, two consecutive alkyl radical oxidative ligations to copper would generate a high‐valent Cu(III) species **C**, from which facile reductive elimination would provide the desired product (Figure 1c). Based on our findings and previous literature,^[^ ^37^ ^]^ we provide a plausible catalytic cycle shown in Figure 2. The mechanism may be initiated by photoexcitation of the 4CzIPN photocatalyst (PC) for single electron transfer (SET) to tris(trimethylsilyl)silanol (TMS_3_SiOH), which would generate a silicon‐centered radical **I** after deprotonation followed by radical Brook rearrangement (E_1/2_^red^ 4CzIPN *PC/PC^−^ = +1.35 V vs. SCE in MeCN and E_p_^red^ TMS_3_SiOH^+•^/TMS_3_SiOH = +1.54 V vs. SCE in MeCN). The silyl radical **I** is expected to abstract a bromine atom from alkyl bromide **1a** at a rate on the order of 10^7^ M^−1^ s^−1^ to form the corresponding alkyl radical **II**.^[^ ^45^ ^]^ The SET between the reducing photocatalyst (PC^−^) and the BCP−TT^+^ salt would generate the BCP radical **III** and regenerate the photocatalyst (PC) (E_1/2_^red^ 4CzIPN PC/PC^−^ = −1.21 V vs. SCE in MeCN and E_1/2_ BCP − TT^+^ = −1.40 V vs. SCE in MeCN). Oxidative ligation of BCP radical **III** with Cu^I^ species **IV** would generate Cu^II^−BCP adduct **V**. Subsequent combination of Cu^II^−BCP adduct **V** with alkyl radical **II** would generate the high valent alkyl‐Cu^III^−BCP species **VI**, which upon reductive elimination would yield the desired cross‐coupled product **1** and regenerate the Cu^I^ catalyst **IV**.

**Figure 2:**
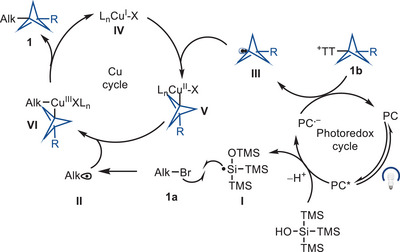
Proposed mechanism.

The reaction between methyl 3‐(bromomethyl)benzoate (**1a**) and trifluoromethyl bicyclopentyl thianthrenium salt (CF_3_‐BCP−TT^+^BF_4_^−^, **1b**) occurred with CuCl as catalyst, K_2_HPO_4_ as base, 1,2,3,5‐tetrakis(carbazol‐9‐yl)‐4,6‐dicyanobenzene (4CzIPN) as photocatalyst, tris(trimethylsilyl)silanol (TMS_3_SiOH) as reducing agent as well as a halogen atom abstraction source under blue LED irradiation at 460 nm to give bicyclopentylmethylarene **1** in 72% yield. Optimization of the reaction conditions revealed that the combinations of CuCl, K_2_HPO_4_, 4CzIPN, and TMS_3_SiOH, as well as blue LED irradiation (Table 1) are all required for an efficient reaction (entries 2–6). The reaction can also be carried out with tris(trimethylsilyl)silane (TMS_3_SiH) but the silanol TMS_3_SiOH is more efficient (entry 7). Additionally, n‐tributylamine has been employed in lieu of silanol (entries 9 and 10) as it is also known to generate an alpha amino radical under photoredox conditions, which can abstract the halogen atom to generate the alkyl radical.

**Table 1:** Reaction development.

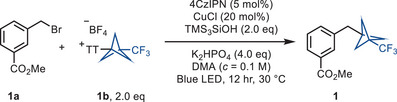		
Entries	Condition	Yield of **1** a)
1	No change	72%
2	No CuCl	0%
3	No TMS_3_SiOH	0%
4	No 4CzIPN	0%
5	No Base	26%
6	No Blue LED	0%
7	TMS_3_SiH instead of TMS_3_SiOH	33%
8	[Ir{dF(CF_3_)ppy}_2_(dtbbpy)]PF_6_ instead of 4CzIPN	31%
9	*^n^* Bu_3_N instead of TMS_3_SiOH, K_2_HPO_4_	45%
10	*^n^* Bu_3_N, K_2_HPO_4_ instead of TMS_3_SiOH	53%

^a)^ Yields were determined by ^19^F NMR with PhCF_3_ as internal standard.

In general, the reaction proceeds with activated alkyl bromides such as benzyl bromide (e.g., **2**, **4**, **6**, and **8**), (bromomethyl)heterocycles (e.g., **3**), and unactivated alkyl bromides including primary alkyl bromides (e.g., **10**, **12**, and **14**) and secondary alkyl bromides (e.g., **15**, **16**, and **17**) (Scheme 1). Given the importance of diarylmethanes in drug discovery^[^ ^16^, ^17^ ^]^ and the fact that our reaction can afford the BCP analog of diarylmethanes, we focused primarily on benzyl bromide substrates. A series of benzyl bromides containing electron‐withdrawing as well as electron‐donating groups afforded the BCP functionalized product. Dimerization of the benzyl bromides is a side reaction and accounts for some of the mass balance. The reaction tolerates a variety of functional groups, including esters (**1**, **7**, **12**, **13**, and **14**), halides (**3** and **13**), cyano (**2**), methoxy (**4**), phthalimide (**8, 10,** and **11**), and ketone (**17**). (Bromomethyl)pyridines (**3**) also afford the corresponding BCP adducts. We can prepare the celecoxib‐BCP adduct (**9**) from the corresponding benzyl bromide of the nonsteroidal anti‐inflammatory drug, celecoxib. Secondary alkyl bromides also afford the BCP‐alkyl product (**15**, **16**, and **17**), however, with lower reactivity than the primary alkyl bromides (**10**, **12**, and **13**). The scope of reactivity with BCP−TT^+^ reagents was further expanded to NC‐BCP−TT^+^ (**1c**) and C_4_F_9_‐BCP−TT^+^ (**1d**) reagents. Benzyl bromides (**19** and **20**), unactivated primary alkyl bromides (**18** and **21**), and (bromomethyl)pyridines (**22**) all give the corresponding BCP‐alkyl product reacting with C_4_F_9_‐BCP−TT^+^ (**1d**) or NC‐BCP−TT^+^ (**1c**) reagents. The reactivity of NC‐BCP−TT^+^ (**1c**) is usually lower than for CF_3_‐BCP−TT^+^ (**1b**) or C_4_F_9_‐BCP−TT^+^ (**1d**) presumably due to consequence of electronic through‐space interactions of the more electron‐withdrawing cyano substituent, which lowers the nucleophilicity of the bridgehead carbon‐based radical, and this is evident from the chemical shift value of the bridgehead C–TT^+^ atom, and for **1c**, the bridgehead C–TT^+^ atom is more deshielded compared to **1b** or **1d**. In addition, the cyano group in the BCP scaffold can further be diversified to other substituents, such as carboxylic acid, methylcarboxylate, methylamino, amide, amino substituents, and etc.^[^ ^21^, ^22^ ^]^

**Scheme 1:**
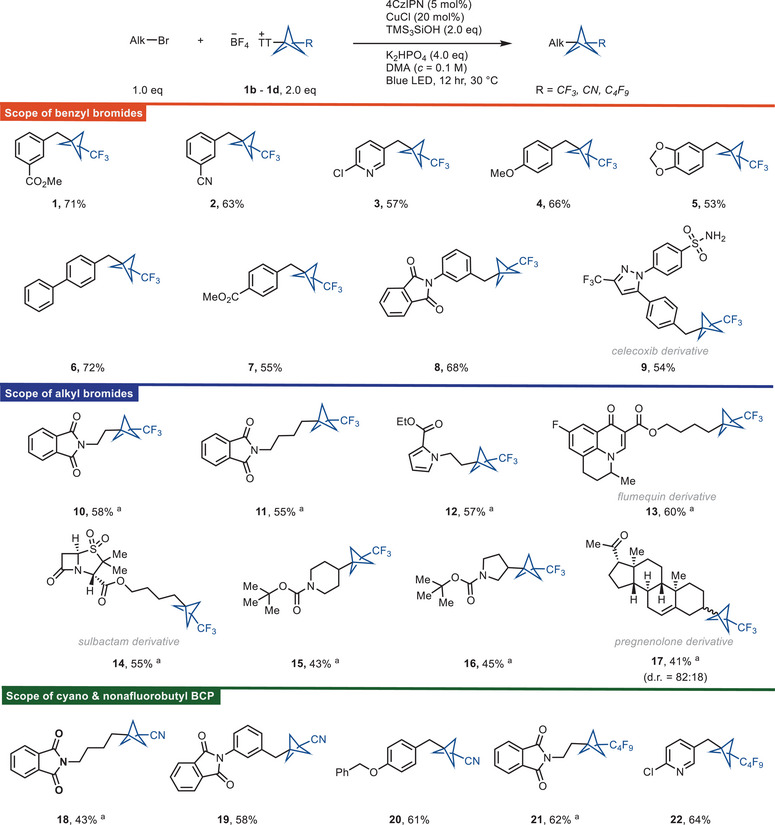
Substrate scope of the reductive cross‐coupling between alkyl bromides and BCP‐thianthrenium salts. General reaction conditions: alkyl bromide (0.25 mmol, 1.0 equiv.), **1b**–**1d** (2.0 equiv.), 4CzIPN (5.0 mol%), CuCl (20 mol%), TMS_3_SiOH (2.0 equiv.), K_2_HPO_4_ (4.0 equiv.), DMA (*c *= 0.1 M), blue LED (460 nm, 40 W), 12 hr, 30 °C. ^a )^CuCl_2_ (20 mol%) was used as catalyst, Na_2_CO_3_ (4.0 equiv.) was used as base, and DMSO (*c *= 0.05 M) was used as solvent. d.r., diastereomeric ratio.

The scope of BCP−TT^+^ reagents is currently limited to a few substituents. Expansion beyond these substituents is conceptually challenging and would represent an advance in the field of BCP chemistry.^[^ ^22^ ^]^ However, in contrast to the limited scope of BCP substituents, a wide range of alkyl substituents have been employed, including benzyl, primary and secondary alkyl. In our approach, we have not been able to couple tertiary alkyl bromides with BCP−TT^+^, presumably due to unfavorable steric interactions, and the fact that tertiary bromides are prone to give significant amounts of protodebromination and elimination by‐products.^[^ ^43^ ^]^ Our approach is therefore complementary to radical based reactions that can incorporate tertiary alkyl substituents.^[^ ^47^ ^]^ The fundamental difference of our current method is the synthesis of BCP compounds from a stable, storable, and easy to handle reagent. Reagents, such as **1b**, can be prepared and stored under ambient conditions for future use. This shows an advancement in the use of synthetic methods for accessing BCP compounds. In addition, to best of our knowledge, the CF_3_ and C_4_F_9_ substituted BCP alkyl products (e.g., **9**, **14**, **17**, **21**, **22**, and etc.) are not currently known to be accessed by other methods, including the approaches that access BCP compounds directly from propellane. However, we can achieve these products by reductive cross‐electrophilic‐coupling reactions with **1b** or **1d**.

The cyano group in BCP reagent **1c** can serve as a linchpin to synthesize analogs of drug molecules. For example, the BCP analog of tesmilifene, **25**, was synthesized from **20**, by hydrolysis the corresponding carboxylic acid **23**, and hydrodecarboxylation (Scheme 2).

**Scheme 2:**
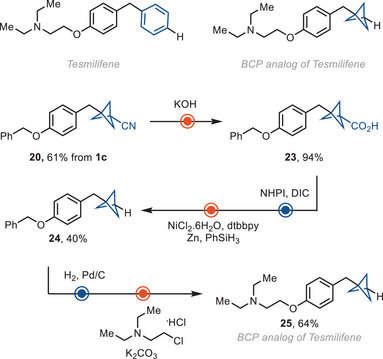
Synthesis of BCP analog of tesmilifene.

In summary, we have described an efficient reductive cross‐coupling reaction of alkyl bromides and BCP−TT^+^ reagents, which yields a range of BCP‐alkylated products through the formation of a C(sp^3^)─C(sp^3^) bond. It is noteworthy that our approach employed the dual/copper‐photoredox condition for the coupling of two alkyl electrophiles, in contrast to the conventional use of nickel reductive conditions. We anticipate that our approach may facilitate the development of saturated analogs of diarylmethane drugs in the pharmaceutical industry.

## Conflict of Interests

The authors declare no conflict of interest.

## Supporting information



Supporting Information

## Data Availability

The data that support the findings of this study are available in the supporting information of this article.
