# Supplementary material for: Copper‐Photoredox‐Catalyzed C(sp3)–C(sp3) Reductive Cross‐Coupling of Alkyl Bromides with BCP‐Thianthrenium Reagents
Source: Angew Chem Int Ed Engl. 2025 May 26;64(30):e202506785. doi: 10.1002/anie.202506785 (PMC12281079; doi:10.1002/anie.202506785)
Supplement: Supplementary file 1 — Supporting Information [file ANIE-64-e202506785-s001.pdf]

SUPPORTING INFORMATION

Copper-photoredox-catalysed C(sp^3)–C(sp^3) reductive cross-coupling of alkyl bromides with BCP-thianthrenium reagents

Saikat Pandit and Tobias Ritter*

Max-Planck-Institut für Kohlenforschung, Mülheim an der Ruhr, Germany

Institute of Organic Chemistry, RWTH Aachen University, Aachen, Germany

E-mail: ritter@kofo.mpg.de

TABLE OF CONTENTS

TABLE OF CONTENTS 1

MATERIALS AND METHODS 6

EXPERIMENTAL DATA 7

Preparation of [1.1.1]propellane stock solution 7

Preparation of bicyclo[1.1.1]pentyl thianthrenium salts 7

S-(Trifluoromethyl)thianthrenium triflate **1b-1** 7

Trifluoromethylbicyclo[1.1.1]pentyl thianthrenium salt (**1b**) 8

Nonafluorobutyl thianthrenium salt (**1d-1**) 9

Nonafluorobutylbicyclo[1.1.1] pentyl thianthrenium salt (**1d**) 10

Thianthrenium tetrafluoroborate (**$TT^{•+}$ BF_4^-**) 11

Cyanobicyclo[1.1.1]pentyl thianthrenium salt (**1c**) 11

General procedure for Cu-photoredox-catalysed reductive cross-coupling of alkyl bromides with BCP-thianthrenium reagents 12

Preparation of TMS_3SiOH and alkyl bromides 13

TMS_3SiOH 13

Celecoxib derivative **S9** 14

Pyrrole derivative **S12** 15

Flumequin derivative **S13** 15

Sulbactam derivative **S14** 16

Pregnenolone derivative **S17** 17

Reaction condition optimization for Cu-photoredox-catalysed reductive cross-coupling of alkyl bromides with BCP-thianthrenium reagent **1b** 18

Bicyclo[1.1.1]pentylmethylarene **1** 24

Bicyclo[1.1.1]pentylmethylarene **2** 25

Bicyclo[1.1.1]pentylmethylpyridine **3** 25

Bicyclo[1.1.1]pentylmethylarene **4** 26

Bicyclo[1.1.1]pentylmethylarene **5** 27

Bicyclo[1.1.1]pentylmethylarene **6** 28

Bicyclo[1.1.1]pentylmethylarene **7** 29

Bicyclo[1.1.1]pentylmethylarene **8** 29

Bicyclo[1.1.1]pentylmethylarene **9** 30

Bicyclo[1.1.1]pentylalkane **10** 31

Bicyclo[1.1.1]pentylalkane **11** 32

Bicyclo[1.1.1]pentylalkane **12** 33

Bicyclo[1.1.1]pentylalkane **13** 33
Bicyclo[1.1.1]pentylalkane **14** 34
Bicyclo[1.1.1]pentylalkane **15** 35
Bicyclo[1.1.1]pentylalkane **16** 36
Bicyclo[1.1.1]pentylalkane **17** 37
Bicyclo[1.1.1]pentylalkane **18** 38
Bicyclo[1.1.1]pentylmethylarene **19** 38
Bicyclo[1.1.1]pentylmethylarene **20** 39
Bicyclo[1.1.1]pentylalkane **21** 40
Bicyclo[1.1.1]pentylmethylpyridine **22** 41
Synthesis of BCP analog of tesmilifene **25** 42
Unsuccessful substratres 44

SPECTROSCOPIC DATA 45

^{1}H NMR of TMS_3SiOH 45
^{13}C NMR of TMS_3SiOH 46
^{1}H NMR of pyrrole derivative **S12** 47
^{13}C NMR of pyrrole derivative **S12** 48
^{1}H NMR of flumequin derivative **S13** 49
^{13}C NMR of flumequin derivative **S13** 50
^{19}F NMR of flumequin derivative **S13** 51
^{1}H NMR of sulbactam derivative **S14** 52
^{13}C NMR of sulbactam derivative **S14** 53
^{1}H NMR of bicyclo[1.1.1]pentylmethylarene **1** 54
^{13}C NMR of bicyclo[1.1.1]pentylmethylarene **1** 55
^{19}F NMR of bicyclo[1.1.1]pentylmethylarene **1** 56
^{1}H NMR of bicyclo[1.1.1]pentylmethylarene **2** 57
^{13}C NMR of bicyclo[1.1.1]pentylmethylarene **2** 58
^{19}F NMR of bicyclo[1.1.1]pentylmethylarene **2** 59
^{1}H NMR of bicyclo[1.1.1]pentylmethylpyridine **3** 60
^{13}C NMR of bicyclo[1.1.1]pentylmethylpyridine **3** 61
^{19}F NMR of bicyclo[1.1.1]pentylmethylpyridine **3** 62

^{1}H NMR of bicyclo[1.1.1]pentylmethylarene **4** ... 63

^{13}C NMR of bicyclo[1.1.1]pentylmethylarene **4** ... 64

^{19}F NMR of bicyclo[1.1.1]pentylmethylarene **4** ... 65

^{1}H NMR of bicyclo[1.1.1]pentylmethylarene **5** ... 66

^{13}C NMR of bicyclo[1.1.1]pentylmethylarene **5** ... 67

^{19}F NMR of bicyclo[1.1.1]pentylmethylarene **5** ... 68

^{1}H NMR of bicyclo[1.1.1]pentylmethylarene **6** ... 69

^{13}C NMR of bicyclo[1.1.1]pentylmethylarene **6** ... 70

^{19}F NMR of bicyclo[1.1.1]pentylmethylarene **6** ... 71

^{1}H NMR of bicyclo[1.1.1]pentylmethylarene **7** ... 72

^{13}C NMR of bicyclo[1.1.1]pentylmethylarene **7** ... 73

^{19}F NMR of bicyclo[1.1.1]pentylmethylarene **7** ... 74

^{1}H NMR of bicyclo[1.1.1]pentylmethylarene **8** ... 75

^{13}C NMR of bicyclo[1.1.1]pentylmethylarene **8** ... 76

^{19}F NMR of bicyclo[1.1.1]pentylmethylarene **8** ... 77

^{1}H NMR of bicyclo[1.1.1]pentylmethylarene **9** ... 78

^{13}C NMR of bicyclo[1.1.1]pentylmethylarene **9** ... 79

^{19}F NMR of bicyclo[1.1.1]pentylmethylarene **9** ... 80

^{1}H NMR of bicyclo[1.1.1]pentylalkane **10** ... 81

^{13}C NMR of bicyclo[1.1.1]pentylalkane **10** ... 82

^{19}F NMR of bicyclo[1.1.1]pentylalkane **10** ... 83

^{1}H NMR of bicyclo[1.1.1]pentylalkane **11** ... 84

^{13}C NMR of bicyclo[1.1.1]pentylalkane **11** ... 85

^{19}F NMR of bicyclo[1.1.1]pentylalkane **11** ... 86

^{1}H NMR of bicyclo[1.1.1]pentylalkane **12** ... 87

^{13}C NMR of bicyclo[1.1.1]pentylalkane **12** ... 88

^{19}F NMR of bicyclo[1.1.1]pentylalkane **12** ... 89

^{1}H NMR of bicyclo[1.1.1]pentylalkane **13** ... 90

^{13}C NMR of bicyclo[1.1.1]pentylalkane **13** ... 91

^{19}F NMR of bicyclo[1.1.1]pentylalkane **13** 92

^{1}H NMR of bicyclo[1.1.1]pentylalkane **14** 93

^{13}C NMR of bicyclo[1.1.1]pentylalkane **14** 94

^{19}F NMR of bicyclo[1.1.1]pentylalkane **14** 95

^{1}H NMR of bicyclo[1.1.1]pentylalkane **15** 96

^{13}C NMR of bicyclo[1.1.1]pentylalkane **15** 97

^{19}F NMR of bicyclo[1.1.1]pentylalkane **15** 98

^{1}H NMR of bicyclo[1.1.1]pentylalkane **16** 99

^{13}C NMR of bicyclo[1.1.1]pentylalkane **16** 100

^{19}F NMR of bicyclo[1.1.1]pentylalkane **16** 101

^{1}H NMR of bicyclo[1.1.1]pentylalkane **17** 102

^{13}C NMR of bicyclo[1.1.1]pentylalkane **17** 103

^{19}F NMR of bicyclo[1.1.1]pentylalkane **17** 104

^{1}H NMR of bicyclo[1.1.1]pentylalkane **18** 105

^{13}C NMR of bicyclo[1.1.1]pentylalkane **18** 106

^{1}H NMR of bicyclo[1.1.1]pentylmethylarene **19** 107

^{13}C NMR of bicyclo[1.1.1]pentylmethylarene **19** 108

^{1}H NMR of bicyclo[1.1.1]pentylmethylarene **20** 109

^{13}C NMR of bicyclo[1.1.1]pentylmethylarene **20** 110

^{1}H NMR of bicyclo[1.1.1] pentylalkane **21** 111

^{13}C {^{1}H, ^{19}F} NMR of bicyclo[1.1.1]pentylalkane **21** 112

^{19}F NMR of bicyclo[1.1.1]pentylalkane **21** 113

^{1}H NMR of bicyclo[1.1.1]pentylmethylpyridine **22** 114

^{13}C {^{1}H, ^{19}F} NMR of bicyclo[1.1.1]pentylmethylpyridine **22** 115

^{19}F NMR of bicyclo[1.1.1]pentylmethylpyridine **22** 116

^{1}H NMR of intermediate **23** 117

^{13}C NMR of intermediate **23** 118

^{1}H NMR of intermediate **24** 119

^{13}C NMR of intermediate **24** 120

^{1}H NMR of BCP analog of tesmilifene **25** 121

^{13}C NMR of BCP analog of tesmilifene **25** 122

REFERENCES 123

MATERIALS AND METHODS

All reactions were carried out under an ambient atmosphere unless otherwise stated and monitored by thin-layer chromatography (TLC). Air- and moisture-sensitive manipulations were performed using standard Schlenk- and glove-box techniques under an atmosphere of argon or dinitrogen. High-resolution mass spectra were obtained using *Q Exactive Plus* from *Thermo*. Concentration under reduced pressure was performed by rotary evaporation at 25–40 °C at an appropriate pressure. Purified compounds were further dried under high vacuum (0.010–0.005 mBar). Yields refer to purified and spectroscopically pure compounds, unless otherwise stated.

Solvents

Anhydrous DMA and DMSO were purchased from Acros Organics and Sigma Aldrich. Other anhydrous solvents were obtained from Phoenix Solvent Drying Systems. All deuterated solvents were purchased from Euriso-Top.

Chromatography

Thin layer chromatography (TLC) was performed using EMD TLC plates pre-coated with 250 µm thickness silica gel 60 F_{254} plates and visualized by fluorescence quenching under UV light and $KMnO_4$ stain. Flash column chromatography was performed using silica gel (40–63 µm particle size) purchased from Geduran®.

Photochemistry

All *BCP*-alkylation reactions were performed with blue LEDs (Kessil A160WE Tuna Blue (460 nm), LED lighting, 40 W).

Spectroscopy and Instruments

NMR spectra were recorded on a Bruker Ascend™ 500 spectrometer operating at 500 MHz, 471 MHz and 126 MHz, for ^{1}H, ^{19}F and ^{13}C acquisitions, respectively; or on a Varian Unity/Inova 600 spectrometer operating at 600 MHz and 151 MHz for ^{1}H and ^{13}C acquisitions, respectively. Chemical shifts are reported in ppm with the solvent residual peak as the internal standard. For ^{1}H NMR: $CDCl_3$, δ 7.26; CD_3CN, δ 1.96; CD_2Cl_2, δ 5.32; DMSO-d_6, δ 2.50. For ^{13}C NMR: $CDCl_3$, δ 77.16; CD_3CN, δ 1.32; CD_2Cl_2, δ 53.84; DMSO-d_6, δ 39.52.[1] ^{19}F NMR spectra were referenced using a unified chemical shift scale based on the ^{1}H resonance of tetramethylsilane (1% v/v solution in the respective solvent).[2] Data is reported as follows: s = singlet, d = doublet, t = triplet, q = quartet, quin = quintet, sext = sextet, sept = septet, m = multiplet, bs = broad singlet; coupling constants are reported in Hz.

Starting materials

All substrates were used as received from commercial suppliers, unless otherwise stated. CF_3-BCP–TT^+ BF_4^- (**1b**), NC-BCP–TT^+ BF_4^- (**1c**), and C_4F_9-BCP–TT^+ BF_4^- (**1d**) was prepared according to the literature.[3] TMS_3SiOH[5], **S9**[6], and **S17**[7] was prepared according to the literature.

EXPERIMENTAL DATA

Preparation of [1.1.1]propellane stock solution

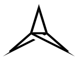

Propellane solution was prepared following the reported procedure[3]:

To an oven-dried 500 mL round-bottom flask containing a teflon-coated magnetic stirring bar was added 1,1-dibromo-2,2-bis(chloromethyl)cyclopropane (16.0 g, 52.8 mmol, 1.00 equiv.). The flask was sealed with a septum-cap, evacuated, and back-filled with argon three times, and then anhydrous Et_2O (33 mL) was added. The mixture was cooled to –45 °C (dry ice/isopropanol bath). Phenyllithium (56 mL, 1.9 M in Bu_2O, 0.11 mol, 2.0 equiv.) was added dropwise via syringe over 15 min at –45°C. The cooling bath was replaced with an ice bath, and the mixture was warmed to 0°C, and then stirred at this temperature for 2 h.

Upon completion of the reaction, the mixture was then distilled at 25 °C (70 mbar) using a rotary evaporator with dry ice trap, the receiving flask of which was immersed in a dry ice/acetone bath. The [1.1.1]propellane stock solution (31 mL, 0.85 M in Et_2O, 50%) was transferred to a flame-dried septum-sealed bottle under an inert atmosphere, and stored at –20°C. The approximate concentration of the solution was determined by quantitative ^1H NMR spectroscopy with 1,2-dichloroethane as an internal standard.

Quantitative NMR Experiment

A sample of the solution containing [1.1.1]propellane in diethyl ether (100 µL) was diluted with dichloroethane (DCE) (25 µL) and $CDCl_3$ was added (*ca.* 0.5 mL). The ratio of the DCE:propellane was determined and used for the calculation of the concentration of the propellane solution. This was performed in duplicate and the average of the two runs was used as the final approximated concentration.

Preparation of bicyclo[1.1.1]pentyl thianthrenium salts

S-(Trifluoromethyl)thianthrenium triflate (1b-1, $TTCF_3^+OTf^-$)

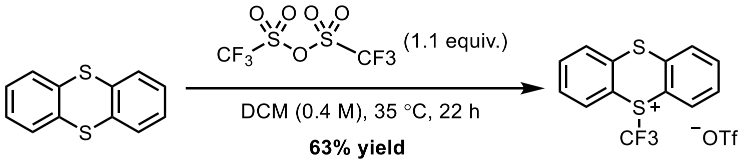

1b-1

1b-1 was prepared according to the literature procedure.[4] Under an ambient atmosphere, a 500 mL two-neck round-bottom flask equipped with a teflon-coated magnetic stirring bar, was charged with thianthrene (17.3 g, 80.0 mmol, 1.00 equiv.) and dichloromethane (200 mL, c = 0.4 M). Subsequently, triflic anhydride (24.8 g, 14.8 mL, 88.0 mmol, 1.10 equiv.) was added in one portion at room temperature. Upon addition of triflic anhydride, the reaction mixture rapidly turned light purple and gradually deepened, accompanied by formation of suspended particles. The reaction mixture was stirred at 35 ºC for 22 h. Subsequently, a saturated aqueous $NaHCO_3$ solution (ca. 100 mL) was added carefully. At this point, the purple color faded

away, and the suspension turned light brown. The suspension was poured into a 500 mL separatory funnel, and the aqueous layer was discarded. The organic layer (light brown solution) was concentrated to dryness under reduced pressure, resulting in the formation of a light brown residue. Diethyl ether (100 mL) was added to the residue and the suspension was stirred vigorously at room temperature for 30 min. The mixture was allowed to stand for 5 min, subsequently, the solvent was decanted carefully. In order to obtain an analytically pure compound, the decanting process was repeated four times with diethyl ether. The resulting yellow slurry was concentrated to dryness under reduced pressure, accumulating to a total of 21.1 g (63%) of S-(trifluoromethyl)thianthrenium triflate (**1b-1**, $TTCF_3^+OTf^-$) as pale yellow solid.

R_f = 0.35 (DCM/MeOH, 10:1 (v/v)).

NMR Spectroscopy:

^{1}H NMR (500 MHz, $CDCl_3$, 298 K, δ): 8.56 (dd, *J* = 8.1, 1.4 Hz, 1H), 7.90 (ddd, *J* = 8.4, 7.4, 1.3 Hz, 1H), 7.82 (dd, *J* = 8.1, 1.3 Hz, 1H), 7.76 (ddd, *J* = 8.4, 7.4, 1.3 Hz, 1H).

^{13}C NMR (126 MHz, $CDCl_3$, 298 K, δ): 137.1, 136.7, 136.6, 130.3, 129.6, 124.4 (q, *J* = 337.3 Hz), 120.7 (q, *J* = 320.2 Hz), 108.7.

^{19}F NMR (471 MHz, $CDCl_3$, 298 K, δ): –51.15, –78.40.

HRMS-ESI (m/z) calculated for $C_{13}H_8S_2F_3^+$ $[M–CSO_2F_3]^+$, 285.0013; found, 285.0014; deviation: +0.4 ppm.

The analytical data was matched with the reported procedure.[4]

Trifluoromethylbicyclo[1.1.1]pentyl thianthrenium salt (1b)

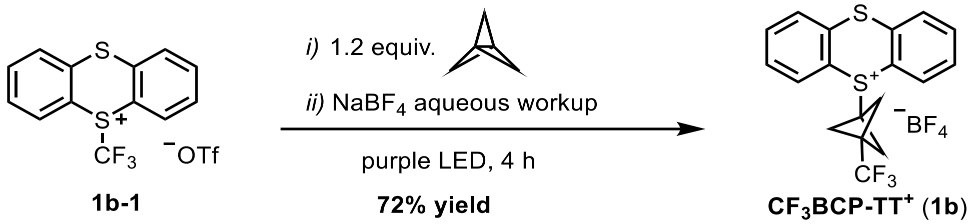

1b was prepared according to the literature procedure.[3] To a 500 mL round-bottom flask equipped with a stirring bar were added trifluoromethyl thianthrenium salt **1b-1** (9.23 g, 21.2 mmol, 1.00 equiv.) and anhydrous MeCN (152 mL, 0.140 M). The flask was capped with a rubber septum, and subsequently [1.1.1]propellane solution in Et_2O (*c* = 0.85 M, 30 mL, 1.7 g, 1.2 equiv.) was added dropwise via syringe to the reaction over 10 min while stirring. Subsequently, the reaction flask was placed 11 cm away from two Kessil PR160-390 nm LEDs. The mixture was irradiated for 4 h while maintaining the temperature at approximately 35 °C through cooling with a fan. After 4 h, the reaction flask was removed from the two Kessil PR160-390 nm LEDs. The mixture was concentrated under reduced pressure, diluted with DCM (150 mL). The DCM solution was poured into a separation funnel and washed with aqueous $NaBF_4$ solution (3 × ca. 150 mL, 10% w/w). All organic phases were combined, dried over $MgSO_4$ (10 g), filtered, and the solvent evaporated under reduced pressure. The crude material was purified by chromatography on silica gel eluting

first with 100% EtOAc and later DCM/*i*-PrOH (1/0–95/5 (v/v)) to afford the title compound as a brown solid. The solid material was dissolved in DCM (ca. 30 mL), and the flask containing the solid in DCM was placed in an ice bath, at which point the residue was triturated with Et_2O (ca. 200 mL). The flask was kept at 0 °C for 1h. The resulting solid was decanted and washed with ice-cold Et_2O (2 × ca. 50 mL), and dried under vacuum overnight yielding a beige solid **1b** (6.73 g, 15.3 mmol, 72%).

Note: Colored trace impurities might result in **1b** as a beige solid (even at >99% purity), but have no observable effect on the reactivity reported in this work.

$\mathbf{R_f}$ = 0.53 (DCM/MeOH, 10:1 (v/v)).

NMR Spectroscopy:

^{1}**H NMR** (500 MHz, CD_2Cl_2, 298 K, δ): 8.34 (d, *J* = 6.9 Hz, 2H), 7.79 (d, *J* = 3.8 Hz, 4H), 7.73 – 7.63 (m, 2H), 2.44 (s, 6H).

^{13}**C NMR** (126 MHz, CD_3CN, 298 K, δ): 136.4, 136.1, 135.3, 130.9, 130.8, 122.4 (q, *J* = 276.0 Hz), 115.6, 53.92 – 53.90 (m), 49.99 – 49.87 (m), 38.7 (q, *J* = 41.3 Hz).

^{19}**F NMR** (471 MHz, $CDCl_3$, 298 K, δ): –71.40 (s), –149.44 (bs), –149.49 (bs).

HRMS-ESI (m/z) calculated for $C_{18}H_{14}F_3S_2^+$ $[M–BF_4]^+$, 351.0483; found, 351.0484; deviation: +0.2 ppm.

The analytical data was matched with the reported procedure.[3]

Nonafluorobutyl thianthrenium salt (1d-1)

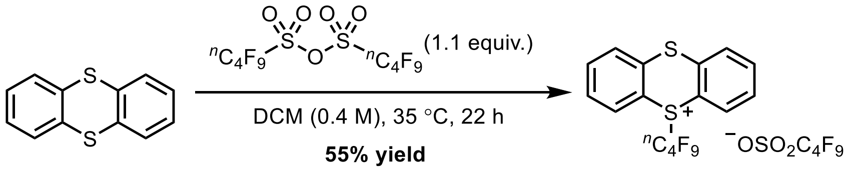

1d-1

1d-1 was prepared according to the literature procedure.[3] Under an ambient atmosphere, a 25 mL two-neck round bottom flask equipped with a teflon-coated magnetic stirring bar, was charged with thianthrene (647 mg, 3.12 mmol, 1.00 equiv.) and DCM (8 mL, c = 0.4 M). Subsequently, nonafluorobutanesulfonic anhydride (2.0 g, 1.1 mL, 3.4 mmol, 1.1 equiv.) was added in one portion at 25 °C. Upon addition of anhydride, the reaction mixture rapidly turned light purple and gradually deepened, accompanied by formation of suspended particles. The reaction mixture was stirred at 35 ºC for 22 h. Subsequently, a saturated aqueous $NaHCO_3$ solution (ca. 5 mL) was added carefully. At this point, the purple color faded away, and the suspension turned light brown. The suspension was poured into a 50 mL separatory funnel, and the aqueous layer was discarded. The organic layer was concentrated to dryness under reduced pressure, resulting in the formation of a light brown residue. Diethyl ether (10 mL) was added to the residue and the suspension was stirred vigorously at 25 °C for 30 min. The mixture was allowed to stand for 5 min, subsequently, the solvent was decanted carefully. In order to obtain an analytically pure compound, the decanting process was repeated

there times with diethyl ether. The resulting yellow slurry was concentrated to dryness under reduced pressure to afford **1d-1** (1.3 g, 1.7 mmol, 55%) as a pale yellow solid.

R*f* = 0.36 (DCM/MeOH, 10:1 (v/v)).

NMR Spectroscopy:

^{1}H NMR (600 MHz, $CDCl_3$, 298 K, δ): 8.55 (d, *J* = 8.1 Hz, 2H), 8.02 – 7.83 (m, 4H), 7.77 (t, *J* = 6.6 Hz, 2H).

^{13}C {^{1}H, ^{19}F} NMR (126 MHz, $CDCl_3$, 298 K, δ): 138.0, 137.8, 136.7, 130.7, 129.5, 120.8, 117.7, 116.8, 114.0, 110.9, 110.1, 109.9, 109.1, 108.2.

^{19}F NMR (471 MHz, $CDCl_3$, 298 K, δ): –80.54 (t, *J* = 9.5 Hz, 3F), –80.78 – –81.23 (m, 3F), –87.64 (t, *J* = 12.6 Hz, 2F), –113.71 – –115.41 (m, 2F), –117.46 – –119.84 (m, 2F), –121.69 (dt, *J* = 8.7, 4.3 Hz, 2F), –125.46 (td, *J* = 13.4, 6.5 Hz, 2F), –126.04 (td, *J* = 13.9, 5.2 Hz, 2F).

HRMS-ESI (m/z) calculated for $C_{16}H_8S_2F_9^+$ [M–$OSO_2C_4F_9$]$^+$, 434.9919; found, 434.9918; deviation: –0.3 ppm.

The analytical data was matched with the reported procedure.[3]

Nonafluorobutylbicyclo[1.1.1] pentyl thianthrenium salt (1d)

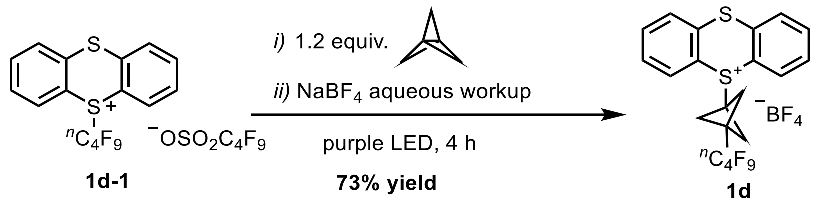

1d was prepared according to the literature procedure.[3] To a 25 mL round-bottom flask equipped with a stirring bar were added nonafluorobutyl thianthrenium salt **1d-1** (734 mg, 1.00 mmol, 1.00 equiv.) and anhydrous MeCN (7.2 mL, 0.14 M). The flask was capped with a rubber septum, and subsequently [1.1.1]propellane solution in Et_2O (*c* = 1.0 M, 1.2 mL, 1.2 equiv.) was added dropwise via syringe to the reaction over 10 min while stirring. Subsequently, the reaction flask was placed in front of two Kessil PR160-390 nm LEDs. The mixture was irradiated for 4 h while maintaining the temperature at approximately 35 °C through cooling with a fan. After 4 h, the reaction flask was removed from the two Kessil PR160-390 nm LEDs. The mixture was concentrated under reduced pressure, diluted with DCM (20 mL). The DCM solution was poured into a separation funnel and washed with aqueous $NaBF_4$ solution (3 × ca. 20 mL, 10% w/w). All organic phases were combined, dried over $MgSO_4$ (10 g), filtered, and the solvent evaporated under reduced pressure. The crude material was purified by chromatography on silica gel eluting first with 100% EtOAc and later DCM/MeOH (100/0–98/2 (v/v)) to afford the title compound **1d** as a brown solid (427 mg, 726 µmol, 73%).

R*f* = 0.53 (DCM/MeOH, 10:1 (v/v)).

NMR Spectroscopy:

^{1}H NMR (600 MHz, $CDCl_3$, 298 K, δ): 8.31 (dt, *J* = 7.9, 1.0 Hz, 2H), 7.85 – 7.78 (m, 4H), 7.69 (dt, *J* = 8.0, 4.4 Hz, 2H), 2.49 (s, 6H).

^{13}C {^{1}H, ^{19}F} NMR (126 MHz, $CDCl_3$, 298 K, δ): 135.3, 135.3, 134.9, 130.3, 129.8, 117.2, 115.0, 112.6, 110.8, 108.4, 54.0, 49.7, 38.0.

^{19}F NMR (471 MHz, $CDCl_3$, 298 K, δ): –80.93 – –81.30 (m, 3F), –115.15 – –115.25 (m, 2F), –121.92 – –122.29 (m, 2F), –126.02 – –126.33 (m, 2F), –149.48 (d, *J* = 25.5 Hz, 4F).

HRMS-ESI (m/z) calculated for $C_{21}H_{14}S_2F_9^+$ [M–BF_4]$^+$, 501.0390; found, 501.0387; deviation: –0.6 ppm.

The analytical data was matched with the reported procedure.[3]

Thianthrenium tetrafluoroborate ($TT^{\bullet+}$ BF_4^-)

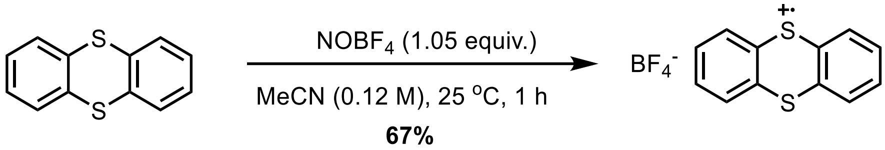

Thianthrenium tetrafluoroborate was synthesized according to a reported procedure.[3] In a nitrogen-filled glove box, thianthrene (2.00 g, 9.25 mmol, 1.00 equiv) was added to nitrosonium tetrafluoroborate (1.13 g, 9.71 mmol, 1.05 equiv) in acetonitrile (80 mL, *c* = 0.12 M) to produce a dark purple solution. The glove box was purged while the reaction mixture was stirred for 1 h at 25 °C, at which point, diethyl ether (250 mL) was added to the stirred reaction mixture. The precipitate was collected by filtration and washed with diethyl ether until the filtrate was colorless (5 × 10 mL). The filter cake was transferred to a 20 mL borosilicate vial and put under vacuum for 5 h, yielding the title compound as a free-flowing black-purple solid (1.88 g, 6.20 mmol, 67% yield).

Determination of purity:

A precise amount (approx. 150 mg) of the thianthrenium tetrafluoroborate was dissolved in anhydrous DCM (30 mL) and MeCN (5 mL) under N_2 atmosphere. KI (500 mg, 3.00 mmol) was added, and the mixture was stirred until the deep purple color of $TT^{\bullet+}$ had been replaced with the dark red brown color of I_2. The liberated iodine was titrated with standard sodium thiosulfate. The procedure was repeated twice, and assays were 99.5% and 98.5% of $TT^{\bullet+}$ BF_4^-.

Cyanobicyclo[1.1.1]pentyl thianthrenium salt (1c)

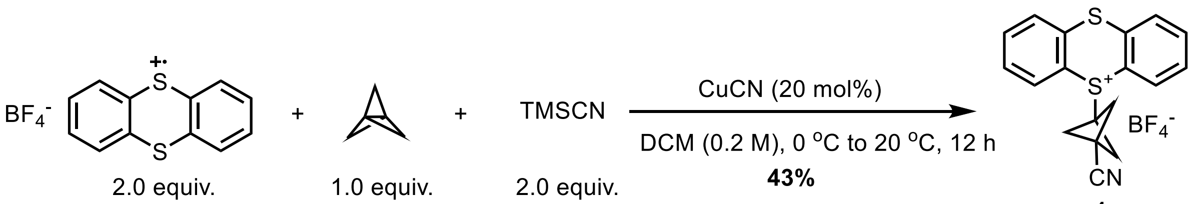

1c

1c was prepared according to the literature procedure.[3] Under nitrogen atmosphere, to a 50 mL round-bottom flask equipped with a magnetic stir bar were added thianthrenium tetrafluoroborate ($TT^{•+}$ BF_4^-) (3.18 g, 10.5 mmol, 1.98 equiv.), CuCN (95 mg, 1.1 mmoL, 20 mol%), and anhydrous DCM (26 mL, *c* = 0.20 M). The flask was sealed with a septum-cap. After cooling to 0 °C, TMSCN (1.33 mL, 1.05 g, 10.6 mmol, 2.00 equiv.) was added in one portion. The mixture was stirred for 10 min at 0 °C. Subsequently, [1.1.1]propellane solution in Et_2O (*c* = 0.71 M) (7.5 mL, 5.3 mmol, 1.0 equiv.) was added to the mixture. Then, the mixture was stirred at 0 °C for 3 h, followed by stirring at 20 °C for 9 h. The mixture was diluted with DCM (20 mL) and MeCN (20 mL), and washed with aqueous $NaBF_4$ solution (1 × 20 mL, 10% w/w). The resulting mixture was poured into a separation funnel, vigorously shaken, and the layers were separated. The aqueous layer was extracted with DCM (2 × 50 mL). The combined organic phase was dried over Na_2SO_4, filtered, and the solvent was removed under reduced pressure. The residue was purified by fast chromatography on silica gel eluting with DCM/MeOH (1/0–30/1 (v/v)) to afford the title compound **1c** as a pale brown solid (890 mg, 2.25 mmol, 43%).

R_f = 0.47 (DCM/MeOH, 10:1 (v/v)).

NMR Spectroscopy:

^{1}H NMR (500 MHz, CD_3CN, 298 K, δ): 8.07 (d, *J* = 7.9 Hz, 2H), 7.92 (d, *J* = 7.9 Hz, 2H), 7.86 (t, *J* = 7.7 Hz, 2H), 7.72 (t, *J* = 7.7 Hz, 2H), 2.61 (s, 6H).

^{13}C NMR (126 MHz, CD_3CN, 298 K, δ): 136.4, 136.2, 135.2, 131.0, 130.8, 115.5, 115.2, 58.5, 51.3, 25.7.

^{19}F NMR (282 MHz, $CDCl_3$, 298 K, δ): –151.67 (bs), –151.72 (bs).

HRMS-ESI (m/z) calculated for $C_{18}H_{14}NS_2^+$ [M–BF_4]$^+$, 308.0560; found, 308.0562; deviation: +0.8 ppm.

The analytical data was matched with the reported procedure.[3]

General procedure for Cu-photoredox-catalysed reductive cross-coupling of alkyl bromides with BCP-thianthrenium reagents

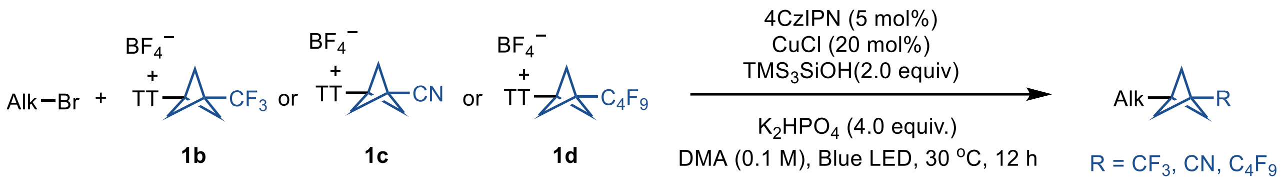

Under nitrogen atmosphere, to a 4 mL borosilicate vial equipped with a magnetic stir bar were added alkyl bromide (0.250 mmol, 1.00 equiv.), **1b** (220 mg, 0.500 mmol, 2.00 equiv.) or **1c** (198 mg, 0.500 mmol, 2.00 equiv.) or **1d** (294 mg, 0.500 mmol, 2.00 equiv.), 4CzIPN (10.0 mg, 12.5 µmoL, 5.00 mol%), CuCl (5.0 mg, 50 µmoL, 20 mol%), K_2HPO_4 (174 mg, 1.00 mmol, 4.00 equiv.), TMS_3SiOH (132 mg, 0.500 mmol, 2.00 equiv.), and anhydrous DMA (2.5 mL, *c* = 0.10 M). The vial was sealed with a septum-cap. Then, the mixture was stirred for 10 min at 25 °C, and placed 5 cm away from two blue LEDs (Kessil A160WE Tuna Blue (460 nm), LED lighting, 40 W). The mixture was irradiated for 12 h while maintaining the temperature at

approximately 30 °C through cooling with a fan. After irradiation, EtOAc (6 mL) was added to the reaction mixture, and the resultant solution was washed with brine (2 × 3 mL). The organic phase was dried over Na_2SO_4, filtered, and the solvent was removed under reduced pressure. The residue was purified by flash column chromatography on silica gel to afford the desired product.

Note: The reaction is air- and water sensitive. Schlenk technique was used to avoid air and water. For simplicity, in our research, we have opted to execute the transformation for most compounds by using a glovebox. Control experiments showed that yields were within error of measurement if the reaction was carried out using a glovebox or Schlenk technique.

Preparation of TMS_3SiOH and alkyl bromides

TMS_3SiOH

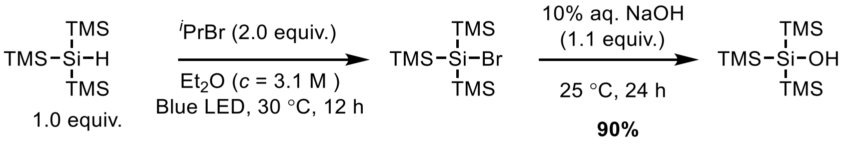

TMS_3SiOH was synthesized according to a reported procedure.[5] To a 20 mL vial equipped with a magnetic stir bar were added TMS_3SiH (3.60 mL, 12.5 mmol, 1.00 equiv.), 2-bromopropane (2.30 mL, 25.0 mmol, 2.00 equiv.), and Et_2O (4 mL, *c* = 3.1 M). The vial was sealed with a septum-cap. Then, the mixture was placed 5 cm away from two blue LEDs (Kessil A160WE Tuna Blue (460 nm), LED lighting, 40 W). The mixture was irradiated for 12 h while maintaining the temperature at approximately 30 °C through cooling with a fan. After irradiation, the reaction vial was slowly opened to allow for a slow gas evolution. After gas evolution completed, the mixture was poured into a 50 mL round-bottom flask containing a 10% aq. NaOH solution (11 mL, 1.1 eq). This mixture was stirred at 25 °C for 24 hours. Et_2O was then added and the organic layer was separated. The organic solution was dried with Na_2SO_4, and concentrated under reduced pressure. The residue was purified by chromatography on silica gel eluting with Et_2O/pentane (5:95 (v/v)) to afford the title compound as a colorless oil (3.00 g, 11.2 mmol, 90%).

R_f = 0.12 (Et_2O/pentane, 5:95 (v/v)).

NMR Spectroscopy:

^{1}H NMR (500 MHz, $CDCl_3$, 298 K, δ): 0.53 (s, 1H), 0.18 (s, 27H).

^{13}C NMR (126 MHz, $CDCl_3$, 298 K, δ): –0.4.

HRMS-ESI (m/z) calc'd for $C_9H_{28}NaOSi_4^+$ [M+Na]$^+$, 287.1106; found, 287.1105; deviation: +0.9 ppm.

Celecoxib derivative S9

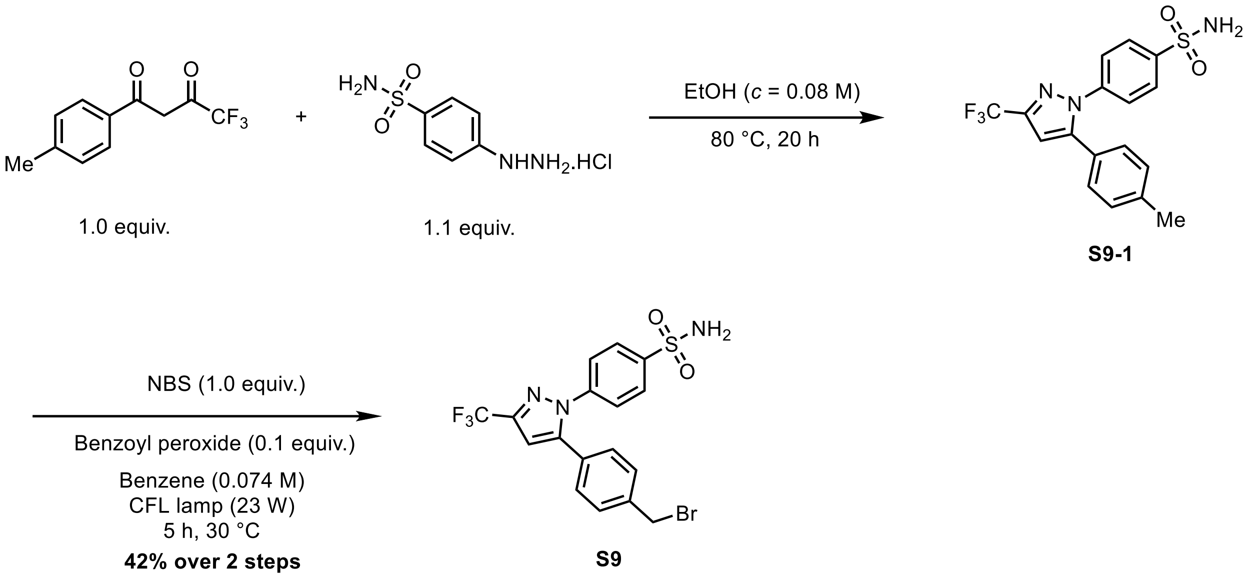

Celecoxib derivative **S9** was synthesized according to a reported procedure.[6] To a 100 mL round-bottom flask equipped with a magnetic stir bar were added 4,4,4-trifluoro-1-(p-tolyl)butane-1,3-dione (921 mg, 4.00 mmol, 1.00 equiv.), 4-hydrazineylbenzenesulfonamide hydrochloride (980 mg, 4.40 mmol, 1.10 equiv.), and EtOH (50 mL, c = 0.08 M). The mixture was refluxed at 80 °C for 20 h, and was concentrated under reduced pressure. The residue was dissolved in EtOAc (25 mL), and the mixture was extracted with water (10 mL) and then brine (10 mL). The organic layers were combined, dried over Na_2SO_4, and concentrated under reduced pressure to afford the white solid compound **S9-1**, which was directly used for the next step without further purification.

To a 50 mL round-bottom flask equipped with a magnetic stir bar were added **S9-1** (800 mg, 2.10 mmol, 1.00 equiv.), n-bromosuccinimide (374 mg, 2.10 mmol, 1.00 equiv.), benzoyl peroxide (51 mg, 0.21 mmol, 0.10 equiv.), and benzene (28.0 mL, c= 0.074M). Then, the flask was sealed with a septum-cap, and the reaction mixture was then irradiated with CFL lamp (23 W) for 5 h at 30 °C. The mixture was washed with water (10 mL) and brine (10 mL), and the combined organic phase was dried over Na_2SO_4, and concentrated under reduced pressure. The residue was purified by chromatography on silica gel eluting with EtOAc/pentane (1:9–3:7 (v/v)) to afford the title compound **S9** as a white solid (406 mg, 0.882 mmol, 42%).

R_f = 0.25 (EtOAc/pentane, 3:7 (v/v)).

NMR Spectroscopy:

^{1}H NMR (500 MHz, $CDCl_3$, 298 K, δ): 4.50 (s, 2H), 4.92 (s, 2H), 6.80 (s, 1H), 7.22 (d, J = 8.0 Hz, 2H), 7.42 (d, J = 8.0 Hz, 2H), 7.50 (dd, J = 6.7, 2.4 Hz, 2H), 7.94 (dd, J = 6.7, 2.4 Hz, 2H).

^{13}C NMR (126 MHz, $CDCl_3$, 298 K, δ): 144.4, 143.5, 142.2, 141.6, 139.3, 129.7, 129.1, 128.5, 127.5, 125.5, 120.9, 106.7, 32.2.

^{19}F NMR (471 MHz, $CDCl_3$, 298 K, δ): –62.49 (s).

HRMS-ESI (m/z) calc'd for $C_{17}H_{14}O_2N_3F_3SBr^+$ [M+H]$^+$, 459.9935; found, 459.9936; deviation: +0.4 ppm.

The NMR spectroscopy data was matched with the reported literature procedure.[6]

Pyrrole derivative S12

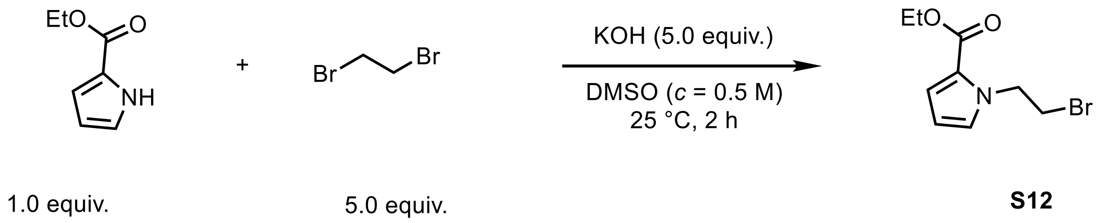

S12

To a 50 mL round-bottom flask equipped with a magnetic stir bar were added ethyl 1H-pyrrole-2-carboxylate (1.0 g, 8.0 mmol, 1.0 equiv.), KOH (2.0 g, 40 mmol, 5.0 equiv.), and DMSO (15 mL, c= 0.50 M). Then, the flask was sealed with a septum-cap, and the reaction mixture was stirred for 1 h at 25 °C. 1,2-Dibromoethane (3.4 mL, 40 mmol, 5.0 equiv.) was added to the mixture via syringe. The mixture was diluted with EtOAc (30 mL) and was extracted with brine (2 × 15 mL) and water (15 mL). The organic layers were combined, dried over Na_2SO_4, and concentrated under reduced pressure. The residue was purified by chromatography on silica gel eluting with EtOAc/pentane (1:9 (v/v)) to afford the title compound **S12** as a pale yellowish liquid (1.6 g, 6.4 mmol, 80%).

R_f = 0.32 (EtOAc/pentane, 1:4 (v/v)).

NMR Spectroscopy:

^{1}H NMR (500 MHz, $CDCl_3$, 298 K, δ): 7.00 (dd, J = 4.1, 1.9 Hz, 1H), 6.91 (t, J = 2.2 Hz, 1H), 6.14 (dt, J = 4.0, 2.0 Hz, 1H), 4.65 (t, J = 6.4 Hz, 2H), 4.28 (qd, J = 7.0, 1.4 Hz, 2H), 3.67 (td, J = 6.4, 1.4 Hz, 2H), 1.35 (td, J = 7.1, 1.4 Hz, 3H).

^{13}C NMR (126 MHz, $CDCl_3$, 298 K, δ): 161.2, 129.7, 121.6, 118.8, 108.2, 60.1, 50.8, 31.9, 14.5.

HRMS-EI (m/z) calc'd for $C_9H_{12}NO_2Br^+$ [M]$^+$, 245.0048; found, 245.0046; deviation: –1.0 ppm.

Flumequin derivative S13

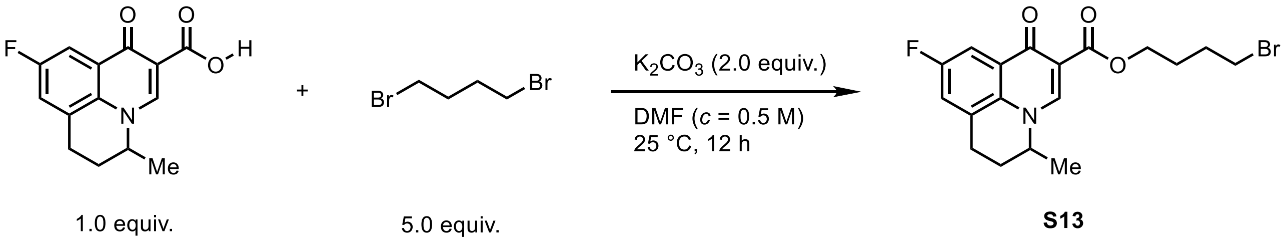

S13

To a 25 mL round-bottom flask equipped with a magnetic stir bar were added flumequin (600 mg, 2.30 mmol, 1.00 equiv.), K_2CO_3 (633 mg, 4.60 mmol, 2.00 equiv.), 1,4-dibromobutane (1.30 mL, 11.5 mmol, 5.00 equiv.), and DMF (4.6 mL, c= 0.50 M). Then, the flask was sealed with a septum-cap, and the reaction mixture was stirred for 12 h at 25 °C. The mixture was diluted with EtOAc (20 mL) and was extracted with brine (2 × 10 mL) and water (10 mL). The organic layers were combined, dried over Na_2SO_4, and concentrated under

reduced pressure. The residue was purified by chromatography on silica gel eluting with MeOH/DCM (0:100–3:97 (v/v)) to afford the title compound **S13** as a white solid (711 mg, 1.79 mmol, 78%).

R*f* = 0.22 (MeOH/DCM, 1:19 (v/v)).

NMR Spectroscopy:

^{1}H NMR (500 MHz, $CDCl_3$, 298 K, δ): 8.41 (s, 1H), 7.94 (dd, *J* = 9.1, 3.0 Hz, 1H), 7.17 (dd, *J* = 8.3, 3.0 Hz, 1H), 4.44 (tq, *J* = 6.9, 3.4 Hz, 1H), 4.38 – 4.28 (m, 2H), 3.49 (t, *J* = 6.5 Hz, 2H), 3.13 (ddd, *J* = 17.8, 12.8, 5.3 Hz, 1H), 2.98 (ddd, *J* = 17.1, 5.0, 2.9 Hz, 1H), 2.24 (tt, *J* = 13.2, 4.7 Hz, 1H), 2.12 (ddt, *J* = 13.7, 5.5, 2.9 Hz, 1H), 2.05 (dq, *J* = 8.6, 6.6 Hz, 2H), 1.93 (dq, *J* = 9.7, 6.4 Hz, 2H), 1.46 (d, *J* = 6.8 Hz, 3H).

^{13}C NMR (126 MHz, $CDCl_3$, 298 K, δ): 173.4 (d, *J* = 2.6 Hz), 165.9, 160.5, 158.5, 147.0, 132.1, 129.1 (d, *J* = 7.3 Hz), 120.1 (d, *J* = 24.5 Hz), 110.6 (d, *J* = 23.0 Hz), 109.8, 63.9, 57.2, 33.7, 29.4, 27.4, 26.3, 22.1, 20.4.

^{19}F NMR (471 MHz, $CDCl_3$, 298 K, δ): –115.75 (t, *J* = 8.4 Hz).

HRMS-EI (m/z) calc'd for $C_{18}H_{19}NO_3FBr^+$ $[M]^+$, 395.0529; found, 395.0526; deviation: –0.7 ppm.

Sulbactam derivative S14

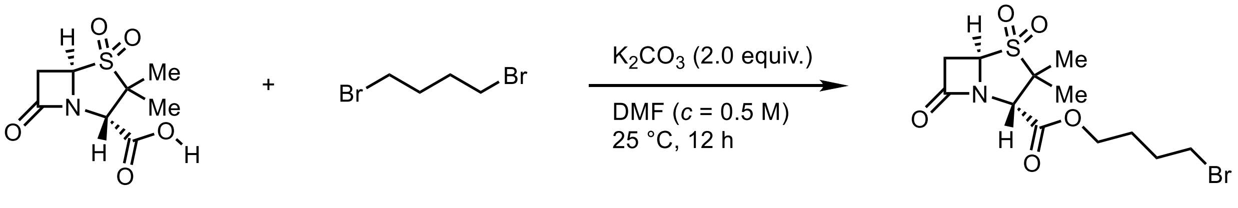

To a 25 mL round-bottom flask equipped with a magnetic stir bar were added sulbactam (225 mg, 0.96 mmol, 1.00 equiv.), K_2CO_3 (265 mg, 1.92 mmol, 2.00 equiv.), 1,4-dibromobutane (0.57 mL, 4.8 mmol, 5.0 equiv.), and DMF (2.0 mL, c= 0.50 M). Then, the flask was sealed with a septum-cap, and the reaction mixture was stirred for 12 h at 25 °C. The mixture was diluted with EtOAc (20 mL) and was extracted with brine (2 × 10 mL) and water (10 mL). The organic layers were combined, dried over Na_2SO_4, and concentrated under reduced pressure. The residue was purified by chromatography on silica gel eluting with EtOAc/pentane (1:4–2:3 (v/v)) to afford the title compound **S14** as a colorless liquid (297 mg, 0.806 mmol, 84%).

R*f* = 0.28 (EtOAc/pentane, 1:1 (v/v)).

NMR Spectroscopy:

^{1}H NMR (500 MHz, $CDCl_3$, 298 K, δ): 4.61 (dd, *J* = 4.3, 2.2 Hz, 1H), 4.38 (s, 1H), 4.25 (td, *J* = 6.4, 1.7 Hz, 2H), 3.53 – 3.39 (m, 4H), 1.95 (dqd, *J* = 7.5, 6.0, 1.4 Hz, 2H), 1.87 (dtd, *J* = 11.2, 7.5, 5.5 Hz, 2H), 1.61 (s, 3H), 1.42 (s, 3H).

^{13}C NMR (126 MHz, $CDCl_3$, 298 K, δ): 170.8, 167.0, 65.6, 63.3, 62.8, 61.2, 38.4, 32.8, 29.1, 27.2, 20.5,

18.7.

HRMS-ESI (m/z) calc'd for $C_{12}H_{18}O_5NNaSBr^+$ [M+Na]$^+$, 389.9980; found, 389.9981; deviation: +0.4 ppm.

Pregnenolone derivative S17

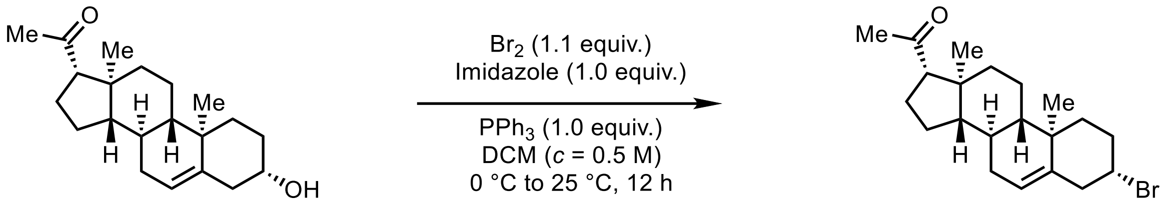

S17

Pregnenolone derivative **S17** was synthesized according to a reported procedure.[7] To a 25 mL round-bottom flask equipped with a magnetic stir bar were added Pregnenolone (500 mg, 1.58 mmol, 1.00 equiv., d.r. > 99:1), imidazole (108 mg, 1.58 mmol, 1.00 equiv.), PPh_3 (414 mg, 1.58 mmol, 1.00 equiv.), and DCM (3.0 mL, c = 0.50 M). Then, the flask was sealed with a septum-cap, and the reaction mixture cooled to 0 °C, and Br_2 (0.10 mL, 1.7 mmol, 1.1 equiv.) was added drop wise via syringe over 5 minutes at 0 °C. The reaction mixture was then allowed to warm to 25 °C and stirred for 12 h. The mixture was washed with brine (5 mL) and saturated aqueous Na_2SO_3 solution (5 mL), and the aqueous phase was extracted with DCM (2 × 10 mL). The combined organic phases were dried over anhydrous Na_2SO_4, filtered, and the solvents were evaporated under reduced pressure. The residue was purified by chromatography on silica gel eluting with EtOAc/pentane (0/100–1/24 (v/v)) to afford the title compound **S17** as a colorless solid (480 mg, 1.26 mmol, 80%, d.r. > 99:1).

R_f = 0.40 (EtOAc/pentane, 5:95 (v/v)).

NMR Spectroscopy:

^{1}H NMR (500 MHz, $CDCl_3$, 298 K, δ): 5.34–5.38 (m, 1H), 3.87–3.95(m, 1H), 2.70–2.78 (m, 1H), 2.56–2.62 (m, 1H), 22.52 (t, J = 8.7 Hz, 1H), 2.14–2.23 (m, 2H), 2.12 (s, 3H), 1.95–2.07 (m, 3H), 1.84–1.90 (m, 1H), 1.42–1.72 (m, 7H), 1.10–1.28 (m, 3H), 1.04 (s, 3H), 0.95–1.02 (m, 1H), 0.63 (s, 3H).

^{13}C NMR (126 MHz, $CDCl_3$, 298 K, δ): 209.4, 141.5, 122.0, 63.7, 56.8, 52.3, 50.0, 44.2, 44.0, 40.3, 38.8, 36.4, 34.3, 31.6, 31.6, 31.5, 24.5, 22.8, 20.9, 19.2, 13.2.

HRMS-EI (m/z) calc'd for $C_{21}H_{31}OBr^+$ [M]$^+$, 378.1553; found, 378.1552; deviation: –0.2 ppm.

The NMR spectroscopy data was matched with the reported literature procedure.[7]

Reaction condition optimization for Cu-photoredox-catalysed reductive cross-coupling of alkyl bromides with BCP-thianthrenium reagent 1b

Table S1. Screening of photocatalyst (PC) [a]

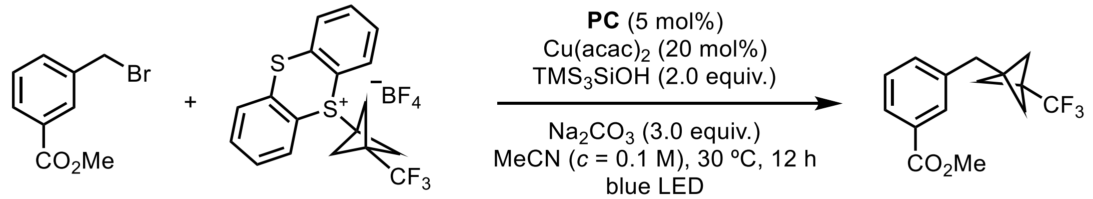

1a, 1.0 equiv. **1b**, 1.5 equiv. **1**

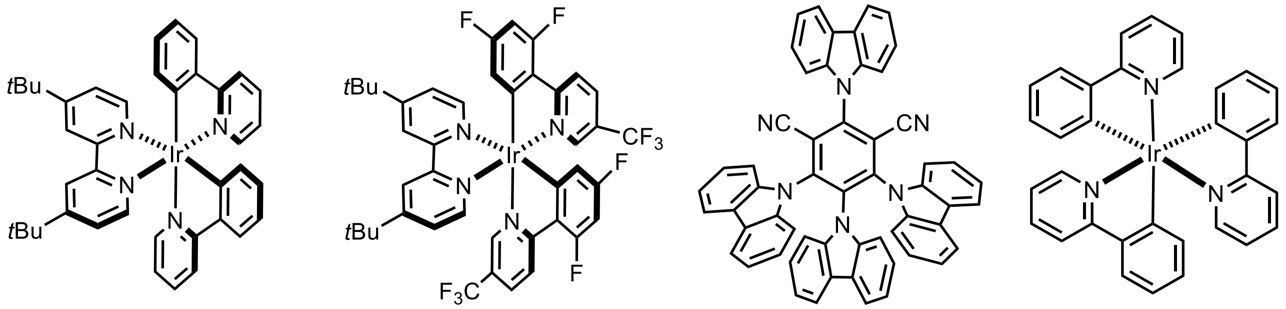

Ir(dtbbpy)(ppy)$_2$PF$_6$ Ir[dF(CF$_3$)ppy]$_2$(dtbbpy)PF$_6$ 4CzIPN Ir(ppy)$_3$

Entry	Photocatalyst (PC)	Yield [%][b]
1	Ir[(dtbbpy)(ppy)$_2$]PF$_6$	26
2	Ir[(dF(CF$_3$)$_2$(ppy)]$_2$(dtbbpy)PF$_6$	31
3	4CzIPN	39
4	Ir(ppy)$_3$	0
5	None	0

[a]Methyl 3-(bromomethyl)benzoate (0.05 mmol, 1 equiv.), **1b** (0.075 mmol, 1.5 equiv.), PC (5 mol%), Cu(acac)$_2$ (20 mol%), TMS$_3$SiOH (2.0 equiv.), Na$_2$CO$_3$ (3.0 equiv.), MeCN (*c* = 0.1 M), 30 °C, 12 h, Blue LED. [b]^{19}F NMR yield using PhCF$_3$ as an internal standard.

Table S2. Screening of base [a]

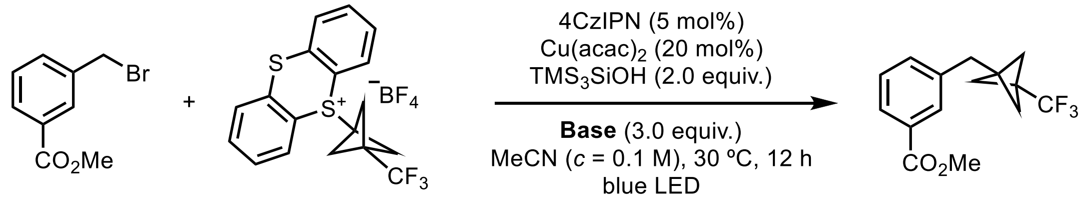

1a, 1.0 equiv. **1b**, 1.5 equiv. **1**

Entry	Base	Yield [%][b]
1	Na_2CO_3	39
2	Cs_2CO_3	7
3	K_2HPO_4	44
4	K_3PO_4	23
5	Leutidine	19

[a]Methyl 3-(bromomethyl)benzoate (0.05 mmol, 1 equiv.), **1b** (0.075 mmol, 1.5 equiv.), 4CzIPN (5 mol%), $Cu(acac)_2$ (20 mol%), TMS_3SiOH (2.0 equiv.), base (3.0 equiv.), MeCN (c = 0.1 M), 30 °C, 12 h, Blue LED. [b]^{19}F NMR yield using $PhCF_3$ as an internal standard.

Table S3. Screening of solvent [a]

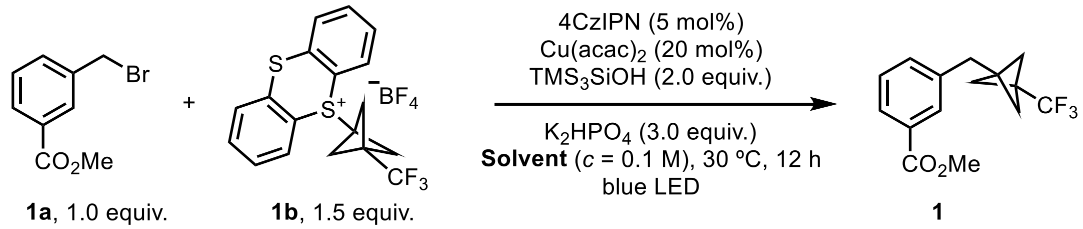

Entry	Sovent	Yield [%][b]
1	MeCN	39
2	Dioxane	18
3	DMA	55
4	THF	15
5	DCE	23
6	DMSO	36

[a]Methyl 3-(bromomethyl)benzoate (0.05 mmol, 1 equiv.), **1b** (0.075 mmol, 1.5 equiv.), 4CzIPN (5 mol%), $Cu(acac)_2$ (20 mol%), TMS_3SiOH (2.0 equiv.), K_2HPO_4 (3.0 equiv.), solvent (c = 0.1 M), 30 °C, 12 h, Blue LED. [b]^{19}F NMR yield using $PhCF_3$ as an internal standard.

Table S4. Screening of Cu-catalyst [a]

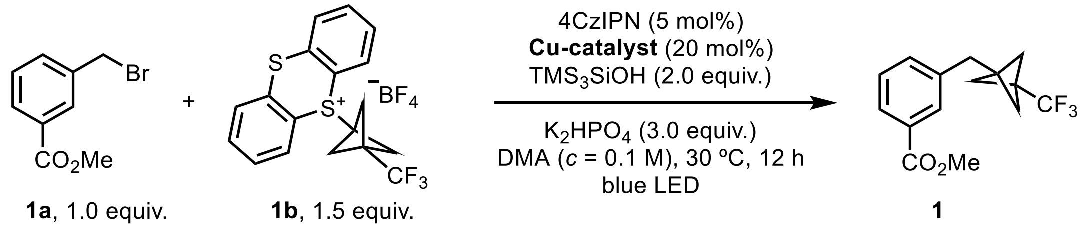

Entry	Cu-catalyst	Yield [%][b]
1	$Cu(acac)_2$	55
2	$CuBr_2$	50
3	$Cu(OAc)_2$	60
4	$CuCl_2$	44
5	CuCl	62
6	$Cu(MeCN)_4BF_4$	57

[a]Methyl 3-(bromomethyl)benzoate (0.05 mmol, 1 equiv.), **1b** (0.075 mmol, 1.5 equiv.), 4CzIPN (5 mol%), Cu-catalyst (20 mol%), TMS_3SiOH (2.0 equiv.), K_2HPO_4 (3.0 equiv.), DMA (c = 0.1 M), 30 °C, 12 h, Blue LED. [b]^{19}F NMR yield using $PhCF_3$ as an internal standard.

Table S5. Screening of ligand [a]

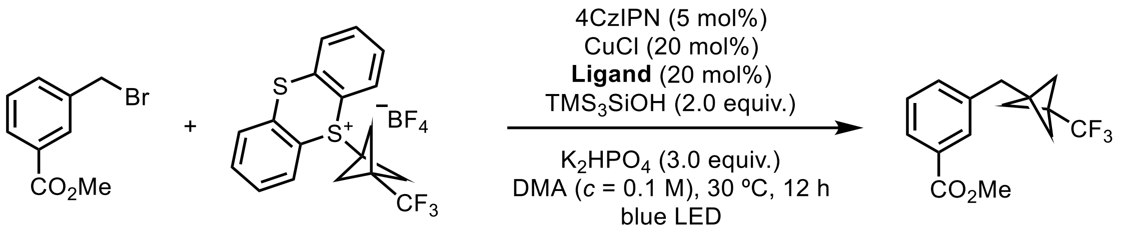

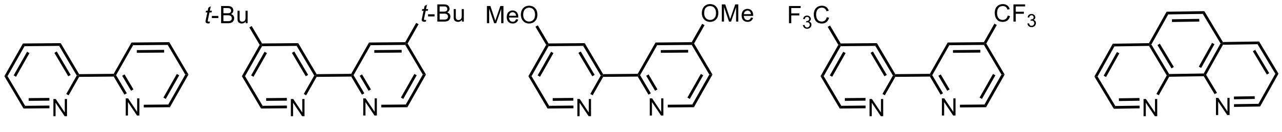

Entry	Base	Yield [%][b]
1	bpy	26
2	dtbbpy	50
3	di(OMe)bpy	44
4	di(CF_3)bpy	58
5	phen	10

[a]Methyl 3-(bromomethyl)benzoate (0.05 mmol, 1 equiv.), **1b** (0.075 mmol, 1.5 equiv.), 4CzIPN (5 mol%), CuCl (20 mol%), ligand (20 mol%), TMS_3SiOH (2.0 equiv.), base (3.0 equiv.), MeCN (c = 0.1 M), 30 °C, 12 h, Blue LED. [b]^{19}F NMR yield using $PhCF_3$ as an internal standard.

Table S6. Screening of Cu-catalyst loading [a]

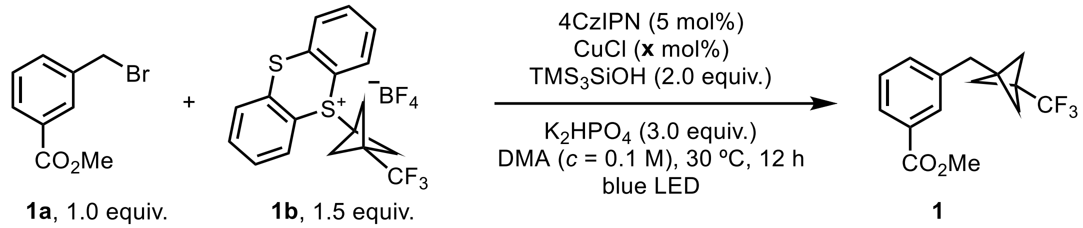

Entry	CuCl (x mol%)	Yield [%][b]
1	CuCl (10 mol%)	55
2	CuCl (20 mol%)	63
3	CuCl (40 mol%)	60
4	CuCl (60 mol%)	58
5	CuCl (100 mol%)	54

[a]Methyl 3-(bromomethyl)benzoate (0.05 mmol, 1 equiv.), **1b** (0.075 mmol, 1.5 equiv.), 4CzIPN (5 mol%), CuCl (x mol%), TMS_3SiOH (2.0 equiv.), K_2HPO_4 (3.0 equiv.), DMA (*c* = 0.1 M), 30 °C, 12 h, Blue LED. [b]^{19}F NMR yield using $PhCF_3$ as an internal standard.

Table S7. Screening of other base [a]

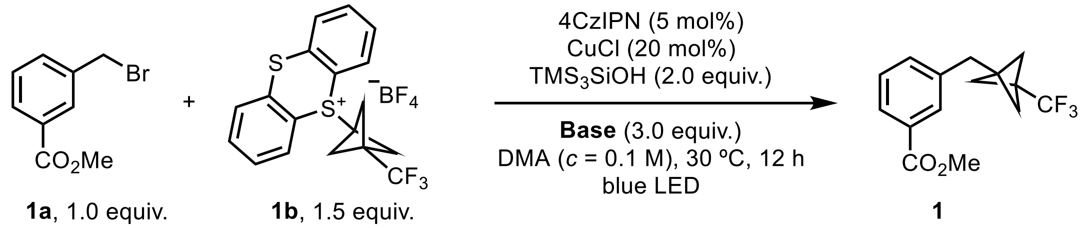

Entry	Base	Yield [%][b]
1	$NaHCO_3$	58
2	K_2CO_3	61
3	K_2HPO_4	63
4	KH_2PO_4	41
5	NaOAc	41
6	DBU	5
7	Na_2HPO_4	49

[a]Methyl 3-(bromomethyl)benzoate (0.05 mmol, 1 equiv.), **1b** (0.075 mmol, 1.5 equiv.), 4CzIPN (5 mol%), CuCl (20 mol%), TMS_3SiOH (2.0 equiv.), base (3.0 equiv.), DMA (*c* = 0.1 M), 30 °C, 12 h, Blue LED. [b]^{19}F NMR yield using $PhCF_3$ as an internal standard.

Table S8. Screening of solvent concentration [a]

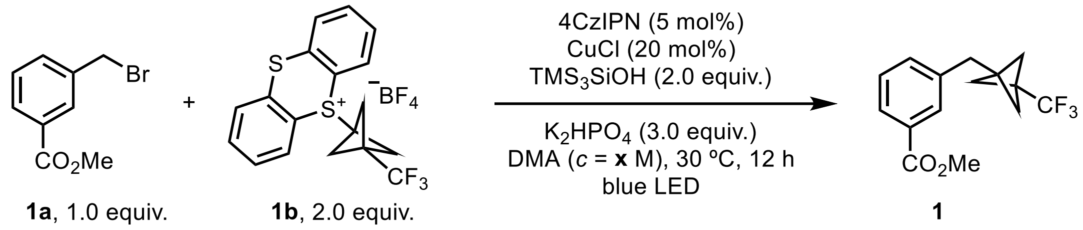

Entry	DMA (c = x M)	Yield [%][b]
1	DMA (c = 0.025 M)	58
2	DMA (c = 0.05 M)	61
3	DMA (c = 0.1 M)	69
4	DMA (c = 0.15 M)	63

[a]Methyl 3-(bromomethyl)benzoate (0.05 mmol, 1 equiv.), **1b** (0.1 mmol, 2.0 equiv.), 4CzIPN (5 mol%), CuCl (20 mol%), TMS_3SiOH (2.0 equiv.), K_2HPO_4 (3.0 equiv.), DMA (c = x M), 30 °C, 12 h, Blue LED. [b]^{19}F NMR yield using $PhCF_3$ as an internal standard.

Table S9. Screening of silicon reagent [a]

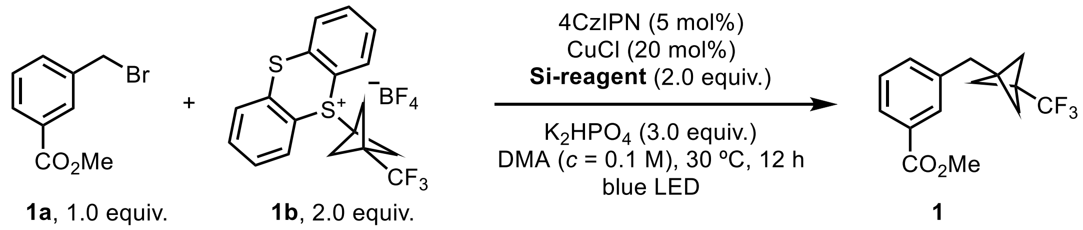

Entry	Si-reagent	Yield [%][b]
1	TMS_3SiH	33
2	Ph_3SiOH	12
3	TMS_3SiOH	69
4	Me_3SiOH	8
5	$(^tBuO)_3SiOH$	0
6	TMS_3SiOH[c]	71

[a]Methyl 3-(bromomethyl)benzoate (0.05 mmol, 1 equiv.), **1b** (0.1 mmol, 2.0 equiv.), 4CzIPN (5 mol%), CuCl (20 mol%), silicon reagent (2.0 equiv.), K_2HPO_4 (3.0 equiv.), DMA (c = 0.1 M), 30 °C, 12 h, Blue LED. [c]K_2HPO_4 (4.0 equiv.). [b]^{19}F NMR yield using $PhCF_3$ as an internal standard.

Table S10. Effect of slow addition and additives [a]

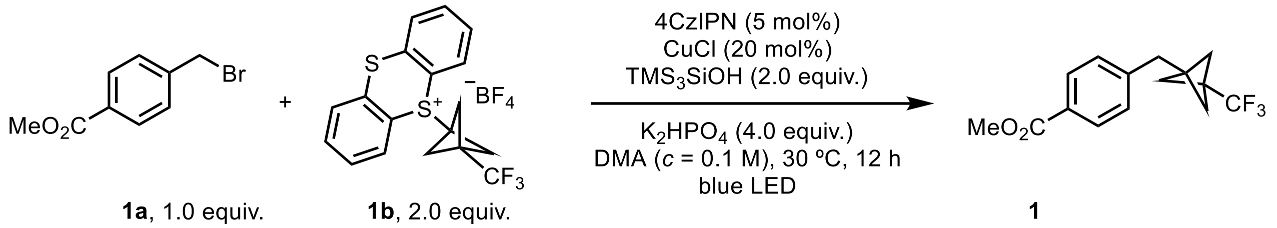

Entry	Condition	Yield [%][b]
1	No change	54
2	Slow addition of **1a** over 12h	36
3	Slow addition of TMS_3SiOH over 12 h	56
4	TBAB (2.0 equiv.) added as additive	35
5	Dipivaloylmethane (2.0 equiv.) added as additive	49

[a]Methyl 4-(bromomethyl)benzoate (0.05 mmol, 1 equiv.), **1b** (0.1 mmol, 2.0 equiv.), 4CzIPN (5 mol%), CuCl (20 mol%), TMS_3SiOH (2.0 equiv.), K_2HPO_4 (4.0 equiv.), DMA (*c* = 0.1 M), 30 °C, 12 h, Blue LED. [b]^{19}F NMR yield using $PhCF_3$ as an internal standard.

Table S11. Control experiments [a]

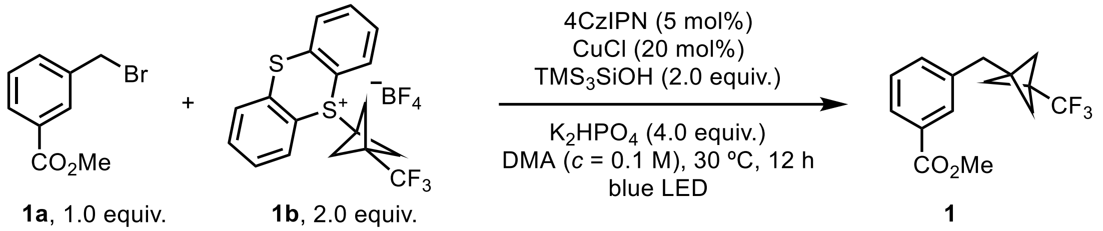

Entry	Condition	Yield [%][b]
1	No change	72
2	No CuCl	0
3	No TMS_3SiOH	0
4	No 4CzIPN	0
5	No K_2HPO_4	26
6	No blue LED	0
7	TMS_3SiH instead of TMS_3SiOH	33
8	$[Ir\{dF(CF_3)ppy\}_2(dtbbpy)]PF_6$ instead of 4CzIPN	31
9 [c]	$^nBu_3N$ instead of TMS_3SiOH, K_2HPO_4	45

10 [d]	^{n}Bu$_3$N and K_2HPO_4 instead of TMS$_3$SiOH	53

[a]Methyl 3-(bromomethyl)benzoate (0.05 mmol, 1 equiv.), **1b** (0.1 mmol, 2.0 equiv.), 4CzIPN (5 mol%), CuCl (20 mol%), TMS$_3$SiOH (2.0 equiv.), K_2HPO_4 (4.0 equiv.), DMA (c = 0.1 M), 30 °C, 12 h, Blue LED. [b]^{19}F NMR yield using $PhCF_3$ as an internal standard. [c] ^{n}Bu$_3$N (5.0 equiv.). [d] ^{n}Bu$_3$N (3.0 equiv.) and K_2HPO_4 (2.0 equiv.).

Bicyclo[1.1.1]pentylmethylarene 1

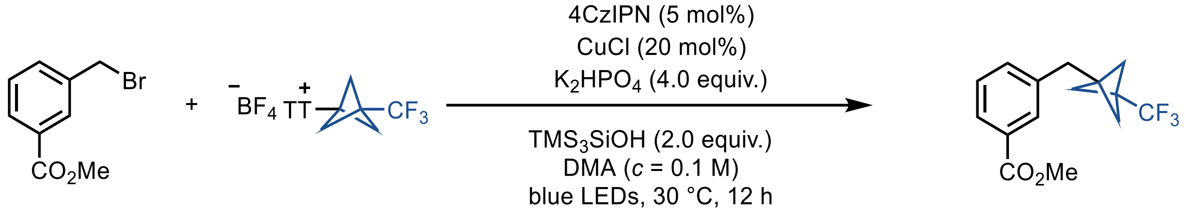

1.0 equiv. 2.0 equiv. **71%** **1**

Under nitrogen atmosphere, to a 4 mL borosilicate vial equipped with a magnetic stir bar were added methyl 3-(bromomethyl)benzoate (57.2 mg, 0.250 mmol, 1.00 equiv.), CF_3BCP-TT$^+$ BF_4^- **1b** (220 mg, 0.500 mmol, 2.00 equiv.), 4CzIPN (10.0 mg, 12.5 µmoL, 5.00 mol%), CuCl (5.0 mg, 50 µmoL, 20 mol%), K_2HPO_4 (174 mg, 1.00 mmol, 4.00 equiv.), TMS$_3$SiOH (132 mg, 0.500 mmol, 2.00 equiv.), and anhydrous DMA (2.5 mL, c = 0.10 M). The vial was sealed with a septum-cap. Then, the mixture was stirred for 10 min at 25 °C, and placed 5 cm away from two blue LEDs (Kessil A160WE Tuna Blue (460 nm), LED lighting, 40 W). The mixture was irradiated for 12 h while maintaining the temperature at approximately 30 °C through cooling with a fan. After irradiation, EtOAc (6 mL) was added to the reaction mixture, and the resultant solution was washed with brine (2 × 3 mL). The organic phase was dried over Na_2SO_4, filtered, and the solvent was removed under reduced pressure. The residue was purified by flash column chromatography on silica gel eluting with EtOAc/pentane (0:100–1:99 (v/v)) to afford the title compound **1** as a colorless solid (50.4 mg, 0.177 mmol, 71%).

R$_f$ = 0.35 (EtOAc/pentane, 1:19 (v/v)).

NMR Spectroscopy:

^{1}H NMR (500 MHz, $CDCl_3$, 298 K, δ): 7.90 (dt, J = 7.6, 1.4 Hz, 1H), 7.77 (t, J = 1.8 Hz, 1H), 7.37 (t, J = 7.6 Hz, 1H), 7.30 – 7.24 (m, 1H), 3.92 (s, 3H), 2.87 (s, 2H), 1.79 (s, 6H).

^{13}C NMR (126 MHz, $CDCl_3$, 298 K, δ): 167.2, 138.7, 133.5, 130.5, 130.0, 128.6, 127.7, 122.8 (q, J = 275.9 Hz), 52.2, 48.5 (q, J = 2.1 Hz), 39.8, 38.2, 37.8 (q, J = 38.2 Hz).

^{19}F NMR (471 MHz, $CDCl_3$, 298 K, δ): –73.37 (s).

HRMS-ESI (m/z) calc'd for $C_{15}H_{15}O_2F_3Na^+$ $[M+Na]^+$, 307.0918; found, 307.0916; deviation: –0.6 ppm.

Bicyclo[1.1.1]pentylmethylarene 2

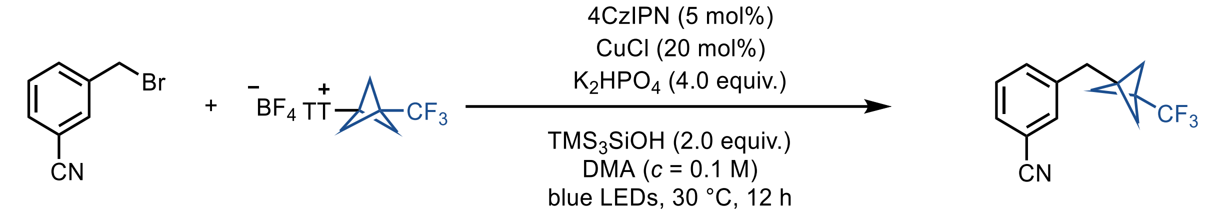

1.0 equiv. 2.0 equiv. **63%** **2**

Under nitrogen atmosphere, to a 4 mL borosilicate vial equipped with a magnetic stir bar were added 3-(bromomethyl)benzonitrile (49.0 mg, 0.250 mmol, 1.00 equiv.), CF_3BCP-TT^+ BF_4^- **1b** (220 mg, 0.500 mmol, 2.00 equiv.), 4CzIPN (10.0 mg, 12.5 µmoL, 5.00 mol%), CuCl (5.0 mg, 50 µmoL, 20 mol%), K_2HPO_4 (174 mg, 1.00 mmol, 4.00 equiv.), TMS_3SiOH (132 mg, 0.500 mmol, 2.00 equiv.), and anhydrous DMA (2.5 mL, *c* = 0.10 M). The vial was sealed with a septum-cap. Then, the mixture was stirred for 10 min at 25 °C, and placed 5 cm away from two blue LEDs (Kessil A160WE Tuna Blue (460 nm), LED lighting, 40 W). The mixture was irradiated for 12 h while maintaining the temperature at approximately 30 °C through cooling with a fan. After irradiation, EtOAc (6 mL) was added to the reaction mixture, and the resultant solution was washed with brine (2 × 3 mL). The organic phase was dried over Na_2SO_4, filtered, and the solvent was removed under reduced pressure. The residue was purified by flash column chromatography on silica gel eluting with EtOAc/pentane (0:100–1:49 (v/v)) to afford the title compound **2** as a colorless solid (39.5 mg, 0.157 mmol, 63%).

R_f = 0.38 (EtOAc/pentane, 1:19 (v/v)).

NMR Spectroscopy:

^{1}H NMR (500 MHz, $CDCl_3$, 298 K, δ): 7.56 – 7.50 (m, 1H), 7.41 (t, *J* = 7.7 Hz, 1H), 7.37 (d, *J* = 1.9 Hz, 1H), 7.32 (dt, *J* = 7.8, 1.6 Hz, 1H), 2.86 (s, 2H), 1.79 (s, 6H).

^{13}C NMR (126 MHz, $CDCl_3$, 298 K, δ): 139.8, 133.4, 132.3, 130.3, 129.4, 122.7 (q, *J* = 275.7 Hz), 118.9, 112.7, 48.5 (q, *J* = 2.4 Hz), 39.5, 38.0, 37.8 (q, *J* = 37.2 Hz).

^{19}F NMR (471 MHz, $CDCl_3$, 298 K, δ): –73.36 (s).

HRMS-ESI (m/z) calc'd for $C_{14}H_{12}NF_3Na^+$ [M+Na]$^+$, 274.0812; found, 274.0814; deviation: +0.7 ppm.

Bicyclo[1.1.1]pentylmethylpyridine 3

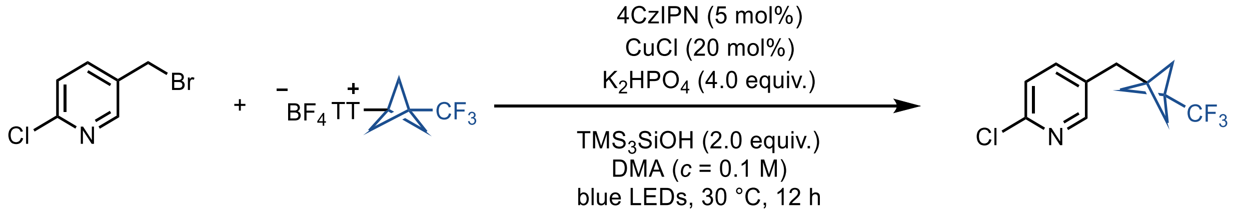

1.0 equiv. 2.0 equiv. **57%** **3**

Under nitrogen atmosphere, to a 4 mL borosilicate vial equipped with a magnetic stir bar were added 5-

(bromomethyl)-2-chloropyridine (51.6 mg, 0.250 mmol, 1.00 equiv.), $CF_3BCP\text{-}TT^+$ BF_4^- **1b** (220 mg, 0.500 mmol, 2.00 equiv.), 4CzIPN (10.0 mg, 12.5 µmoL, 5.00 mol%), CuCl (5.0 mg, 50 µmoL, 20 mol%), K_2HPO_4 (174 mg, 1.00 mmol, 4.00 equiv.), TMS_3SiOH (132 mg, 0.500 mmol, 2.00 equiv.), and anhydrous DMA (2.5 mL, *c* = 0.10 M). The vial was sealed with a septum-cap. Then, the mixture was stirred for 10 min at 25 °C, and placed 5 cm away from two blue LEDs (Kessil A160WE Tuna Blue (460 nm), LED lighting, 40 W). The mixture was irradiated for 12 h while maintaining the temperature at approximately 30 °C through cooling with a fan. After irradiation, EtOAc (6 mL) was added to the reaction mixture, and the resultant solution was washed with brine (2 × 3 mL). The organic phase was dried over Na_2SO_4, filtered, and the solvent was removed under reduced pressure. The residue was purified by flash column chromatography on silica gel eluting with EtOAc/pentane (1:99–3:97 (v/v)) to afford the title compound **3** as a colorless solid (37.2 mg, 0.142 mmol, 57%).

R_f = 0.25 (EtOAc/pentane, 1:9 (v/v)).

NMR Spectroscopy:

^{1}H NMR (500 MHz, $CDCl_3$, 298 K, δ): 8.13 (d, *J* = 2.5 Hz, 1H), 7.38 (dd, *J* = 8.1, 2.5 Hz, 1H), 7.27 (d, *J* = 8.1 Hz, 1H), 2.82 (s, 2H), 1.81 (s, 6H).

^{13}C NMR (126 MHz, $CDCl_3$, 298 K, δ): 149.8, 139.1, 132.6, 124.1, 122.7 (q, *J* = 275.8 Hz), 48.5 (q, *J* = 2.4 Hz), 39.4, 37.9 (q, *J* = 38.4 Hz), 34.7.

^{19}F NMR (471 MHz, $CDCl_3$, 298 K, δ): –73.39 (s).

HRMS-CI (m/z) calc'd for $C_{12}H_{12}NClF_3^+$ $[M+H]^+$, 262.0605; found, 262.0604; deviation: –0.4 ppm.

Bicyclo[1.1.1]pentylmethylarene 4

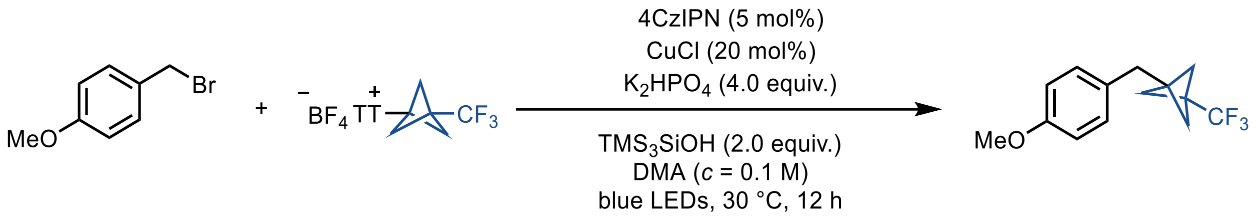

1.0 equiv. 2.0 equiv. **66%** **4**

Under nitrogen atmosphere, to a 4 mL borosilicate vial equipped with a magnetic stir bar were added 1-(bromomethyl)-4-methoxybenzene (50.2 mg, 0.250 mmol, 1.00 equiv.), $CF_3BCP\text{-}TT^+$ BF_4^- **1b** (220 mg, 0.500 mmol, 2.00 equiv.), 4CzIPN (10.0 mg, 12.5 µmoL, 5.00 mol%), CuCl (5.0 mg, 50 µmoL, 20 mol%), K_2HPO_4 (174 mg, 1.00 mmol, 4.00 equiv.), TMS_3SiOH (132 mg, 0.500 mmol, 2.00 equiv.), and anhydrous DMA (2.5 mL, *c* = 0.10 M). The vial was sealed with a septum-cap. Then, the mixture was stirred for 10 min at 25 °C, and placed 5 cm away from two blue LEDs (Kessil A160WE Tuna Blue (460 nm), LED lighting, 40 W). The mixture was irradiated for 12 h while maintaining the temperature at approximately 30 °C through cooling with a fan. After irradiation, EtOAc (6 mL) was added to the reaction mixture, and the resultant solution was washed with brine (2 × 3 mL). The organic phase was dried over Na_2SO_4, filtered, and the solvent was

removed under reduced pressure. The residue was purified by flash column chromatography on silica gel eluting with pentane to afford the title compound **4** as a pale yellowish oil (42.3 mg, 0.165 mmol, 66%).

R*f* = 0.45 (EtOAc/pentane, 1:19 (v/v)).

NMR Spectroscopy:

^{1}**H NMR** (600 MHz, $CDCl_3$, 298 K, δ): 7.00 (d, *J* = 8.6 Hz, 2H), 6.84 (d, *J* = 8.6 Hz, 2H), 3.80 (s, 3H), 2.75 (s, 2H), 1.77 (s, 6H).

^{13}**C NMR** (151 MHz, $CDCl_3$, 298 K, δ): 158.2, 130.5, 129.9, 123.0 (q, *J* = 275.8 Hz), 113.9, 55.3, 48.5 (q, *J* = 2.0 Hz), 40.2 (q, *J* = 1.7 Hz), 37.7 (q, *J* = 38.4 Hz), 37.5.

^{19}**F NMR** (565 MHz, $CDCl_3$, 298 K, δ): –73.38 (s).

HRMS-EI (m/z) calc'd for $C_{14}H_{15}OF_3^+$ $[M]^+$, 256.1070; found, 256.1069; deviation: –0.3 ppm.

Bicyclo[1.1.1]pentylmethylarene 5

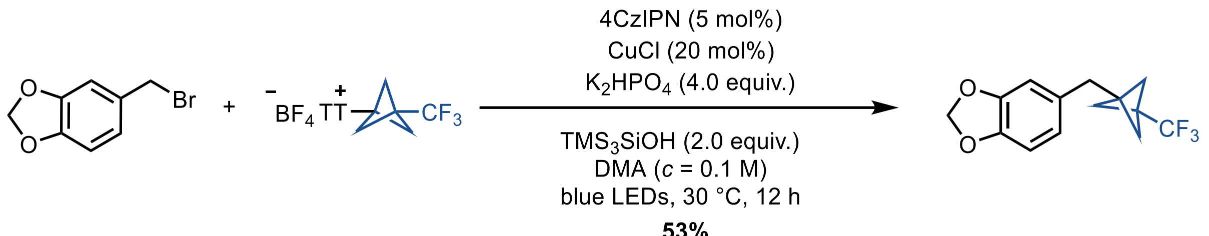

1.0 equiv. 2.0 equiv. **5**

Under nitrogen atmosphere, to a 4 mL borosilicate vial equipped with a magnetic stir bar were added 5-(bromomethyl)-1,3-benzodioxole (54.0 mg, 0.250 mmol, 1.00 equiv.), $CF_3BCP\text{-}TT^+$ BF_4^- **1b** (220 mg, 0.500 mmol, 2.00 equiv.), 4CzIPN (10.0 mg, 12.5 µmoL, 5.00 mol%), CuCl (5.0 mg, 50 µmoL, 20 mol%), K_2HPO_4 (174 mg, 1.00 mmol, 4.00 equiv.), TMS_3SiOH (132 mg, 0.500 mmol, 2.00 equiv.), and anhydrous DMA (2.5 mL, *c* = 0.10 M). The vial was sealed with a septum-cap. Then, the mixture was stirred for 10 min at 25 °C, and placed 5 cm away from two blue LEDs (Kessil A160WE Tuna Blue (460 nm), LED lighting, 40 W). The mixture was irradiated for 12 h while maintaining the temperature at approximately 30 °C through cooling with a fan. After irradiation, EtOAc (6 mL) was added to the reaction mixture, and the resultant solution was washed with brine (2 × 3 mL). The organic phase was dried over Na_2SO_4, filtered, and the solvent was removed under reduced pressure. The residue was purified by flash column chromatography on silica gel eluting with pentane to afford the title compound **5** as a colorless oil (36.0 mg, 0.132 mmol, 53%).

R*f* = 0.38 (EtOAc/pentane, 1:19 (v/v)).

NMR Spectroscopy:

^{1}**H NMR** (500 MHz, $CDCl_3$, 298 K, δ): 6.75 (d, *J* = 7.8 Hz, 1H), 6.57 (d, *J* = 1.7 Hz, 1H), 6.53 (dd, *J* = 7.8, 1.7 Hz, 1H), 5.94 (s, 2H), 2.73 (s, 2H), 1.79 (s, 6H).

^{13}**C NMR** (126 MHz, $CDCl_3$, 298 K, δ): 147.7, 146.1, 132.1, 122.9 (q, *J* = 275.9 Hz), 121.9, 109.3, 108.3, 100.9, 48.5 (q, *J* = 2.4 Hz), 40.0, 38.1, 37.6 (q, *J* = 38.1 Hz).

^{19}F NMR (471 MHz, $CDCl_3$, 298 K, δ): –73.32 (s).

HRMS-EI (m/z) calc'd for $C_{14}H_{13}O_2F_3^+$ $[M]^+$, 270.0865; found, 270.0862; deviation: –1.1 ppm.

Bicyclo[1.1.1]pentylmethylarene 6

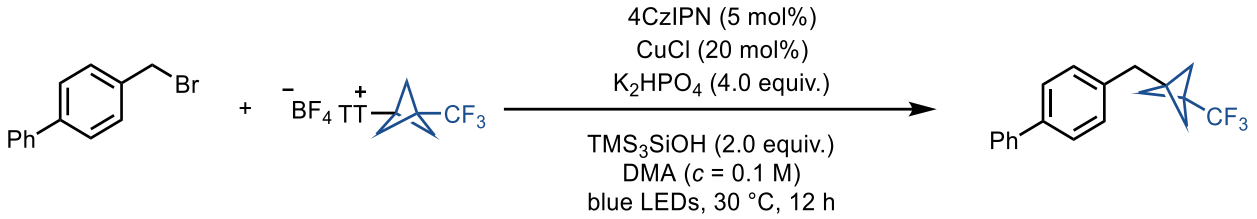

1.0 equiv. 2.0 equiv. **72%** **6**

Under nitrogen atmosphere, to a 4 mL borosilicate vial equipped with a magnetic stir bar were added 4-(bromomethyl)-1,1'-biphenyl (61.7 mg, 0.250 mmol, 1.00 equiv.), CF_3BCP-TT$^+$ BF_4^- **1b** (220 mg, 0.500 mmol, 2.00 equiv.), 4CzIPN (10.0 mg, 12.5 µmoL, 5.00 mol%), CuCl (5.0 mg, 50 µmoL, 20 mol%), K_2HPO_4 (174 mg, 1.00 mmol, 4.00 equiv.), TMS_3SiOH (132 mg, 0.500 mmol, 2.00 equiv.), and anhydrous DMA (2.5 mL, c = 0.10 M). The vial was sealed with a septum-cap. Then, the mixture was stirred for 10 min at 25 °C, and placed 5 cm away from two blue LEDs (Kessil A160WE Tuna Blue (460 nm), LED lighting, 40 W). The mixture was irradiated for 12 h while maintaining the temperature at approximately 30 °C through cooling with a fan. After irradiation, EtOAc (6 mL) was added to the reaction mixture, and the resultant solution was washed with brine (2 × 3 mL). The organic phase was dried over Na_2SO_4, filtered, and the solvent was removed under reduced pressure. The residue was purified by flash column chromatography on silica gel eluting with pentane to afford the title compound **6** as a colorless solid (54.4 mg, 0.180 mmol, 72%).

R_f = 0.45 (pentane).

NMR Spectroscopy:

^{1}H NMR (500 MHz, $CDCl_3$, 298 K, δ): 7.62 (d, J = 7.4 Hz, 2H), 7.56 (d, J = 7.9 Hz, 2H), 7.47 (t, J = 7.6 Hz, 2H), 7.37 (t, J = 7.4 Hz, 1H), 7.18 (d, J = 7.9 Hz, 2H), 2.88 (s, 2H), 1.85 (s, 6H).

^{13}C NMR (126 MHz, $CDCl_3$, 298 K, δ): 141.0, 139.3, 137.4, 129.4, 128.9, 127.3, 127.1, 123.0 (q, J = 275.9 Hz), 48.6 (q, J = 2.1 Hz), 40.0 (d, J = 1.8 Hz), 38.1, 37.7 (q, J = 38.1 Hz).

^{19}F NMR (471 MHz, $CDCl_3$, 298 K, δ): –73.25 (s).

HRMS-EI (m/z) calc'd for $C_{19}H_{17}F_3^+$ $[M]^+$, 302.1279; found, 302.1276; deviation: –0.7 ppm.

Bicyclo[1.1.1]pentylmethylarene 7

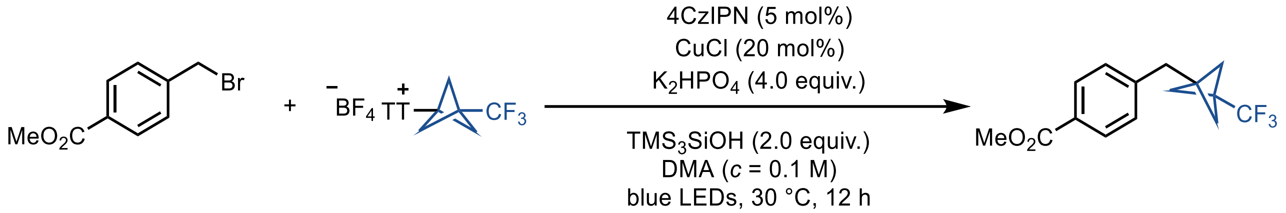

1.0 equiv. 2.0 equiv. **55%** **7**

Under nitrogen atmosphere, to a 4 mL borosilicate vial equipped with a magnetic stir bar were added methyl 4-(bromomethyl)benzoate (57.2 mg, 0.250 mmol, 1.00 equiv.), CF_3BCP-TT$^+$ BF_4^- **1b** (220 mg, 0.500 mmol, 2.00 equiv.), 4CzIPN (10.0 mg, 12.5 µmoL, 5.00 mol%), CuCl (5.0 mg, 50 µmoL, 20 mol%), K_2HPO_4 (174 mg, 1.00 mmol, 4.00 equiv.), TMS_3SiOH (132 mg, 0.500 mmol, 2.00 equiv.), and anhydrous DMA (2.5 mL, *c* = 0.10 M). The vial was sealed with a septum-cap. Then, the mixture was stirred for 10 min at 25 °C, and placed 5 cm away from two blue LEDs (Kessil A160WE Tuna Blue (460 nm), LED lighting, 40 W). The mixture was irradiated for 12 h while maintaining the temperature at approximately 30 °C through cooling with a fan. After irradiation, EtOAc (6 mL) was added to the reaction mixture, and the resultant solution was washed with brine (2 × 3 mL). The organic phase was dried over Na_2SO_4, filtered, and the solvent was removed under reduced pressure. The residue was purified by flash column chromatography on silica gel eluting with EtOAc/pentane (0:100–1:99 (v/v)) to afford the title compound **7** as a colorless solid (39.0 mg, 0.137 mmol, 55%).

R_f = 0.35 (EtOAc/pentane, 1:19 (v/v)).

NMR Spectroscopy:

^{1}H NMR (500 MHz, $CDCl_3$, 298 K, δ): 7.97 (d, *J* = 7.7 Hz, 2H), 7.15 (d, *J* = 7.9 Hz, 2H), 3.91 (s, 3H), 2.87 (s, 2H), 1.78 (s, 6H).

^{13}C NMR (151 MHz, $CDCl_3$, 298 K, δ): 167.1, 143.7, 129.9, 129.0, 128.4, 122.8 (q, *J* = 275.8 Hz), 52.1, 48.6 (q, *J* = 2.1 Hz), 39.6 (q, *J* = 1.7 Hz), 38.5, 37.8 (q, *J* = 38.3 Hz).

^{19}F NMR (471 MHz, $CDCl_3$, 298 K, δ): –73.37 (s).

HRMS-CI (m/z) calc'd for $C_{15}H_{16}O_2F_3^+$ [M+H]$^+$, 285.1095; found, 285.1096; deviation: +0.4 ppm.

Bicyclo[1.1.1]pentylmethylarene 8

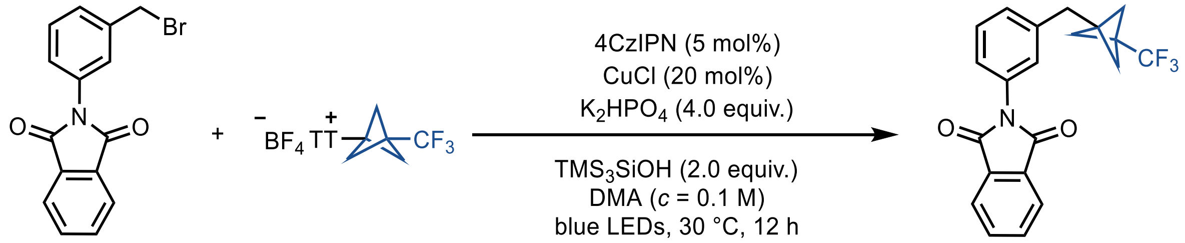

1.0 equiv. 2.0 equiv. **68%** **8**

Under nitrogen atmosphere, to a 4 mL borosilicate vial equipped with a magnetic stir bar were added 2-(3-(bromomethyl)phenyl)isoindoline-1,3-dione (79.0 mg, 0.250 mmol, 1.00 equiv.), CF_3BCP-TT$^+$ BF_4^- **1b** (220 mg, 0.500 mmol, 2.00 equiv.), 4CzIPN (10.0 mg, 12.5 μmoL, 5.00 mol%), CuCl (5.0 mg, 50 μmoL, 20 mol%), K_2HPO_4 (174 mg, 1.00 mmol, 4.00 equiv.), TMS_3SiOH (132 mg, 0.500 mmol, 2.00 equiv.), and anhydrous DMA (2.5 mL, *c* = 0.10 M). The vial was sealed with a septum-cap. Then, the mixture was stirred for 10 min at 25 °C, and placed 5 cm away from two blue LEDs (Kessil A160WE Tuna Blue (460 nm), LED lighting, 40 W). The mixture was irradiated for 12 h while maintaining the temperature at approximately 30 °C through cooling with a fan. After irradiation, EtOAc (6 mL) was added to the reaction mixture, and the resultant solution was washed with brine (2 × 3 mL). The organic phase was dried over Na_2SO_4, filtered, and the solvent was removed under reduced pressure. The residue was purified by flash column chromatography on silica gel eluting with EtOAc/pentane (0:100–1:24 (v/v)) to afford the title compound **8** as a colorless oil (63.1 mg, 0.170 mmol, 68%).

$\mathbf{R}_f$ = 0.25 (EtOAc/pentane, 1:9 (v/v)).

NMR Spectroscopy:

^{1}H NMR (500 MHz, $CDCl_3$, 298 K, δ): 7.95 (dd, *J* = 5.5, 3.1 Hz, 2H), 7.79 (dd, *J* = 5.5, 3.0 Hz, 2H), 7.44 (t, *J* = 7.8 Hz, 1H), 7.32 (d, *J* = 8.0 Hz, 1H), 7.18 (s, 1H), 7.12 (d, *J* = 7.6 Hz, 1H), 2.88 (s, 2H), 1.83 (s, 6H).

^{13}C NMR (126 MHz, $CDCl_3$, 298 K, δ): 167.3, 139.4, 134.5, 131.8, 129.2, 128.6, 127.0, 124.4, 123.8, 122.9 (q, *J* = 275.9 Hz), 48.6 (q, *J* = 2.4 Hz), 39.8, 38.3, 37.8 (q, *J* = 38.1 Hz).

^{19}F NMR (471 MHz, $CDCl_3$, 298 K, δ): –73.25 (s).

HRMS-ESI (m/z) calc'd for $C_{21}H_{16}NO_2NaF_3^+$ [M+Na]$^+$, 394.1024; found, 394.1025; deviation: +0.3 ppm.

Bicyclo[1.1.1]pentylmethylarene 9

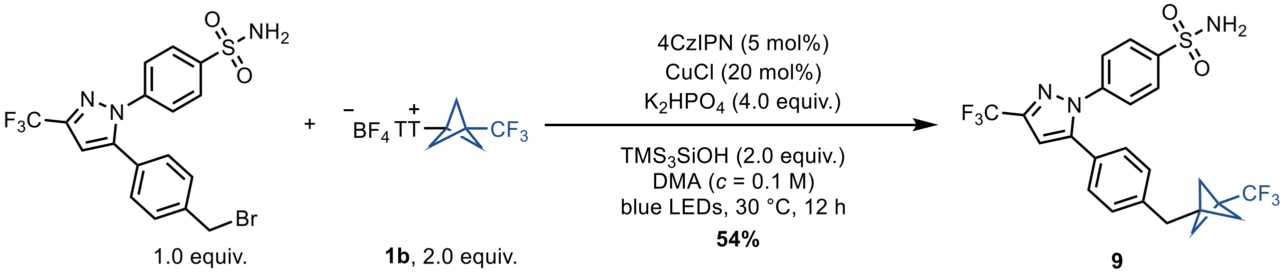

Under nitrogen atmosphere, to a 4 mL borosilicate vial equipped with a magnetic stir bar were added 4-(5-(4-(bromomethyl)phenyl)-3-(trifluoromethyl)-1H-pyrazol-1-yl)benzenesulfonamide (115 mg, 0.250 mmol, 1.00 equiv.), CF_3BCP-TT$^+$ BF_4^- **1b** (220 mg, 0.500 mmol, 2.00 equiv.), 4CzIPN (10.0 mg, 12.5 μmoL, 5.00 mol%), CuCl (5.0 mg, 50 μmoL, 20 mol%), K_2HPO_4 (174 mg, 1.00 mmol, 4.00 equiv.), TMS_3SiOH (132 mg, 0.500 mmol, 2.00 equiv.), and anhydrous DMA (2.5 mL, *c* = 0.10 M). The vial was sealed with a septum-cap. Then, the mixture was stirred for 10 min at 25 °C, and placed 5 cm away from two blue LEDs (Kessil A160WE Tuna Blue (460 nm), LED lighting, 40 W). The mixture was irradiated for 12 h while maintaining the

temperature at approximately 30 °C through cooling with a fan. After irradiation, EtOAc (6 mL) was added to the reaction mixture, and the resultant solution was washed with brine (2 × 3 mL). The organic phase was dried over Na_2SO_4, filtered, and the solvent was removed under reduced pressure. The residue was purified by flash column chromatography on silica gel eluting with EtOAc/pentane (1:9–1:3 (v/v)) to afford the title compound **9** as a colorless oil (69.6 mg, 0.135 mmol, 54%).

R*f* = 0.5 (EtOAc/pentane, 2:3 (v/v)).

NMR Spectroscopy:

^{1}H NMR (500 MHz, $CDCl_3$, 298 K, δ): 7.89 (d, *J* = 8.8 Hz, 2H), 7.46 (d, *J* = 8.8 Hz, 2H), 7.16 (d, *J* = 8.2 Hz, 2H), 7.09 (d, *J* = 8.3 Hz, 2H), 6.77 (s, 1H), 5.10 (s, 1H), 2.84 (s, 2H), 1.79 (s, 6H).

^{13}C NMR (126 MHz, $CDCl_3$, 298 K, δ): 145.1, 144.3 (q, *J* = 38.9 Hz), 142.6, 141.5, 140.2, 129.6, 129.0, 127.6, 126.7, 125.6, 123.0 (q, *J* = 275.9 Hz), 121.7, 106.6, 48.6 (d, *J* = 3.0 Hz), 39.6, 38.1, 37.8 (q, *J* = 38.3 Hz).

^{19}F NMR (471 MHz, $CDCl_3$, 298 K, δ): –62.43 (s, 3F), –73.27 (s, 3F).

HRMS-ESI (m/z) calc'd for $C_{23}H_{19}O_2N_3NaF_6S^+$ [M+Na]$^+$, 538.0995; found, 538.0994; deviation: –0.2 ppm.

Bicyclo[1.1.1]pentylalkane 10

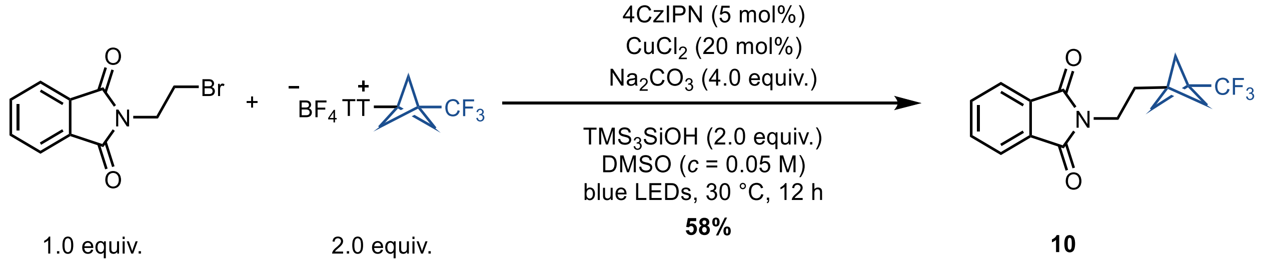

Under nitrogen atmosphere, to a 8 mL borosilicate vial equipped with a magnetic stir bar were added 2-(2-bromoethyl)isoindoline-1,3-dione (63.5 mg, 0.250 mmol, 1.00 equiv.), CF_3BCP-TT$^+$ BF_4^- **1b** (220 mg, 0.500 mmol, 2.00 equiv.), 4CzIPN (10.0 mg, 12.5 µmoL, 5.00 mol%), $CuCl_2$ (6.7 mg, 50 µmoL, 20 mol%), Na_2CO_3 (106 mg, 1.00 mmol, 4.00 equiv.), TMS_3SiOH (132 mg, 0.500 mmol, 2.00 equiv.), and anhydrous DMSO (5.0 mL, *c* = 0.05 M). The vial was sealed with a septum-cap. Then, the mixture was stirred for 10 min at 25 °C, and placed 5 cm away from two blue LEDs (Kessil A160WE Tuna Blue (460 nm), LED lighting, 40 W). The mixture was irradiated for 12 h while maintaining the temperature at approximately 30 °C through cooling with a fan. After irradiation, EtOAc (10 mL) was added to the reaction mixture, and the resultant solution was washed with brine (2 × 5 mL). The organic phase was dried over Na_2SO_4, filtered, and the solvent was removed under reduced pressure. The residue was purified by flash column chromatography on silica gel eluting with EtOAc/pentane (0:100–1:24 (v/v)) to afford the title compound **10** as a colorless solid (44.8 mg, 0.145 mmol, 58%).

R*f* = 0.32 (EtOAc/pentane, 1:9 (v/v)).

NMR Spectroscopy:

^{1}H NMR (500 MHz, $CDCl_3$, 298 K, δ): 7.85 (dd, *J* = 5.5, 3.1 Hz, 2H), 7.72 (dd, *J* = 5.4, 3.0 Hz, 2H), 3.68 (t, *J* = 7.4 Hz, 2H), 1.96 (t, *J* = 7.4 Hz, 2H), 1.89 (s, 6H).

^{13}C NMR (126 MHz, $CDCl_3$, 298 K, δ): 168.3, 134.1, 132.1, 123.4, 122.6 (q, *J* = 275.7 Hz), 48.8 (q, *J* = 2.2 Hz), 37.7 (q, *J* = 1.6 Hz), 37.4 (q, *J* = 38.1 Hz), 35.5, 29.5.

^{19}F NMR (471 MHz, $CDCl_3$, 298 K, δ): –73.37 (s).

HRMS-ESI (m/z) calc'd for $C_{16}H_{14}NO_2F_3Na^+$ [M+Na]$^+$, 332.0866; found, 332.0868; deviation: +0.7 ppm.

Bicyclo[1.1.1]pentylalkane 11

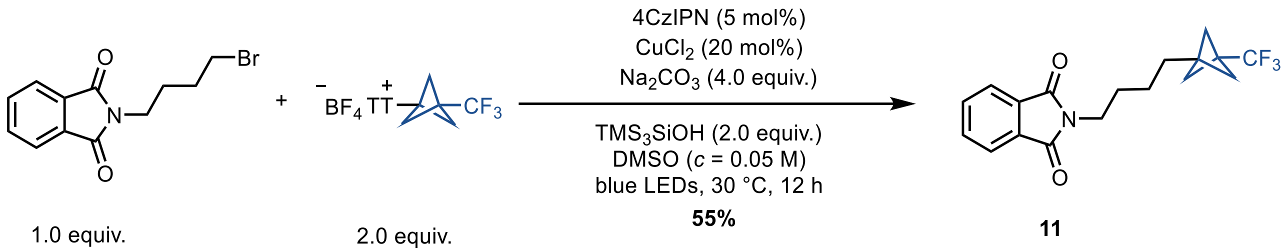

1.0 equiv. 2.0 equiv. **11**

Under nitrogen atmosphere, to a 8 mL borosilicate vial equipped with a magnetic stir bar were added 2-(4-bromobutyl)isoindoline-1,3-dione (70.5 mg, 0.250 mmol, 1.00 equiv.), CF_3BCP-TT$^+$ BF_4^- **1b** (220 mg, 0.500 mmol, 2.00 equiv.), 4CzIPN (10.0 mg, 12.5 µmoL, 5.00 mol%), $CuCl_2$ (6.7 mg, 50 µmoL, 20 mol%), Na_2CO_3 (106 mg, 1.00 mmol, 4.00 equiv.), TMS_3SiOH (132 mg, 0.500 mmol, 2.00 equiv.), and anhydrous DMSO (5.0 mL, *c* = 0.05 M). The vial was sealed with a septum-cap. Then, the mixture was stirred for 10 min at 25 °C, and placed 5 cm away from two blue LEDs (Kessil A160WE Tuna Blue (460 nm), LED lighting, 40 W). The mixture was irradiated for 12 h while maintaining the temperature at approximately 30 °C through cooling with a fan. After irradiation, EtOAc (10 mL) was added to the reaction mixture, and the resultant solution was washed with brine (2 × 5 mL). The organic phase was dried over Na_2SO_4, filtered, and the solvent was removed under reduced pressure. The residue was purified by flash column chromatography on silica gel eluting with EtOAc/pentane (0:100–1:24 (v/v)) to afford the title compound **11** as a colorless solid (46.4 mg, 0.137 mmol, 55%).

R_f = 0.22 (EtOAc/pentane, 1:9 (v/v)).

NMR Spectroscopy:

^{1}H NMR (500 MHz, $CDCl_3$, 298 K, δ): 7.83 (dd, *J* = 5.7, 3.0 Hz, 2H), 7.70 (dd, *J* = 5.6, 3.1 Hz, 2H), 3.66 (t, *J* = 7.3 Hz, 2H), 1.79 (s, 6H), 1.67 (p, *J* = 7.7 Hz, 2H), 1.54 (t, *J* = 7.9 Hz, 2H), 1.31 (p, *J* = 7.7 Hz, 2H).

^{13}C NMR (126 MHz, $CDCl_3$, 298 K, δ): 168.5, 134.0, 132.2, 123.3, 122.9 (q, *J* = 275.9 Hz), 48.5 (q, *J* = 2.4 Hz), 39.6, 37.8, 37.0 (q, *J* = 38.0 Hz), 30.6, 28.6, 23.6.

^{19}F NMR (471 MHz, $CDCl_3$, 298 K, δ): –73.39 (s).

HRMS-EI (m/z) calc'd for $C_{18}H_{18}NO_2F_3^+$ [M]$^+$, 337.1281; found, 337.1284; deviation: +0.8 ppm.

Bicyclo[1.1.1]pentylalkane 12

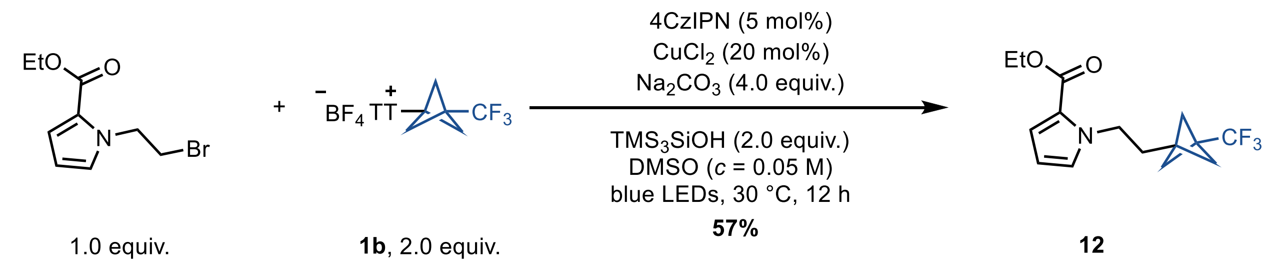

Under nitrogen atmosphere, to a 8 mL borosilicate vial equipped with a magnetic stir bar were added ethyl 1-(2-bromoethyl)-1H-pyrrole-2-carboxylate (61.5 mg, 0.250 mmol, 1.00 equiv.), CF_3BCP-TT$^+$ BF_4^- **1b** (220 mg, 0.500 mmol, 2.00 equiv.), 4CzIPN (10.0 mg, 12.5 µmoL, 5.00 mol%), $CuCl_2$ (6.7 mg, 50 µmoL, 20 mol%), Na_2CO_3 (106 mg, 1.00 mmol, 4.00 equiv.), TMS_3SiOH (132 mg, 0.500 mmol, 2.00 equiv.), and anhydrous DMSO (5.0 mL, *c* = 0.05 M). The vial was sealed with a septum-cap. Then, the mixture was stirred for 10 min at 25 °C, and placed 5 cm away from two blue LEDs (Kessil A160WE Tuna Blue (460 nm), LED lighting, 40 W). The mixture was irradiated for 12 h while maintaining the temperature at approximately 30 °C through cooling with a fan. After irradiation, EtOAc (10 mL) was added to the reaction mixture, and the resultant solution was washed with brine (2 × 5 mL). The organic phase was dried over Na_2SO_4, filtered, and the solvent was removed under reduced pressure. The residue was purified by flash column chromatography on silica gel eluting with EtOAc/pentane (0:100–1:99 (v/v)) to afford the title compound **12** as a colorless liquid (42.8 mg, 0.142 mmol, 57%).

R_f = 0.57 (EtOAc/pentane, 1:9 (v/v)).

NMR Spectroscopy:

^{1}H NMR (300 MHz, $CDCl_3$, 298 K, δ): 6.96 (dd, *J* = 4.0, 1.8 Hz, 1H), 6.82 (t, *J* = 2.2 Hz, 1H), 6.12 (dd, *J* = 4.0, 2.5 Hz, 1H), 4.34 – 4.22 (m, 4H), 2.10 – 1.96 (m, 2H), 1.85 (s, 6H), 1.35 (t, *J* = 7.1 Hz, 3H).

^{13}C NMR (151 MHz, $CDCl_3$, 298 K, δ): 161.2, 128.3, 122.7 (q, *J* = 275.9 Hz), 122.0, 118.4, 108.2, 59.9, 48.8 (q, *J* = 2.1 Hz), 46.5, 37.7 (q, *J* = 1.7 Hz), 37.4 (q, *J* = 38.1 Hz), 32.7, 14.5.

^{19}F NMR (282 MHz, $CDCl_3$, 298 K, δ): –73.45 (s).

HRMS-EI (m/z) calc'd for $C_{15}H_{18}NO_2F_3^+$ [M]$^+$, 301.1286; found, 301.1284; deviation: –0.9 ppm.

Bicyclo[1.1.1]pentylalkane 13

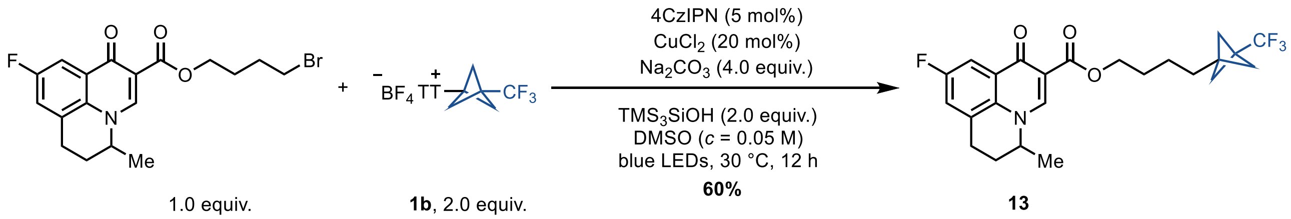

Under nitrogen atmosphere, to a 8 mL borosilicate vial equipped with a magnetic stir bar were added 4-bromobutyl 9-fluoro-5-methyl-1-oxo-6,7-dihydro-1H,5H-pyrido[3,2,1-ij]quinoline-2-carboxylate (99.0 mg, 0.250 mmol, 1.00 equiv.), CF_3BCP-TT$^+$ BF_4^- **1b** (220 mg, 0.500 mmol, 2.00 equiv.), 4CzIPN (10.0 mg, 12.5 µmoL, 5.00 mol%), $CuCl_2$ (6.7 mg, 50 µmoL, 20 mol%), Na_2CO_3 (106 mg, 1.00 mmol, 4.00 equiv.), TMS_3SiOH (132 mg, 0.500 mmol, 2.00 equiv.), and anhydrous DMSO (5.0 mL, *c* = 0.05 M). The vial was sealed with a septum-cap. Then, the mixture was stirred for 10 min at 25 °C, and placed 5 cm away from two blue LEDs (Kessil A160WE Tuna Blue (460 nm), LED lighting, 40 W). The mixture was irradiated for 12 h while maintaining the temperature at approximately 30 °C through cooling with a fan. After irradiation, EtOAc (10 mL) was added to the reaction mixture, and the resultant solution was washed with brine (2 × 5 mL). The organic phase was dried over Na_2SO_4, filtered, and the solvent was removed under reduced pressure. The residue was purified by flash column chromatography on silica gel eluting with EtOAc/pentane (1:9–2:3 (v/v)) to afford the title compound **13** as a pale yellowish solid (67.7 mg, 0.150 mmol, 60%).

R_f = 0.25 (EtOAc/pentane, 3:2 (v/v)).

NMR Spectroscopy:

^{1}H NMR (500 MHz, $CDCl_3$, 298 K, δ): 8.43 (s, 1H), 7.99 (dd, *J* = 9.0, 3.0 Hz, 1H), 7.20 (dd, *J* = 8.1, 3.0 Hz, 1H), 4.44 (tq, *J* = 7.2, 3.4 Hz, 1H), 4.31 (td, *J* = 6.9, 1.4 Hz, 2H), 3.15 (ddd, *J* = 17.9, 12.8, 5.4 Hz, 1H), 2.99 (ddd, *J* = 17.2, 5.0, 2.8 Hz, 1H), 2.24 (tt, *J* = 13.2, 4.7 Hz, 1H), 2.13 (ddt, *J* = 10.9, 5.5, 2.8 Hz, 1H), 1.81 (s, 6H), 1.78 (t, *J* = 7.3 Hz, 2H), 1.56 (dd, *J* = 9.7, 6.2 Hz, 2H), 1.48 (d, *J* = 6.8 Hz, 3H), 1.45 – 1.37 (m, 2H).

^{13}C NMR (151 MHz, $CDCl_3$, 298 K, δ): 173.5 (d, *J* = 2.6 Hz), 166.2, 160.4, 158.7, 147.0, 132.1 (d, *J* = 1.5 Hz), 131.2 (d, *J* = 7.2 Hz), 129.0 (d, *J* = 7.6 Hz), 126.0 – 119.9 (m), 120.1 (d, *J* = 24.7 Hz), 110.8 (d, *J* = 23.1 Hz), 110.1, 64.8, 57.2, 48.6 (q, *J* = 1.9 Hz), 39.8 (q, *J* = 1.6 Hz), 36.9 (q, *J* = 37.9 Hz), 30.7, 28.8, 26.4, 22.9, 22.1 (d, *J* = 1.4 Hz), 20.4.

^{19}F NMR (471 MHz, $CDCl_3$, 298 K, δ): –73.36 (s, 3F), –115.73 (t, *J* = 8.6 Hz, 1F).

HRMS-ESI (m/z) calc'd for $C_{24}H_{26}F_4NO_3^+$ [M+H]$^+$, 452.1846; found, 452.1843; deviation: –0.8 ppm.

Bicyclo[1.1.1]pentylalkane 14

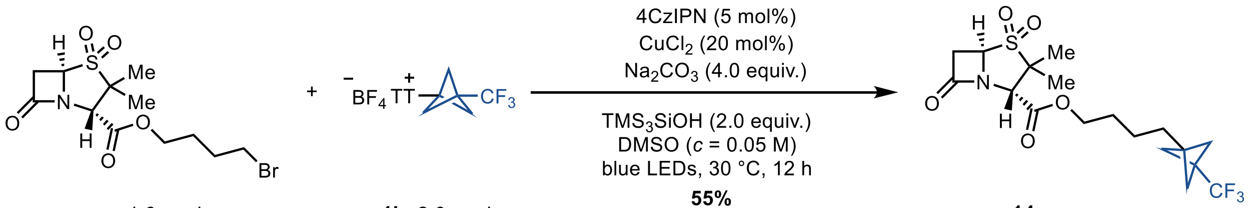

1.0 equiv. **1b**, 2.0 equiv. **14**

Under nitrogen atmosphere, to a 8 mL borosilicate vial equipped with a magnetic stir bar were added 4-bromobutyl (2S,5R)-3,3-dimethyl-7-oxo-4-thia-1-azabicyclo[3.2.0]heptane-2-carboxylate 4,4-dioxide (92.0 mg, 0.250 mmol, 1.00 equiv.), CF_3BCP-TT$^+$ BF_4^- **1b** (220 mg, 0.500 mmol, 2.00 equiv.), 4CzIPN (10.0 mg, 12.5 µmoL, 5.00 mol%), $CuCl_2$ (6.7 mg, 50 µmoL, 20 mol%), Na_2CO_3 (106 mg, 1.00 mmol, 4.00 equiv.),

TMS_3SiOH (132 mg, 0.500 mmol, 2.00 equiv.), and anhydrous DMSO (5.0 mL, *c* = 0.05 M). The vial was sealed with a septum-cap. Then, the mixture was stirred for 10 min at 25 °C, and placed 5 cm away from two blue LEDs (Kessil A160WE Tuna Blue (460 nm), LED lighting, 40 W). The mixture was irradiated for 12 h while maintaining the temperature at approximately 30 °C through cooling with a fan. After irradiation, EtOAc (10 mL) was added to the reaction mixture, and the resultant solution was washed with brine (2 × 5 mL). The organic phase was dried over Na_2SO_4, filtered, and the solvent was removed under reduced pressure. The residue was purified by flash column chromatography on silica gel eluting with EtOAc/pentane (1:19–1:4 (v/v)) to afford the title compound **14** as a colorless liquid (58.0 mg, 0.137 mmol, 55%).

R_f = 0.33 (EtOAc/pentane, 2:3 (v/v)).

NMR Spectroscopy:

^{1}H NMR (500 MHz, $CDCl_3$, 298 K, δ): 4.61 (dd, *J* = 4.3, 2.2 Hz, 1H), 4.37 (s, 1H), 4.19 (td, *J* = 6.7, 2.3 Hz, 2H), 3.52 – 3.39 (m, 2H), 1.81 (s, 6H), 1.73 – 1.64 (m, 2H), 1.60 (s, 3H), 1.56 – 1.50 (m, 2H), 1.40 (s, 3H), 1.37 – 1.29 (m, 2H).

^{13}C NMR (126 MHz, $CDCl_3$, 298 K, δ): 170.8, 167.1, 122.8 (q, *J* = 275.8 Hz), 66.3, 63.3, 62.7, 61.2, 48.5 (d, *J* = 2.4 Hz), 39.5, 38.4, 37.0 (q, *J* = 38.0 Hz), 30.6, 28.5, 27.0, 22.7, 20.4, 18.7.

^{19}F NMR (471 MHz, $CDCl_3$, 298 K, δ): –73.37 (s).

HRMS-ESI (m/z) calc'd for $C_{18}H_{24}O_5NNaF_3S^+$ $[M+Na]^+$, 446.1219; found, 446.1219; deviation: –0.0 ppm.

Bicyclo[1.1.1]pentylalkane 15

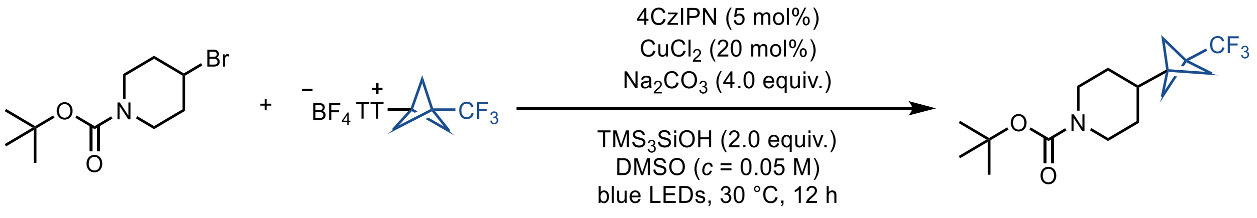

1.0 equiv. 2.0 equiv. **15**

Under nitrogen atmosphere, to a 8 mL borosilicate vial equipped with a magnetic stir bar were added tert-butyl 4-bromopiperidine-1-carboxylate (66.0 mg, 0.250 mmol, 1.00 equiv.), $CF_3BCP\text{-}TT^+$ BF_4^- **1b** (220 mg, 0.500 mmol, 2.00 equiv.), 4CzIPN (10.0 mg, 12.5 µmoL, 5.00 mol%), $CuCl_2$ (6.7 mg, 50 µmoL, 20 mol%), Na_2CO_3 (106 mg, 1.00 mmol, 4.00 equiv.), TMS_3SiOH (132 mg, 0.500 mmol, 2.00 equiv.), and anhydrous DMSO (5.0 mL, *c* = 0.05 M). The vial was sealed with a septum-cap. Then, the mixture was stirred for 10 min at 25 °C, and placed 5 cm away from two blue LEDs (Kessil A160WE Tuna Blue (460 nm), LED lighting, 40 W). The mixture was irradiated for 12 h while maintaining the temperature at approximately 30 °C through cooling with a fan. After irradiation, EtOAc (10 mL) was added to the reaction mixture, and the resultant solution was washed with brine (2 × 5 mL). The organic phase was dried over Na_2SO_4, filtered, and the solvent was removed under reduced pressure. The residue was purified by flash column chromatography on silica gel eluting with EtOAc/pentane (0:100–1:49 (v/v)) to afford the title compound **15** as a colorless solid

(34.4 mg, 0.107 mmol, 43%).

R*f* = 0.32 (EtOAc/pentane, 1:9 (v/v)).

NMR Spectroscopy:

^{1}H NMR (500 MHz, $CDCl_3$, 298 K, δ): 4.13 (s, 2H), 2.63 (s, 2H), 1.77 (s, 6H), 1.59 – 1.49 (m, 3H), 1.44 (s, 9H), 1.12 – 1.00 (m, 2H).

^{13}C NMR (126 MHz, $CDCl_3$, 298 K, δ): 154.9, 123.0 (q, *J* = 275.8 Hz), 79.5, 46.5 (q, *J* = 2.6 Hz), 42.7, 36.5 (q, *J* = 38.0 Hz), 36.0, 28.5, 28.5, 28.2.

^{19}F NMR (471 MHz, $CDCl_3$, 298 K, δ): –73.41 (s).

HRMS-EI (m/z) calc'd for $C_{16}H_{24}NO_2F_3^+$ $[M]^+$, 319.1756; found, 319.1753; deviation: –1.0 ppm.

Bicyclo[1.1.1]pentylalkane 16

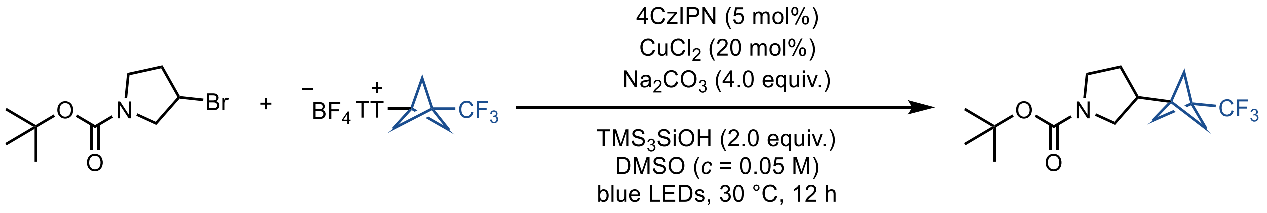

1.0 equiv. 2.0 equiv. **16**

Under nitrogen atmosphere, to a 8 mL borosilicate vial equipped with a magnetic stir bar were added tert-butyl 3-bromopyrrolidine-1-carboxylate (62.5 mg, 0.250 mmol, 1.00 equiv.), CF_3BCP-TT^+ BF_4^- **1b** (220 mg, 0.500 mmol, 2.00 equiv.), 4CzIPN (10.0 mg, 12.5 µmoL, 5.00 mol%), $CuCl_2$ (6.7 mg, 50 µmoL, 20 mol%), Na_2CO_3 (106 mg, 1.00 mmol, 4.00 equiv.), TMS_3SiOH (132 mg, 0.500 mmol, 2.00 equiv.), and anhydrous DMSO (5.0 mL, *c* = 0.05 M). The vial was sealed with a septum-cap. Then, the mixture was stirred for 10 min at 25 °C, and placed 5 cm away from two blue LEDs (Kessil A160WE Tuna Blue (460 nm), LED lighting, 40 W). The mixture was irradiated for 12 h while maintaining the temperature at approximately 30 °C through cooling with a fan. After irradiation, EtOAc (10 mL) was added to the reaction mixture, and the resultant solution was washed with brine (2 × 5 mL). The organic phase was dried over Na_2SO_4, filtered, and the solvent was removed under reduced pressure. The residue was purified by flash column chromatography on silica gel eluting with EtOAc/pentane (0:100–3:97 (v/v)) to afford the title compound **16** as a colorless solid (34.2 mg, 0.112 mmol, 45%).

R*f* = 0.25 (EtOAc/pentane, 1:9 (v/v)).

NMR Spectroscopy:

^{1}H NMR (500 MHz, $CDCl_3$, 298 K, δ): 3.78 – 2.87 (m, 4H), 2.41 – 1.87 (m, 2H), 1.83 (s, 6H), 1.66 – 1.54 (m, 1H), 1.44 (s, 9H).

^{13}C NMR (126 MHz, $CDCl_3$, 298 K, δ): 154.6, 122.8 (q, *J* = 275.7 Hz), 79.3, 48.1, 47.4 (d, *J* = 2.4 Hz), 45.6, 40.5, 36.9 (q, *J* = 38.2 Hz), 28.6, 28.5, 27.9.

^{19}F NMR (471 MHz, $CDCl_3$, 298 K, δ): –73.44 (s).

HRMS-EI (m/z) calc'd for $C_{15}H_{22}NO_2F_3^+$ [M]$^+$, 305.1600; found, 305.1597; deviation: –1.0 ppm.

Bicyclo[1.1.1]pentylalkane 17

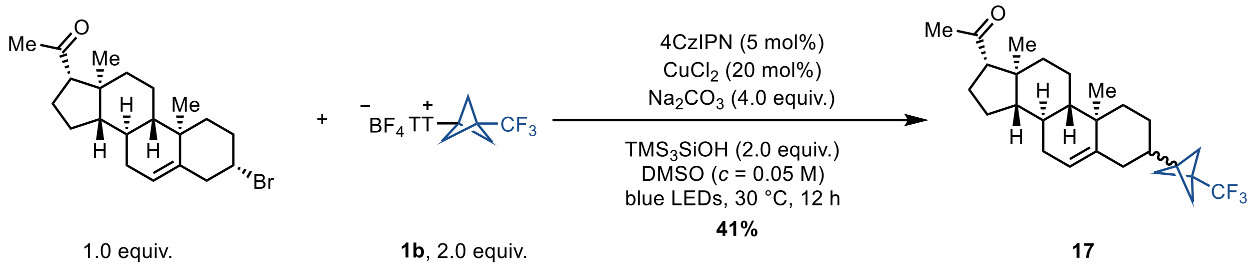

Under nitrogen atmosphere, to a 8 mL borosilicate vial equipped with a magnetic stir bar were added 1-((8S,9S,10R,13S,14S,17S)-3-bromo-10,13-dimethyl-2,3,4,7,8,9,10,11,12,13,14,15,16,17-tetradecahydro-1H-cyclopenta[a]phenanthren-17-yl)ethan-1-one (94.8 mg, 0.250 mmol, 1.00 equiv., d.r. > 99:1), CF_3BCP-TT$^+$ BF_4^- **1b** (220 mg, 0.500 mmol, 2.00 equiv.), 4CzIPN (10.0 mg, 12.5 µmoL, 5.00 mol%), $CuCl_2$ (6.7 mg, 50 µmoL, 20 mol%), Na_2CO_3 (106 mg, 1.00 mmol, 4.00 equiv.), TMS_3SiOH (132 mg, 0.500 mmol, 2.00 equiv.), and anhydrous DMSO (5.0 mL, c = 0.05 M). The vial was sealed with a septum-cap. Then, the mixture was stirred for 10 min at 25 °C, and placed 5 cm away from two blue LEDs (Kessil A160WE Tuna Blue (460 nm), LED lighting, 40 W). The mixture was irradiated for 12 h while maintaining the temperature at approximately 30 °C through cooling with a fan. After irradiation, EtOAc (10 mL) was added to the reaction mixture, and the resultant solution was washed with brine (2 × 5 mL). The organic phase was dried over Na_2SO_4, filtered, and the solvent was removed under reduced pressure. The residue was purified by flash column chromatography on silica gel eluting with EtOAc/pentane (0:100–1:99 (v/v)) to afford the title compound **17** as a colorless liquid (44.3 mg, 0.102 mmol, 41%, d.r. = 82:18). The d.r. was determined by ^{1}H NMR analysis.

R_f = 0.57 (EtOAc/pentane, 1:9 (v/v)).

NMR Spectroscopy:

^{1}H NMR (500 MHz, $CDCl_3$, 298 K, δ): 5.30 (t, J = 2.6 Hz, 1H), 2.53 (t, J = 9.0 Hz, 1H), 2.20 – 2.15 (m, 1H), 2.12 (s, 3H), 2.07 – 1.98 (m, 3H), 1.91 – 1.84 (m, 3H), 1.77 (s, 6H), 1.72 – 1.60 (m, 3H), 1.53 – 1.39 (m, 5H), 1.22 – 1.11 (m, 3H), 1.09 – 1.00 (m, 2H), 0.95 (s, 3H), 0.63 (s, 3H).

^{13}C NMR (126 MHz, $CDCl_3$, 298 K, δ): 209.7, 142.3, 124.9 – 118.4 (m), 119.8, 63.8, 57.1, 50.4, 46.5, 44.1, 43.1, 39.1, 39.0, 39.0, 37.1, 36.5, 35.3, 31.9, 31.9, 31.7, 25.0, 24.6, 22.9, 21.0, 19.5, 13.3.

^{19}F NMR (282 MHz, $CDCl_3$, 298 K, δ): –73.38 (s).

HRMS-EI (m/z) calc'd for $C_{27}H_{37}OF_3^+$ [M]$^+$, 434.2792; found, 434.2791; deviation: –0.4 ppm.

Bicyclo[1.1.1]pentylalkane 18

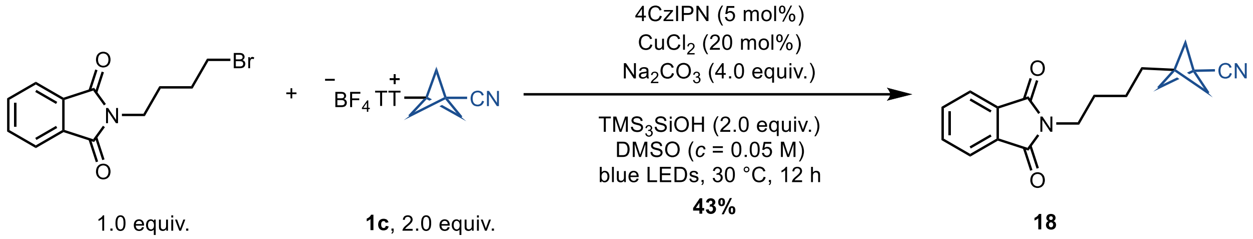

Under nitrogen atmosphere, to a 8 mL borosilicate vial equipped with a magnetic stir bar were added 2-(4-bromobutyl)isoindoline-1,3-dione (70.5 mg, 0.250 mmol, 1.00 equiv.), NC-BCP-TT$^+$ BF_4^- **1c** (198 mg, 0.500 mmol, 2.00 equiv.), 4CzIPN (10.0 mg, 12.5 µmoL, 5.00 mol%), $CuCl_2$ (6.7 mg, 50 µmoL, 20 mol%), Na_2CO_3 (106 mg, 1.00 mmol, 4.00 equiv.), TMS_3SiOH (132 mg, 0.500 mmol, 2.00 equiv.), and anhydrous DMSO (5.0 mL, c = 0.05 M). The vial was sealed with a septum-cap. Then, the mixture was stirred for 10 min at 25 °C, and placed 5 cm away from two blue LEDs (Kessil A160WE Tuna Blue (460 nm), LED lighting, 40 W). The mixture was irradiated for 12 h while maintaining the temperature at approximately 30 °C through cooling with a fan. After irradiation, EtOAc (10 mL) was added to the reaction mixture, and the resultant solution was washed with brine (2 × 5 mL). The organic phase was dried over Na_2SO_4, filtered, and the solvent was removed under reduced pressure. The residue was purified by flash column chromatography on silica gel eluting with EtOAc/pentane (1:99–2:23 (v/v)) to afford the title compound **18** as a colorless liquid (31.5 mg, 0.107 mmol, 43%).

$\mathbf{R}_f$ = 0.13 (EtOAc/pentane, 1:9 (v/v)).

NMR Spectroscopy:

^{1}H NMR (500 MHz, $CDCl_3$, 298 K, δ): 7.83 (dd, J = 5.5, 3.0 Hz, 2H), 7.71 (dd, J = 5.5, 3.0 Hz, 2H), 3.65 (t, J = 7.3 Hz, 2H), 2.07 (s, 6H), 1.70 – 1.60 (m, 2H), 1.53 – 1.45 (m, 2H), 1.32 – 1.22 (m, 2H).

^{13}C NMR (126 MHz, $CDCl_3$, 298 K, δ): 168.5, 134.0, 132.1, 123.3, 118.0, 54.2, 44.5, 37.7, 30.4, 28.5, 23.6, 23.5.

HRMS-EI (m/z) calc'd for $C_{18}H_{18}N_2O_2^+$ [M]$^+$, 294.1363; found, 294.1362; deviation: –0.2 ppm.

Bicyclo[1.1.1]pentylmethylarene 19

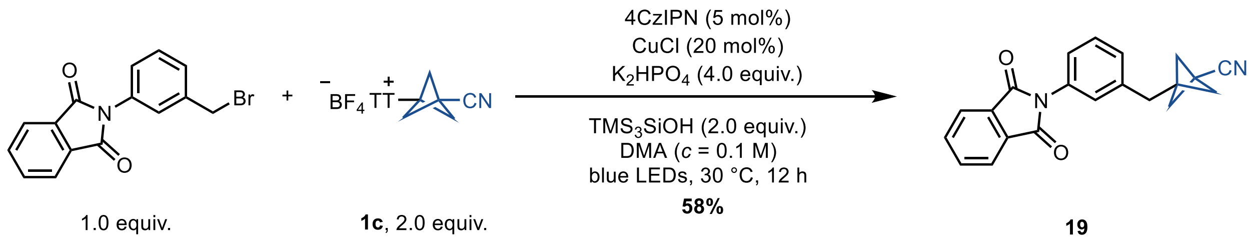

Under nitrogen atmosphere, to a 4 mL borosilicate vial equipped with a magnetic stir bar were added 2-(3-(bromomethyl)phenyl)isoindoline-1,3-dione (79.0 mg, 0.250 mmol, 1.00 equiv.), NC-BCP-TT$^+$ BF_4^- **1c** (198 mg, 0.500 mmol, 2.00 equiv.), 4CzIPN (10.0 mg, 12.5 µmoL, 5.00 mol%), CuCl (5.0 mg, 50 µmoL, 20

mol%), K_2HPO_4 (174 mg, 1.00 mmol, 4.00 equiv.), TMS_3SiOH (132 mg, 0.500 mmol, 2.00 equiv.), and anhydrous DMA (2.5 mL, *c* = 0.10 M). The vial was sealed with a septum-cap. Then, the mixture was stirred for 10 min at 25 °C, and placed 5 cm away from two blue LEDs (Kessil A160WE Tuna Blue (460 nm), LED lighting, 40 W). The mixture was irradiated for 12 h while maintaining the temperature at approximately 30 °C through cooling with a fan. After irradiation, EtOAc (6 mL) was added to the reaction mixture, and the resultant solution was washed with brine (2 × 3 mL). The organic phase was dried over Na_2SO_4, filtered, and the solvent was removed under reduced pressure. The residue was purified by flash column chromatography on silica gel eluting with EtOAc/pentane (1:19–3:17 (v/v)) to afford the title compound **19** as a colorless liquid (47.6 mg, 0.145 mmol, 58%).

R_f = 0.13 (EtOAc/pentane, 3:17 (v/v)).

NMR Spectroscopy:

^{1}H NMR (500 MHz, $CDCl_3$, 298 K, δ): 7.94 (dd, *J* = 5.4, 3.0 Hz, 2H), 7.79 (dd, *J* = 5.5, 3.0 Hz, 2H), 7.43 (t, *J* = 7.8 Hz, 1H), 7.32 (ddd, *J* = 8.0, 2.1, 1.1 Hz, 1H), 7.14 (t, *J* = 1.9 Hz, 1H), 7.08 (dt, *J* = 7.7, 1.4 Hz, 1H), 2.83 (s, 2H), 2.10 (s, 6H).

^{13}C NMR (126 MHz, $CDCl_3$, 298 K, δ): 167.2, 138.7, 134.5, 131.9, 131.7, 129.3, 128.4, 126.8, 124.5, 123.8, 117.8, 54.0, 44.4, 38.0, 24.3.

HRMS-ESI (m/z) calc'd for $C_{21}H_{16}N_2NaO_2^+$ $[M+Na]^+$, 351.1105; found, 351.1104; deviation: –0.3 ppm.

Bicyclo[1.1.1]pentylmethylarene 20

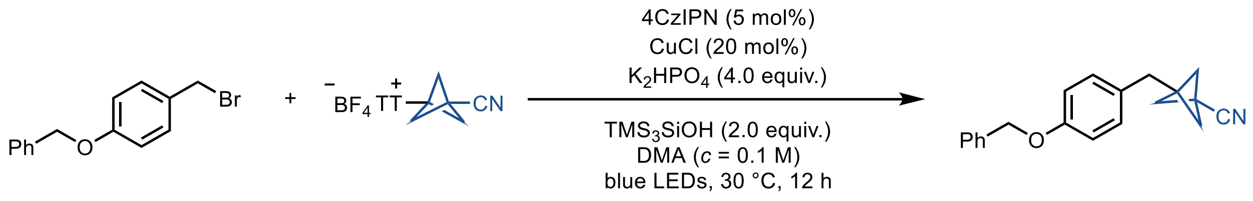

1.0 equiv. **1c**, 2.0 equiv. **20**

Under nitrogen atmosphere, to a 4 mL borosilicate vial equipped with a magnetic stir bar were added 1-(benzyloxy)-4-(bromomethyl)benzene (69.3 mg, 0.250 mmol, 1.00 equiv.), NC-BCP-TT^+ BF_4^- **1c** (198 mg, 0.500 mmol, 2.00 equiv.), 4CzIPN (10.0 mg, 12.5 µmoL, 5.00 mol%), CuCl (5.0 mg, 50 µmoL, 20 mol%), K_2HPO_4 (174 mg, 1.00 mmol, 4.00 equiv.), TMS_3SiOH (132 mg, 0.500 mmol, 2.00 equiv.), and anhydrous DMA (2.5 mL, *c* = 0.10 M). The vial was sealed with a septum-cap. Then, the mixture was stirred for 10 min at 25 °C, and placed 5 cm away from two blue LEDs (Kessil A160WE Tuna Blue (460 nm), LED lighting, 40 W). The mixture was irradiated for 12 h while maintaining the temperature at approximately 30 °C through cooling with a fan. After irradiation, EtOAc (6 mL) was added to the reaction mixture, and the resultant solution was washed with brine (2 × 3 mL). The organic phase was dried over Na_2SO_4, filtered, and the solvent was removed under reduced pressure. The residue was purified by flash column chromatography on silica gel eluting with EtOAc/pentane (0:100–1:99 (v/v)) to afford the title compound **20** as a colorless solid (44.1 mg, 0.152 mmol, 61%).

R$_f$ = 0.27 (EtOAc/pentane, 1:9 (v/v)).

NMR Spectroscopy:

^{1}H NMR (500 MHz, $CDCl_3$, 298 K, δ): 7.48 – 7.42 (m, 2H), 7.42 – 7.37 (m, 2H), 7.36 – 7.31 (m, 1H), 7.00 – 6.94 (m, 2H), 6.94 – 6.87 (m, 2H), 5.05 (s, 2H), 2.70 (s, 2H), 2.05 (s, 6H).

^{13}C NMR (126 MHz, $CDCl_3$, 298 K, δ): 157.6, 137.1, 130.1, 129.8, 128.7, 128.1, 127.6, 118.0, 115.0, 70.1, 54.0, 44.8, 37.4, 24.2.

HRMS-EI (m/z) calc'd for $C_{20}H_{19}NO^+$ [M]$^+$, 289.1461; found, 289.1461; deviation: –0.2 ppm.

Bicyclo[1.1.1]pentylalkane 21

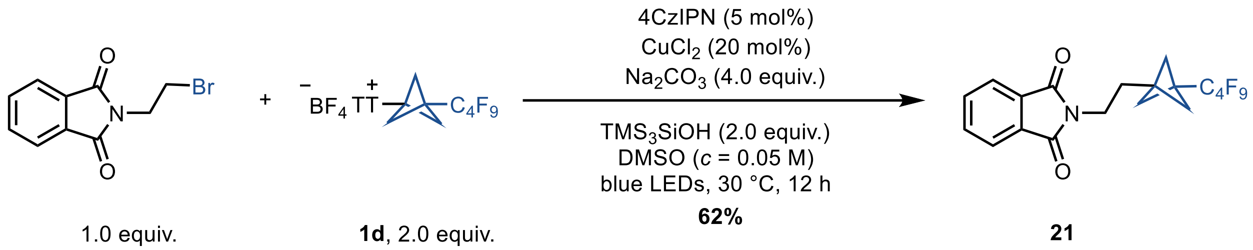

1.0 equiv. **1d**, 2.0 equiv. **21**

Under nitrogen atmosphere, to a 8 mL borosilicate vial equipped with a magnetic stir bar were added 2-(2-bromoethyl)isoindoline-1,3-dione (63.5 mg, 0.250 mmol, 1.00 equiv.), C_4F_9-BCP-TT$^+$ BF_4^- **1d** (294 mg, 0.500 mmol, 2.00 equiv.), 4CzIPN (10.0 mg, 12.5 µmoL, 5.00 mol%), $CuCl_2$ (6.7 mg, 50 µmoL, 20 mol%), Na_2CO_3 (106 mg, 1.00 mmol, 4.00 equiv.), TMS_3SiOH (132 mg, 0.500 mmol, 2.00 equiv.), and anhydrous DMSO (5.0 mL, *c* = 0.05 M). The vial was sealed with a septum-cap. Then, the mixture was stirred for 10 min at 25 °C, and placed 5 cm away from two blue LEDs (Kessil A160WE Tuna Blue (460 nm), LED lighting, 40 W). The mixture was irradiated for 12 h while maintaining the temperature at approximately 30 °C through cooling with a fan. After irradiation, EtOAc (10 mL) was added to the reaction mixture, and the resultant solution was washed with brine (2 × 5 mL). The organic phase was dried over Na_2SO_4, filtered, and the solvent was removed under reduced pressure. The residue was purified by flash column chromatography on silica gel eluting with EtOAc/pentane (0:100–1:24 (v/v)) to afford the title compound **21** as a colorless solid (71.2 mg, 0.155 mmol, 62%).

R$_f$ = 0.28 (EtOAc/pentane, 3:17 (v/v)).

NMR Spectroscopy:

^{1}H NMR (500 MHz, $CDCl_3$, 298 K, δ): 7.84 (dd, *J* = 5.4, 3.1 Hz, 2H), 7.72 (dd, *J* = 5.5, 3.0 Hz, 2H), 3.71 – 3.64 (m, 2H), 1.99 – 1.92 (m, 8H).

^{13}C {^{1}H, ^{19}F} NMR (126 MHz, $CDCl_3$, 298 K, δ): 168.3, 134.1, 132.1, 123.4, 117.5, 113.4, 111.4, 108.7, 49.7, 38.4, 37.4, 35.4, 29.6.

^{19}F NMR (470 MHz, $CDCl_3$, 298 K, δ): –81.16 (td, *J* = 9.5, 4.7 Hz, 3F), –116.60 (ddd, *J* = 16.2, 10.0, 5.7 Hz, 2F), –122.38 (q, *J* = 8.7 Hz, 2F), –126.15 (ddd, *J* = 16.1, 11.8, 4.3 Hz, 2F).

HRMS-ESI (m/z) calc'd for $C_{19}H_{14}F_9NNaO_2^+$ [M+Na]$^+$, 482.0780; found, 482.0773; deviation: –1.5 ppm.

Bicyclo[1.1.1]pentylmethypyridine 22

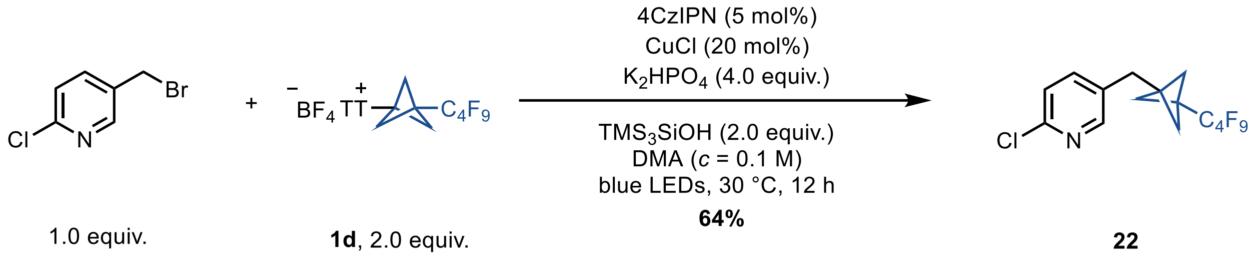

1.0 equiv. **1d**, 2.0 equiv. **22**

Under nitrogen atmosphere, to a 4 mL borosilicate vial equipped with a magnetic stir bar were added 5-(bromomethyl)-2-chloropyridine (51.6 mg, 0.250 mmol, 1.00 equiv.), C_4F_9-BCP-TT$^+$ BF_4^- **1d** (294 mg, 0.500 mmol, 2.00 equiv.), 4CzIPN (10.0 mg, 12.5 µmoL, 5.00 mol%), CuCl (5.0 mg, 50 µmoL, 20 mol%), K_2HPO_4 (174 mg, 1.00 mmol, 4.00 equiv.), TMS_3SiOH (132 mg, 0.500 mmol, 2.00 equiv.), and anhydrous DMA (2.5 mL, *c* = 0.10 M). The vial was sealed with a septum-cap. Then, the mixture was stirred for 10 min at 25 °C, and placed 5 cm away from two blue LEDs (Kessil A160WE Tuna Blue (460 nm), LED lighting, 40 W). The mixture was irradiated for 12 h while maintaining the temperature at approximately 30 °C through cooling with a fan. After irradiation, EtOAc (6 mL) was added to the reaction mixture, and the resultant solution was washed with brine (2 × 3 mL). The organic phase was dried over Na_2SO_4, filtered, and the solvent was removed under reduced pressure. The residue was purified by flash column chromatography on silica gel eluting with EtOAc/pentane (0:100–1:39 (v/v)) to afford the title compound **22** as a colorless liquid (66 mg, 0.16 mmol, 64%).

$\mathbf{R_f}$ = 0.3 (EtOAc/pentane, 1:9 (v/v)).

NMR Spectroscopy:

^{1}H NMR (500 MHz, $CDCl_3$, 298 K, δ): 8.13 (d, *J* = 2.5 Hz, 1H), 7.38 (dd, *J* = 8.1, 2.5 Hz, 1H), 7.27 (d, *J* = 8.4 Hz, 1H), 2.82 (s, 2H), 1.87 (s, 6H).

^{13}C {^{1}H, ^{19}F} NMR (126 MHz, $CDCl_3$, 298 K, δ): 149.9, 149.8, 139.2, 132.6, 124.2, 117.5, 113.5, 111.3, 108.7, 49.3, 40.1, 37.9, 34.7.

^{19}F NMR (471 MHz, $CDCl_3$, 298 K, δ): –81.16 (td, *J* = 9.4, 4.5 Hz, 3F), –116.66 (t, *J* = 13.0 Hz, 2F), –122.39 (q, *J* = 8.5 Hz, 2F), –126.14 (tt, *J* = 15.1, 10.1 Hz, 2F).

HRMS-EI (m/z) calc'd for $C_{15}H_{11}NF_9Cl^+$ [M]$^+$, 411.0437; found, 411.0430; deviation: –1.6 ppm.

Synthesis of BCP analog of tesmilifene 25

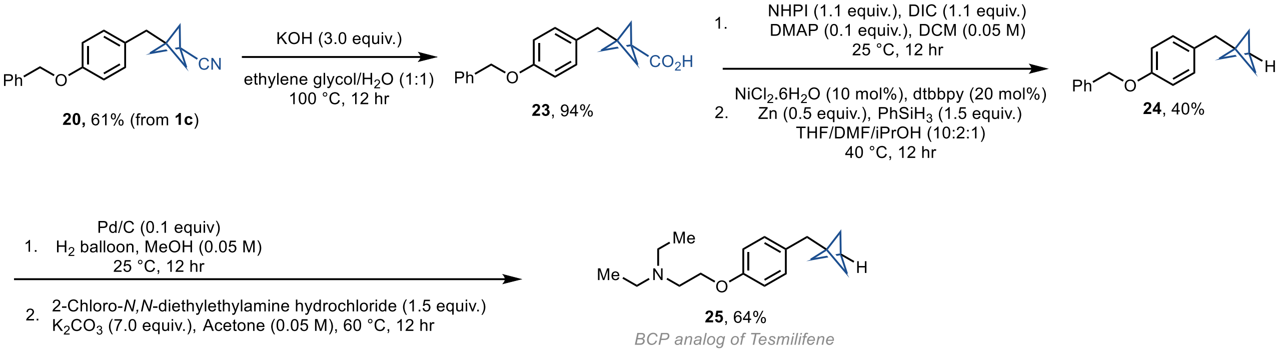

Under ambient atmosphere, to a 10 mL round-bottom flask equipped with a magnetic stir bar were added **20** (86.8 mg, 0.300 mmol, 1.00 equiv.), KOH (51.0 mg, 0.900 mmol, 3.00 equiv.), and 1:1 H_2O-ethylene glycol (3.0 mL, c = 0.10 M). Then, the mixture was stirred at 100 °C for 12 h. After cooling to 25 °C, H_2O (10 mL) was added to the mixture, and the mixture was then acidified to pH = 1–2 with 1M HCl solution. The aqueous phase was extracted with EtOAc (3 × 10 mL). The combined organic phase was washed with brine (1 × 5 mL), dried over Na_2SO_4, filtered, and the solvent was removed under reduced pressure to afford the compound **23** as a pale yellow solid (87.0mg, 0.282 mmol, 94%).

R_f = 0.12 (EtOAc/pentane, 1:4 (v/v)).

NMR Spectroscopy:

^{1}H NMR (500 MHz, $CDCl_3$, 298 K, δ): 7.44 (d, *J* = 7.1 Hz, 2H), 7.39 (t, J = 7.5 Hz, 2H), 7.36 – 7.30 (m, 1H), 6.99 (d, *J* = 8.6 Hz, 2H), 6.90 (d, *J* = 8.6 Hz, 2H), 5.04 (s, 2H), 2.72 (s, 2H), 1.88 (s, 6H).

^{13}C NMR (126 MHz, $CDCl_3$, 298 K, δ): 175.9, 157.4, 137.2, 131.1, 129.9, 128.7, 128.0, 127.6, 114.8, 70.2, 51.5, 40.5, 38.2, 37.7.

HRMS-EI (m/z) calculated for $C_{20}H_{20}O_3^+$ $[M]^+$, 308.1408; found, 308.1406; deviation: –0.4ppm.

Under ambient atmosphere, to a 10 mL round-bottom flask equipped with a magnetic stir bar were added **23** (65 mg, 0.21 mmol, 1.0 equiv.), *N*-hydroxyphthalimide (38 mg, 0.23 mmol, 1.1 equiv.), DMAP (2.5 mg, 0.02 mmol, 0.10 equiv.) and DCM (4.0 mL, c = 0.05 M). DIC (36 µL, 0.23 mmol, 1.1 equiv.) was then added dropwise via syringe, and the reaction mixture was stirred at 25 °C for 12 h. The mixture was filtered through the thin pads of silica and rinsed with additional DCM. The solvent was removed under reduced pressure to afford the NHPI redox-active ester of **23**, which was used for the next step without further purification.

Under nitrogen atmosphere, to a 4 mL borosilicate vial equipped with a magnetic stir bar were added NHPI redox-active ester of **23** (91 mg, 0.20 mmol, 1.0 equiv.), Zn metal (6.4 mg, 0.10 mmol, 0.50 equiv.), anhydrous THF (1.0 mL, *c* = 0.20 M) and ^{i}PrOH (0.1 mL, *c* = 2.0 M). A solution of $NiCl_2$•$6H_2O$/dtbbpy (1.0 M in DMF, 0.2 mL, 10 mol% $NiCl_2$•$6H_2O$, 20 mol% dtbbpy) and $PhSiH_3$ (18 µL, 0.3 mmol, 1.5 equiv.) were added in quick succession. The vial was sealed with a septum-cap and the mixture was stirred at 40 °C for 12hr. EtOAc (10 mL) was added to the reaction mixture, and the resultant solution was washed with brine (2 × 5 mL). The organic phase was dried over Na_2SO_4, filtered, and the solvent was removed under reduced

pressure. The residue was purified by flash column chromatography on silica gel eluting with EtOAc/pentane (1:19(v/v)) to afford the compound **24** as a colorless liquid (22 mg, 0.08mmol, 40%).

R*f* = 0.56 (EtOAc/pentane, 1:19 (v/v)).

NMR Spectroscopy:

> **^{1}H NMR** (500 MHz, $CDCl_3$, 298 K, δ): 7.39 (dd, *J* = 8.3, 6.6 Hz, 2H), 7.32 (t, *J* = 7.3 Hz, 2H), 7.26 (s, 1H), 7.01 (d, *J* = 8.4 Hz, 2H), 6.90 (d, *J* = 8.6 Hz, 2H), 5.04 (s, 2H), 2.66 (s, 1H), 1.60 (s, 6H).

> **^{13}C NMR** (126 MHz, $CDCl_3$, 298 K, δ): 157.1, 137.4, 132.3, 129.9, 128.6, 128.0, 127.6, 114.6, 70.1, 50.2, 45.8, 39.1, 28.1.

HRMS-EI (m/z) calc'd for $C_{19}H_{20}O^{+}$ $[M]^{+}$, 64.1511; found, 264.1508; deviation: –1.2 ppm.

To a 10 mL round-bottom flask equipped with a magnetic stir bar were added **24** (40 mg, 0.15 mmol, 1.0 equiv.), Pd/C (10 wt%) (16.0 mg, 0.015 mmol, 0.100 equiv.). The flask was sealed with a septum-cap and then, evacuated and backfilled with argon (three times). Anhydrous MeOH (1.5 mL, *c* = 0.5 M) was added to the flask using a syringe. Then, the flask evacuated and backfilled with H_2 using a balloon. The H_2 balloon was connected with the flask and the reaction mixture was stirred for 12 hr at 25 °C. The mixture was filtered through the thin pads of celite and rinsed with additional DCM. The solvent was removed under reduced pressure to afford pale yellowish oil, which was used for the next step without further purification.

To a 10 mL round-bottom flask equipped with a magnetic stir bar were added the crude product obtained in the previous step, K_2CO_3 (145 mg, 1.05 mmol, 7.00 equiv.), 2-Chloro-N,N-diethylethylamine hydrochloride (39 mg, 0.22 mmol, 1.5 equiv.) and anhydrous acetone (3.0 mL, *c* = 0.05 M). The flask was sealed with a septum-cap and the reaction mixture was stirred at 60 °C for 12 hr. EtOAc (10 mL) was added to the reaction mixture, and the resultant solution was washed with brine (2 × 5 mL). The organic phase was dried over Na_2SO_4, filtered, and the solvent was removed under reduced pressure. The residue was purified by flash column chromatography on silica gel eluting with MeOH/DCM (0:100 – 6 :94 (v/v)) to afford the compound **25** as a colorless liquid (26 mg, 0.09 mmol, 64%).

R*f* = 0.34 (MeOH/DCM, 1:9 (v/v)).

NMR Spectroscopy:

> **^{1}H NMR** (500 MHz, $CDCl_3$, 298 K, δ): 7.02 – 6.95 (m, 2H), 6.84 – 6.77 (m, 2H), 4.13 (t, *J* = 6.0 Hz, 2H), 3.01 (t, *J* = 6.0 Hz, 2H), 2.79 (q, *J* = 7.2 Hz, 4H), 2.64 (s, 2H), 2.44 (s, 1H), 1.59 (s, 6H), 1.16 (t, *J* = 7.2 Hz, 6H).

> **^{13}C NMR** (126 MHz, $CDCl_3$, 298 K, δ): 156.7, 132.4, 129.9, 114.2, 65.6, 51.6, 50.2, 47.7, 45.7, 39.1, 28.1, 11.1.

HRMS-EI (m/z) calc'd for $C_{18}H_{27}NO^{+}$ $[M]^{+}$, 273.2086; found, 273.2087; deviation: +0.1 ppm.

Unsuccessful substrates

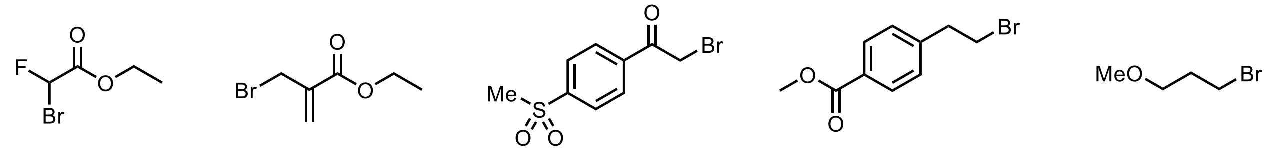

nd | nd | nd | 36% | 31%

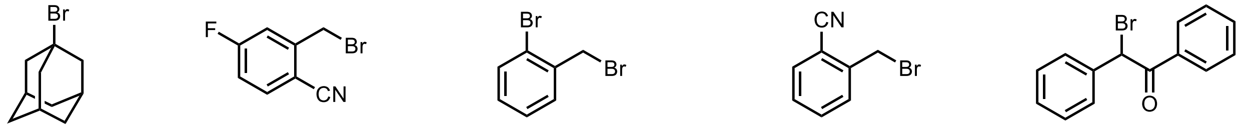

nd | 24% | nd | 36% | nd

Figure S1. Unsuccessful substrates for the cross-coupling reaction with **1b**. ^{19}F NMR yield using $PhCF_3$ as an internal standard, and *nd* means no product was detected.

SPECTROSCOPIC DATA

^{1}H NMR of TMS_3SiOH

$CDCl_3$, 298 K

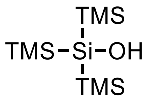

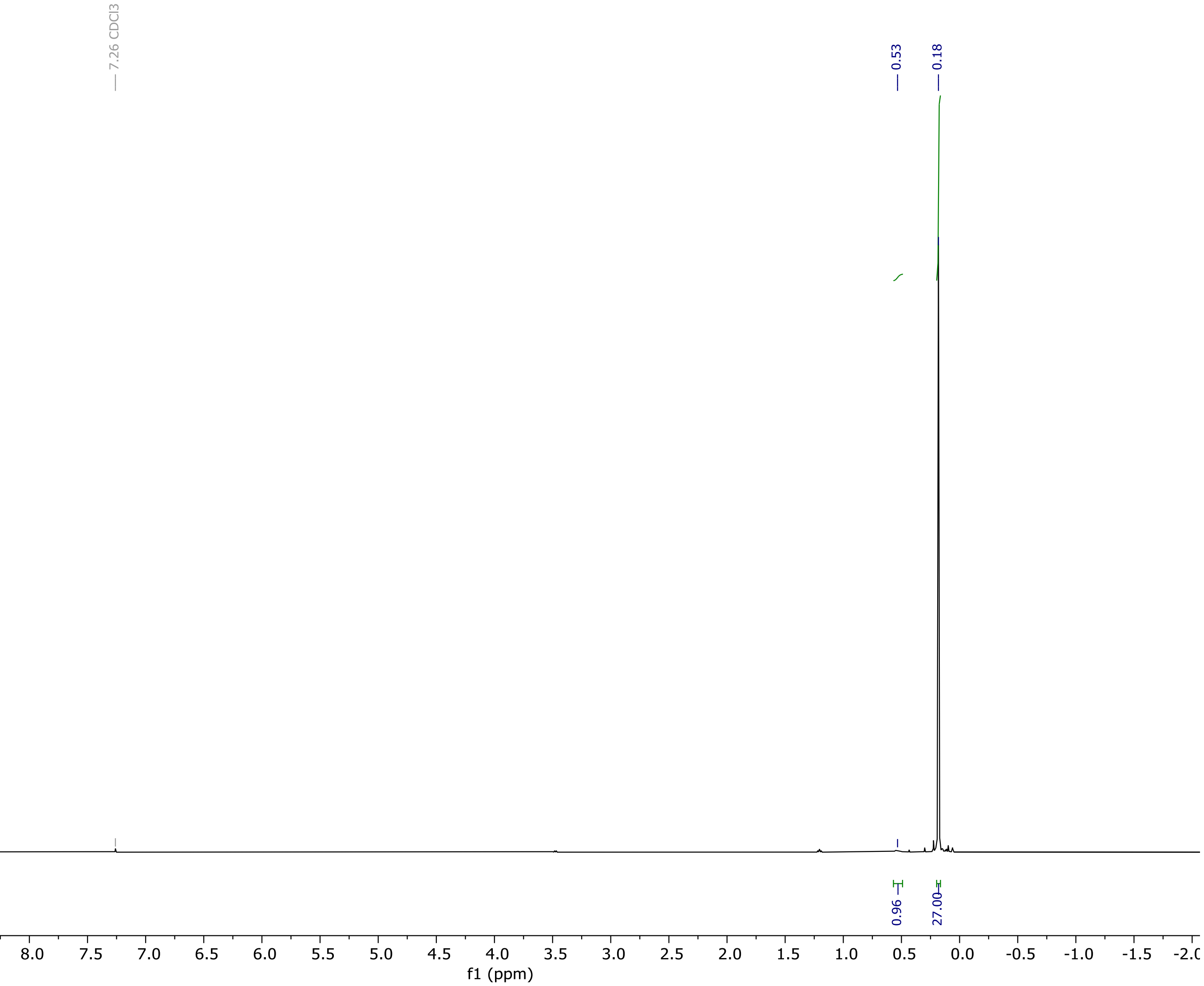

^{13}C NMR of TMS_3SiOH

$CDCl_3$, 298 K

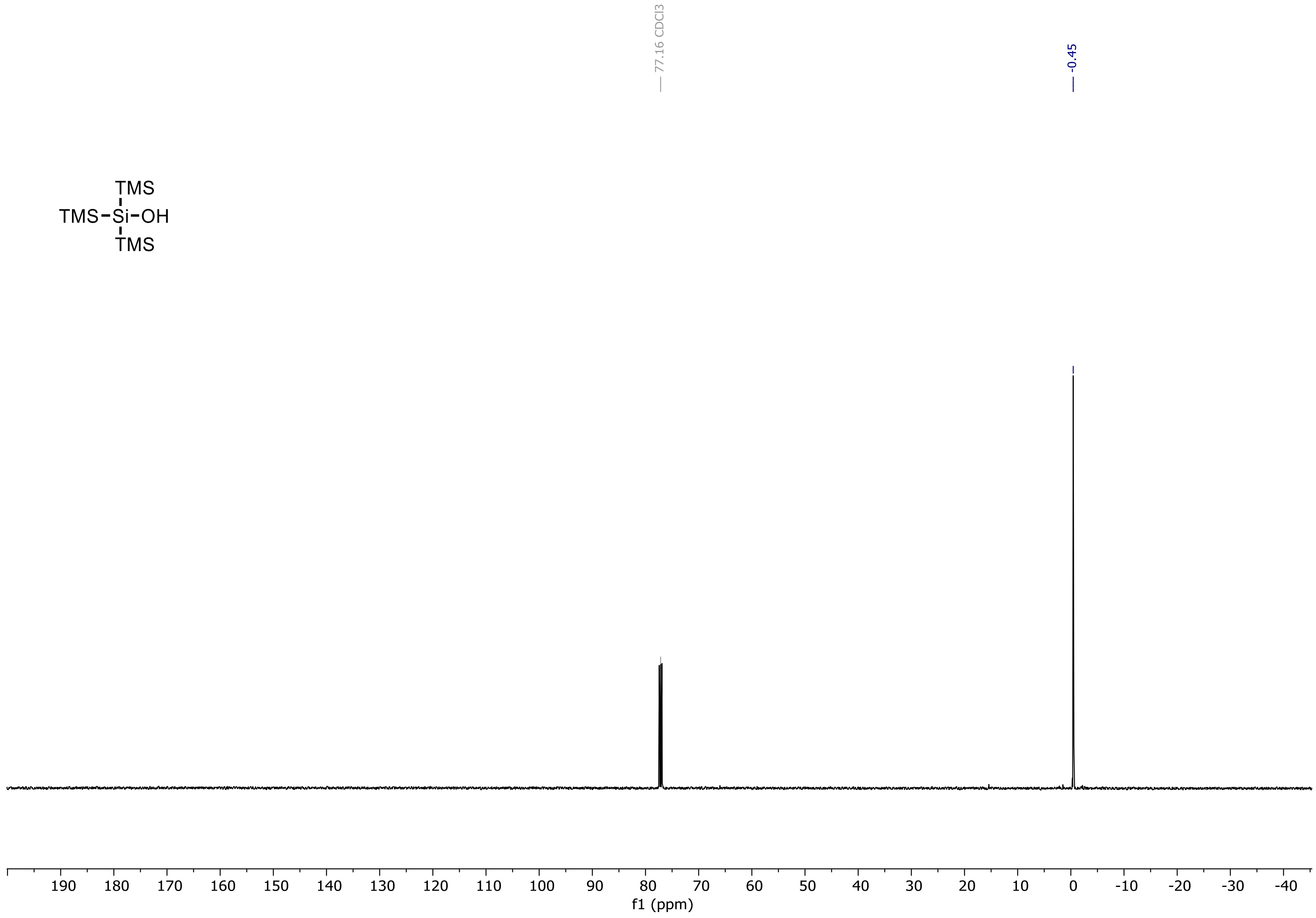

^{1}H NMR of pyrrole derivative S12

$CDCl_3$, 298 K

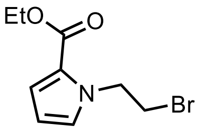

S12

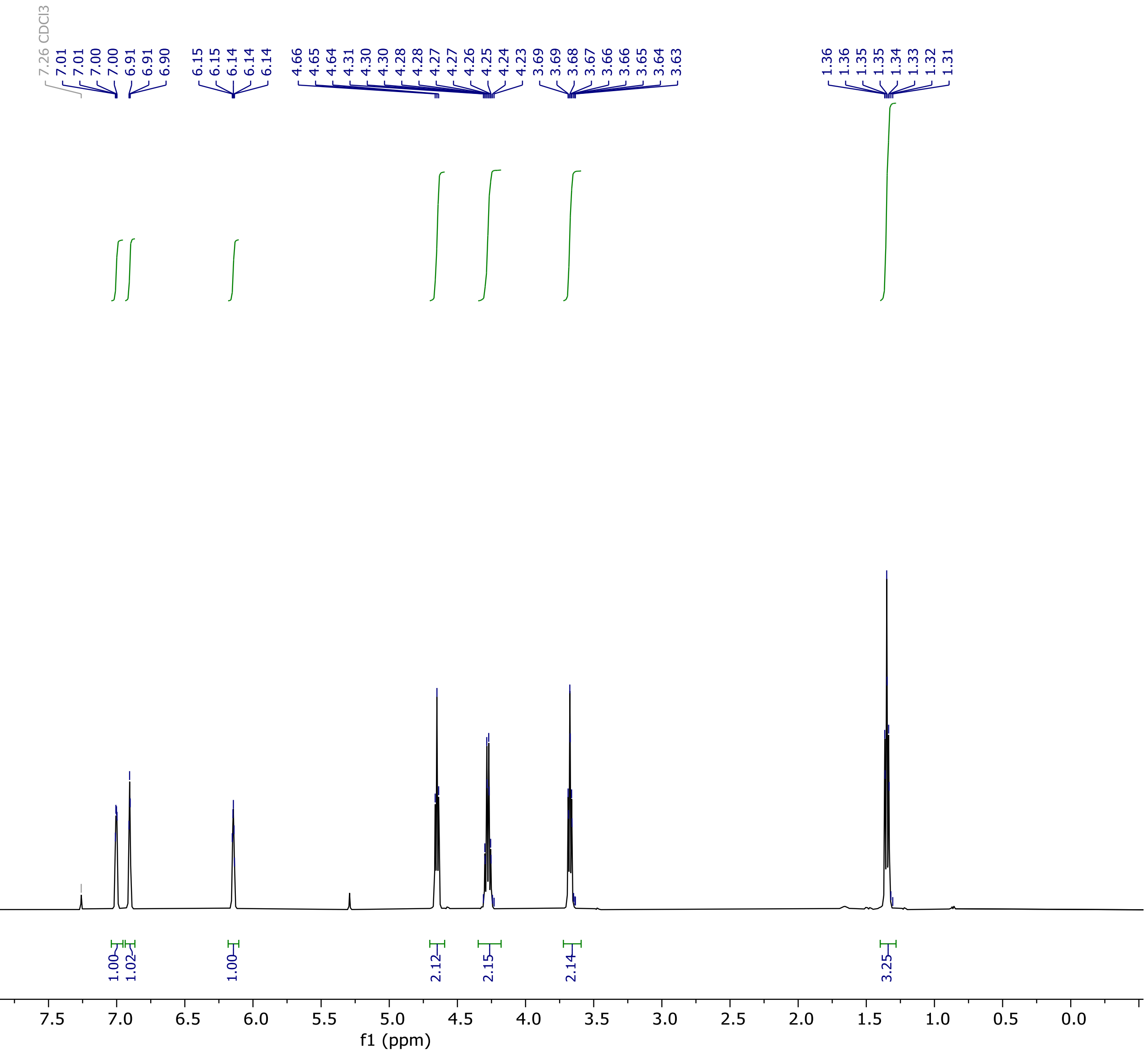

^{13}C NMR of pyrrole derivative S12

$CDCl_3$, 298 K

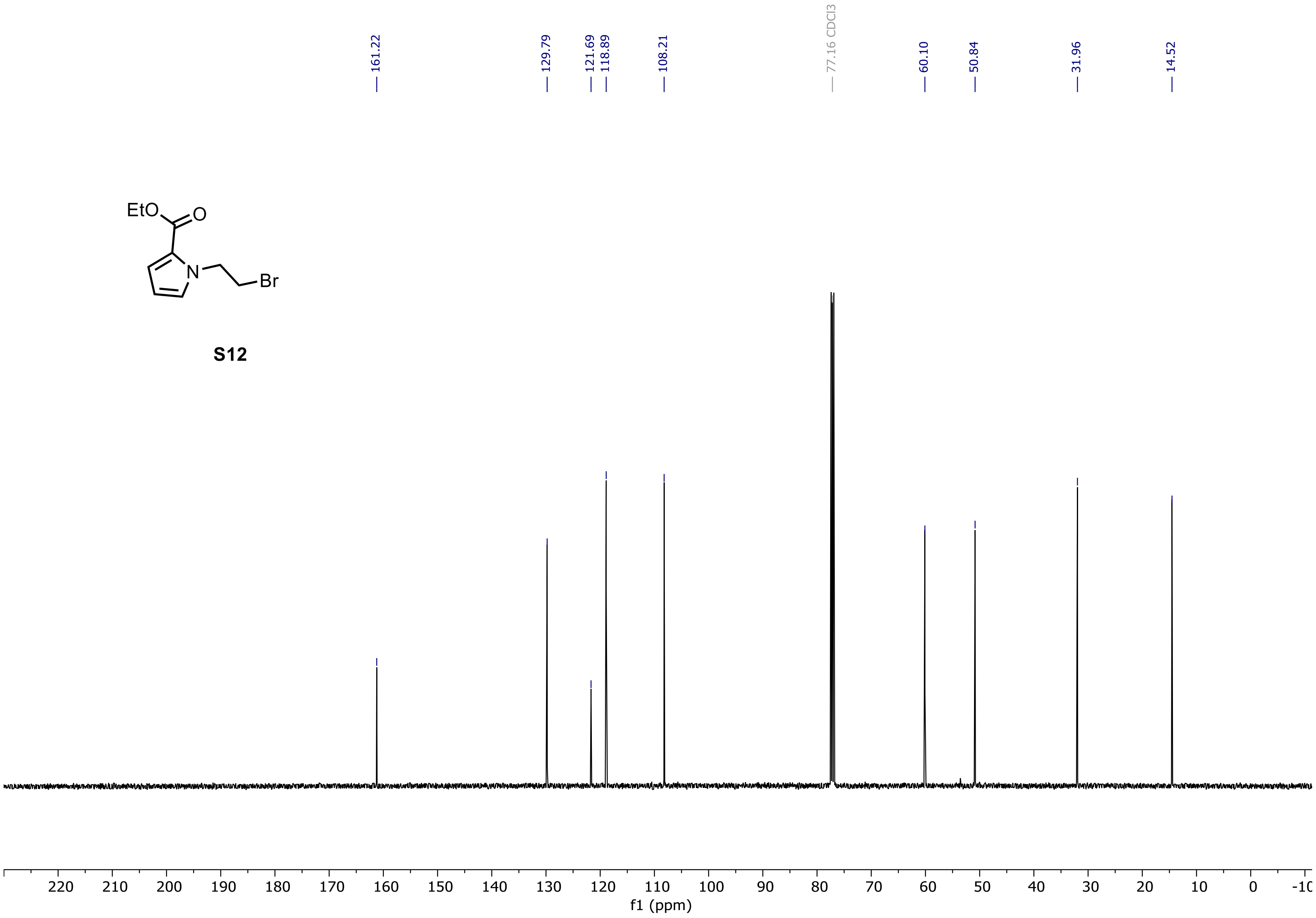

^{1}H NMR of flumequin derivative S13

$CDCl_3$, 298 K

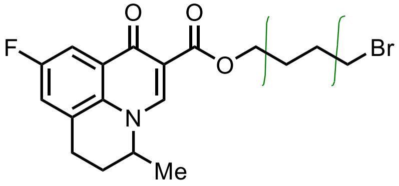

S13

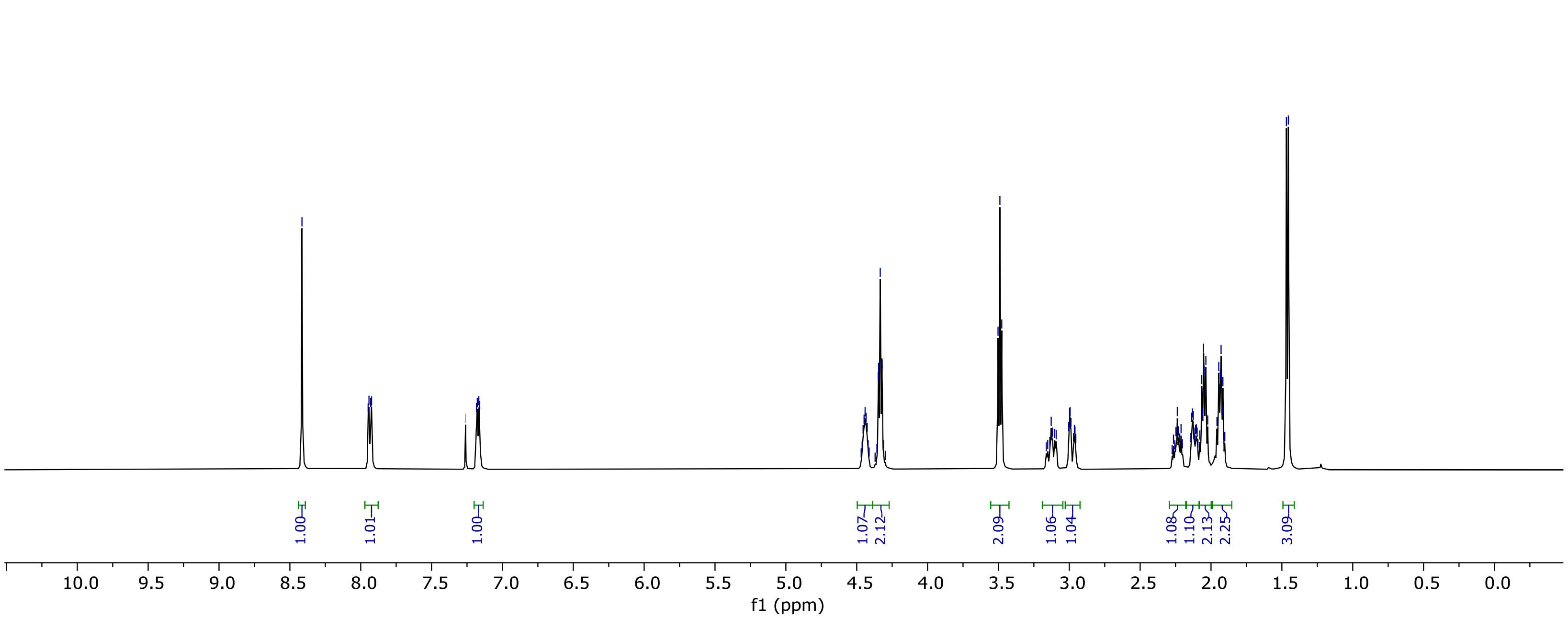

^{13}C NMR of flumequin derivative S13

$CDCl_3$, 298 K

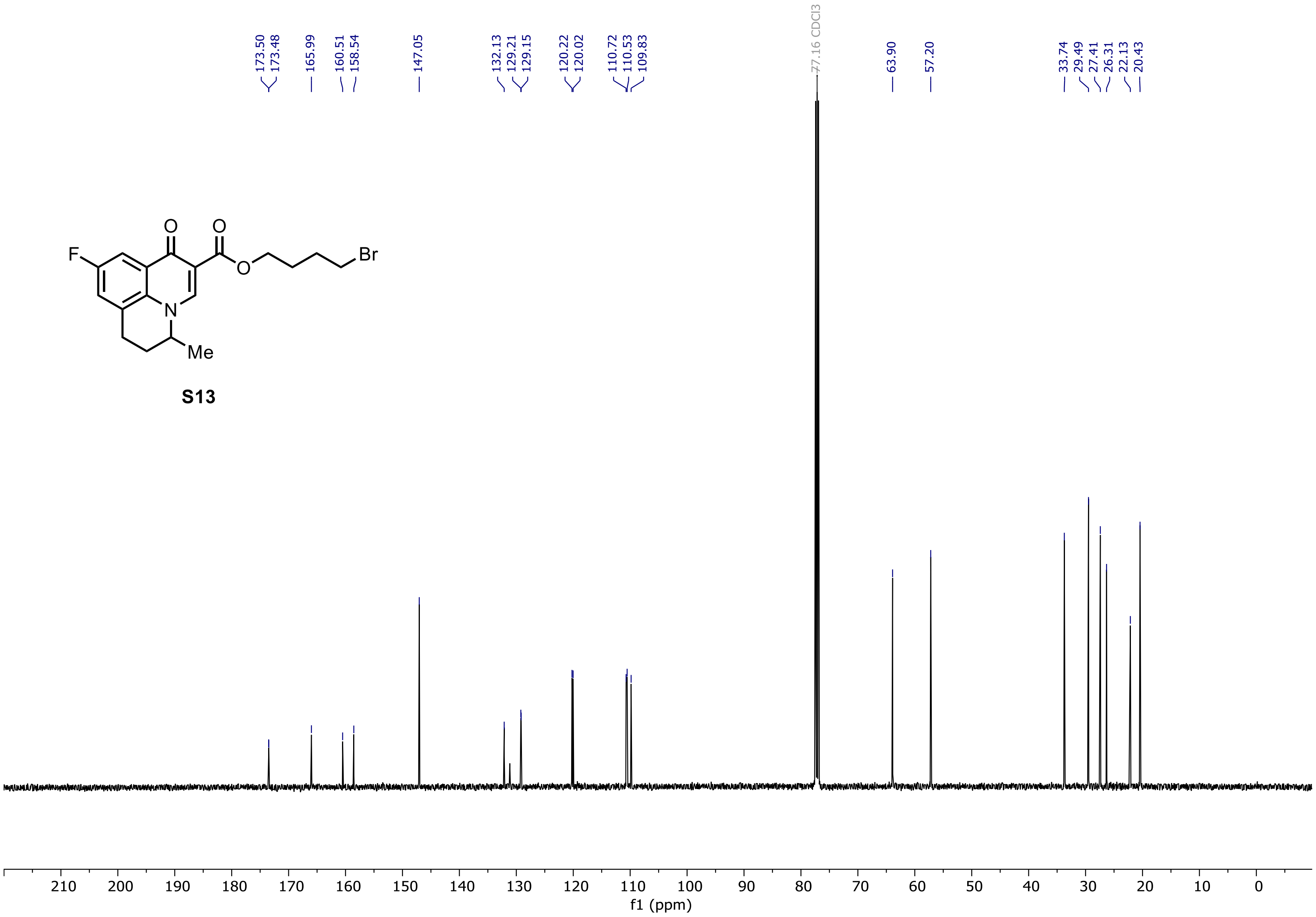

S13

^{19}F NMR of flumequin derivative S13

$CDCl_3$, 298 K

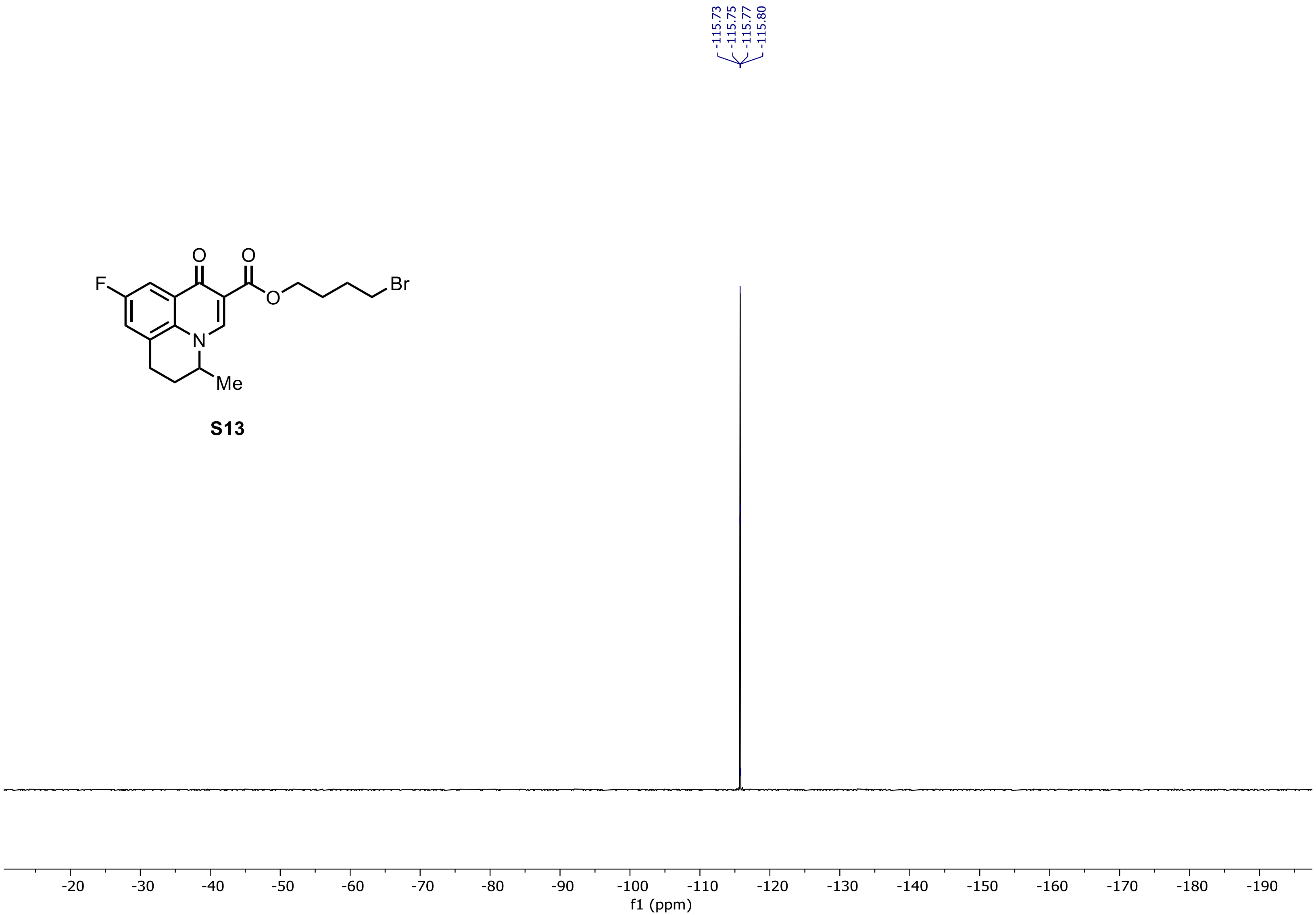

S13

^{1}H NMR of sulbactam derivative S14

CDCl$_3$, 298 K

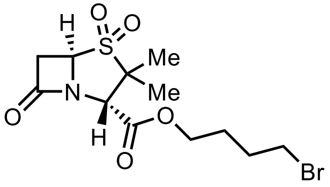

S14

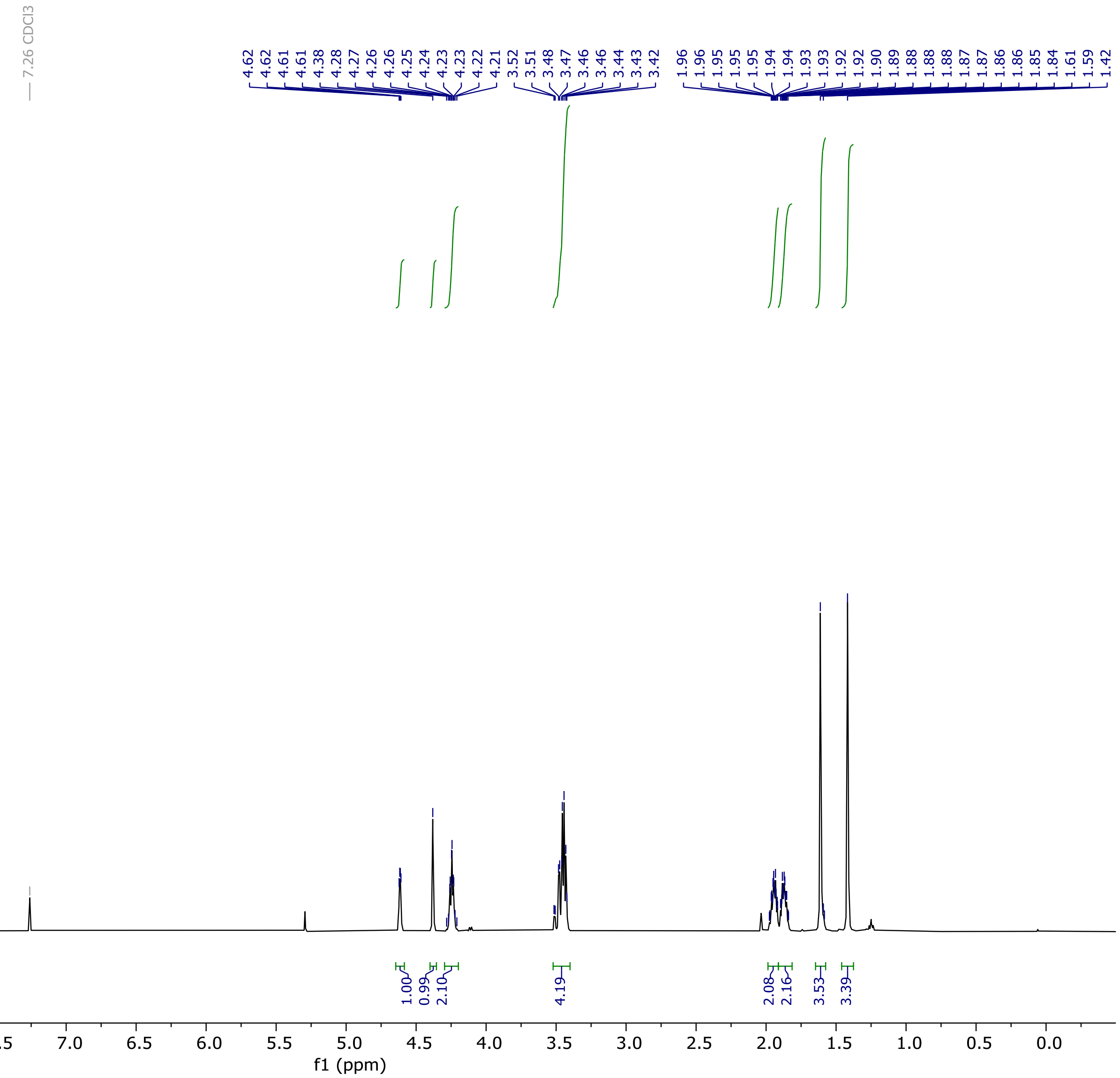

^{13}C NMR of sulbactam derivative S14

CDCl$_3$, 298 K

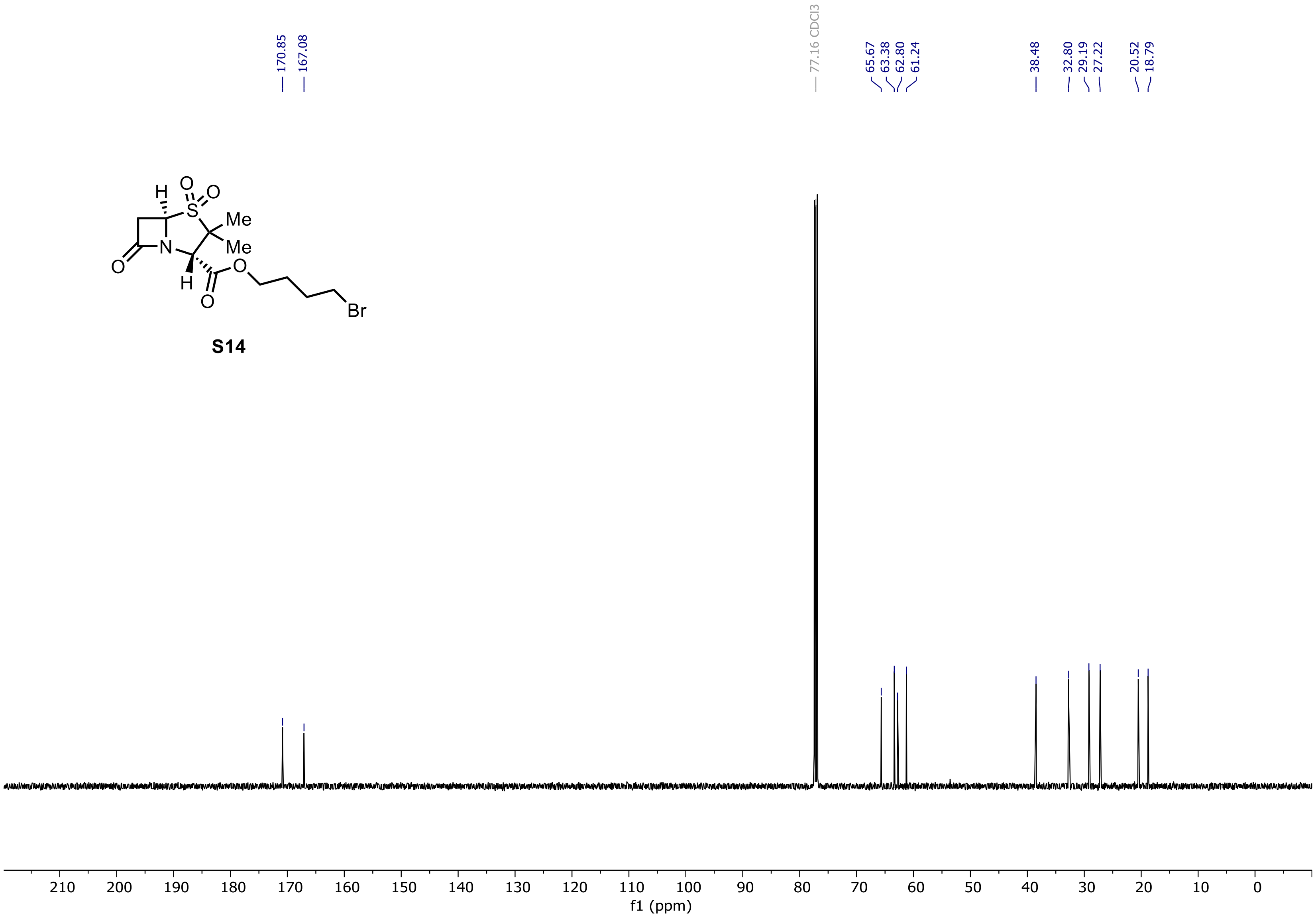

^{1}H NMR of bicyclo[1.1.1]pentylmethylarene 1

$CDCl_3$, 298 K

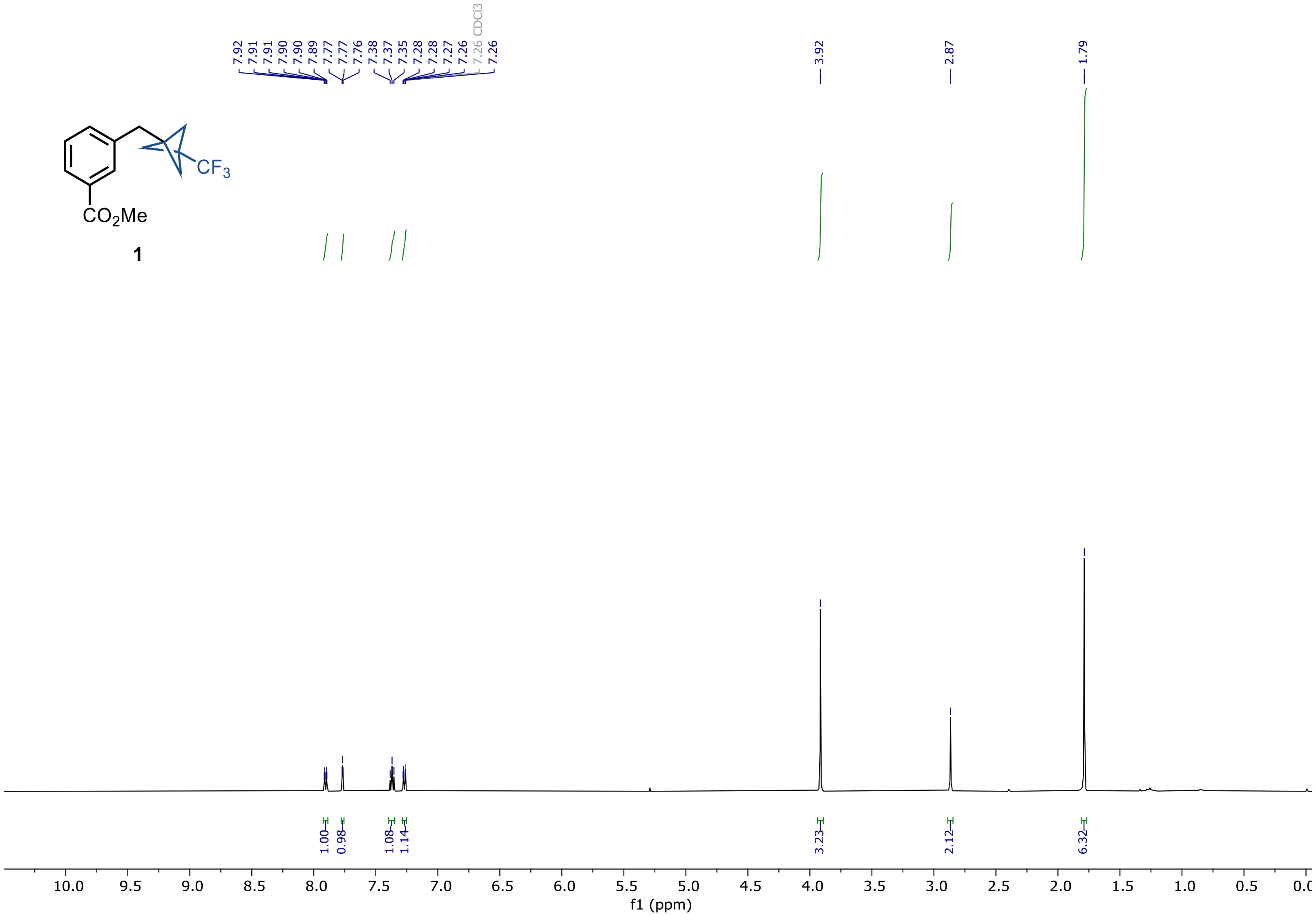

^{13}C NMR of bicyclo[1.1.1]pentylmethylarene 1

$CDCl_3$, 298 K

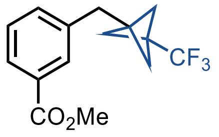

1

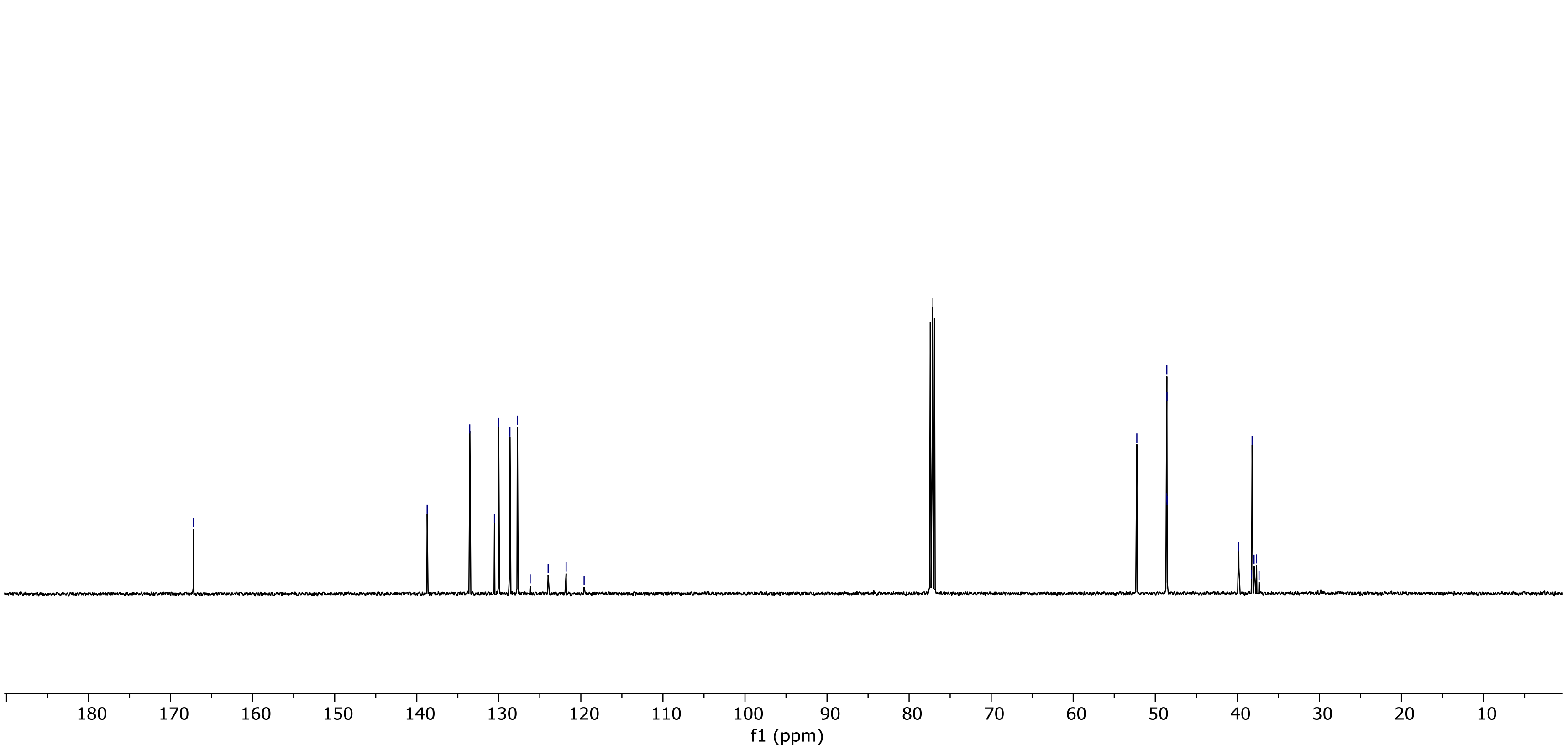

^{19}F NMR of bicyclo[1.1.1]pentylmethylarene 1

$CDCl_3$, 298 K

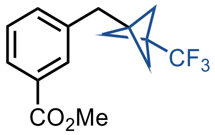

1

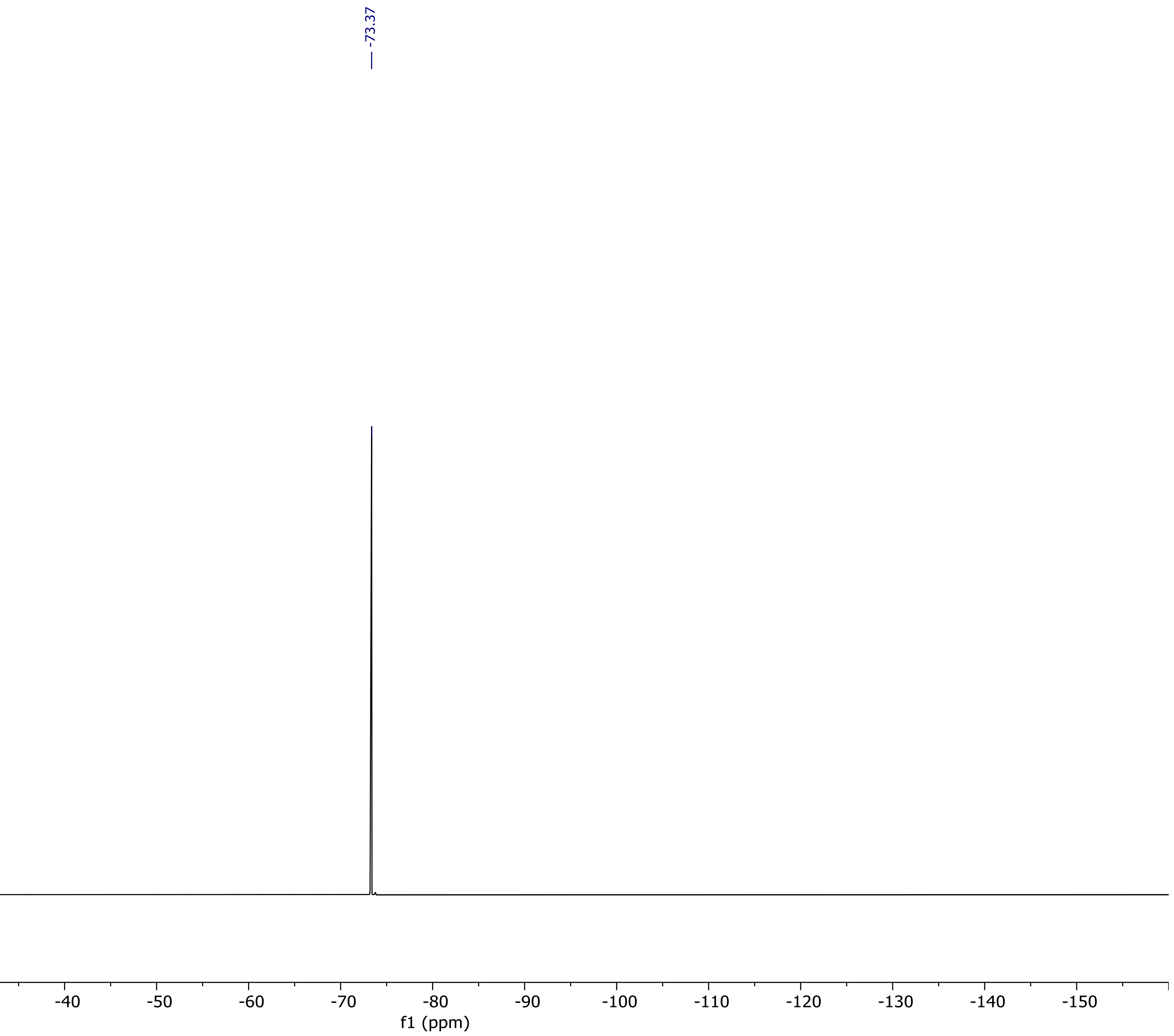

^{1}H NMR of bicyclo[1.1.1]pentylmethylarene 2

CDCl$_3$, 298 K

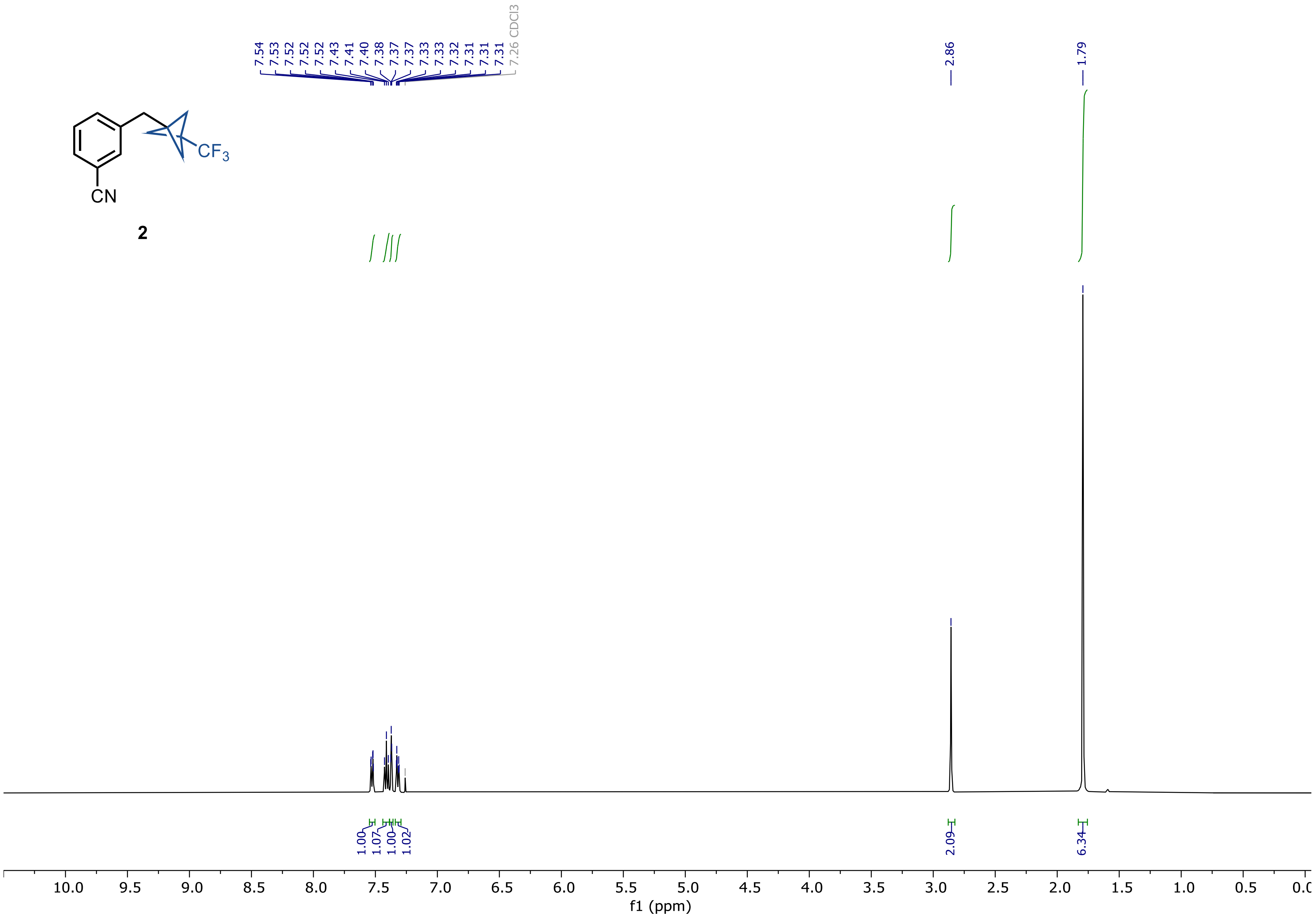

^{13}C NMR of bicyclo[1.1.1]pentylmethylarene 2

$CDCl_3$, 298 K

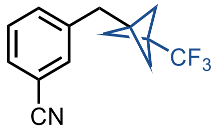

2

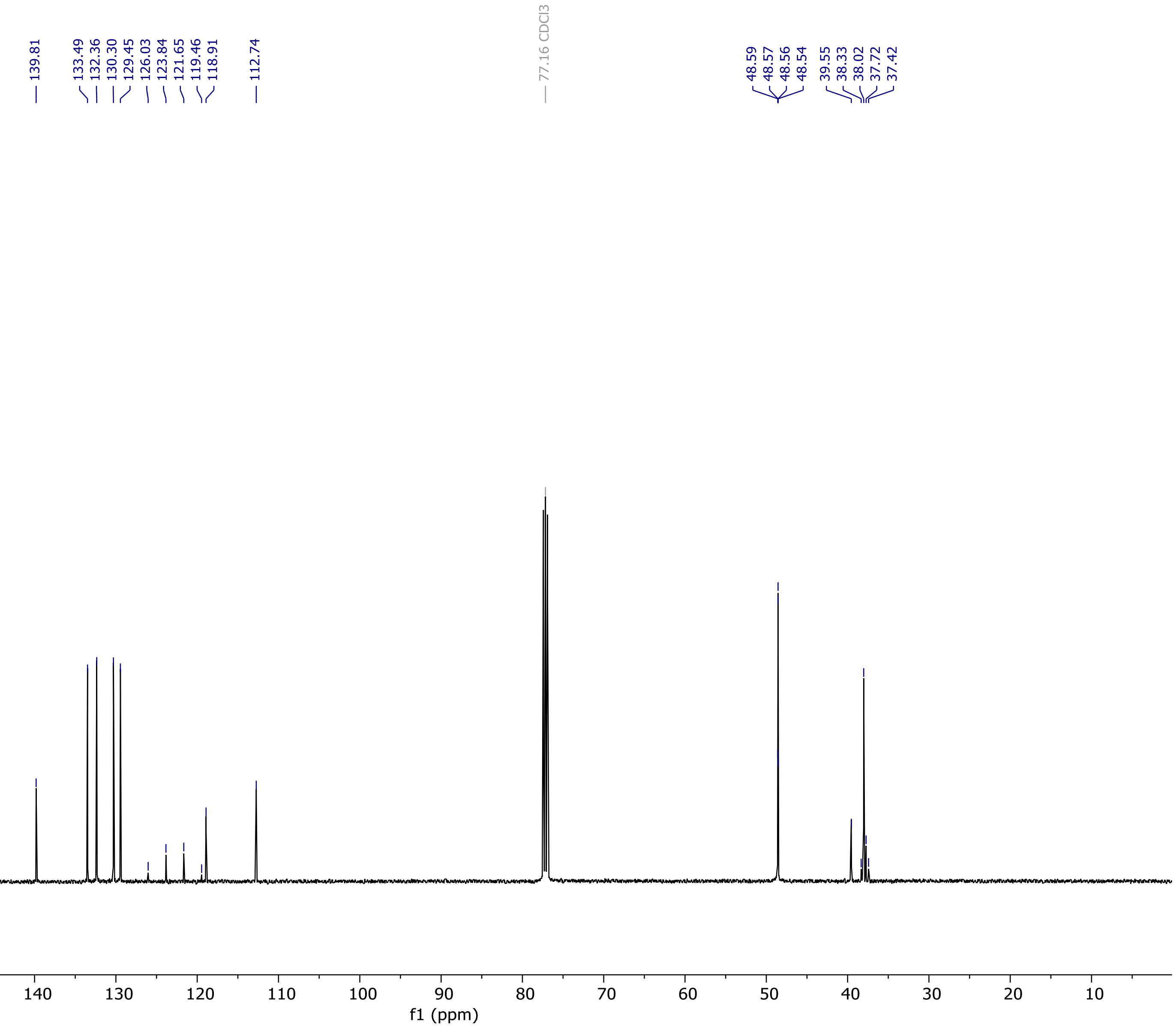

^{19}F NMR of bicyclo[1.1.1]pentylmethylarene 2

$CDCl_3$, 298 K

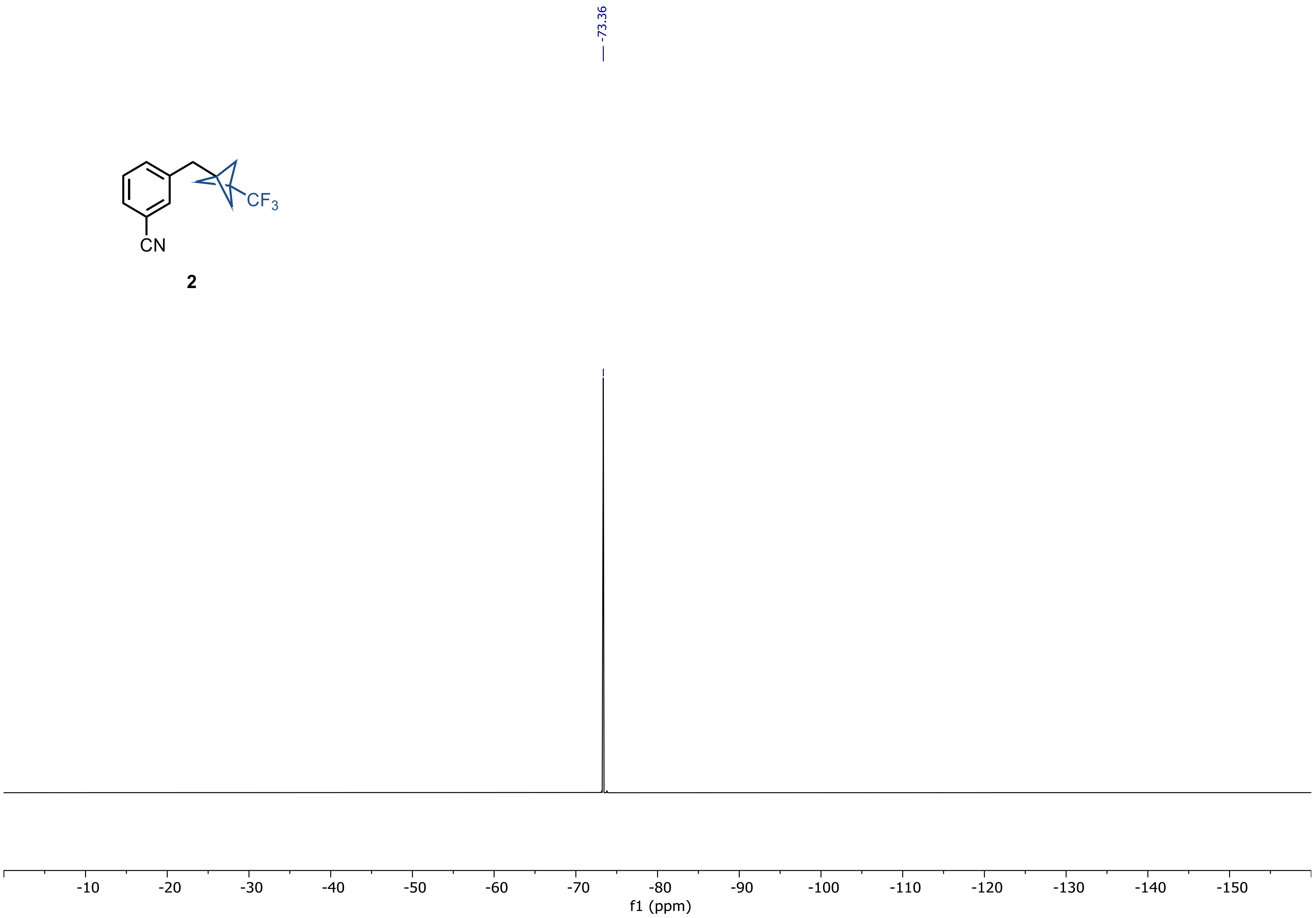

^{1}H NMR of bicyclo[1.1.1]pentylmethylpyridine 3

$CDCl_3$, 298 K

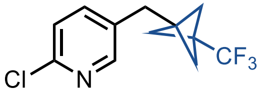

3

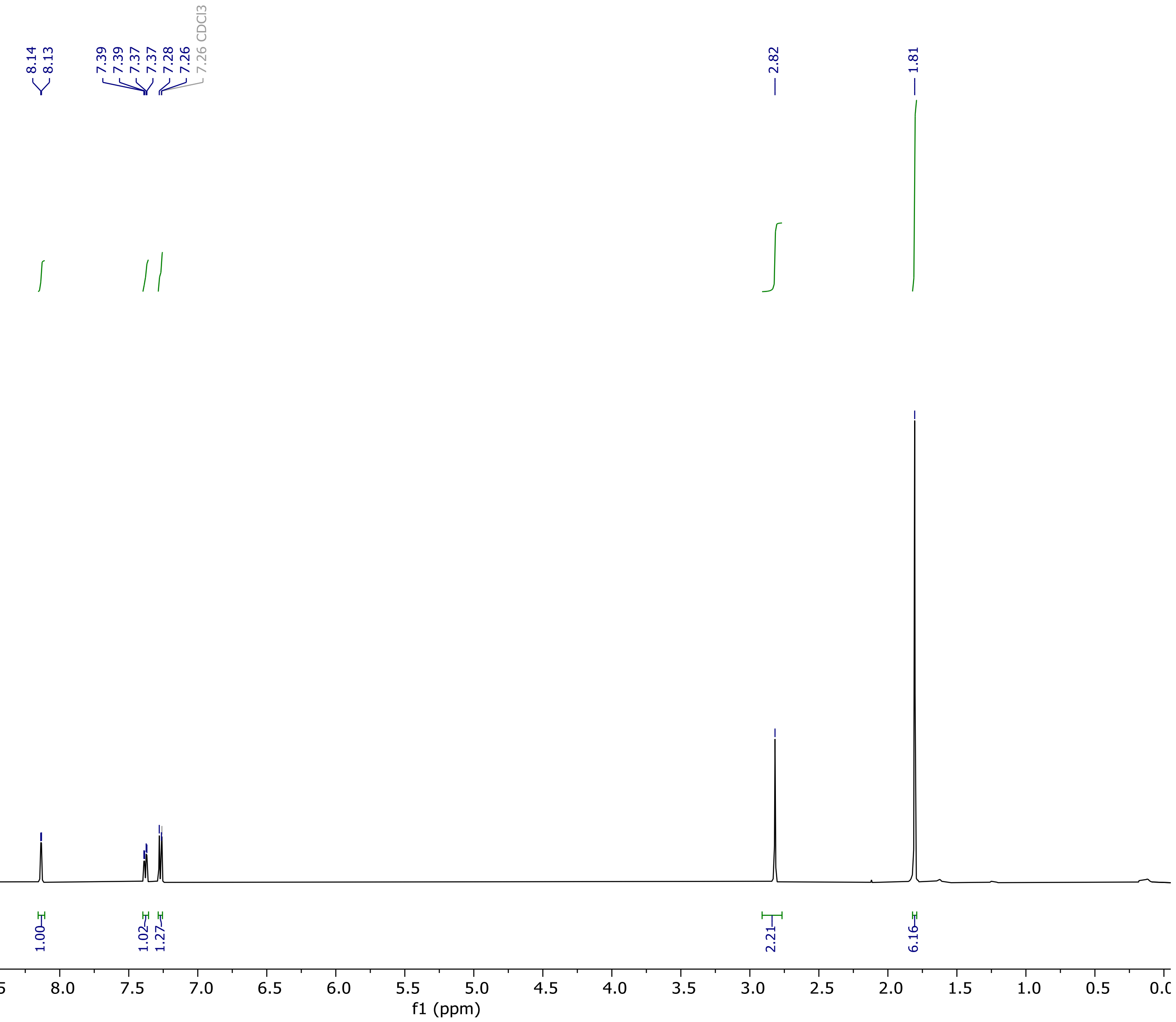

^{13}C NMR of bicyclo[1.1.1]pentylmethylpyridine 3

$CDCl_3$, 298 K

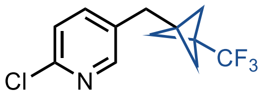

3

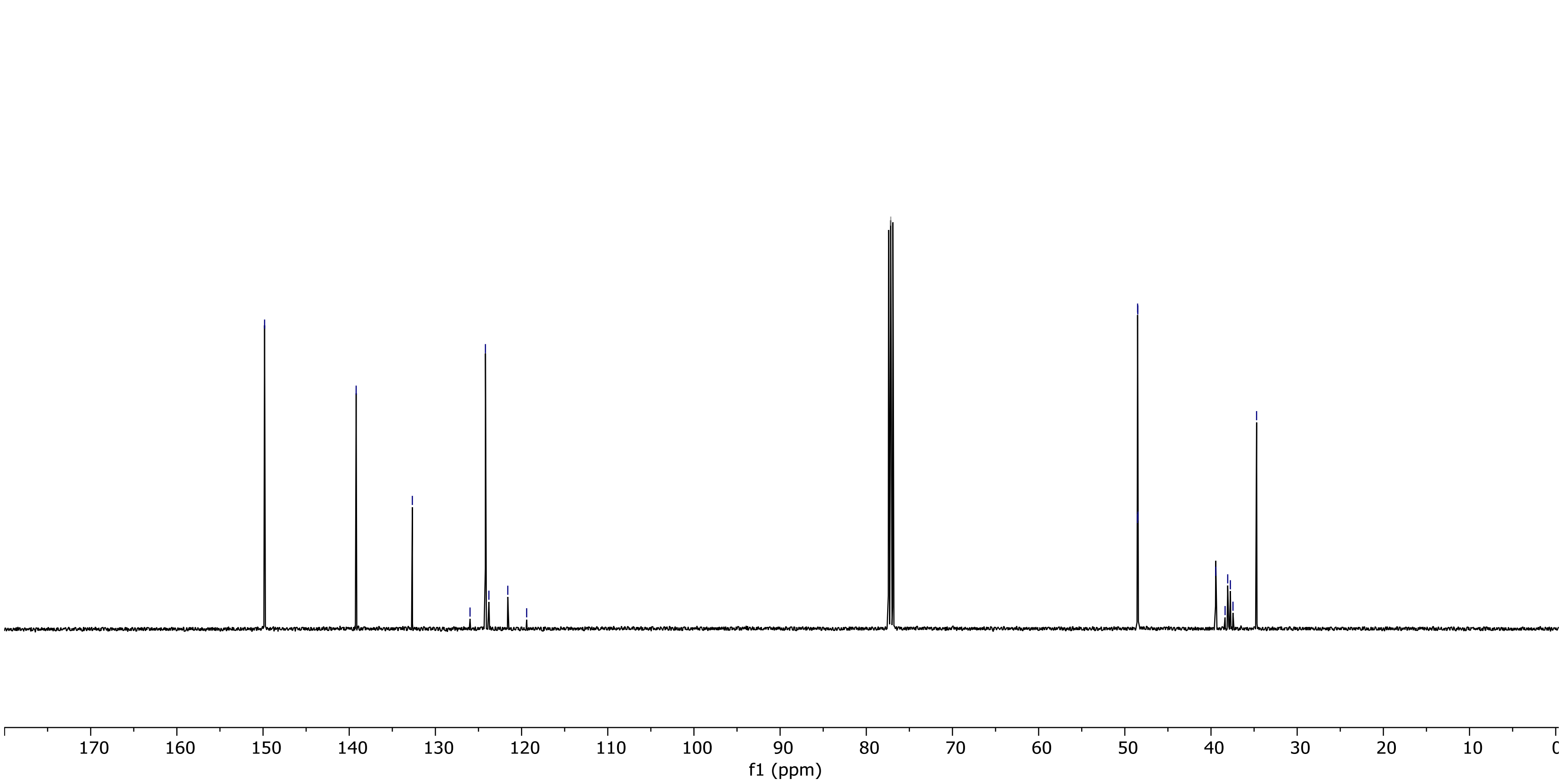

^{19}F NMR of bicyclo[1.1.1]pentylmethylpyridine 3

CDCl$_3$, 298 K

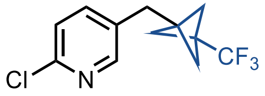

3

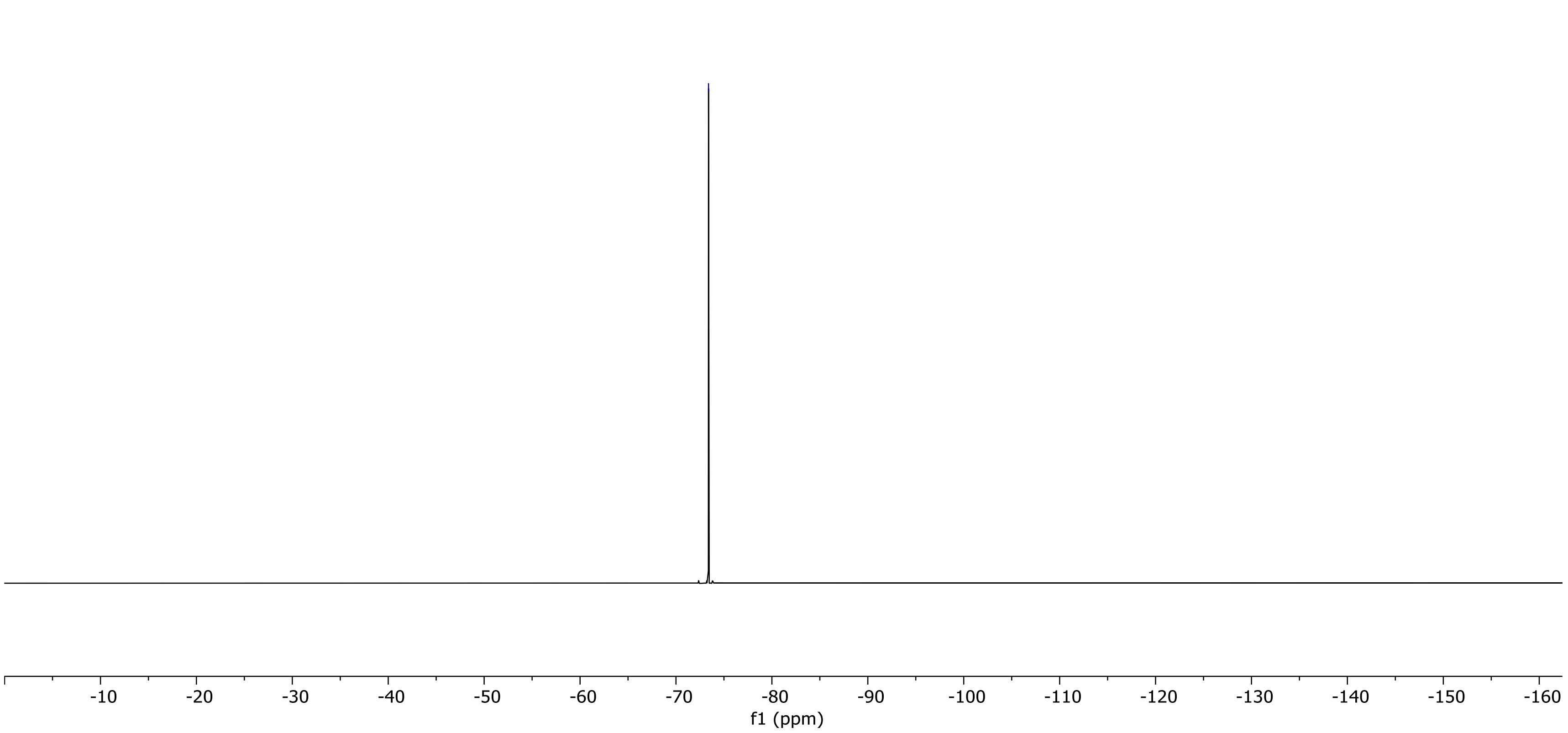

^{1}H NMR of bicyclo[1.1.1]pentylmethylarene 4

$CDCl_3$, 298 K

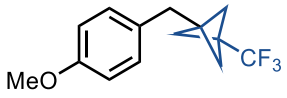

4

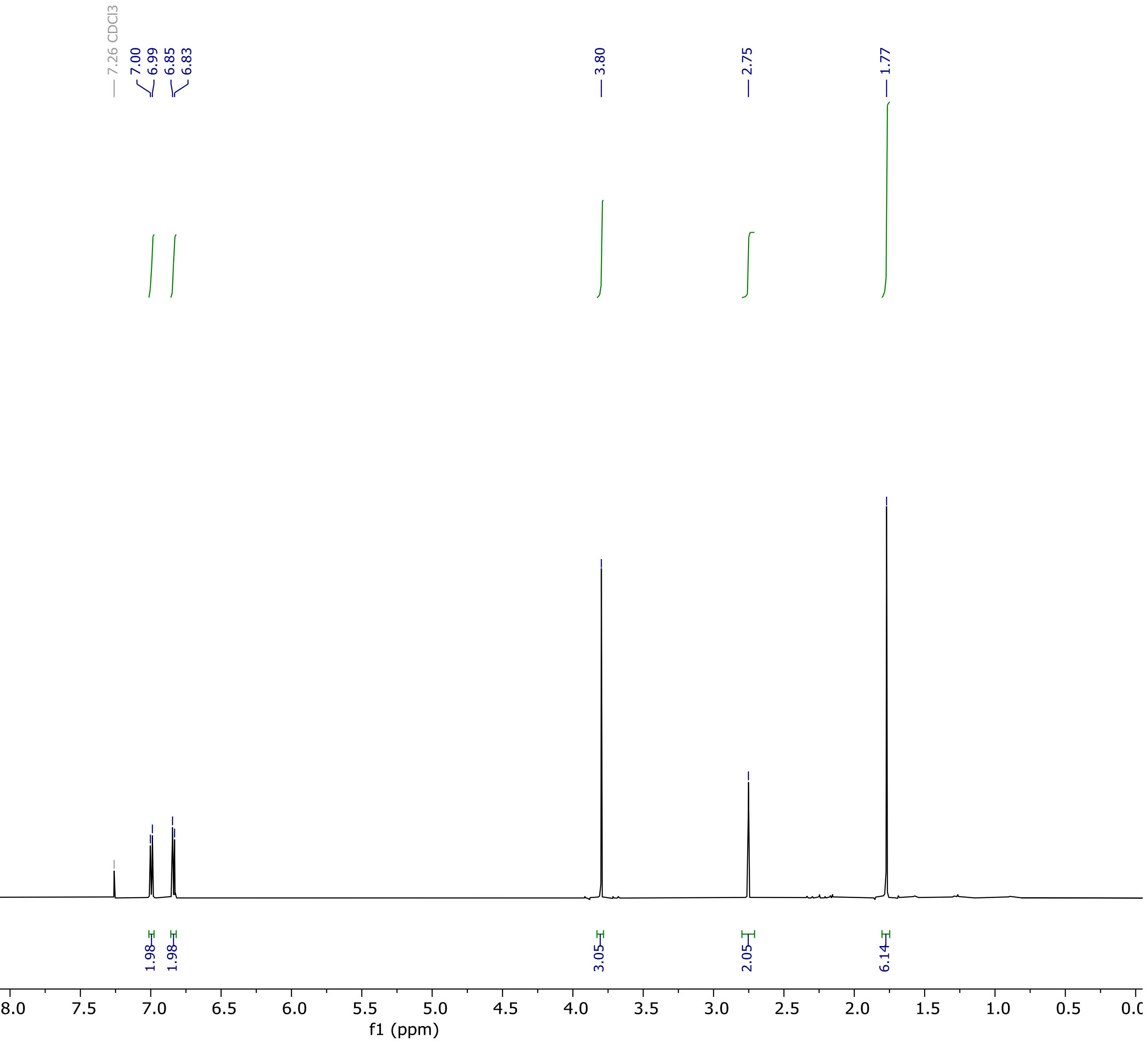

^{13}C NMR of bicyclo[1.1.1]pentylmethylarene 4

$CDCl_3$, 298 K

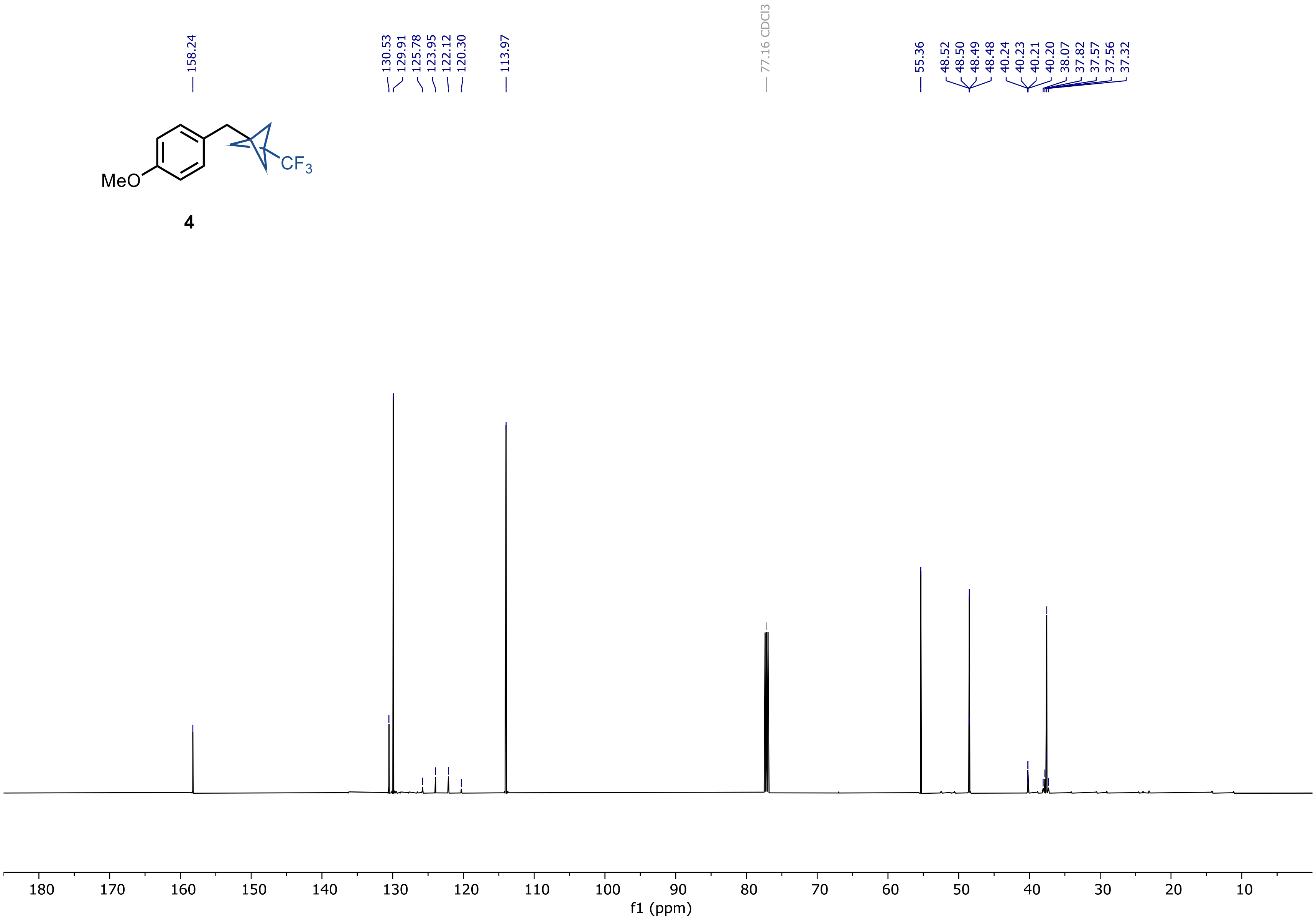

^{19}F NMR of bicyclo[1.1.1]pentylmethylarene 4

CDCl$_3$, 298 K

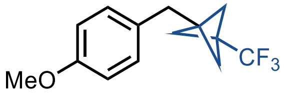

4

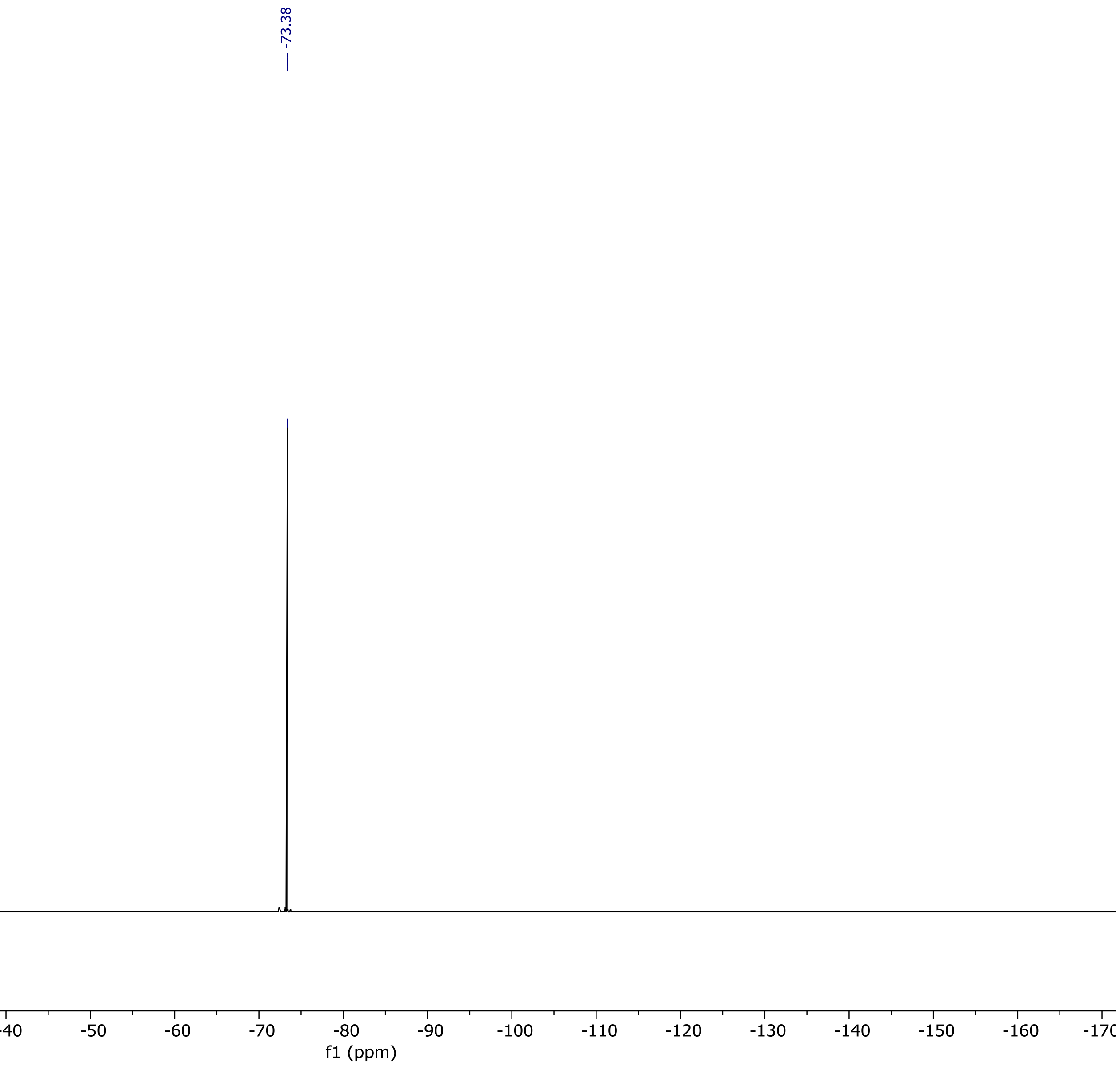

^{1}H NMR of bicyclo[1.1.1]pentylmethylarene 5

$CDCl_3$, 298 K

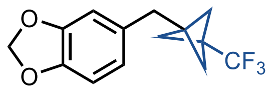

5

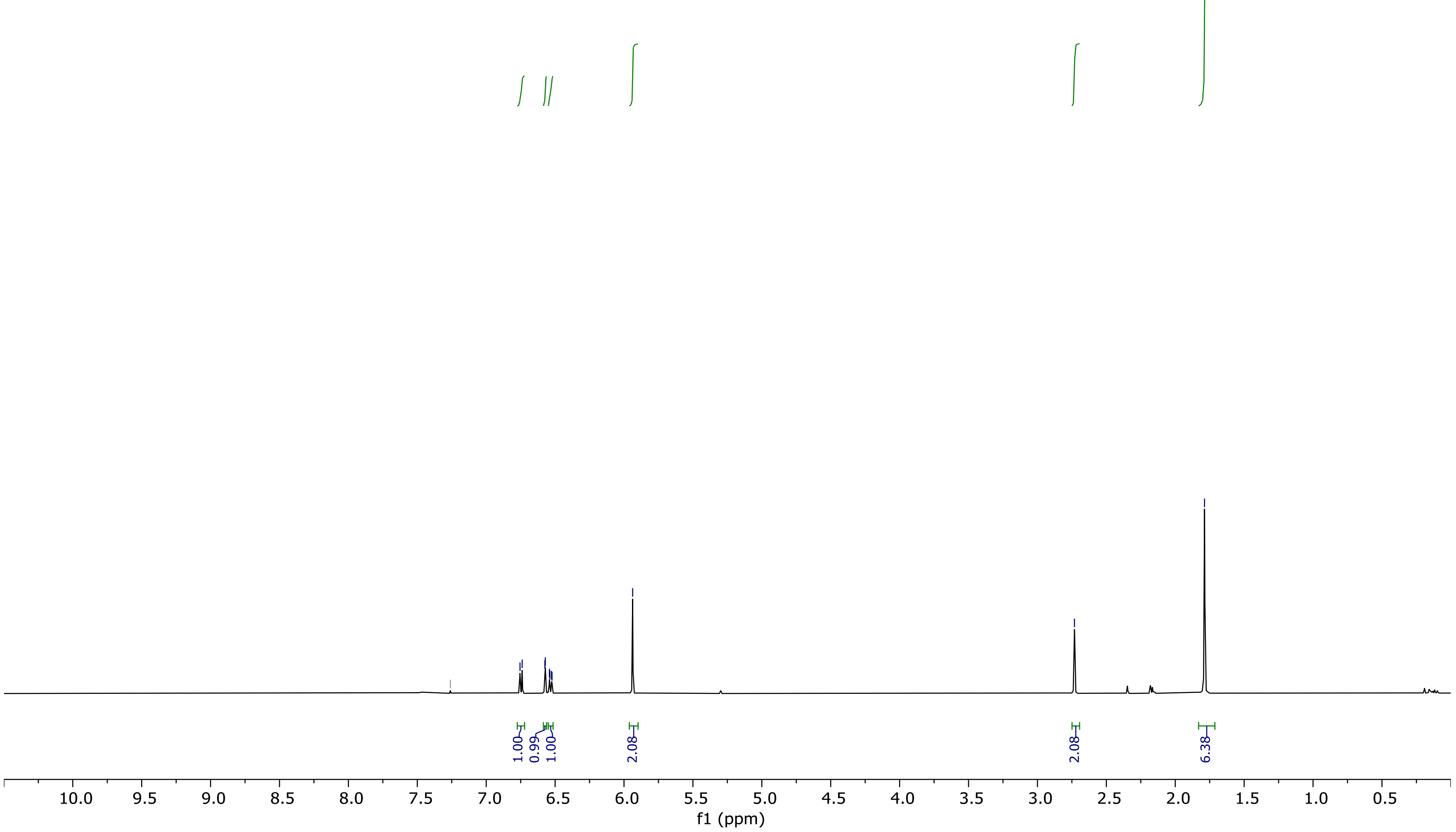

^{13}C NMR of bicyclo[1.1.1]pentylmethylarene 5

$CDCl_3$, 298 K

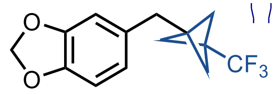

5

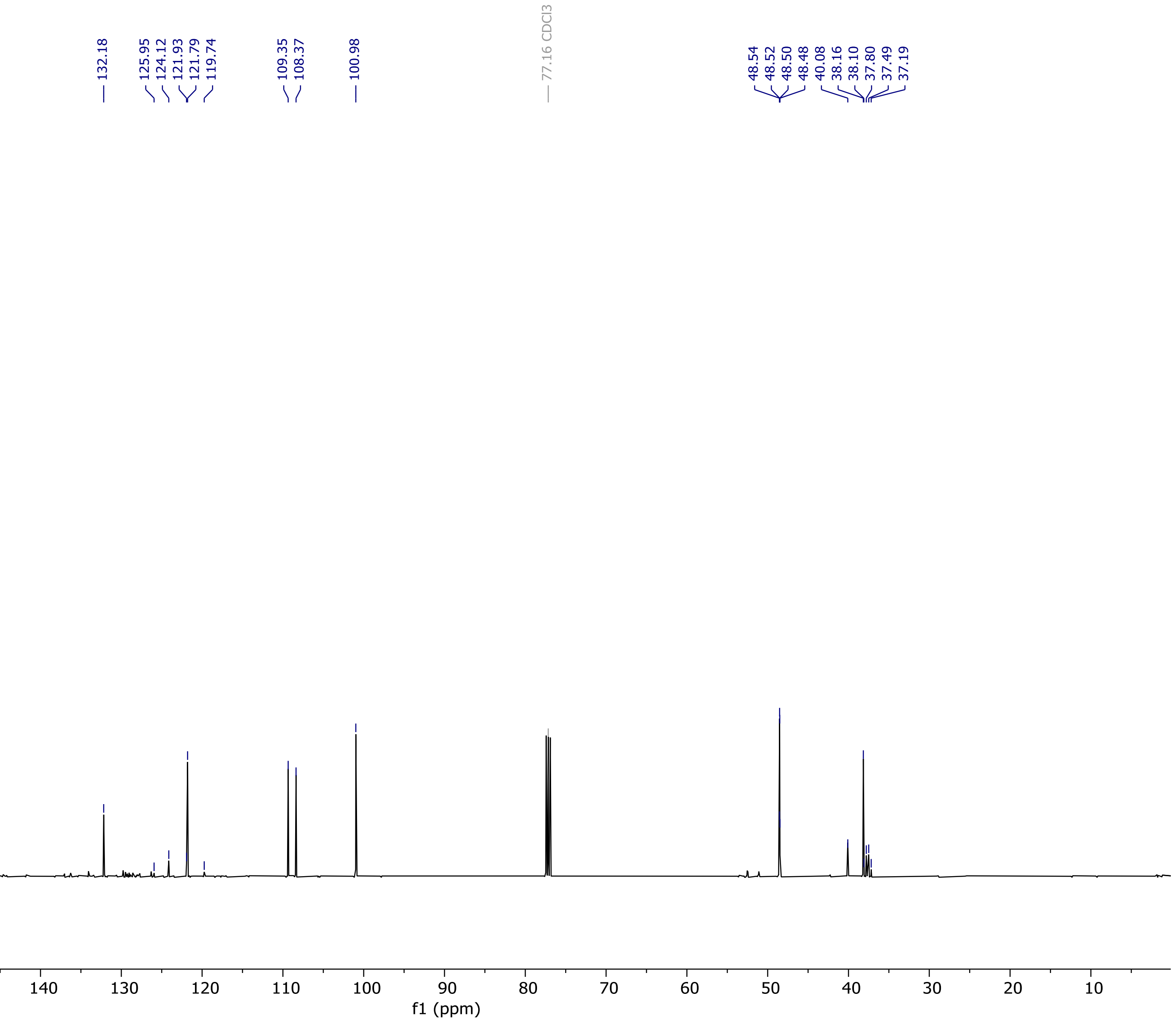

^{19}F NMR of bicyclo[1.1.1]pentylmethylarene 5

$CDCl_3$, 298 K

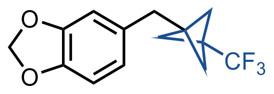

5

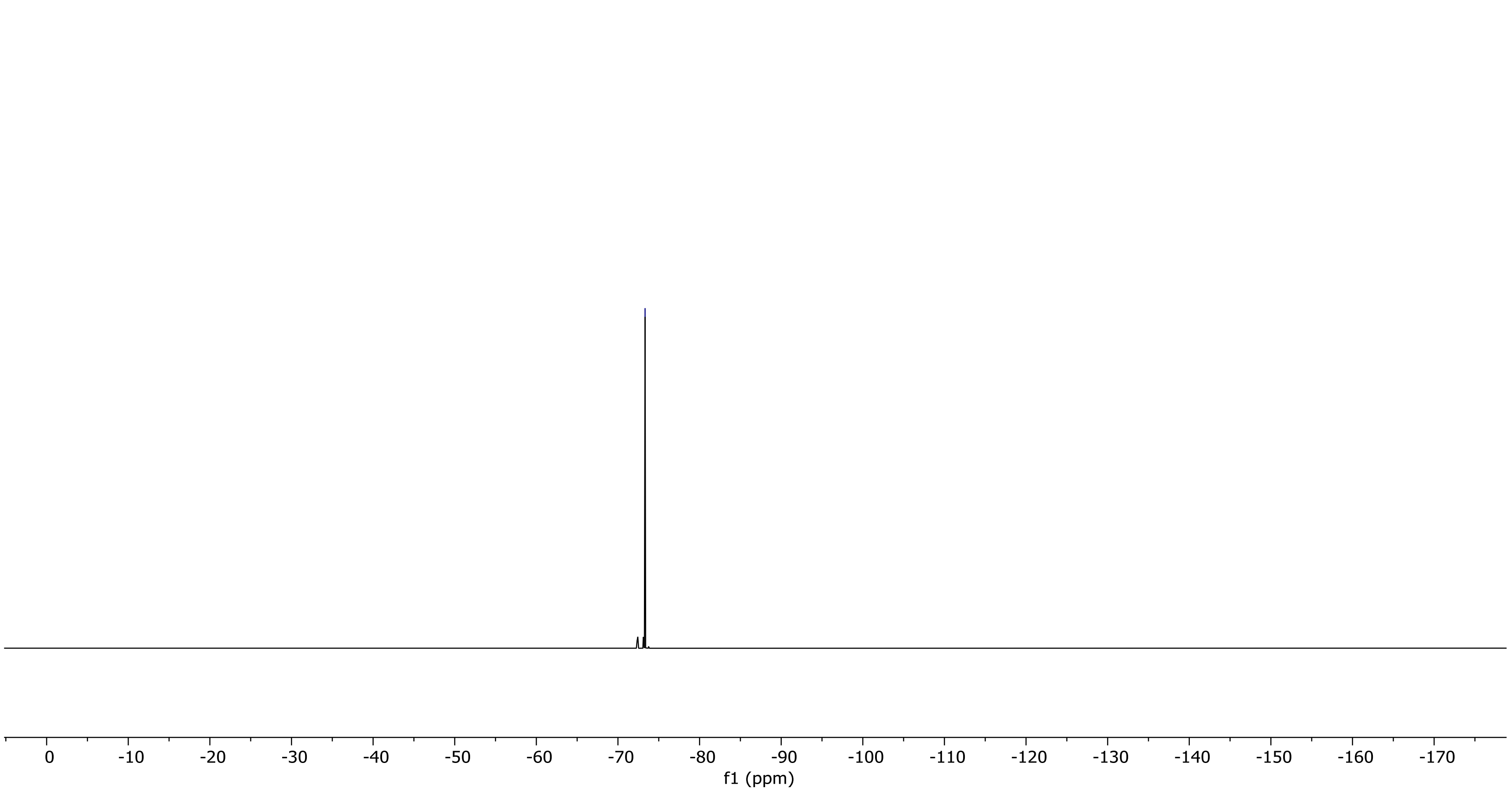

^{1}H NMR of bicyclo[1.1.1]pentylmethylarene 6

$CDCl_3$, 298 K

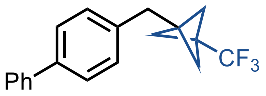

6

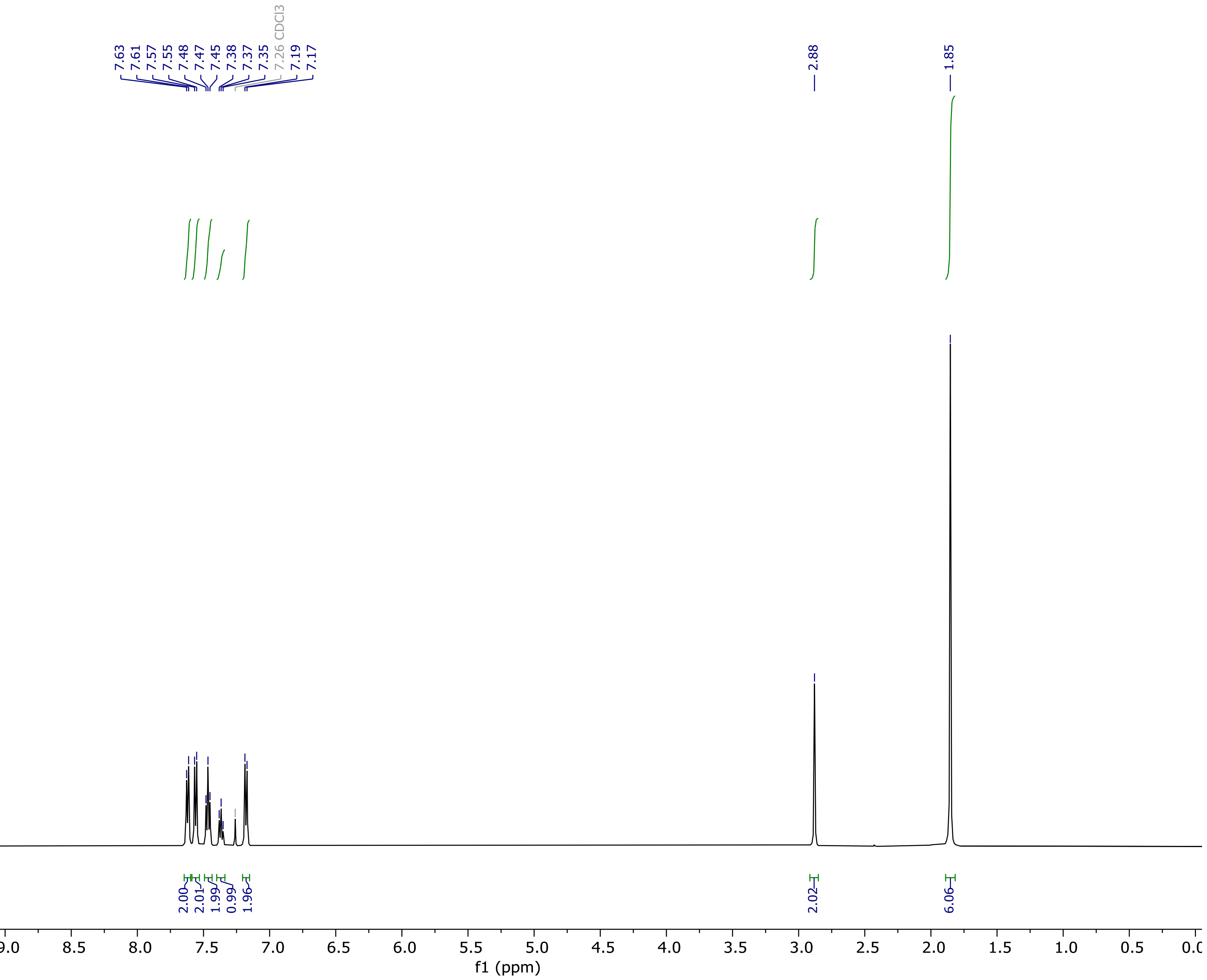

^{13}C NMR of bicyclo[1.1.1]pentylmethylarene 6

$CDCl_3$, 298 K

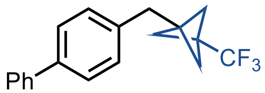

6

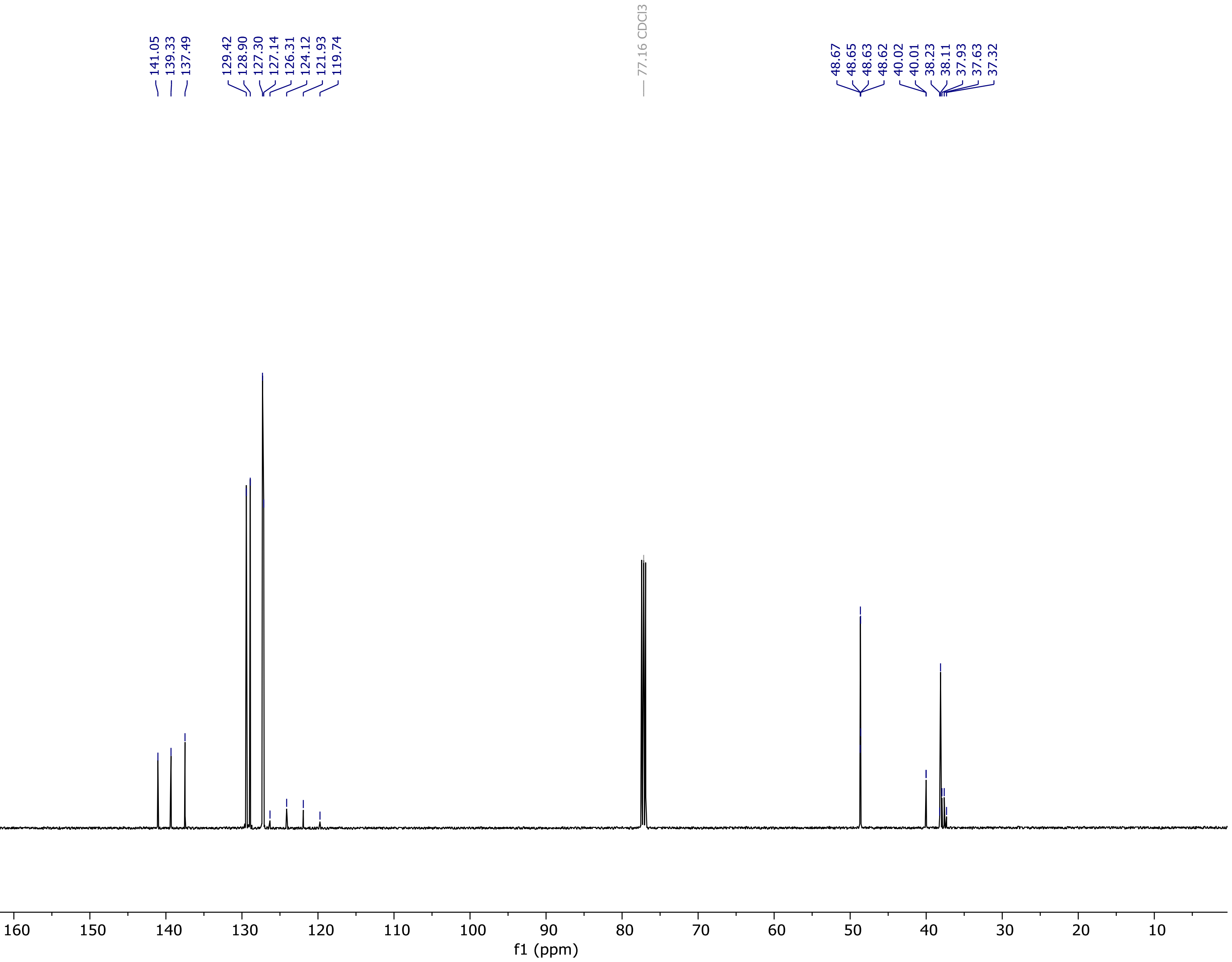

^{19}F NMR of bicyclo[1.1.1]pentylmethylarene 6

CDCl$_3$, 298 K

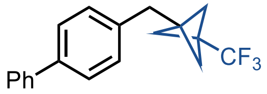

6

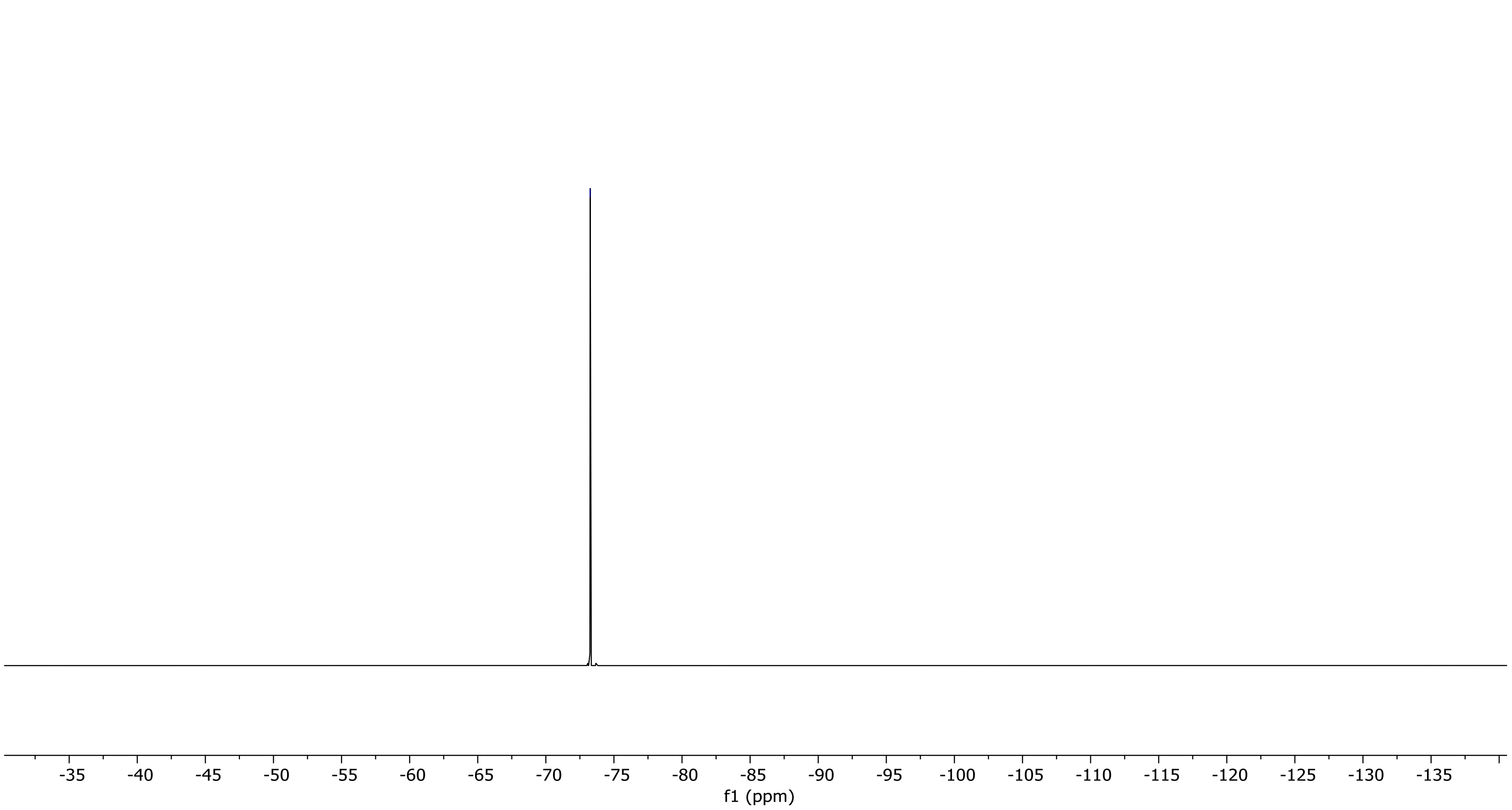

^{1}H NMR of bicyclo[1.1.1]pentylmethylarene 7

CDCl3, 298 K

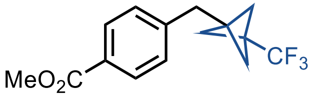

7

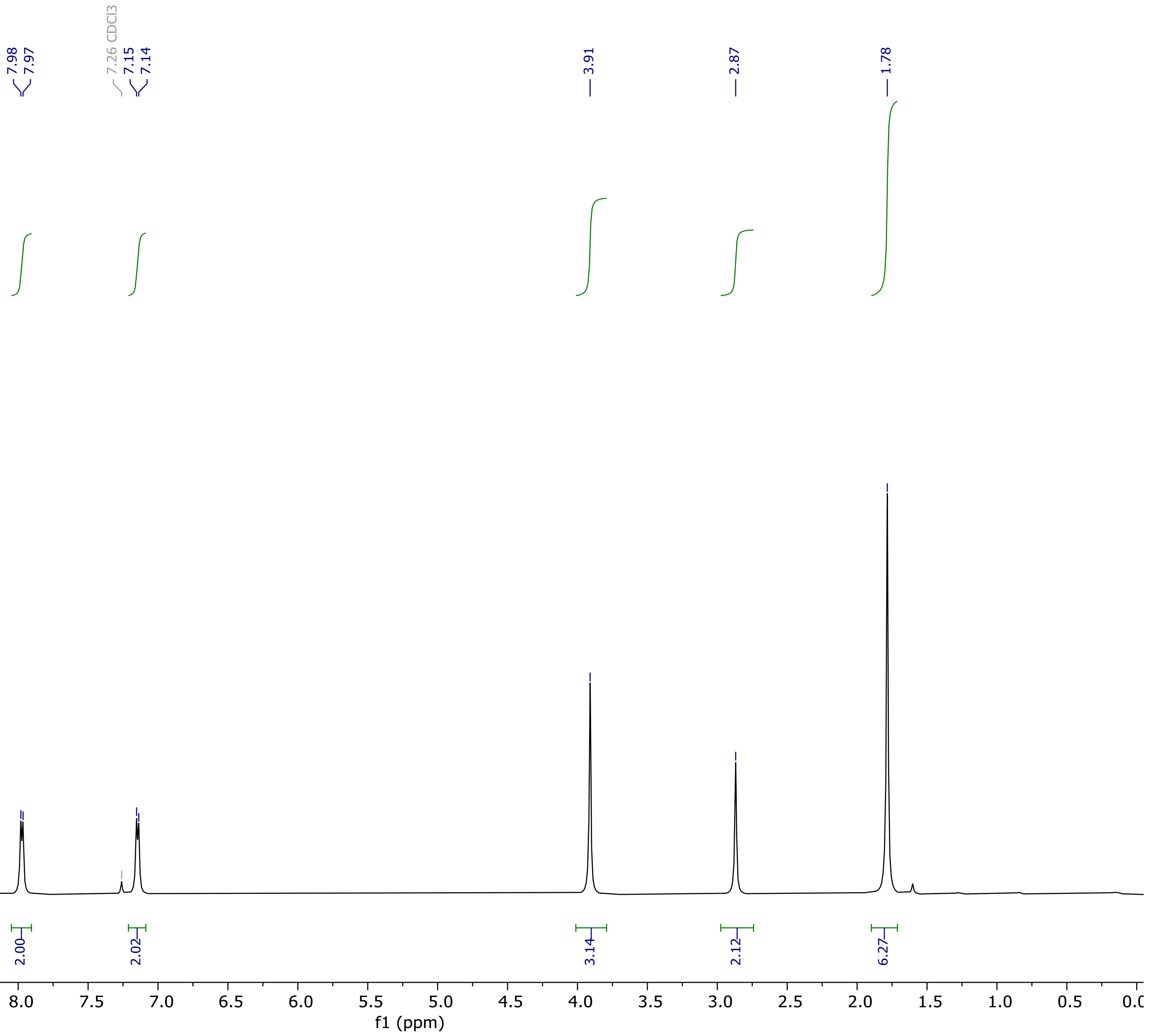

^{13}C NMR of bicyclo[1.1.1]pentylmethylarene 7

$CDCl_3$, 298 K

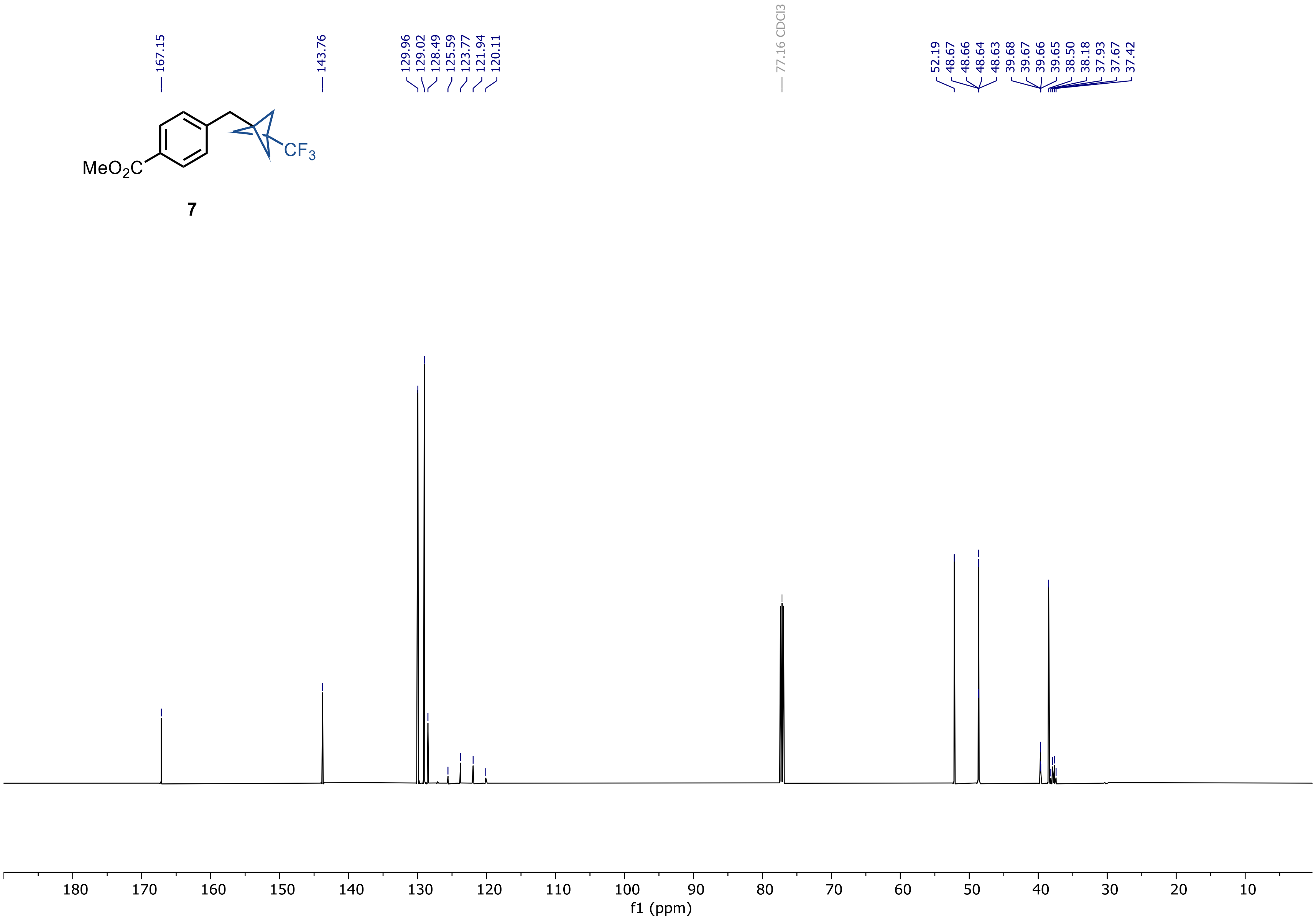

^{19}F NMR of bicyclo[1.1.1]pentylmethylarene 7

$CDCl_3$, 298 K

-73.37

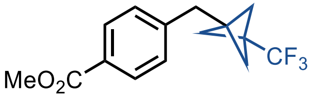

7

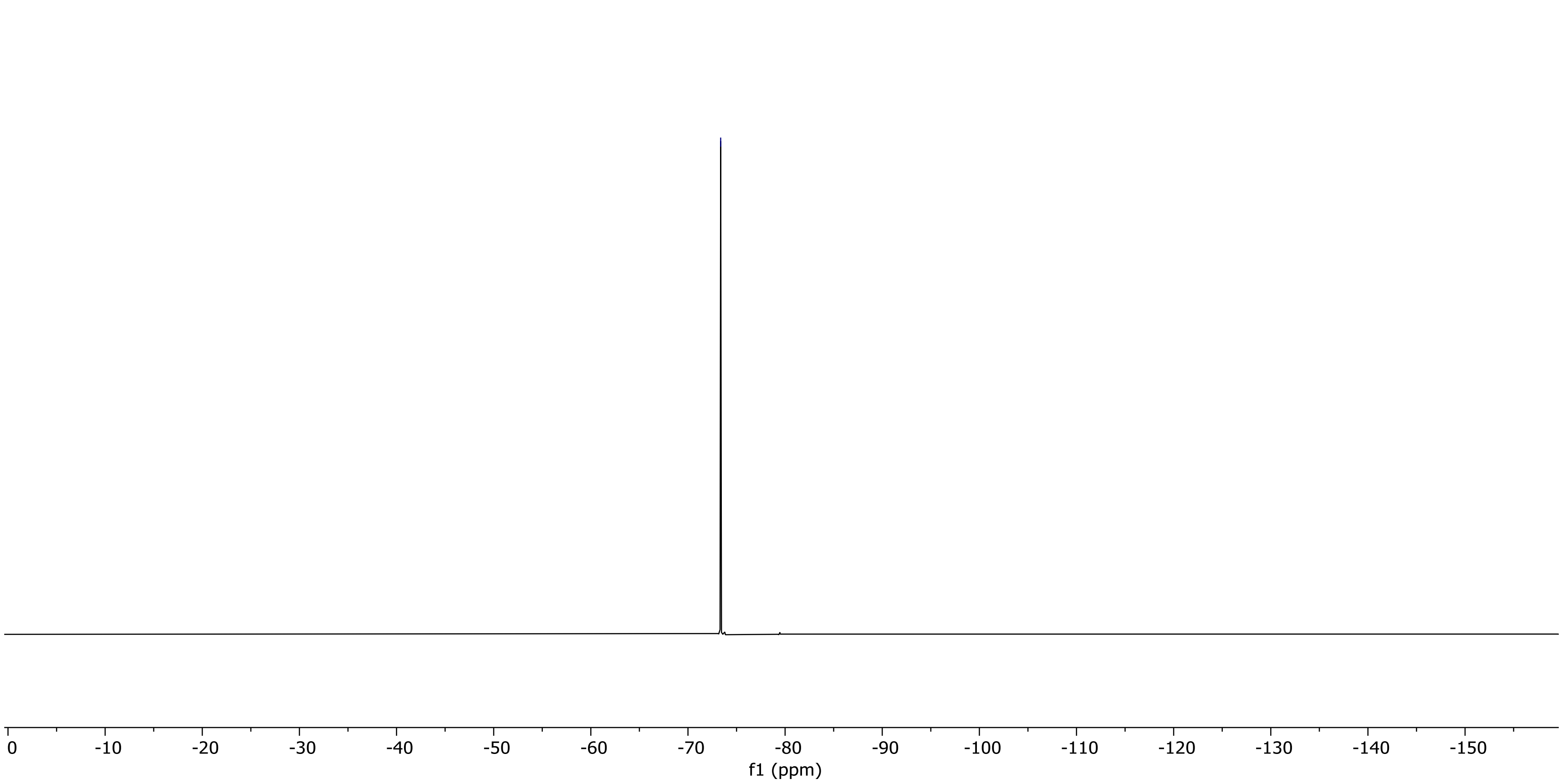

^{1}H NMR of bicyclo[1.1.1]pentylmethylarene 8

$CDCl_3$, 298 K

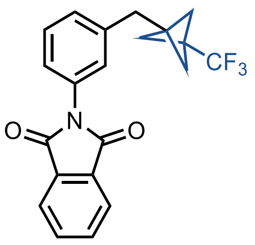

8

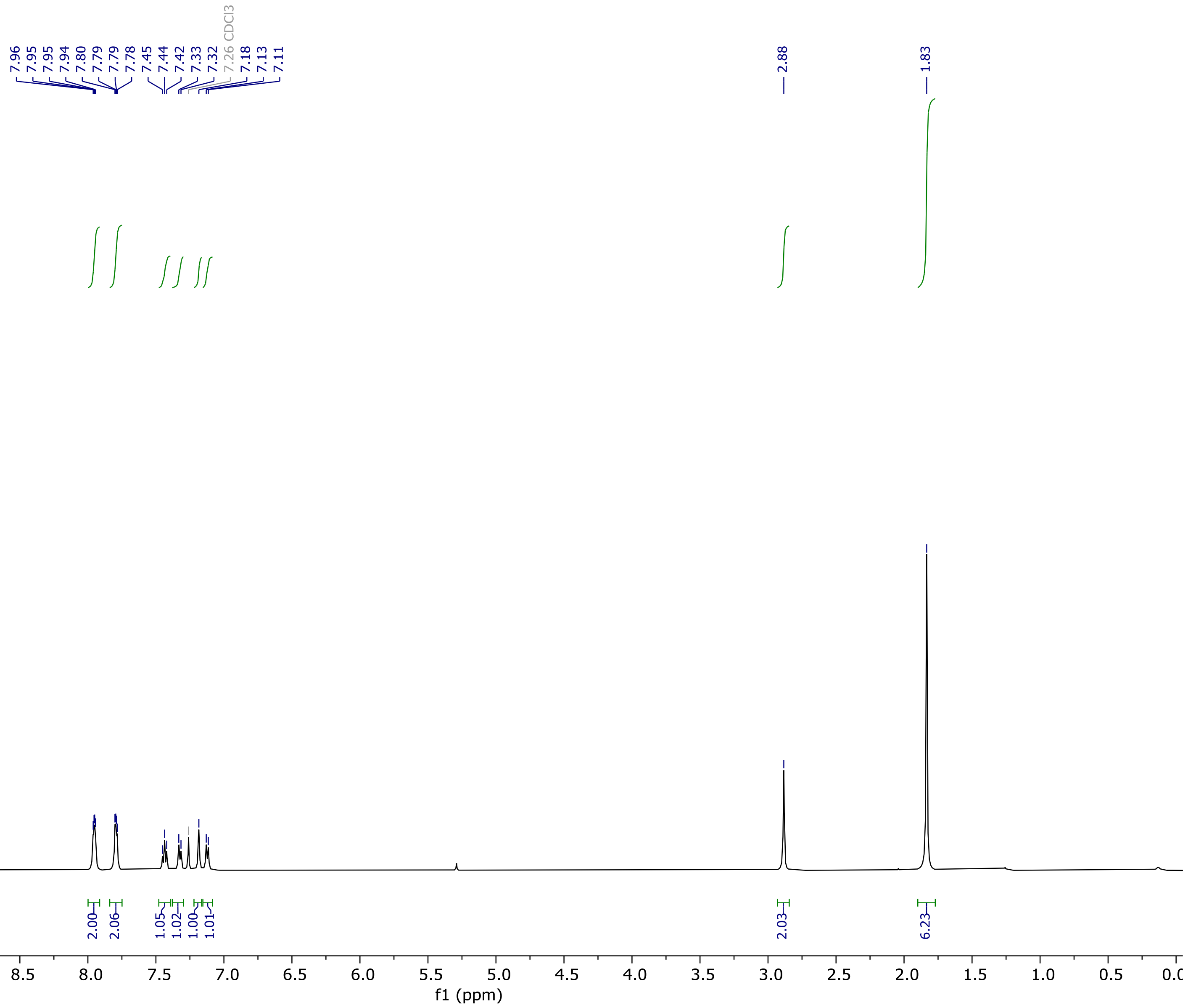

^{13}C NMR of bicyclo[1.1.1]pentylmethylarene 8

$CDCl_3$, 298 K

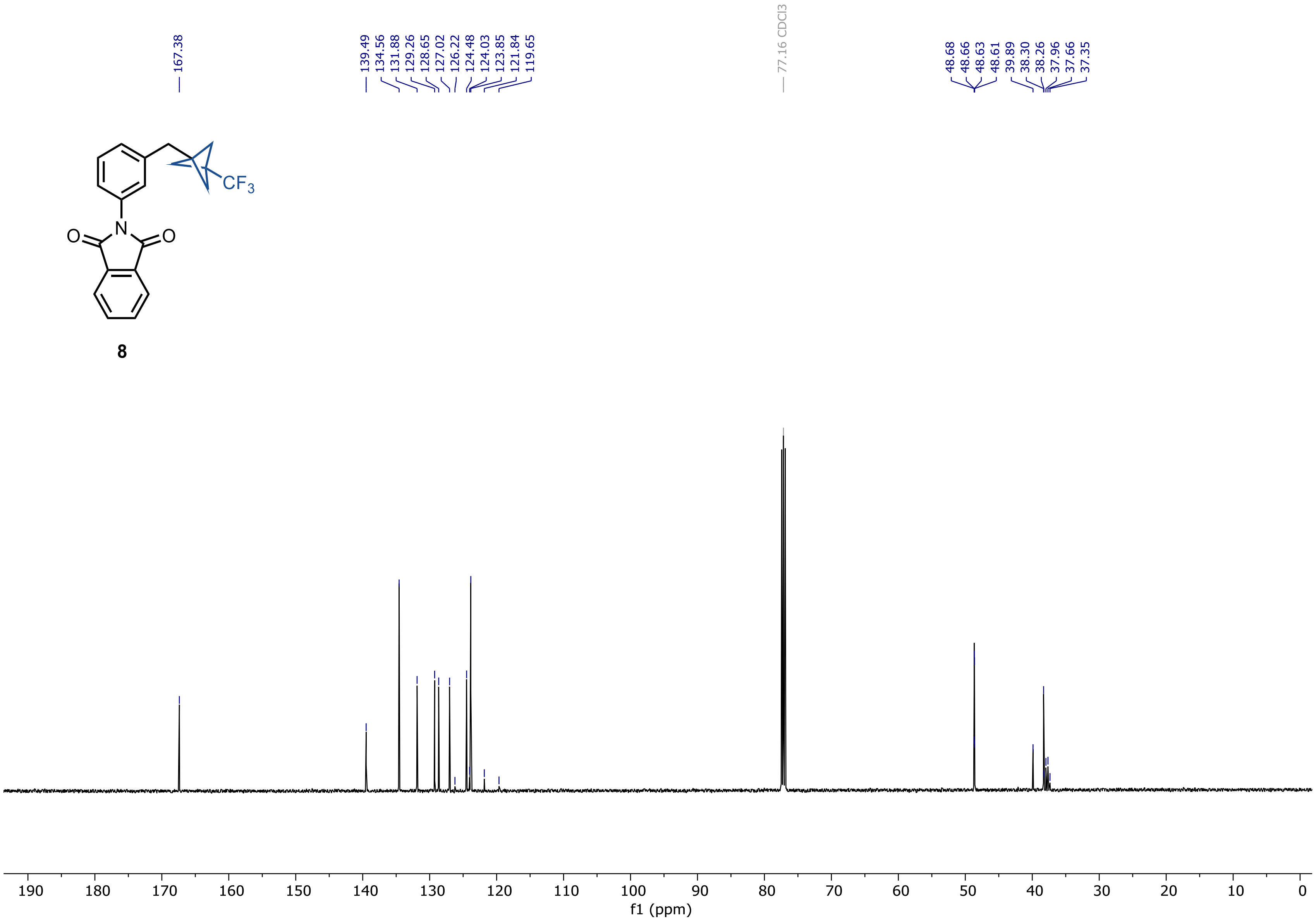

^{19}F NMR of bicyclo[1.1.1]pentylmethylarene 8

CDCl$_3$, 298 K

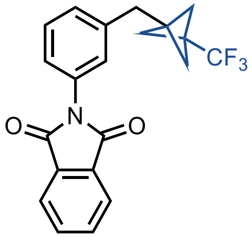

8

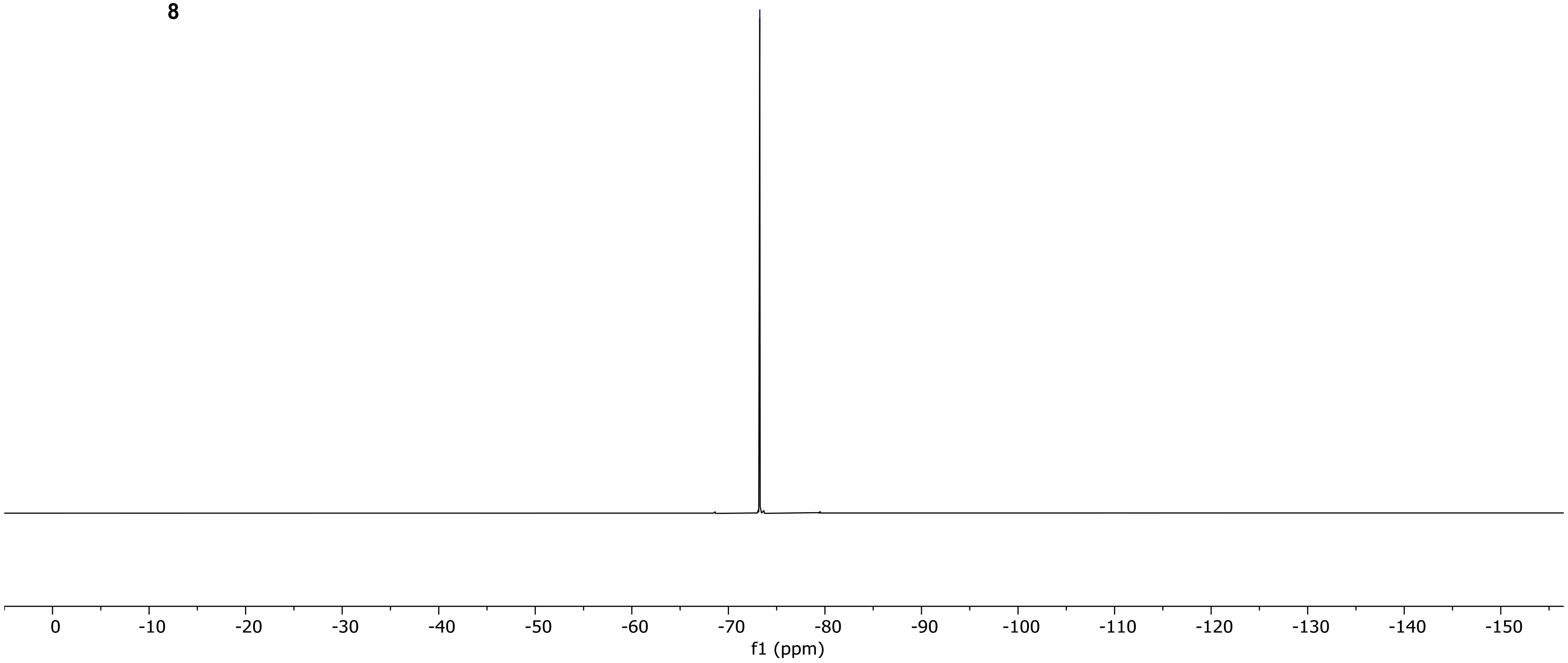

^{1}H NMR of bicyclo[1.1.1]pentylmethylarene 9

CDCl3, 298 K

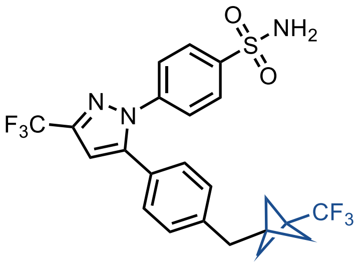

9

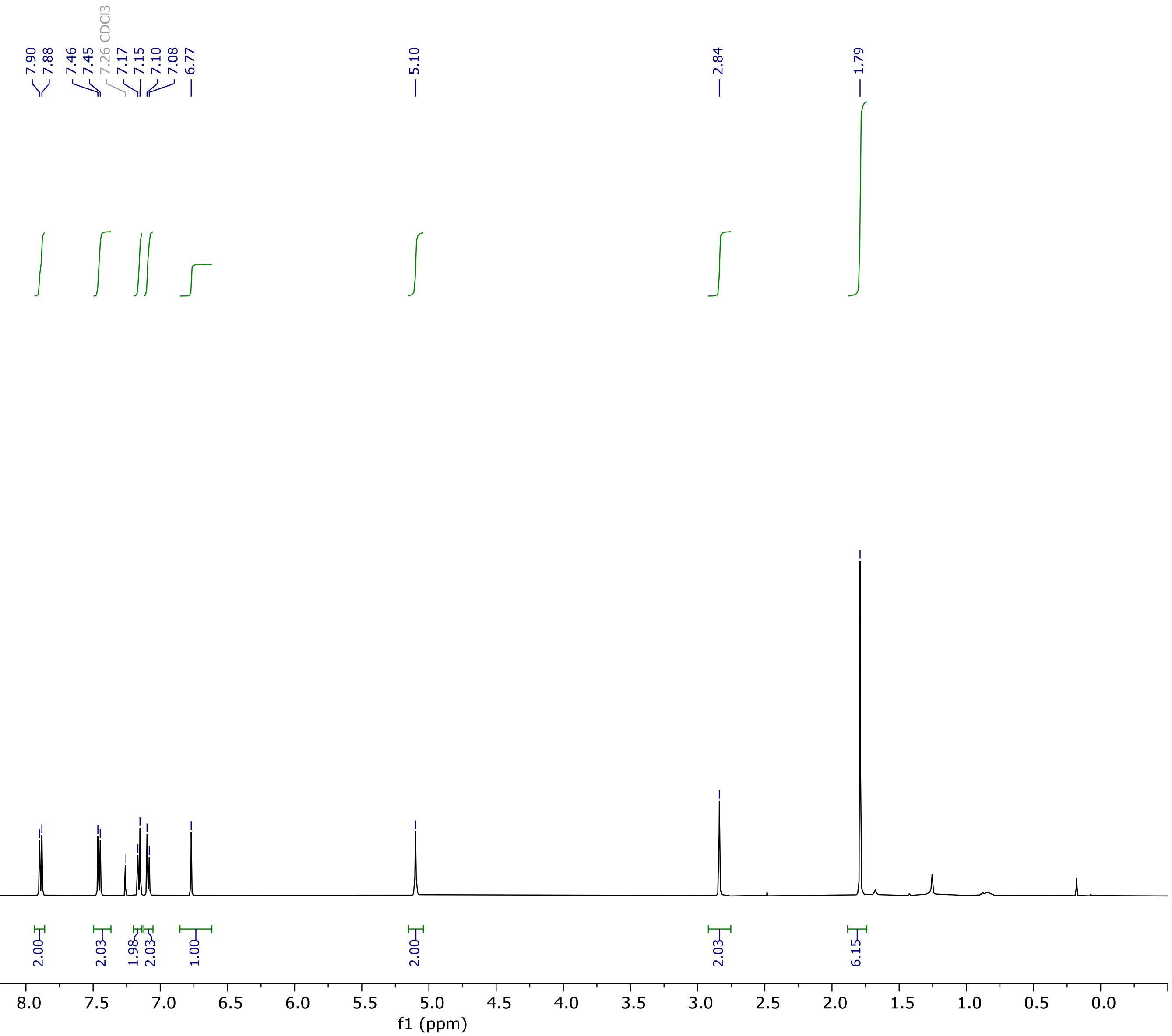

^{13}C NMR of bicyclo[1.1.1]pentylmethylarene 9

$CDCl_3$, 298 K

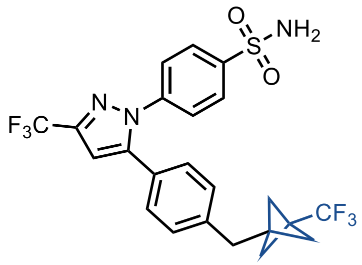

9

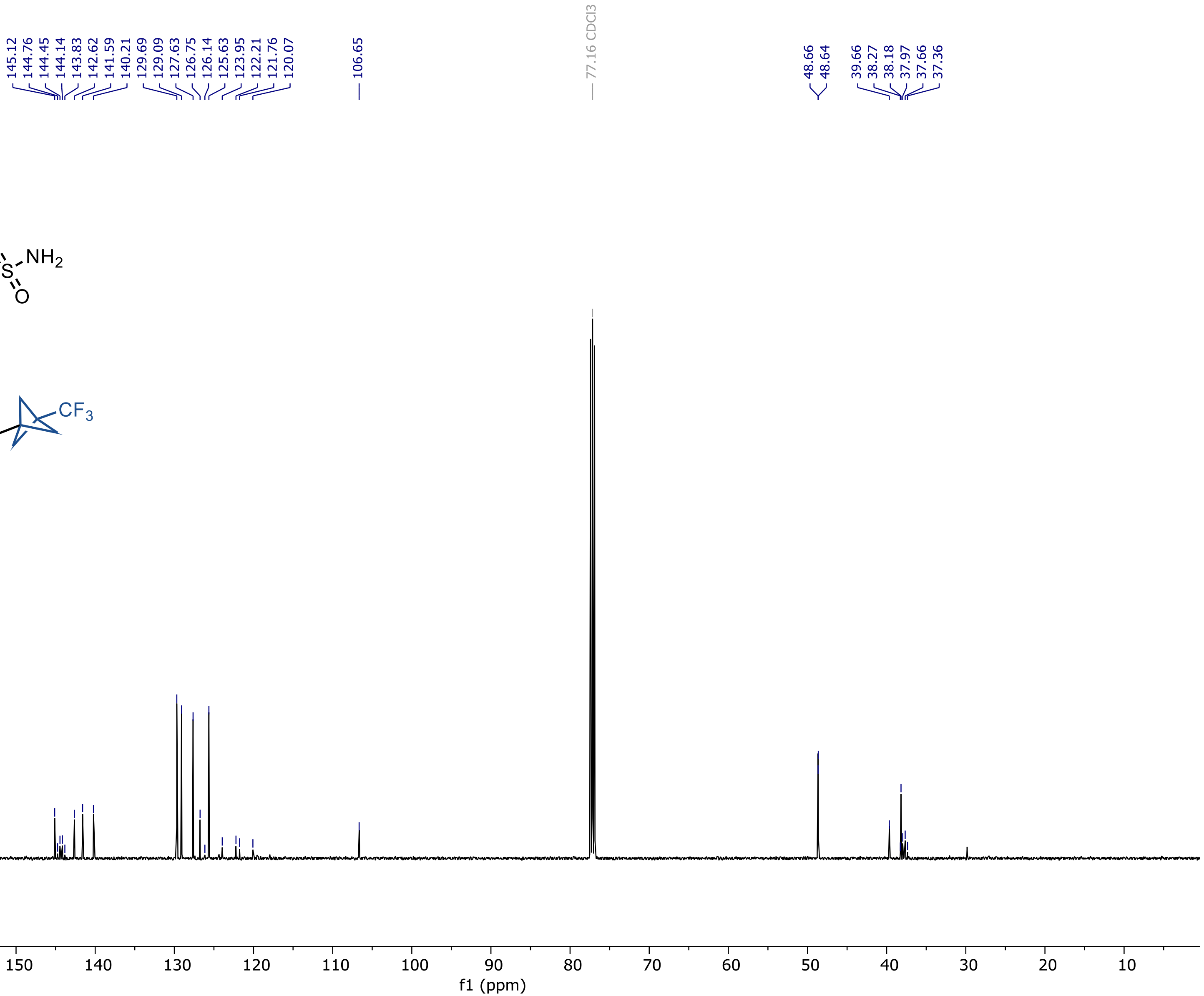

^{19}F NMR of bicyclo[1.1.1]pentylmethylarene 9

$CDCl_3$, 298 K

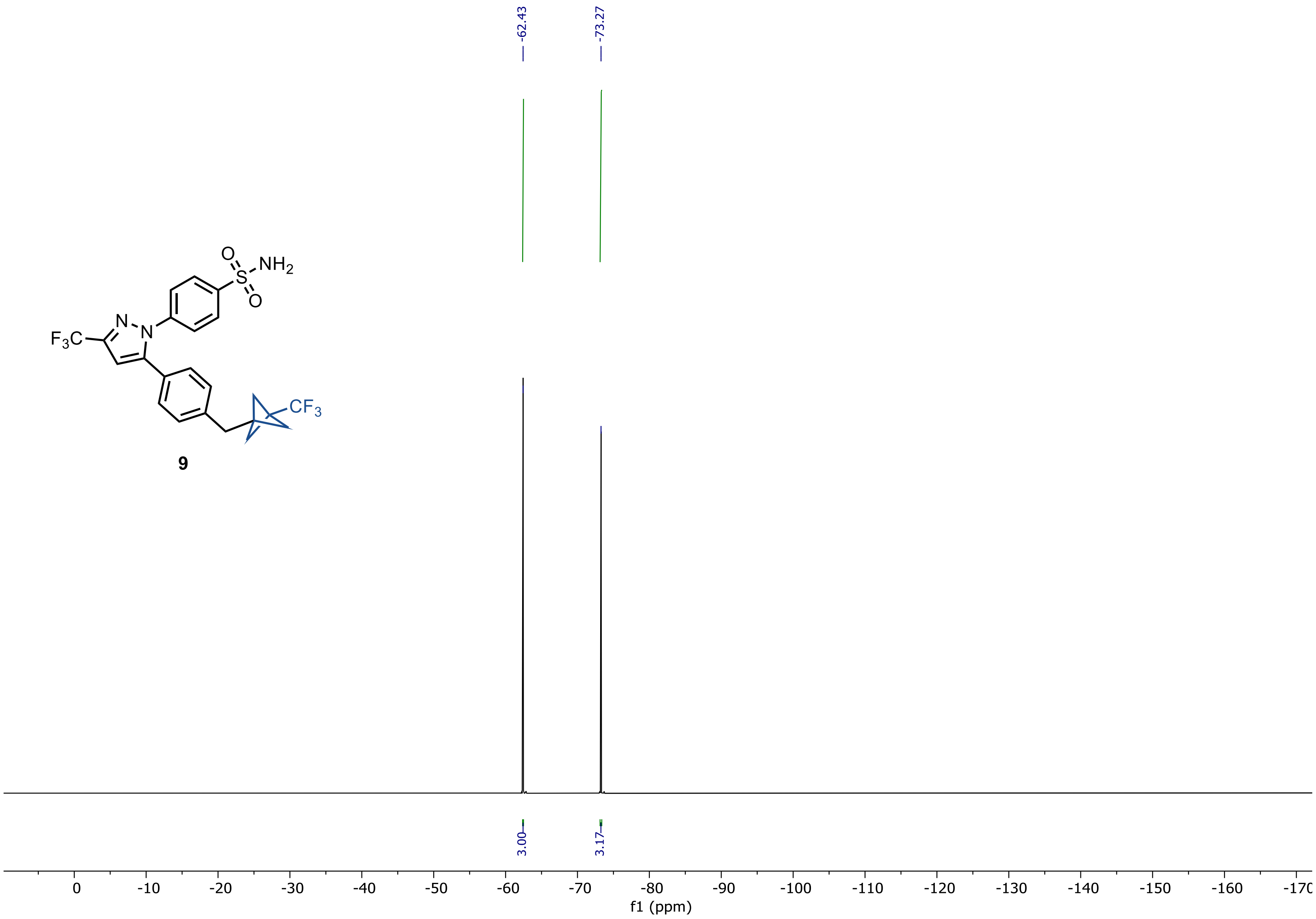

^{1}H NMR of bicyclo[1.1.1]pentylalkane 10

$CDCl_3$, 298 K

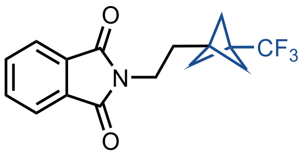

10

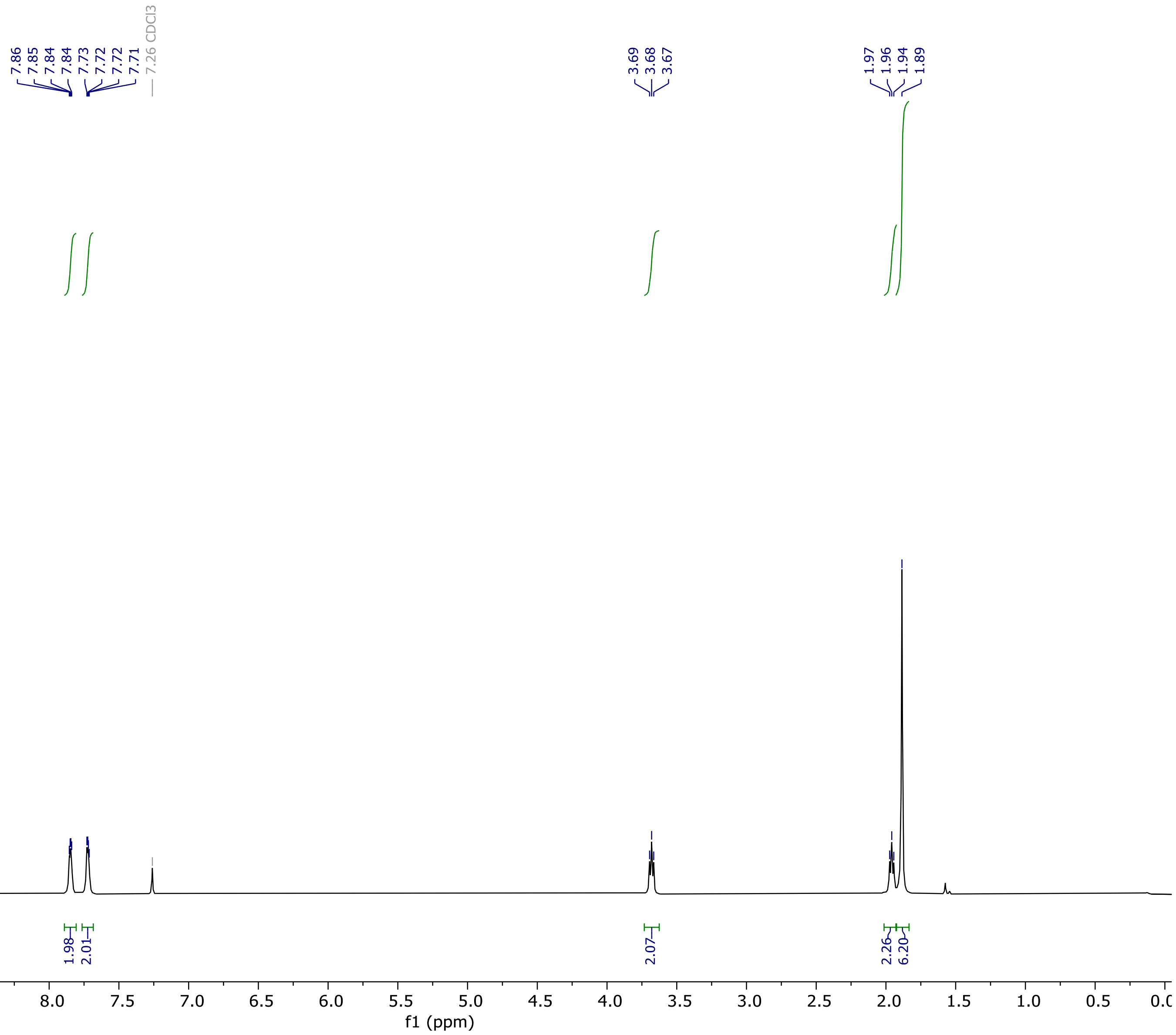

^{13}C NMR of bicyclo[1.1.1]pentylalkane 10

$CDCl_3$, 298 K

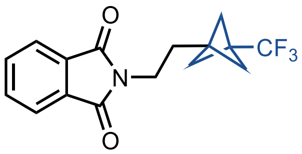

10

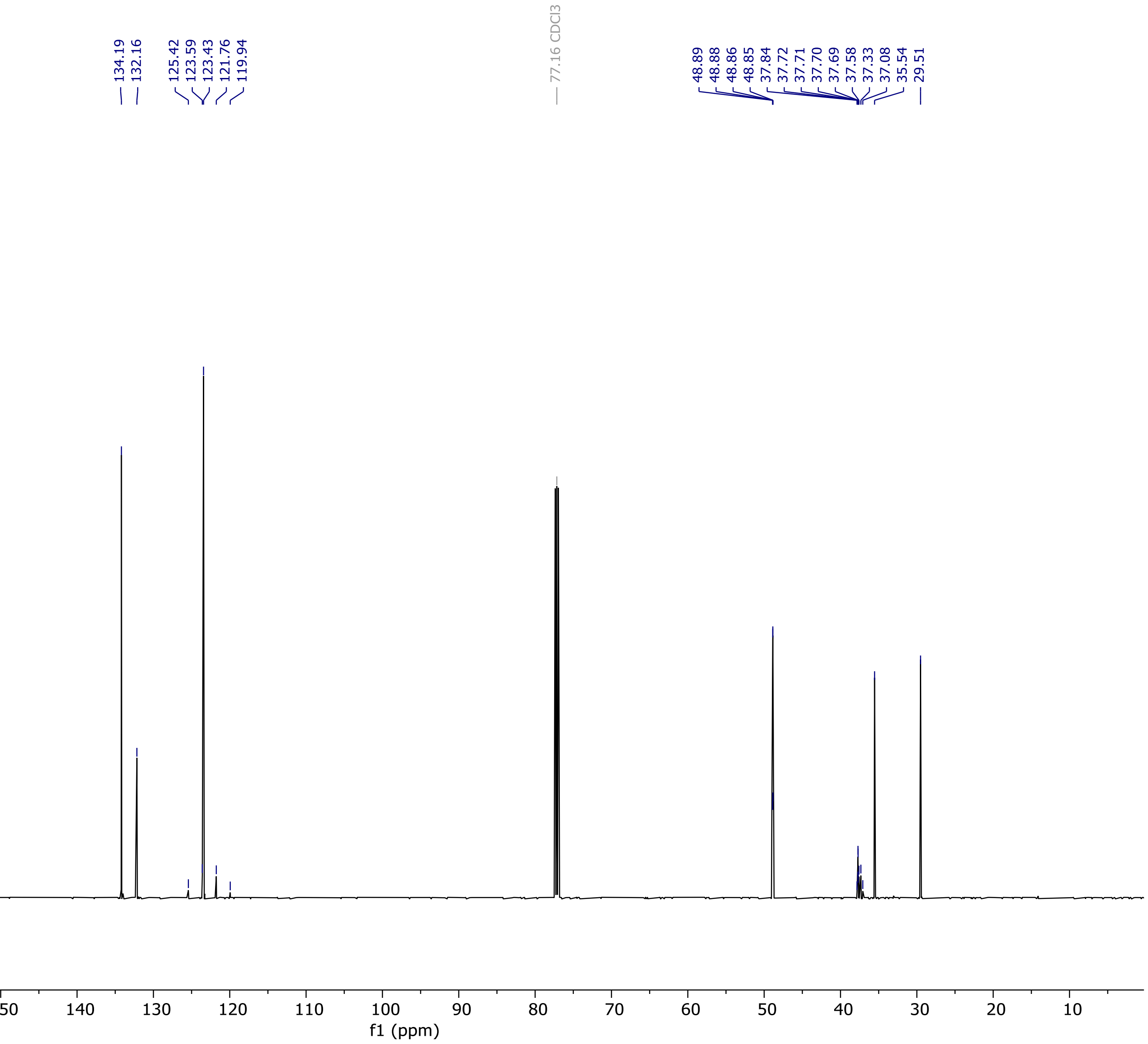

^{19}F NMR of bicyclo[1.1.1]pentylalkane 10

CDCl$_3$, 298 K

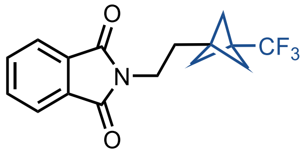

10

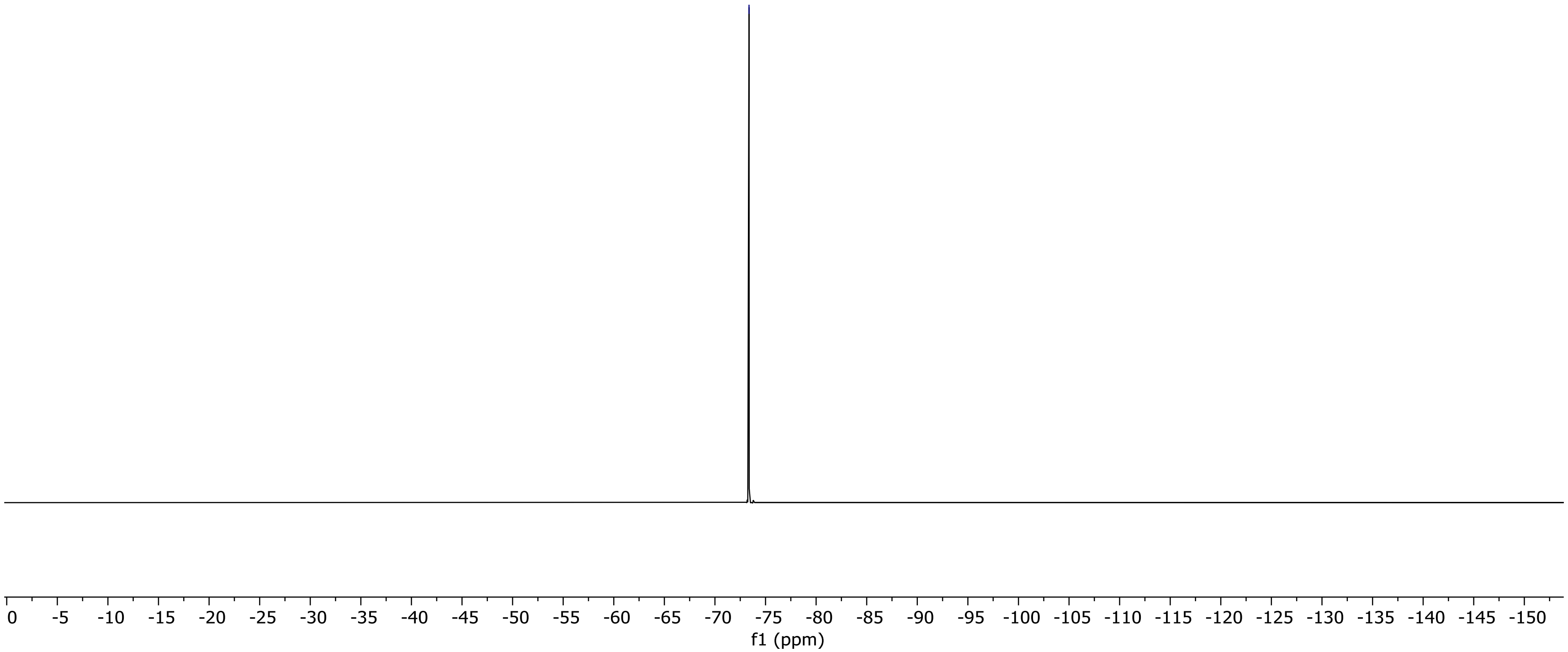

^{1}H NMR of bicyclo[1.1.1]pentylalkane 11

CDCl$_3$, 298 K

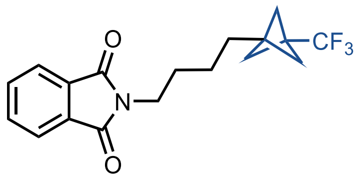

11

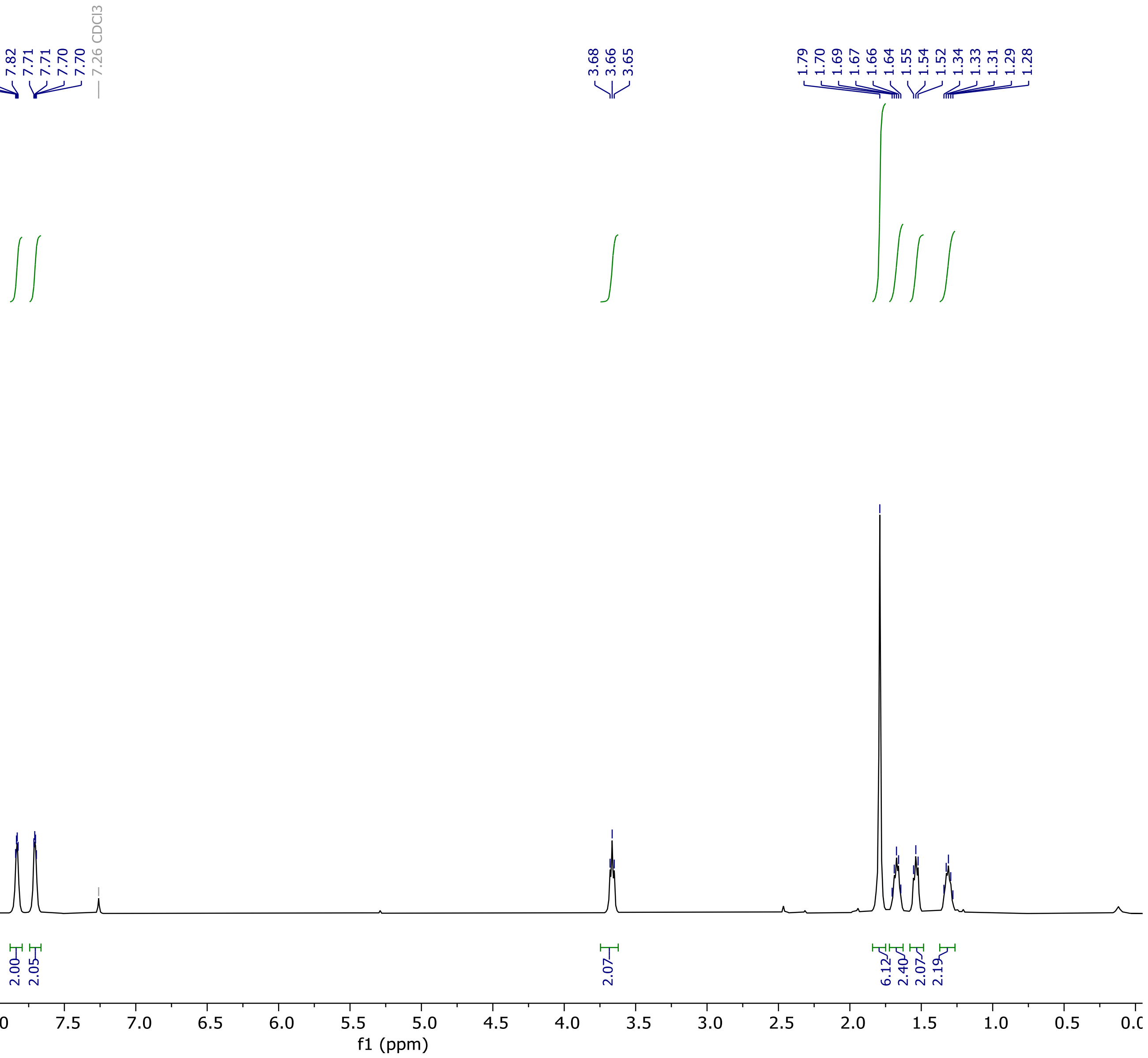

^{13}C NMR of bicyclo[1.1.1]pentylalkane 11

$CDCl_3$, 298 K

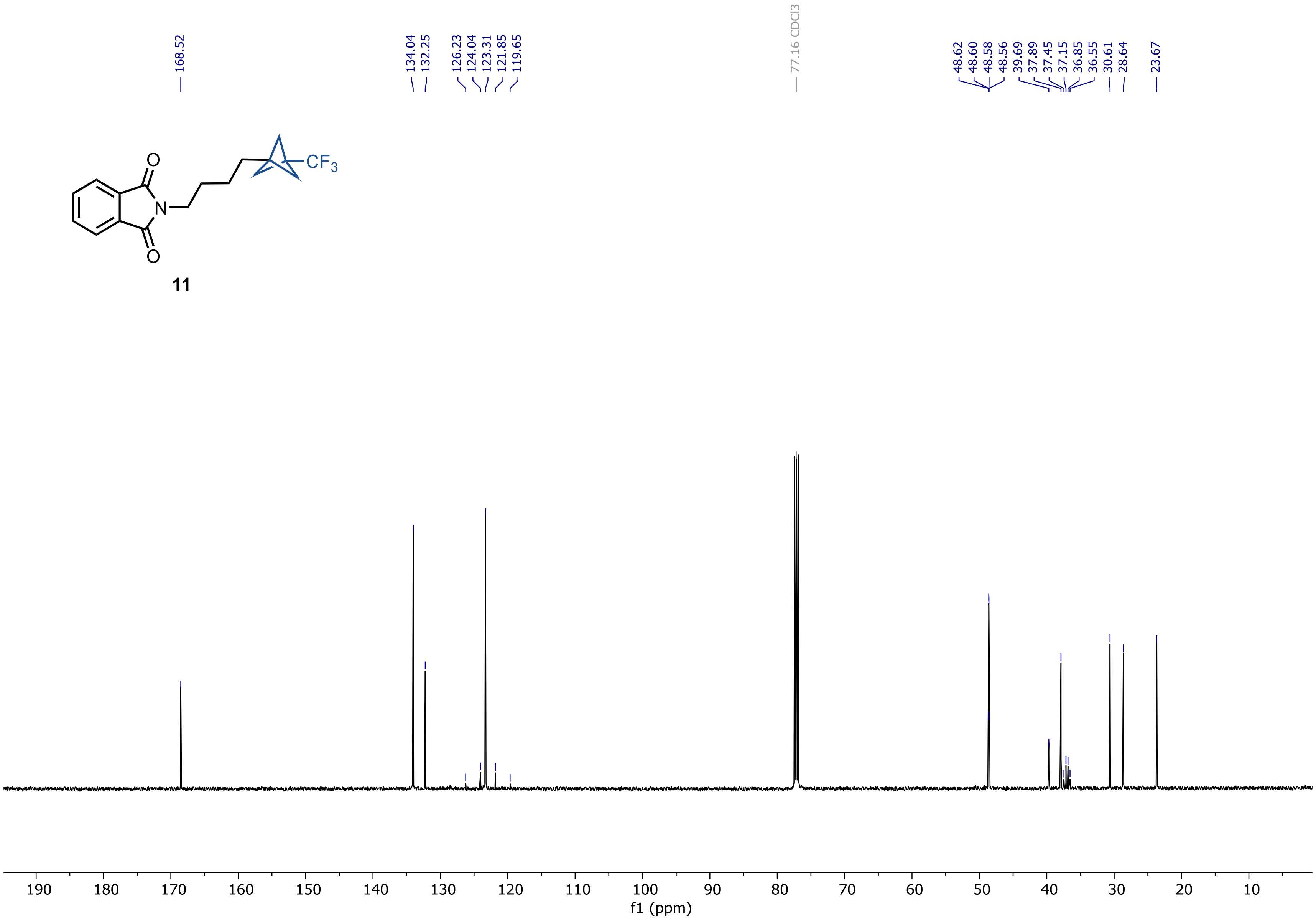

^{19}F NMR of bicyclo[1.1.1]pentylalkane 11

$CDCl_3$, 298 K

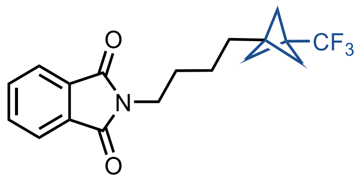

11

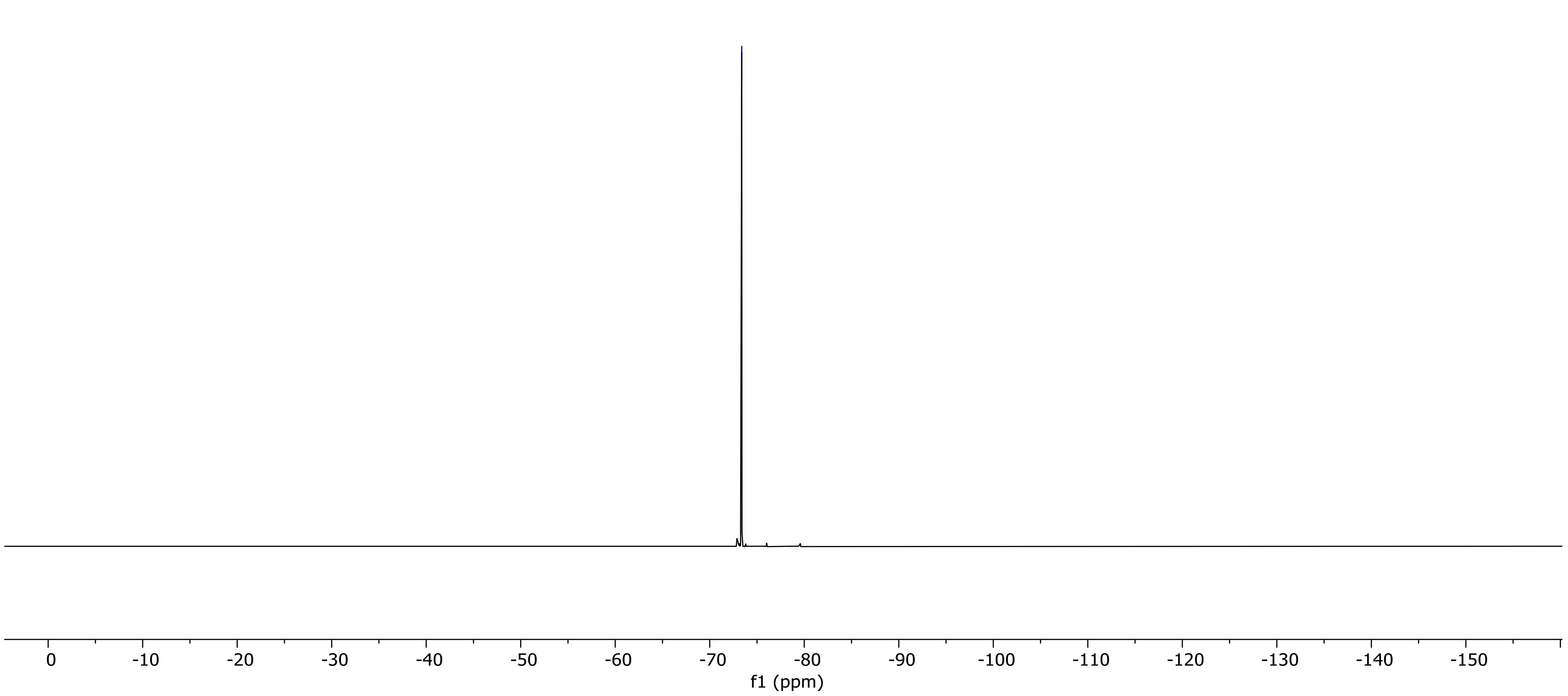

^{1}H NMR of bicyclo[1.1.1]pentylalkane 12

$CDCl_3$, 298 K

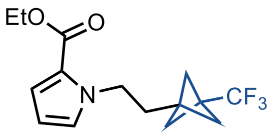

12

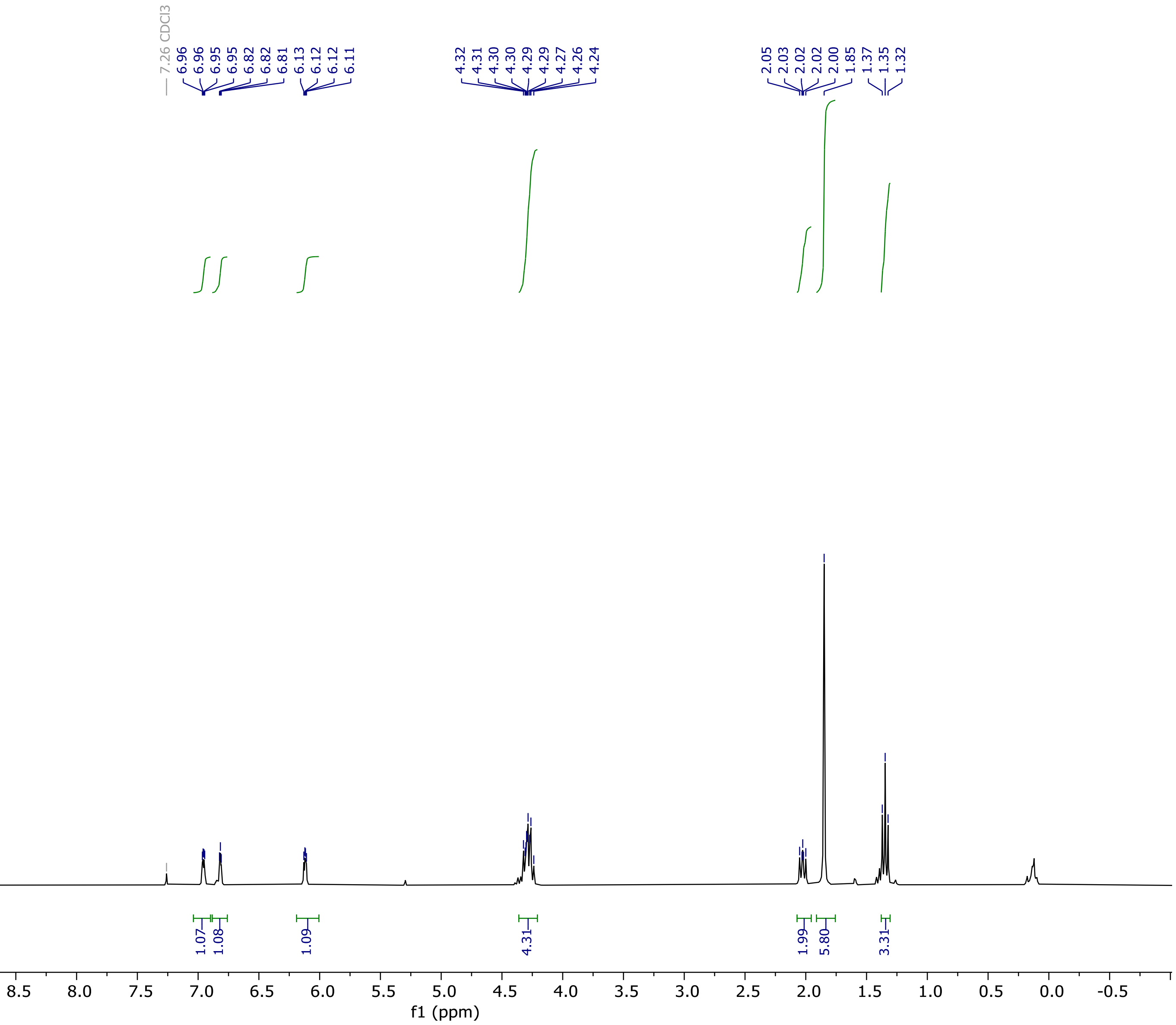

^{13}C NMR of bicyclo[1.1.1]pentylalkane 12

$CDCl_3$, 298 K

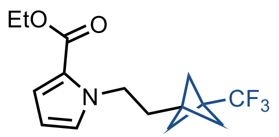

12

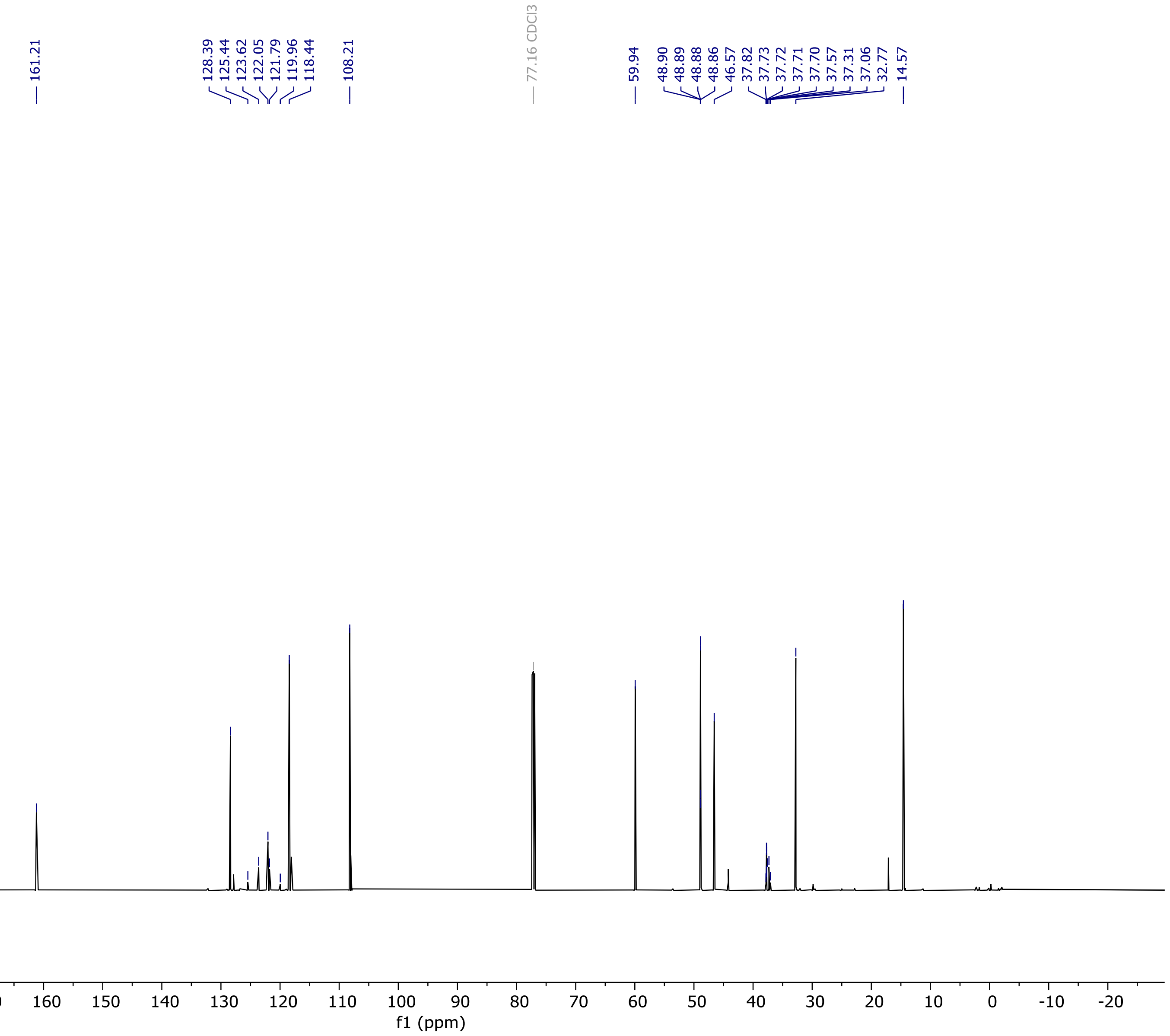

^{19}F NMR of bicyclo[1.1.1]pentylalkane 12

$CDCl_3$, 298 K

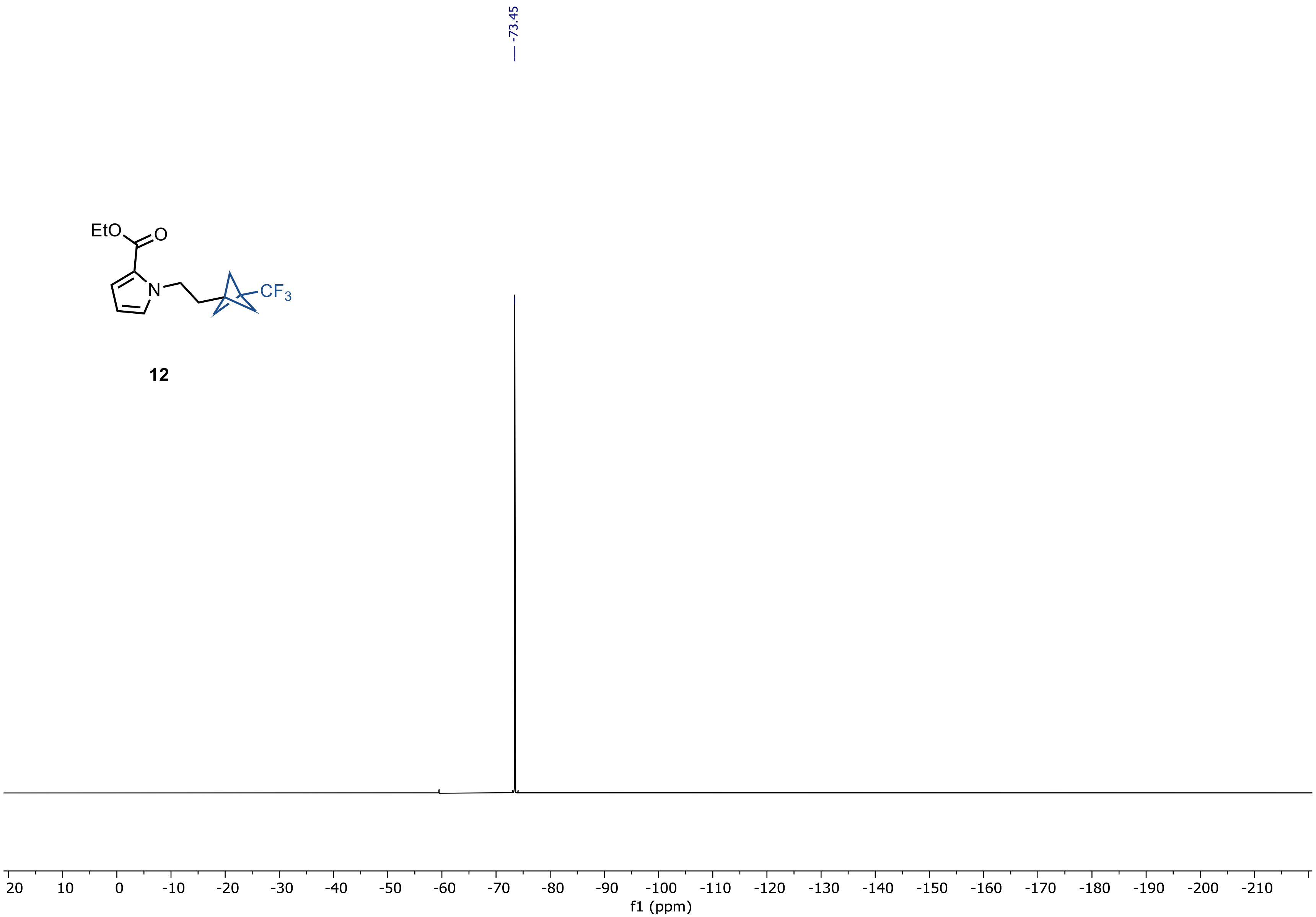

^{1}H NMR of bicyclo[1.1.1]pentylalkane 13

CDCl$_3$, 298 K

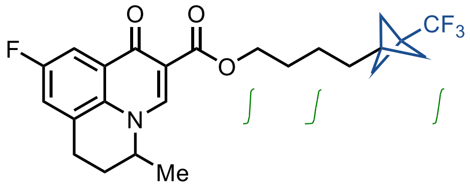

13

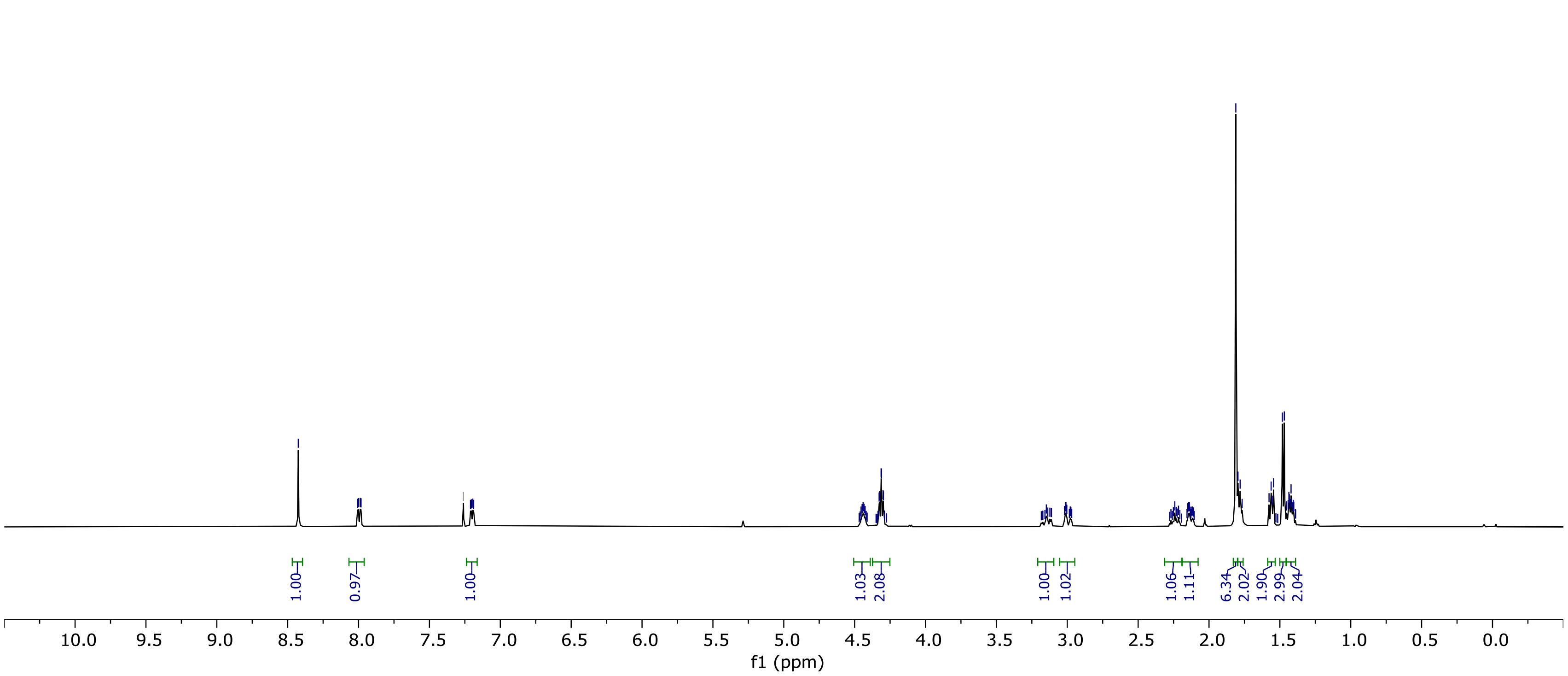

^{13}C NMR of bicyclo[1.1.1]pentylalkane 13

$CDCl_3$, 298 K

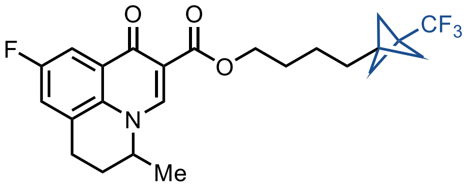

13

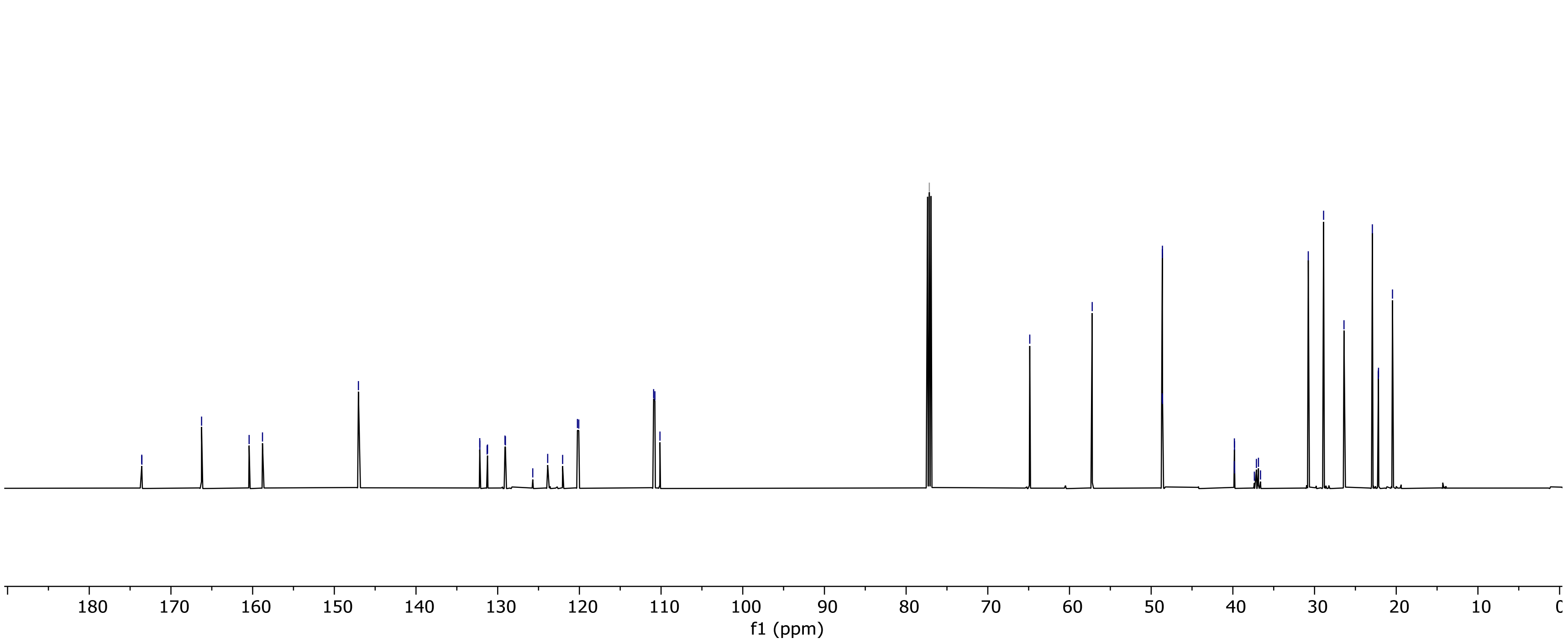

^{19}F NMR of bicyclo[1.1.1]pentylalkane 13

CDCl$_3$, 298 K

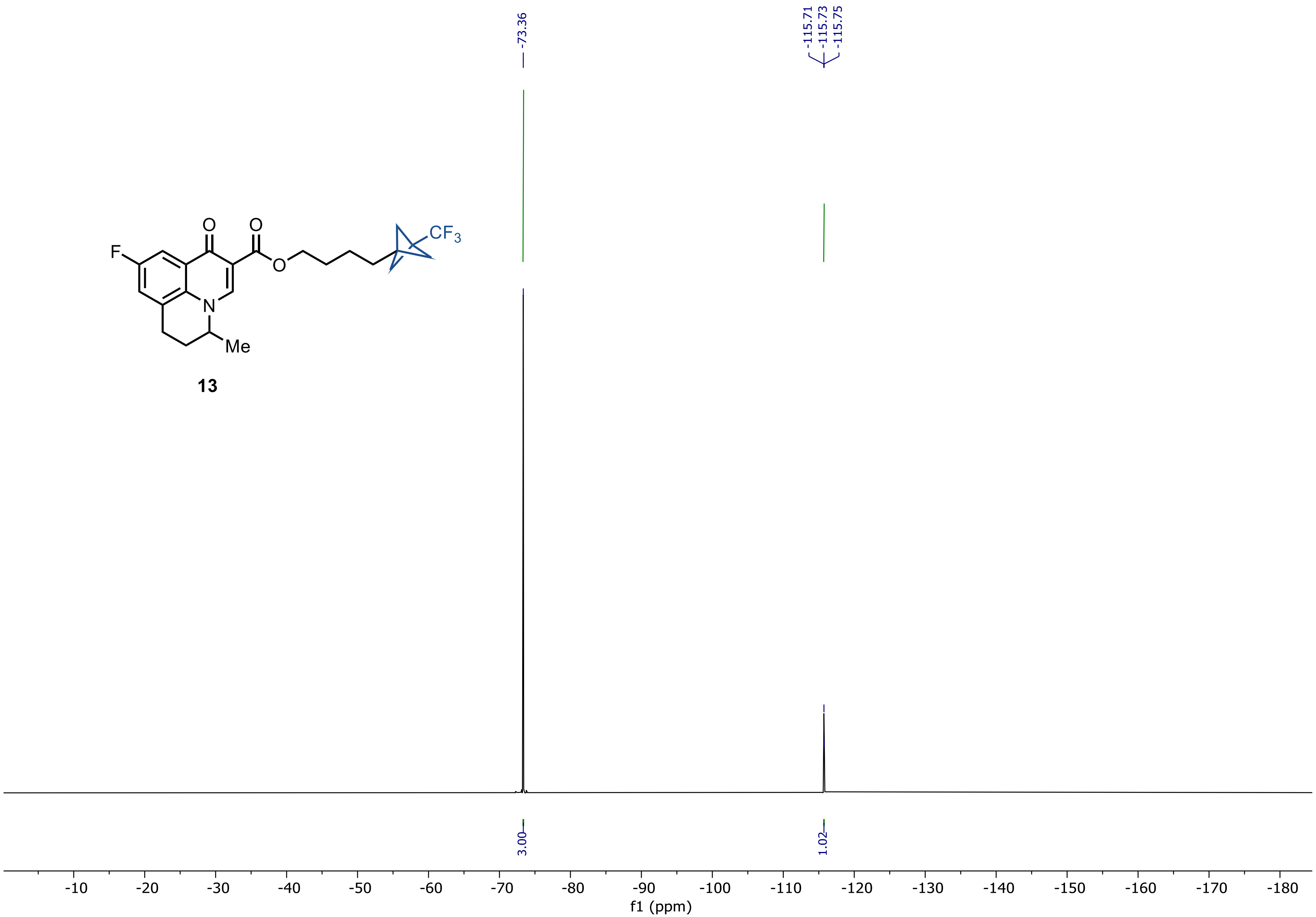

^{1}H NMR of bicyclo[1.1.1]pentylalkane 14

CDCl3, 298 K

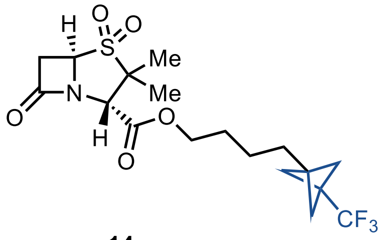

14

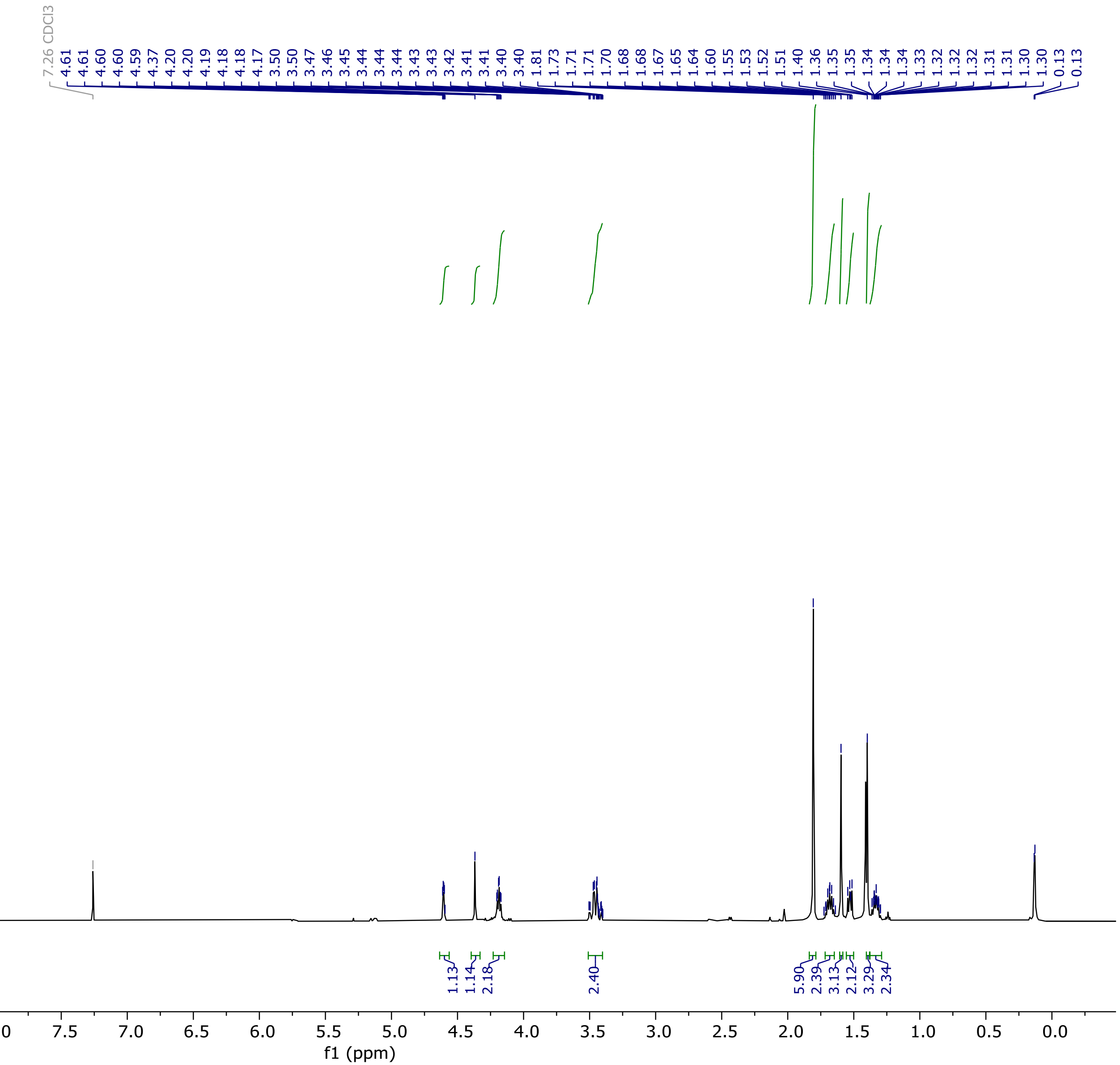

^{13}C NMR of bicyclo[1.1.1]pentylalkane 14

CDCl$_3$, 298 K

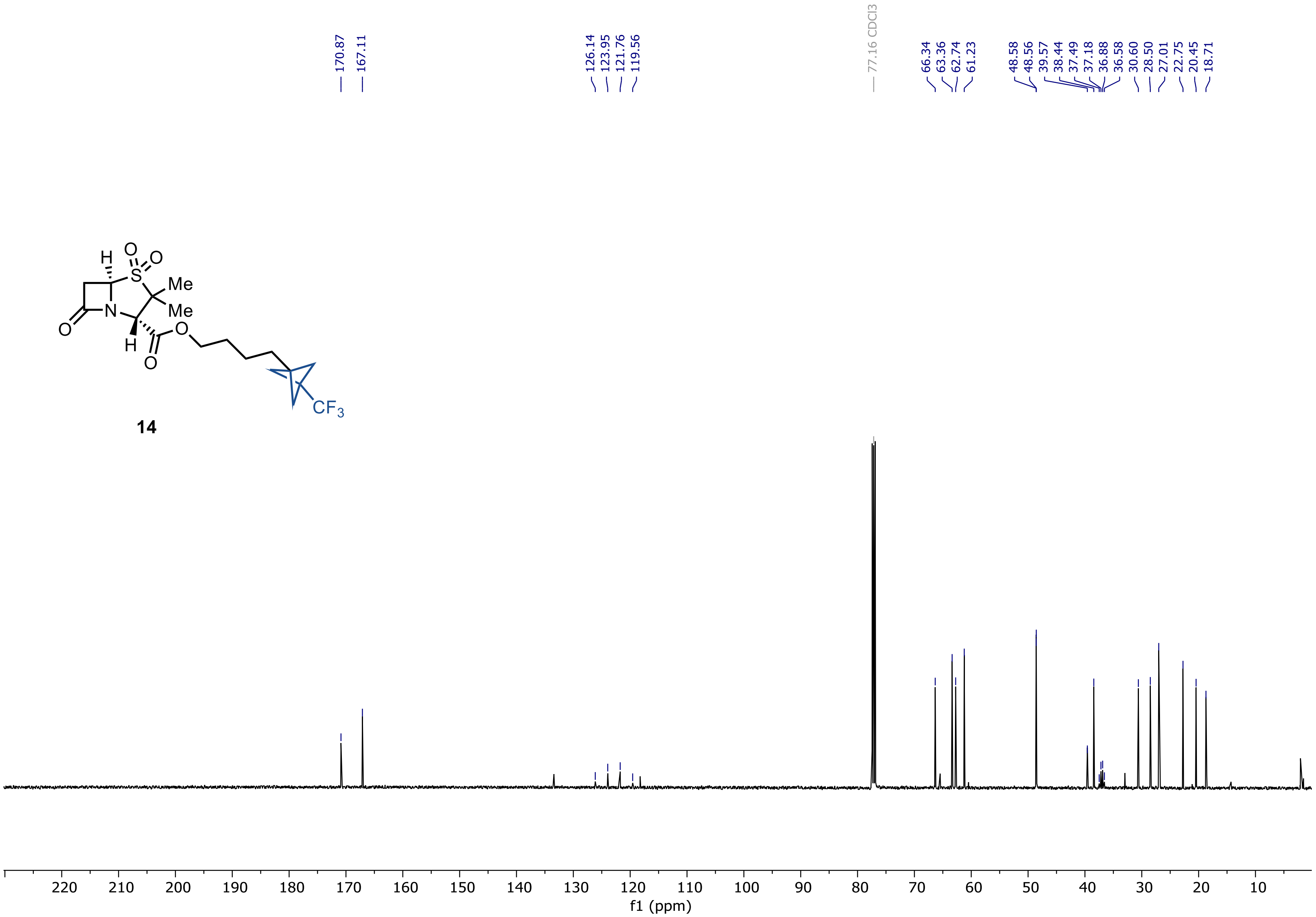

^{19}F NMR of bicyclo[1.1.1]pentylalkane 14

$CDCl_3$, 298 K

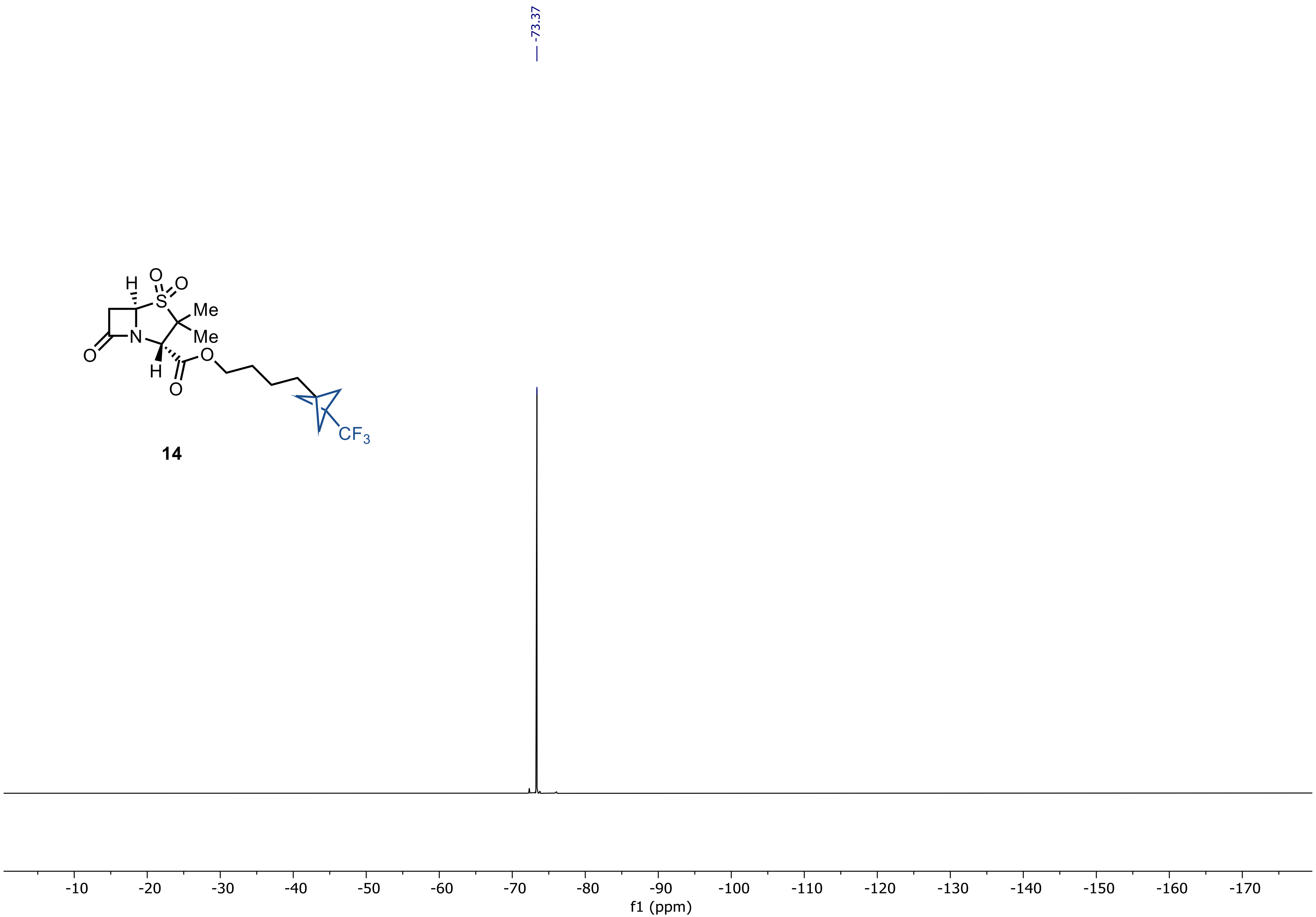

^{1}H NMR of bicyclo[1.1.1]pentylalkane 15

$CDCl_3$, 298 K

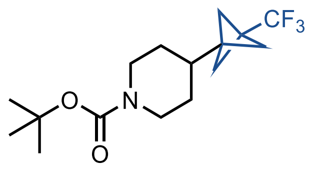

15

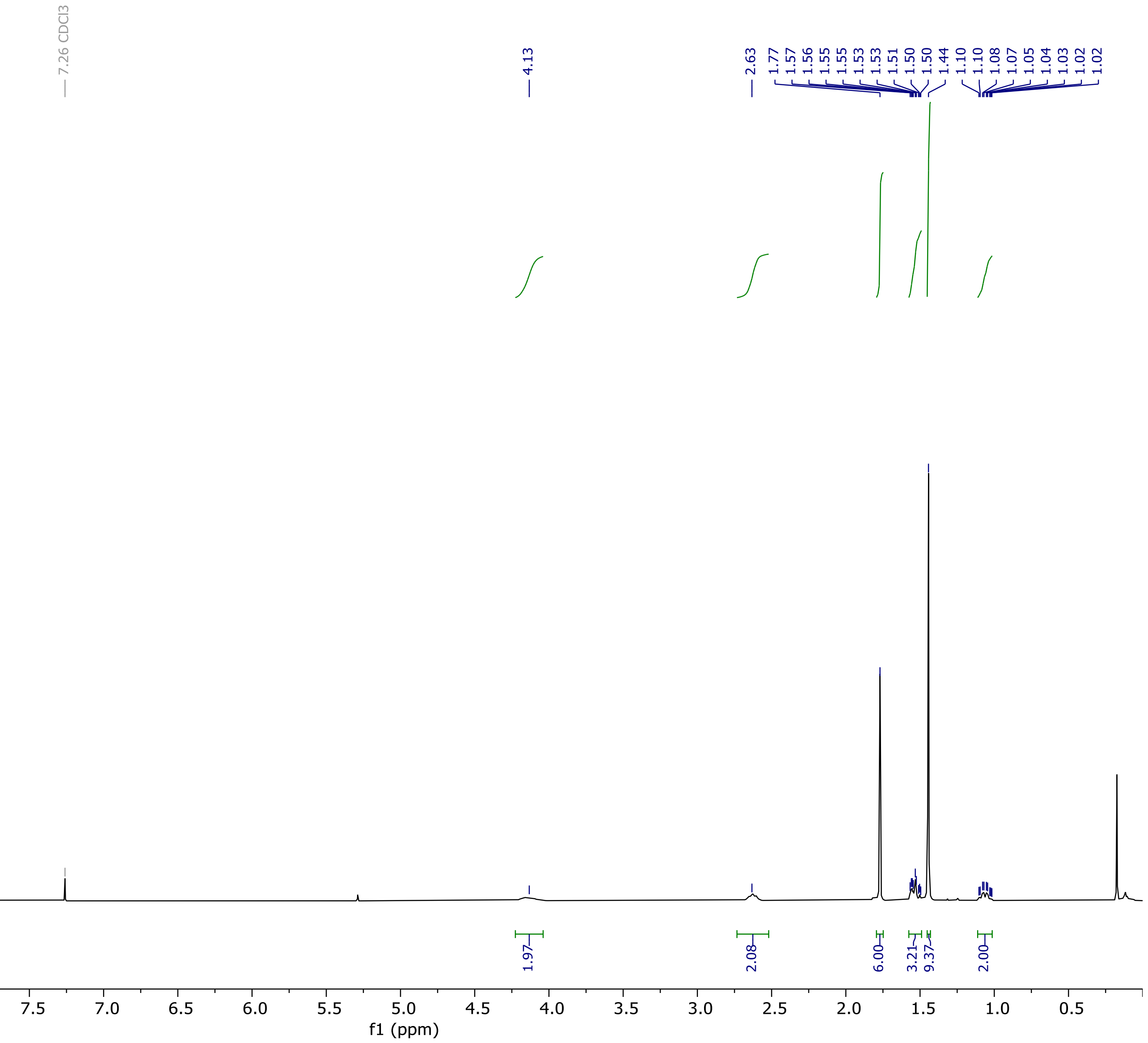

^{13}C NMR of bicyclo[1.1.1]pentylalkane 15

$CDCl_3$, 298 K

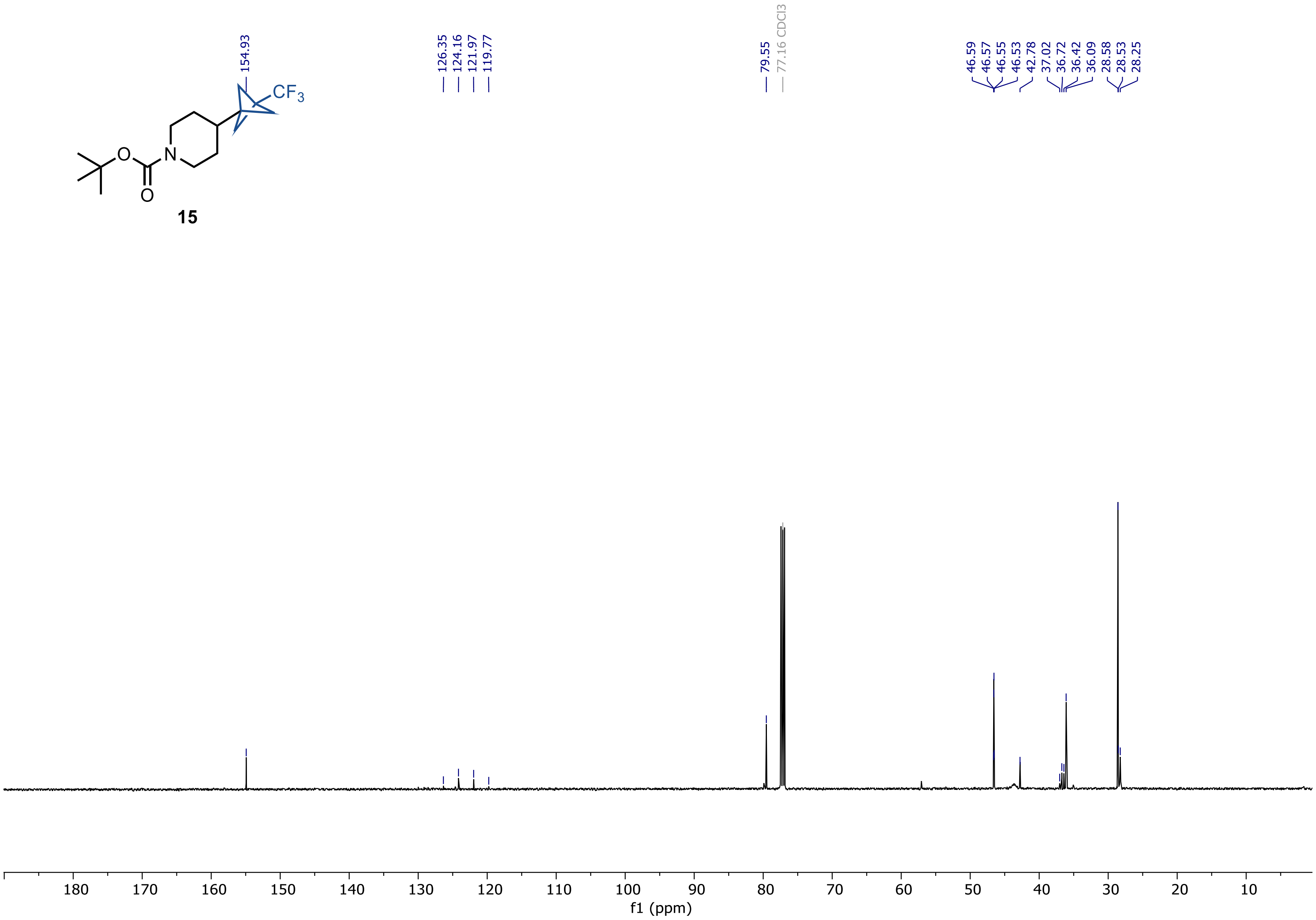

^{19}F NMR of bicyclo[1.1.1]pentylalkane 15

CDCl$_3$, 298 K

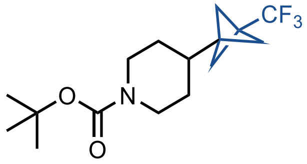

15

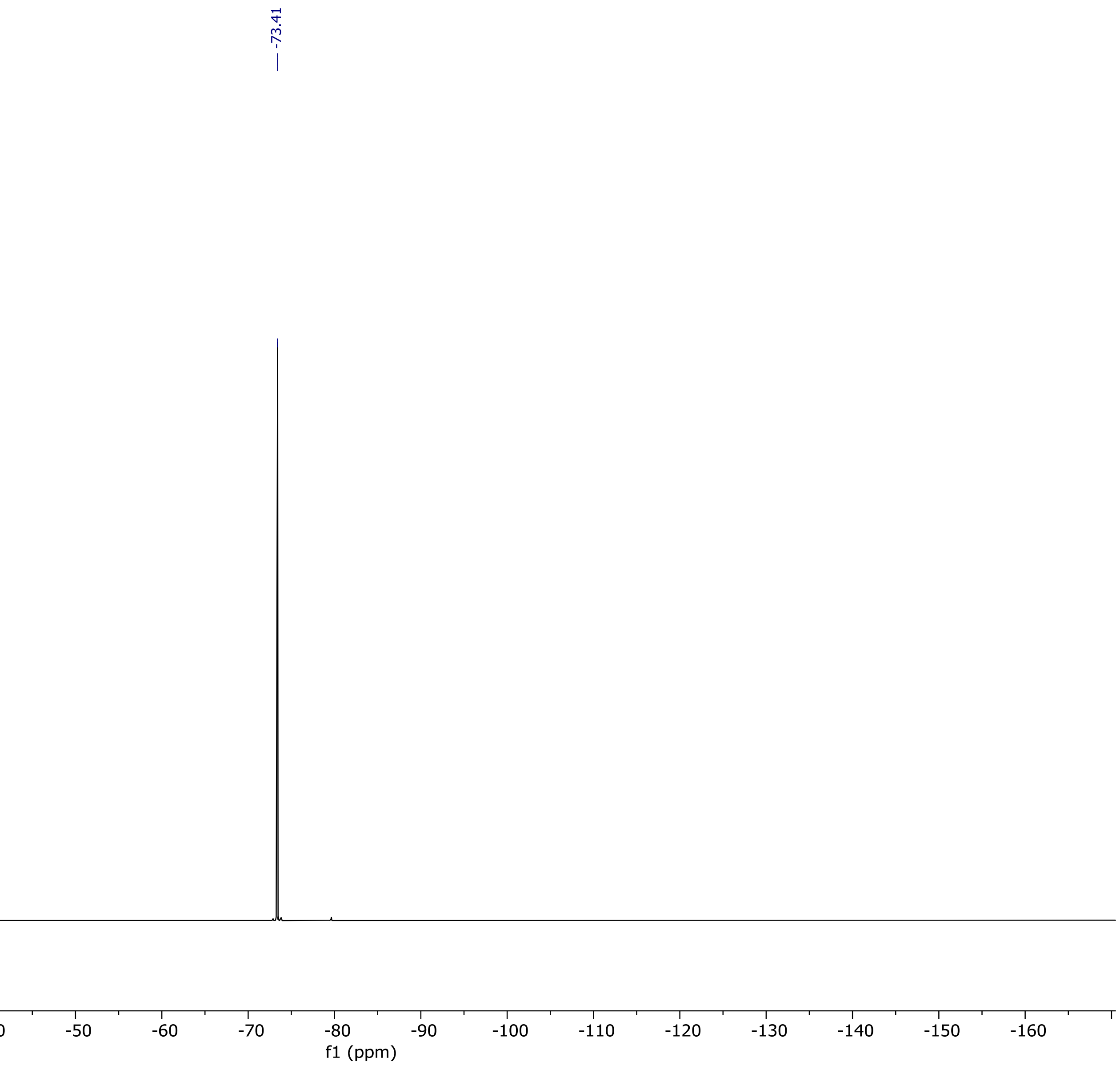

^{1}H NMR of bicyclo[1.1.1]pentylalkane 16

CDCl3, 298 K

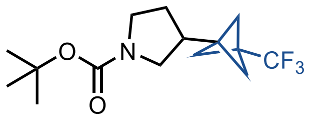

16

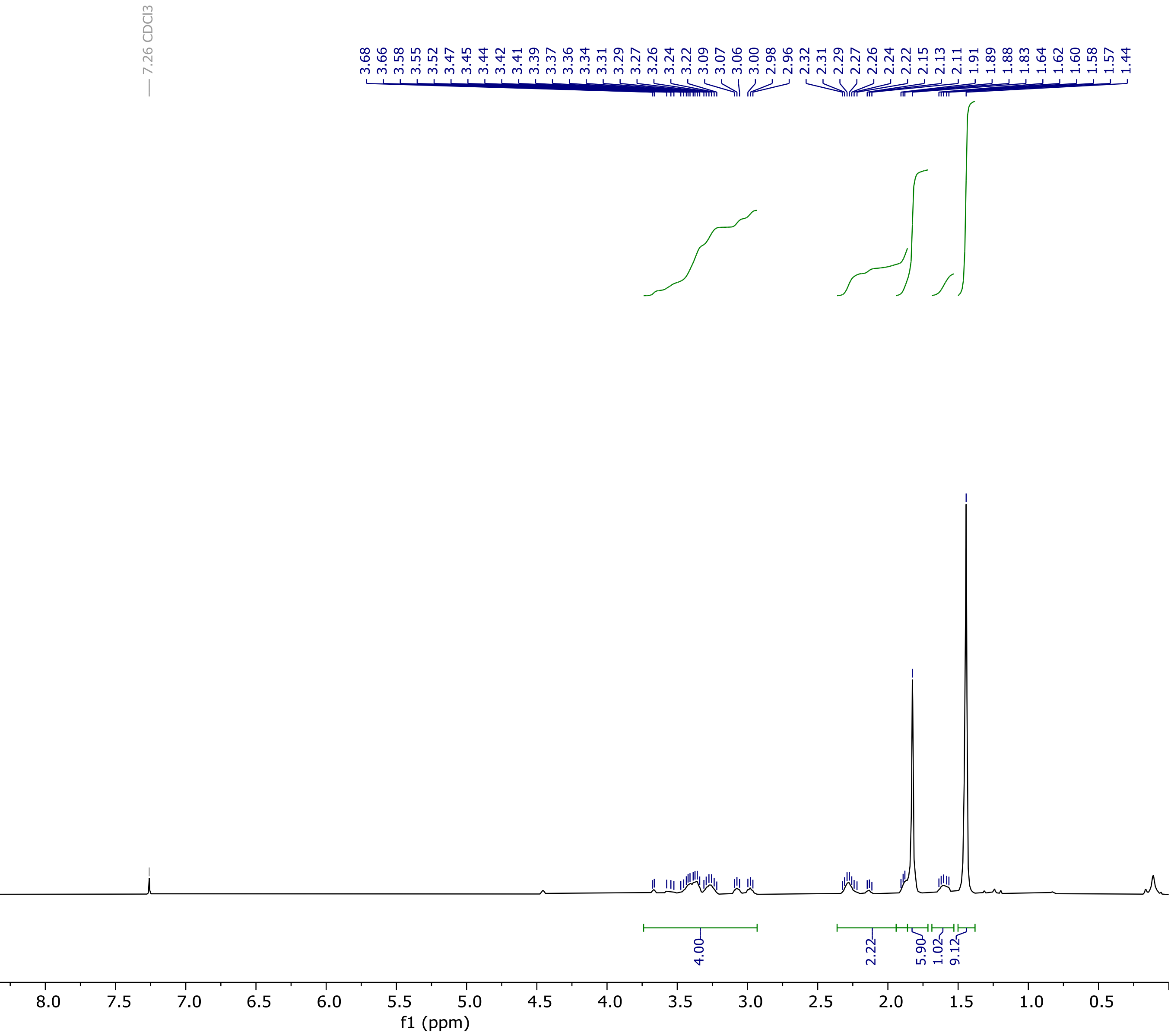

^{13}C NMR of bicyclo[1.1.1]pentylalkane 16

$CDCl_3$, 298 K

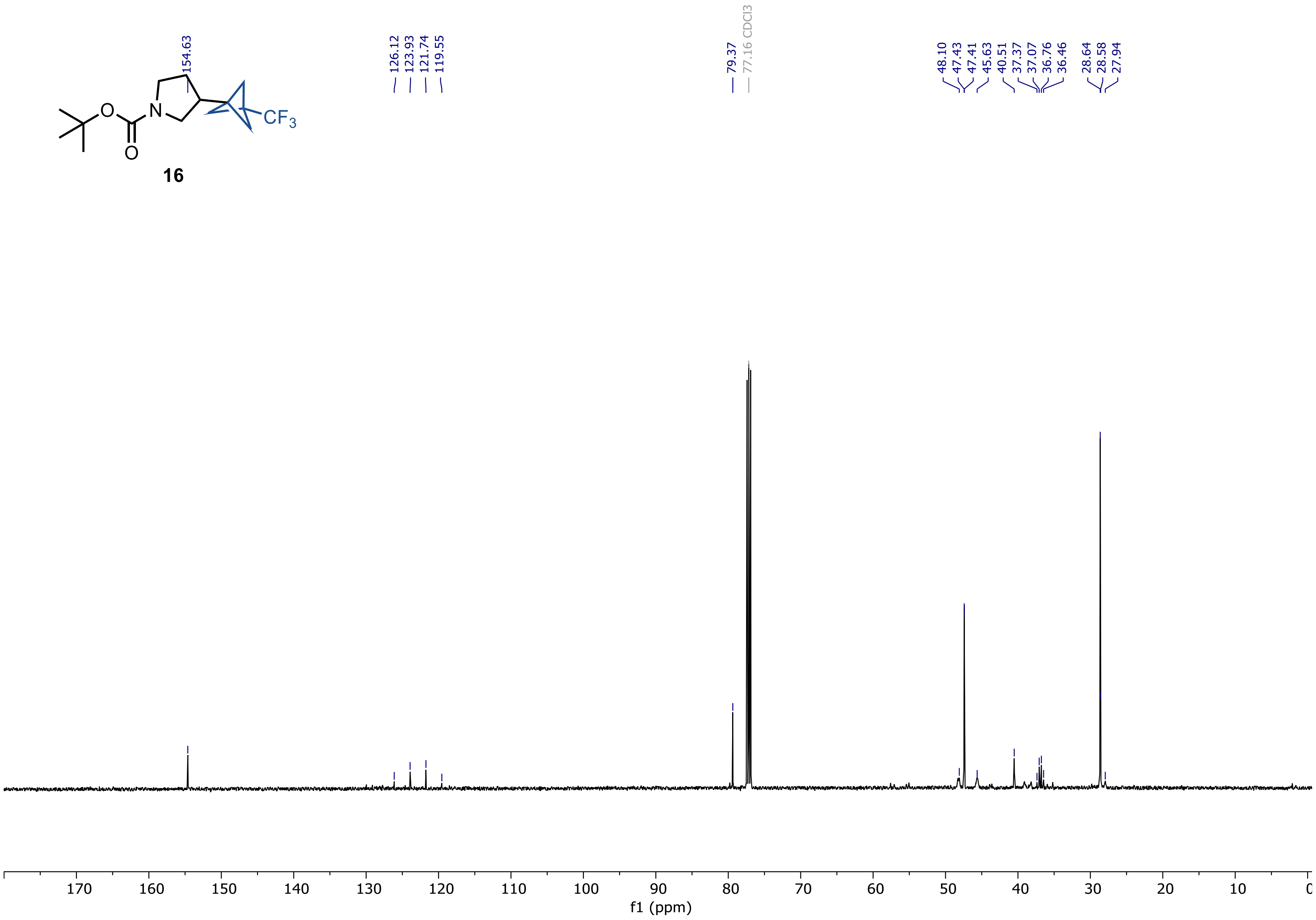

^{19}F NMR of bicyclo[1.1.1]pentylalkane 16

$CDCl_3$, 298 K

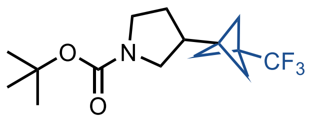

16

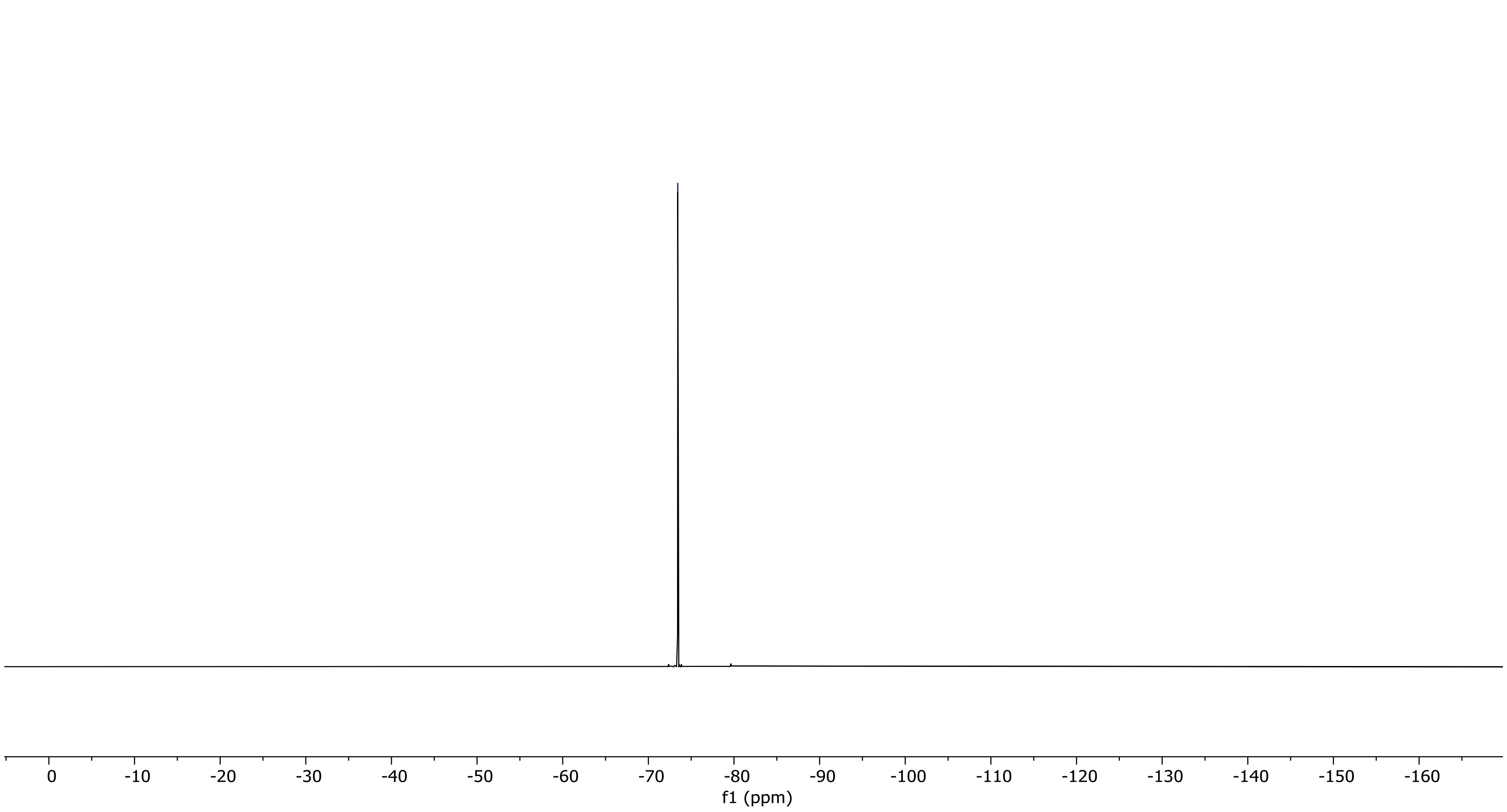

^{1}H NMR of bicyclo[1.1.1]pentylalkane 17

CDCl$_3$, 298 K

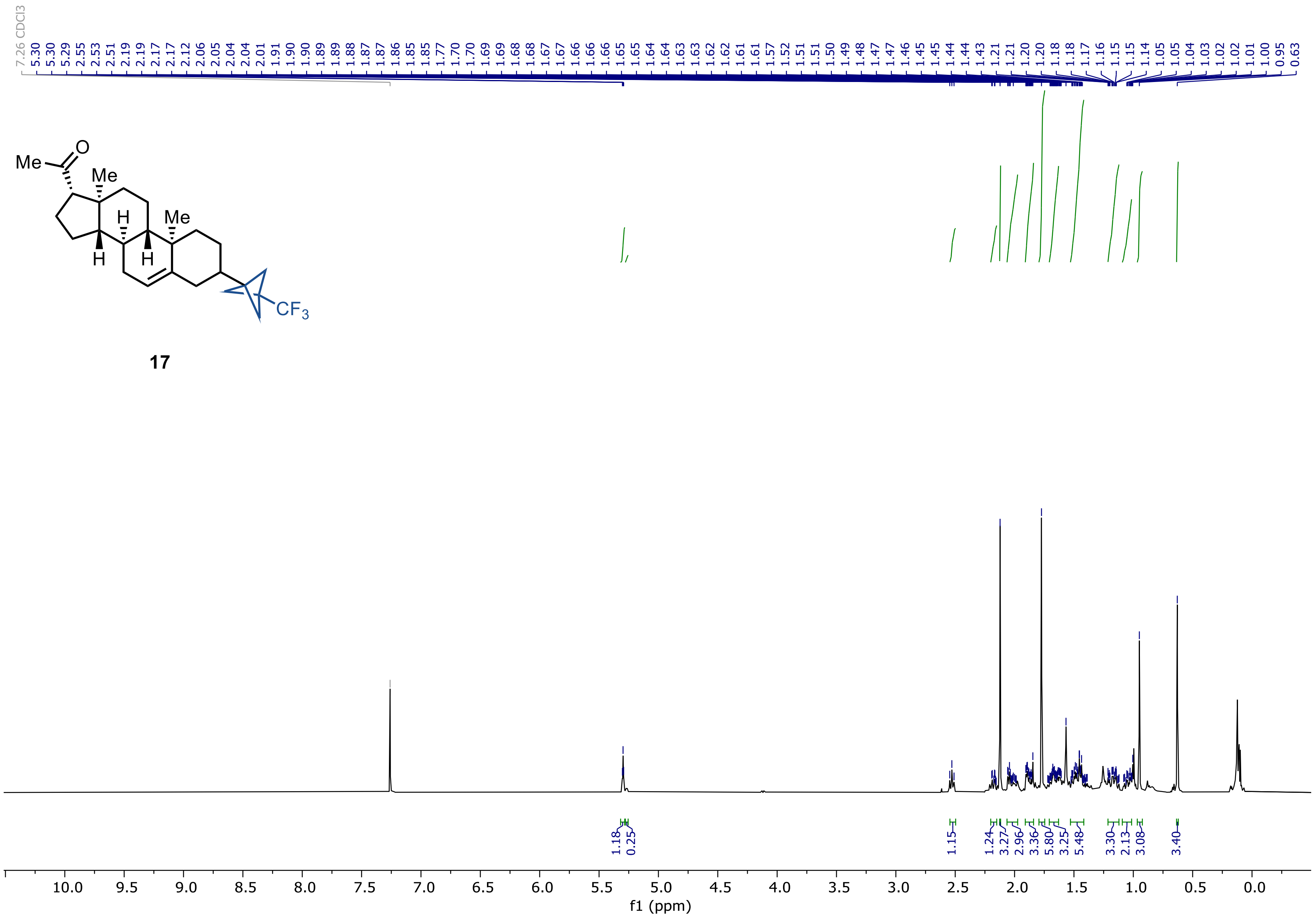

17

^{13}C NMR of bicyclo[1.1.1]pentylalkane 17

$CDCl_3$, 298 K

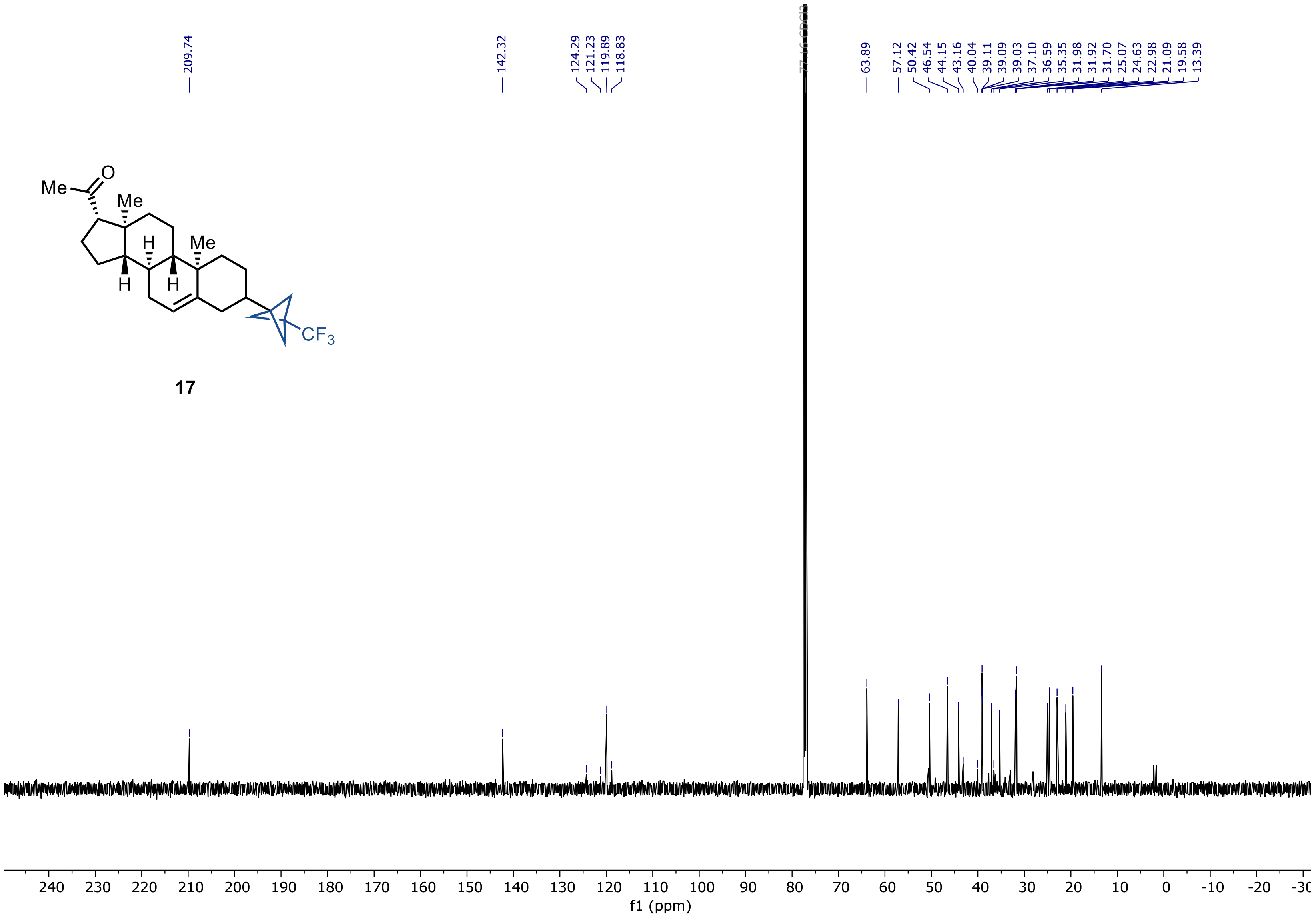

17

^{19}F NMR of bicyclo[1.1.1]pentylalkane 17

$CDCl_3$, 298 K

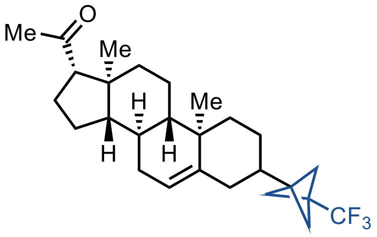

17

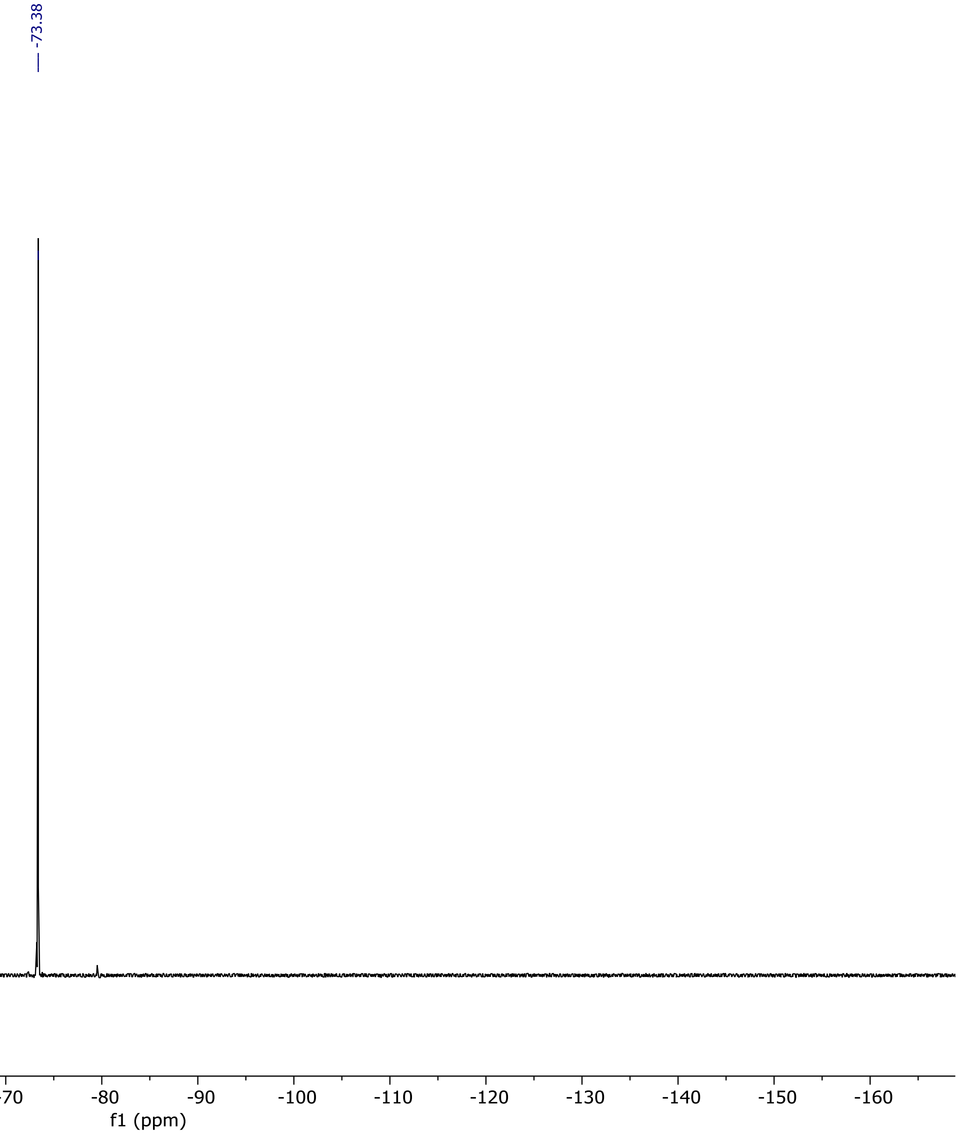

1H NMR of bicyclo[1.1.1]pentylalkane 18

CDCl3, 298 K

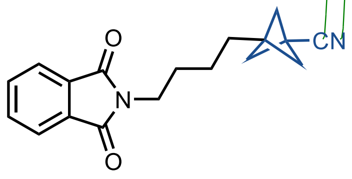

18

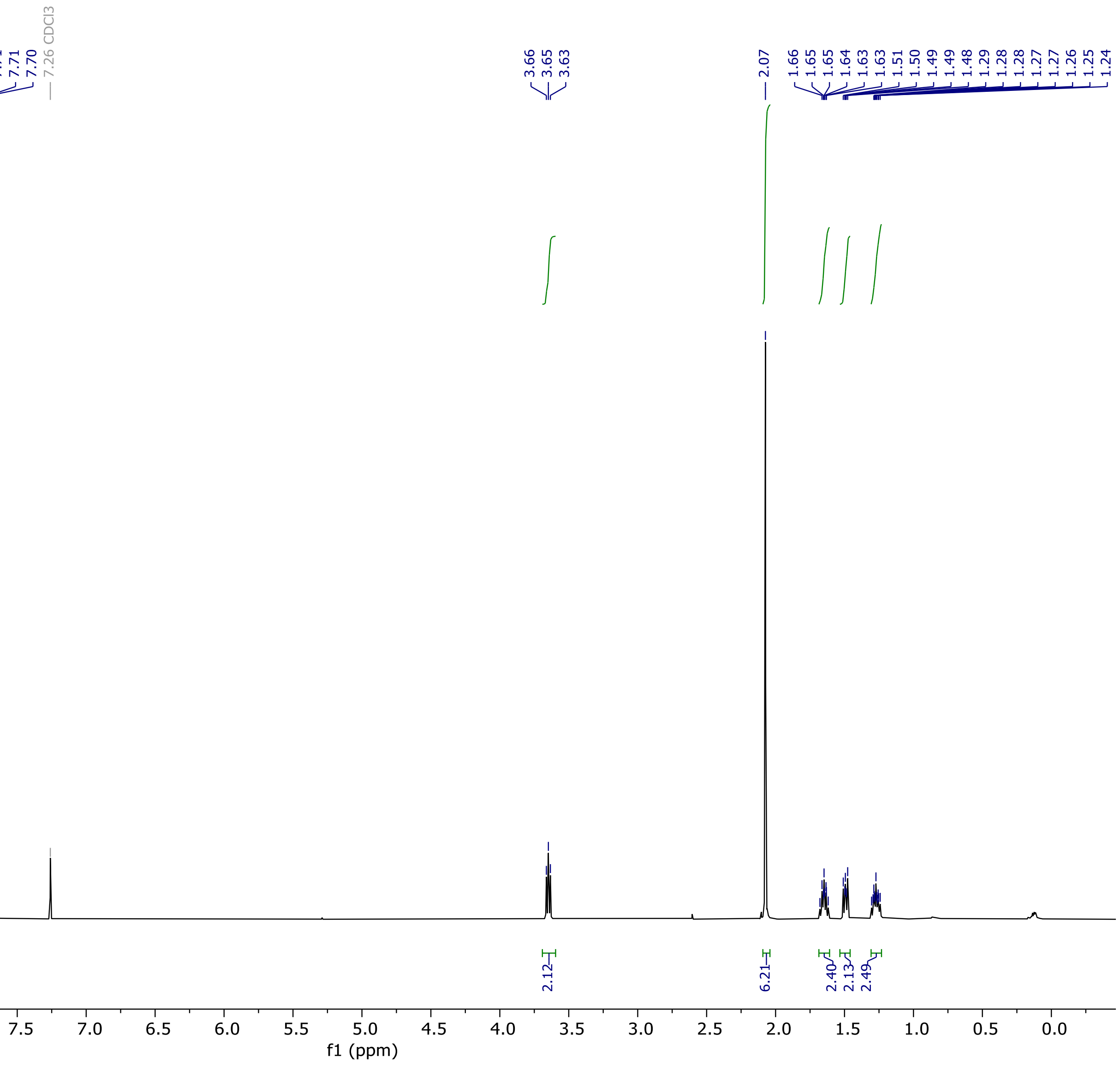

^{13}C NMR of bicyclo[1.1.1]pentylalkane 18

CDCl₃, 298 K

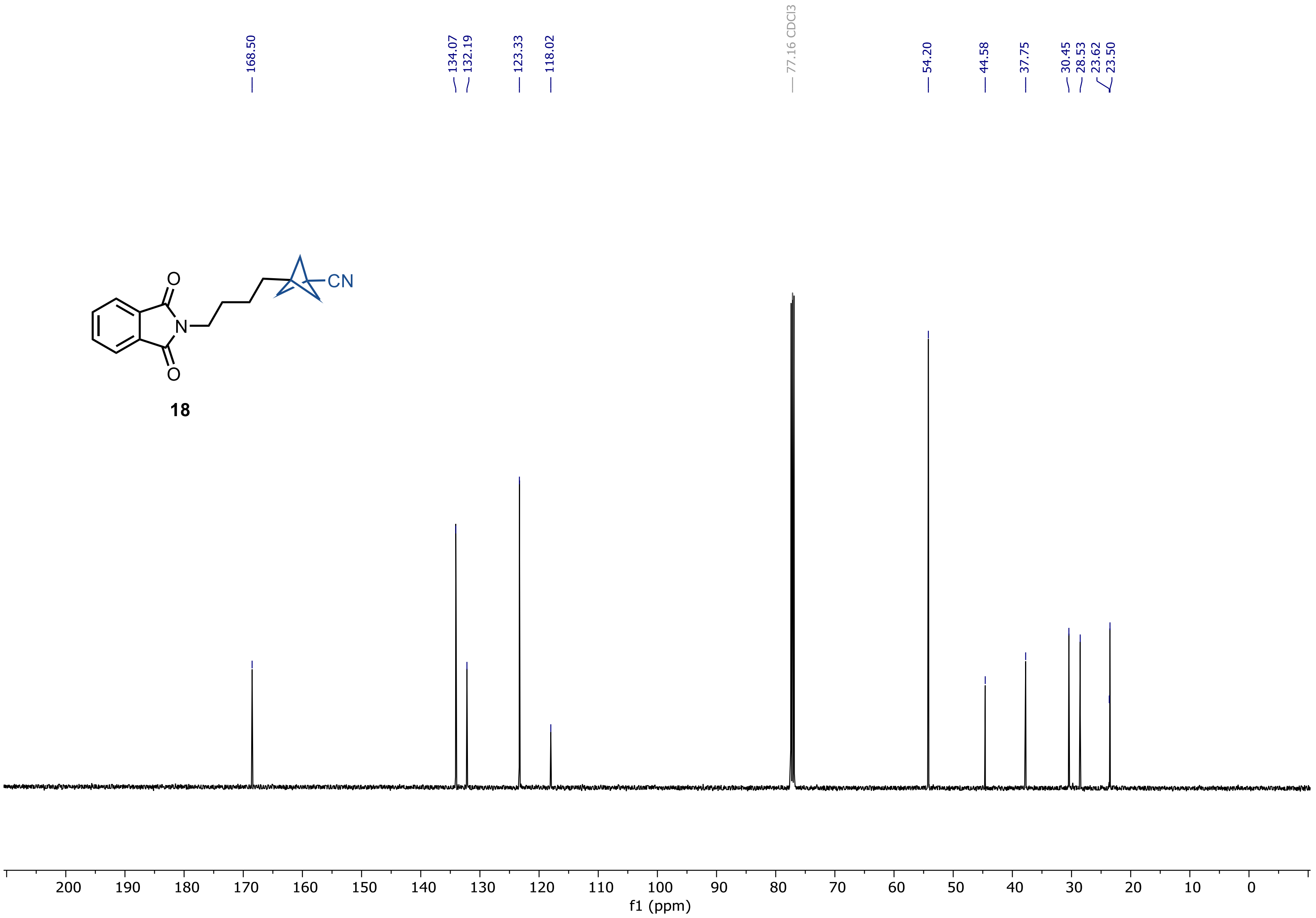

^{1}H NMR of bicyclo[1.1.1]pentylmethylarene 19

$CDCl_3$, 298 K

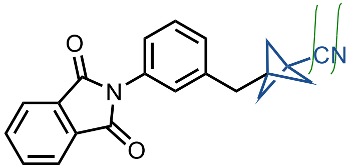

19

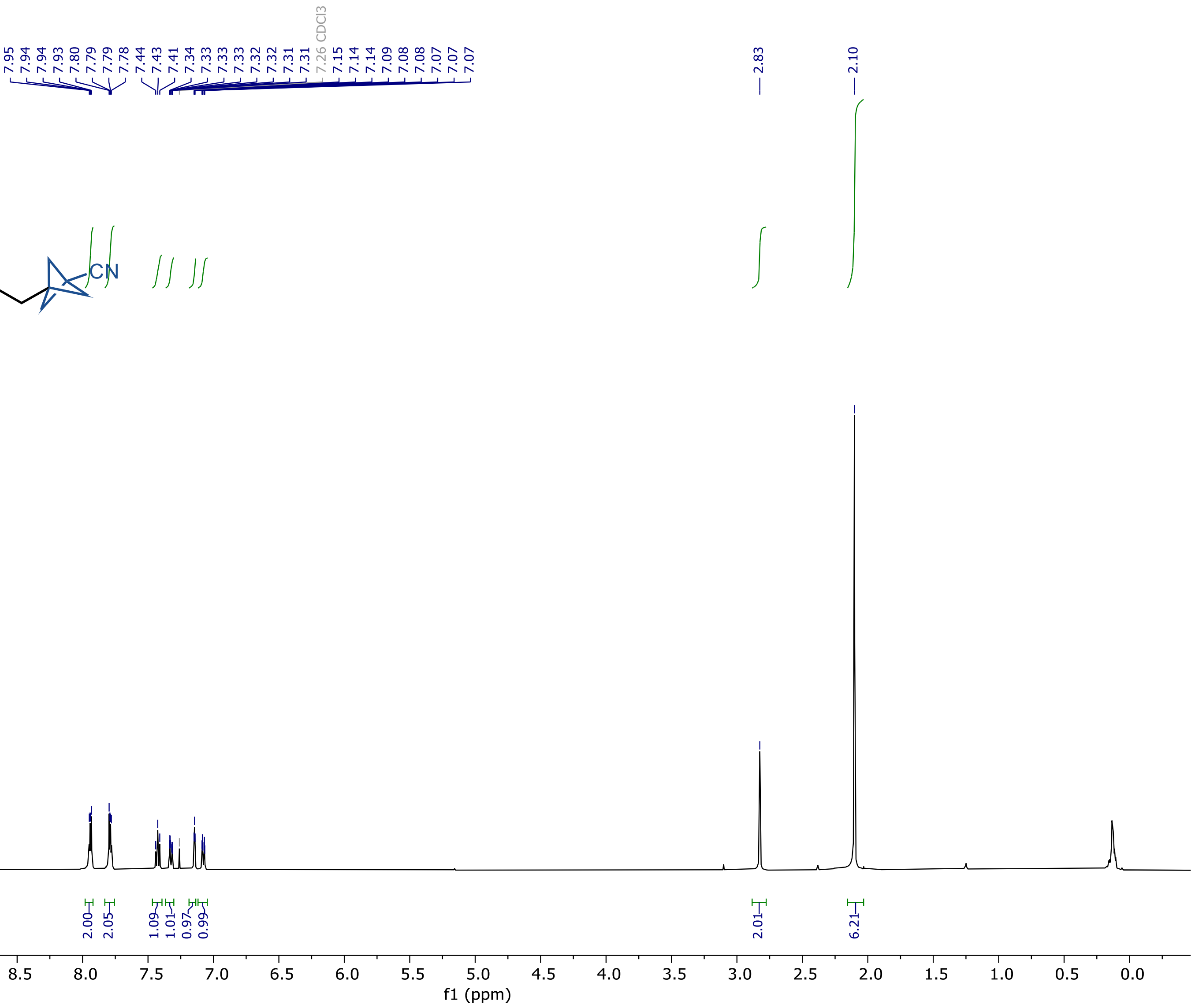

^{13}C NMR of bicyclo[1.1.1]pentylmethylarene 19

$CDCl_3$, 298 K

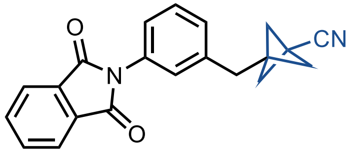

19

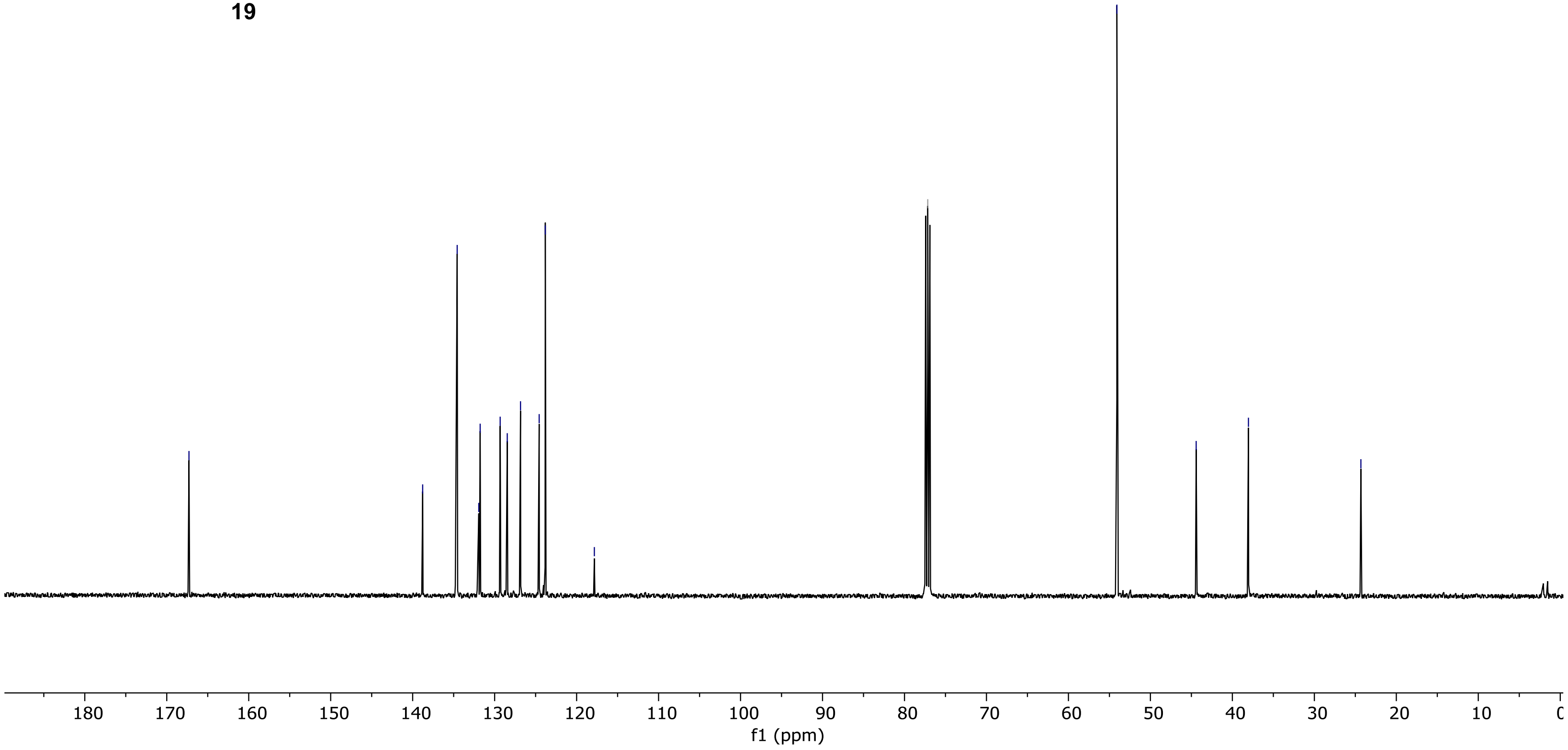

^{1}H NMR of bicyclo[1.1.1]pentylmethylarene 20

CDCl$_3$, 298 K

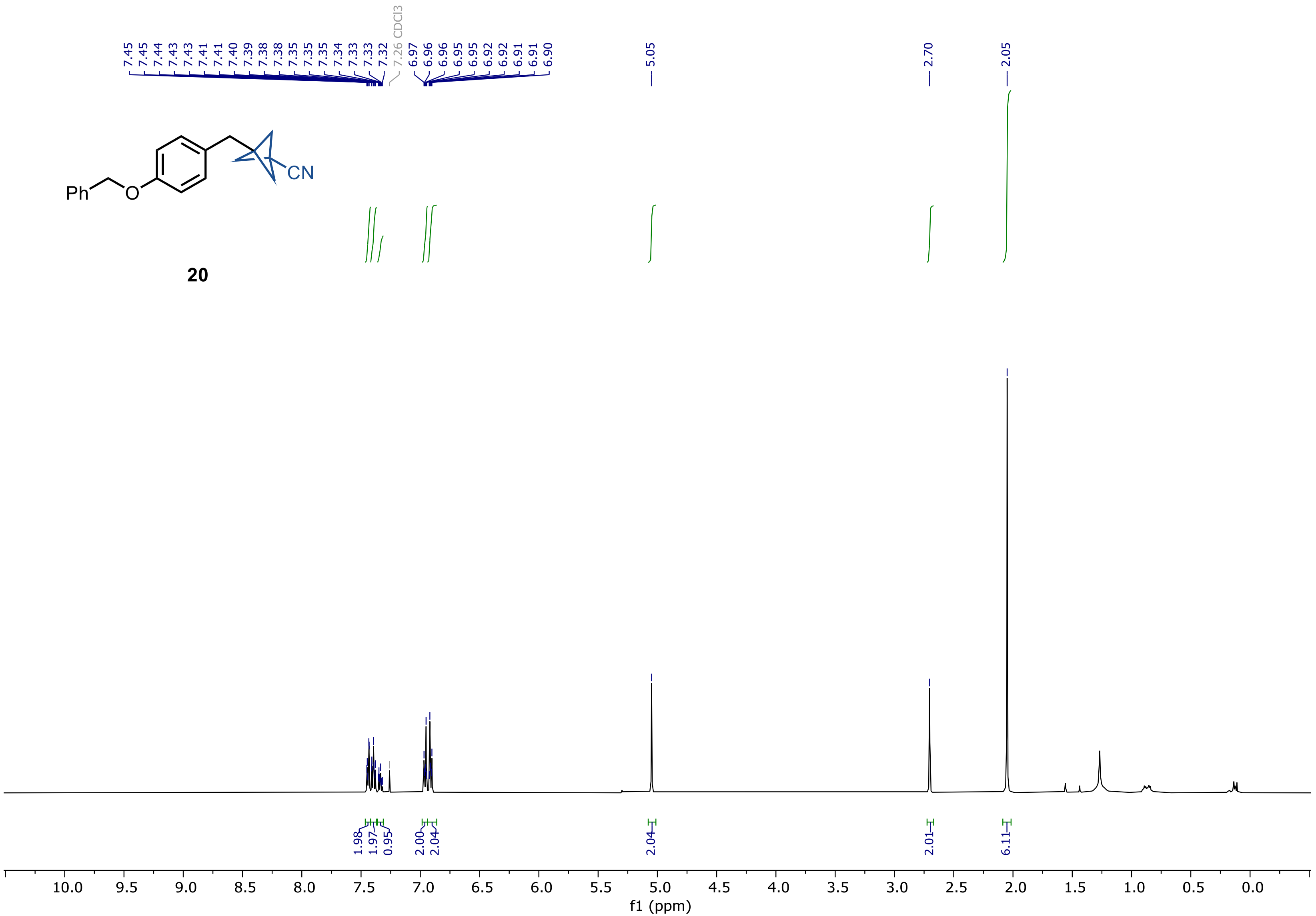

^{13}C NMR of bicyclo[1.1.1]pentylmethylarene 20

$CDCl_3$, 298 K

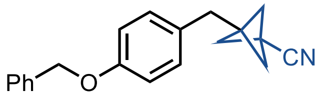

20

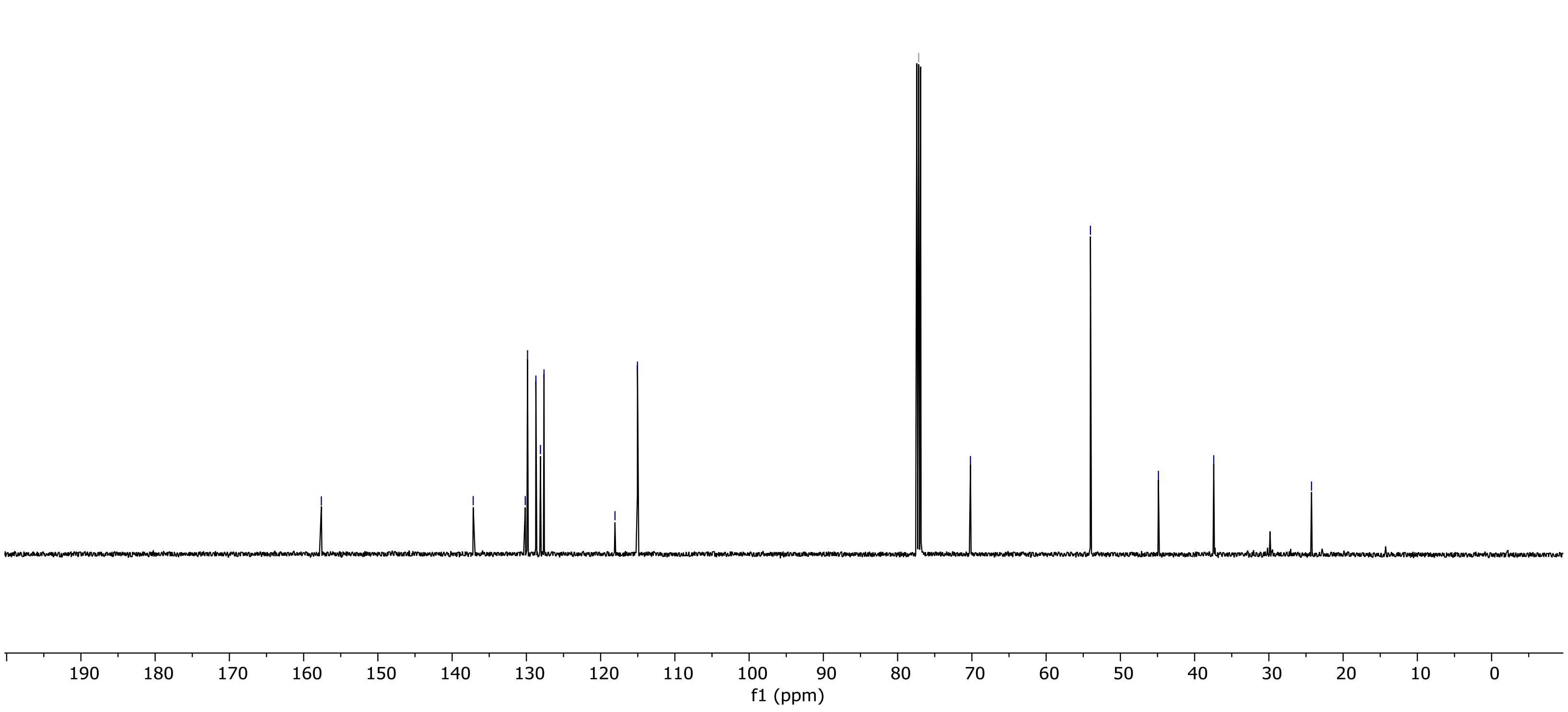

^{1}H NMR of bicyclo[1.1.1]pentylalkane 21

$CDCl_3$, 298 K

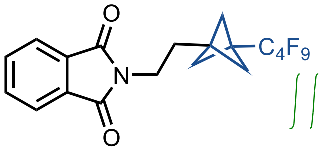

21

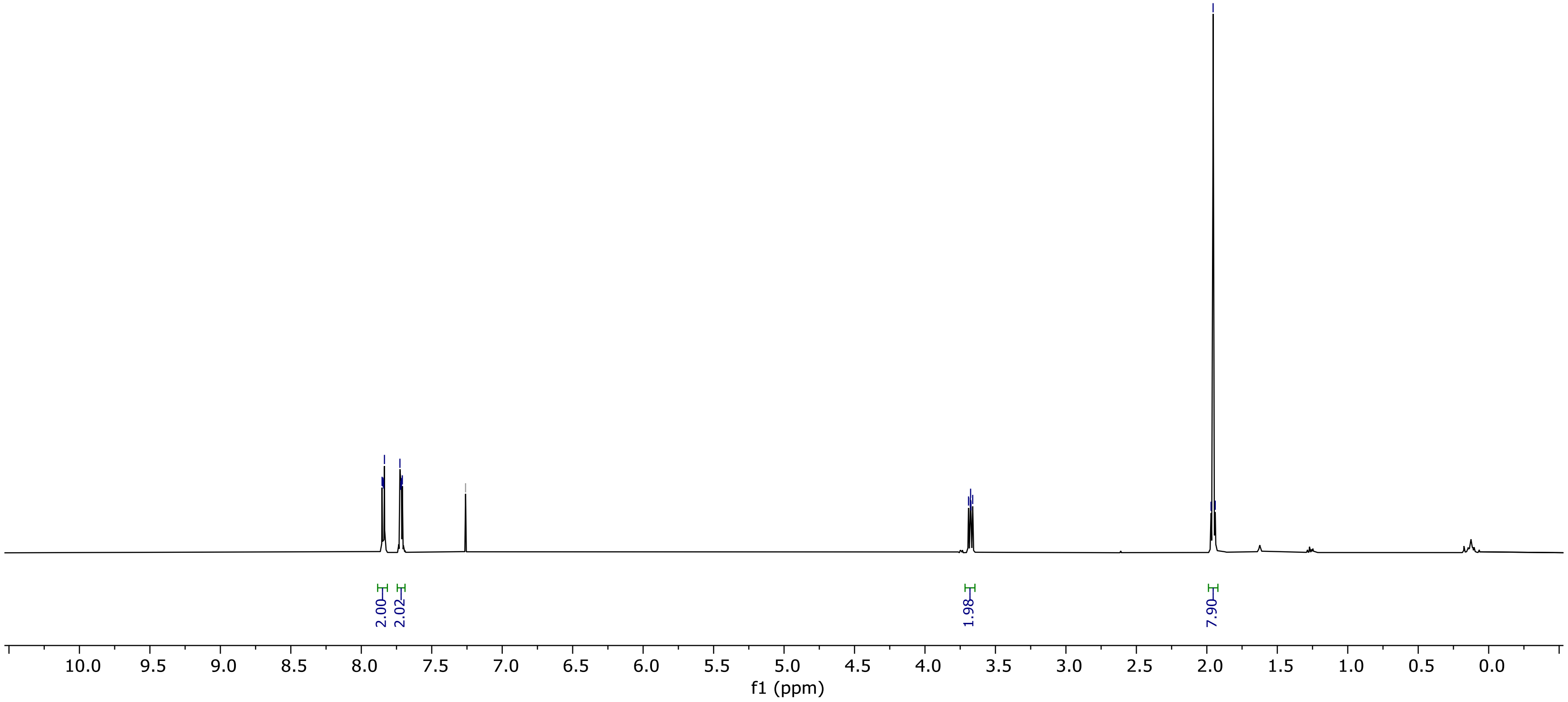

^{13}C {^{1}H, ^{19}F} NMR of bicyclo[1.1.1]pentylalkane 21

$CDCl_3$, 298 K

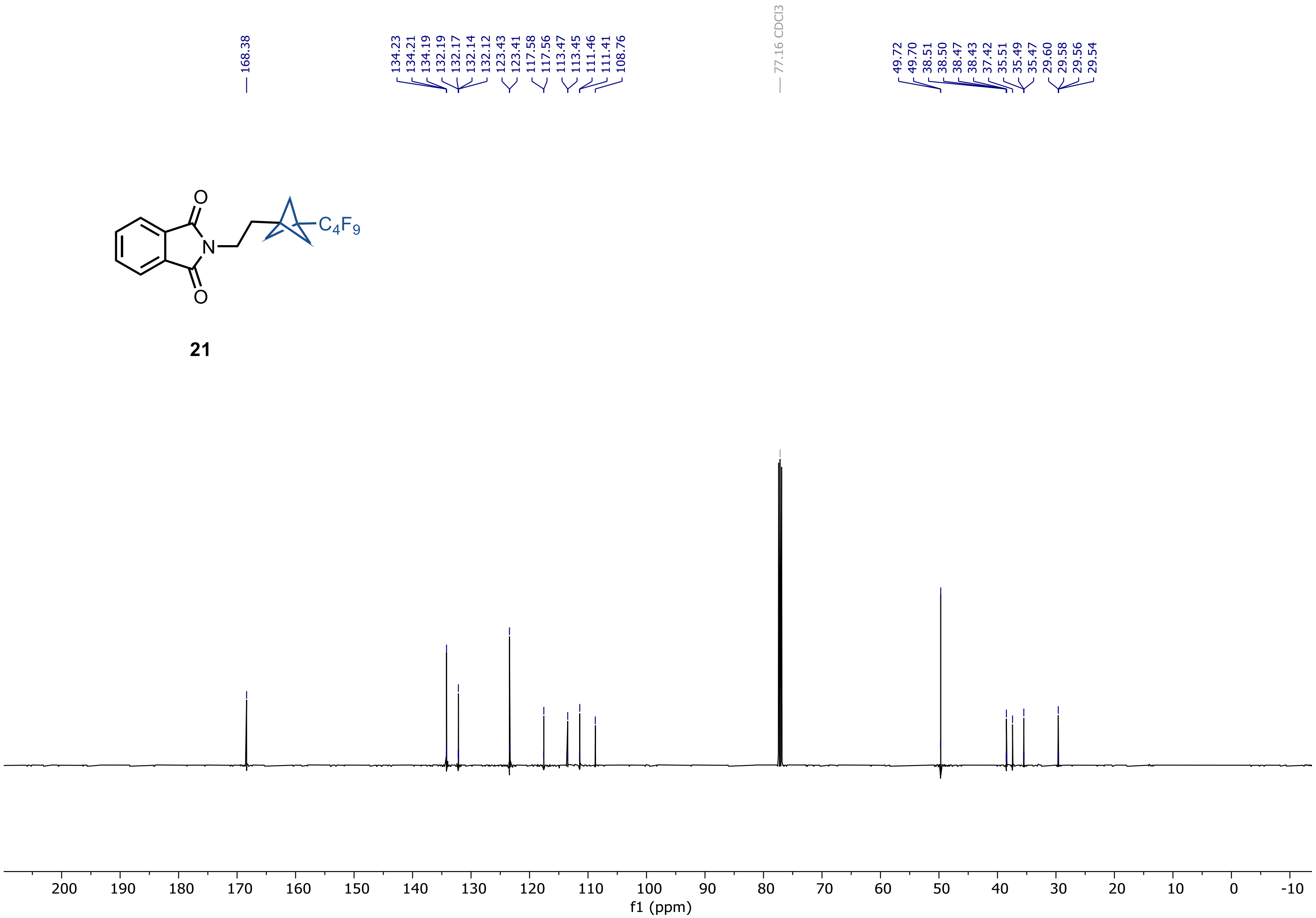

^{19}F NMR of bicyclo[1.1.1]pentylalkane 21

$CDCl_3$, 298 K

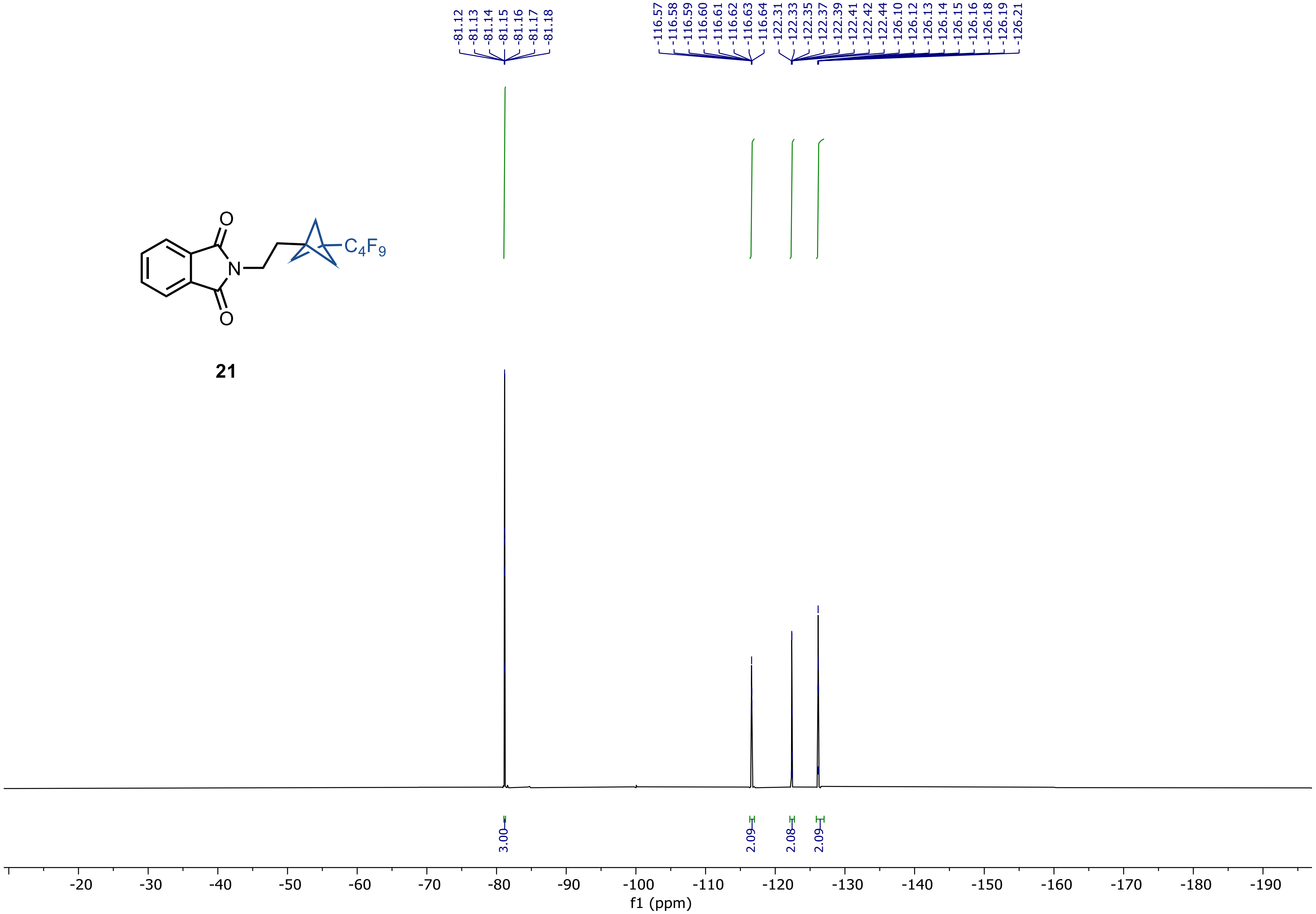

21

^{1}H NMR of bicyclo[1.1.1]pentylmethylpyridine 22

$CDCl_3$, 298 K

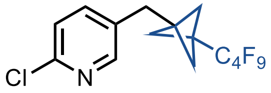

22

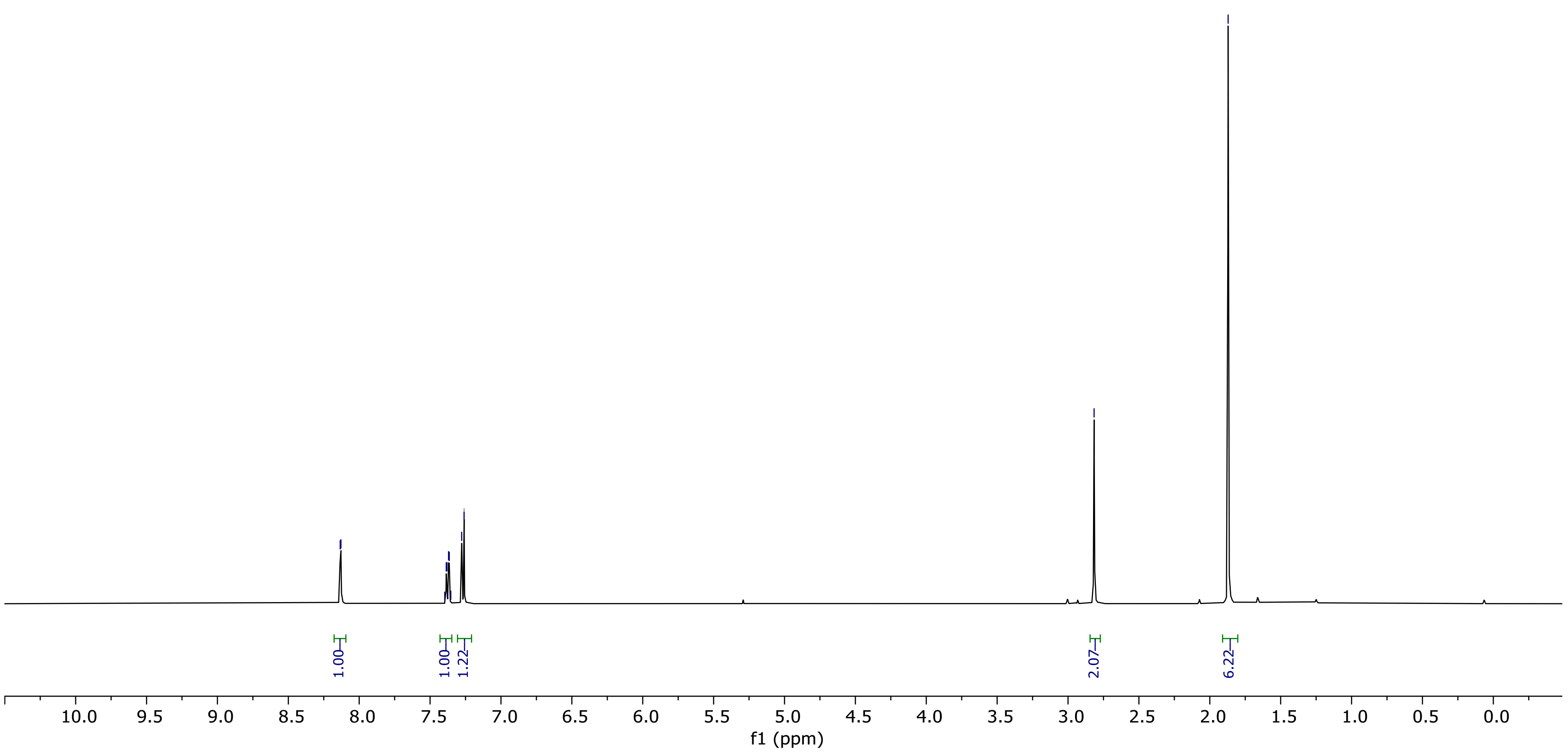

^{13}C {^{1}H, ^{19}F} NMR of bicyclo[1.1.1]pentylmethylpyridine 22

$CDCl_3$, 298 K

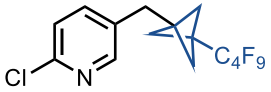

22

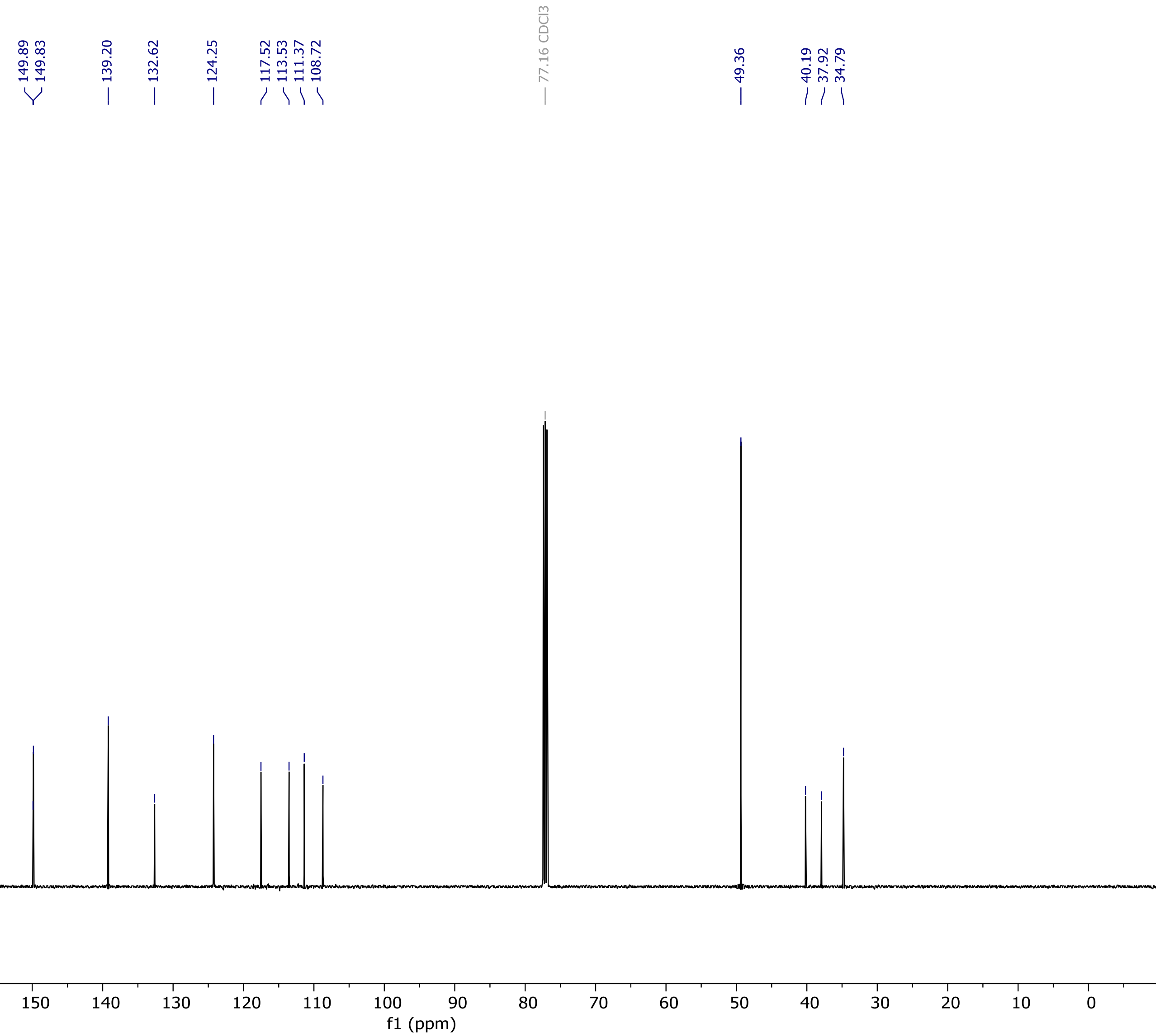

^{19}F NMR of bicyclo[1.1.1]pentylmethylpyridine 22

$CDCl_3$, 298 K

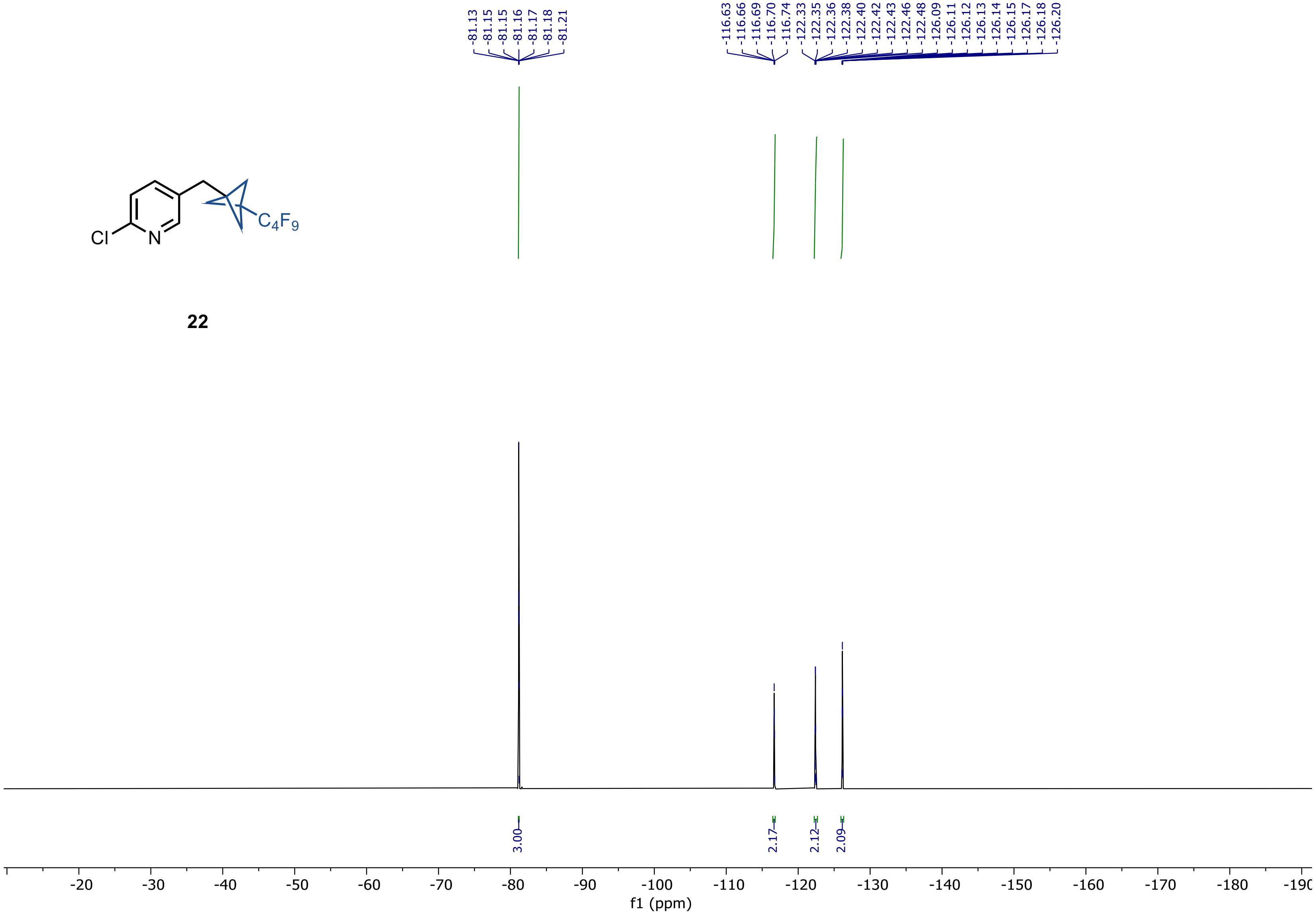

^{1}H NMR of intermediate 23

$CDCl_3$, 298 K

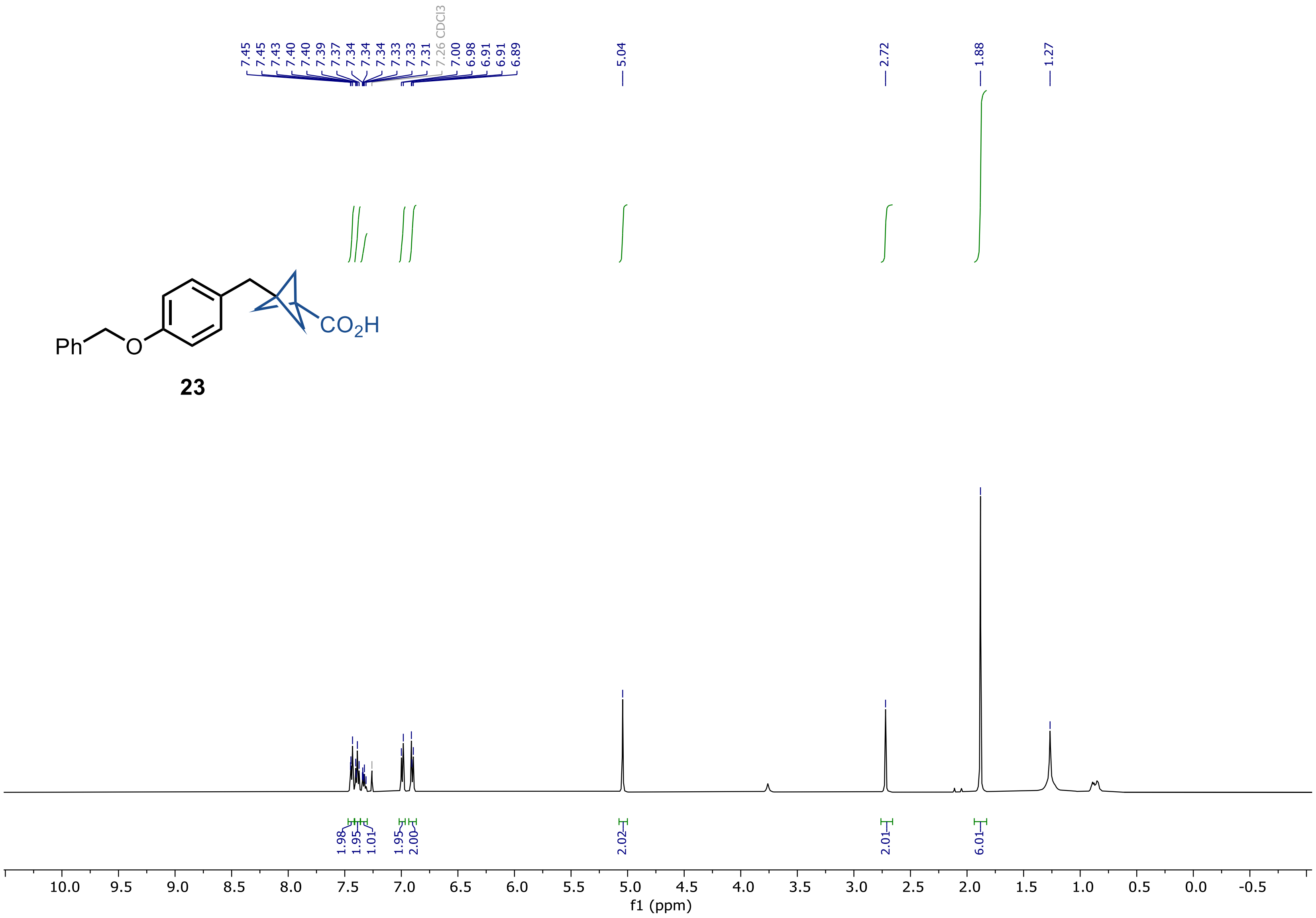

^{13}C NMR of intermediate 23

CDCl$_3$, 298 K

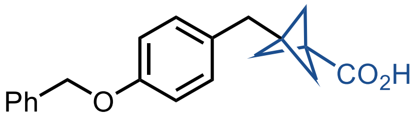

23

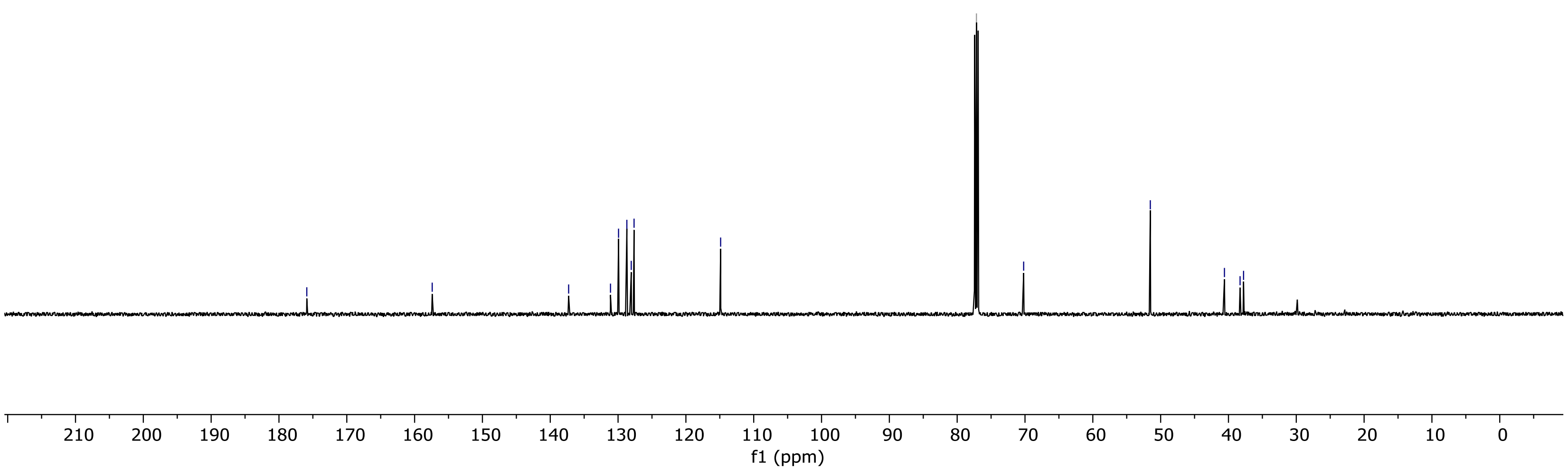

^{1}H NMR of intermediate 24

CDCl$_3$, 298 K

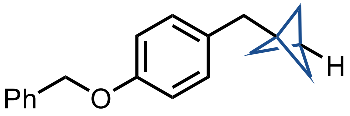

24

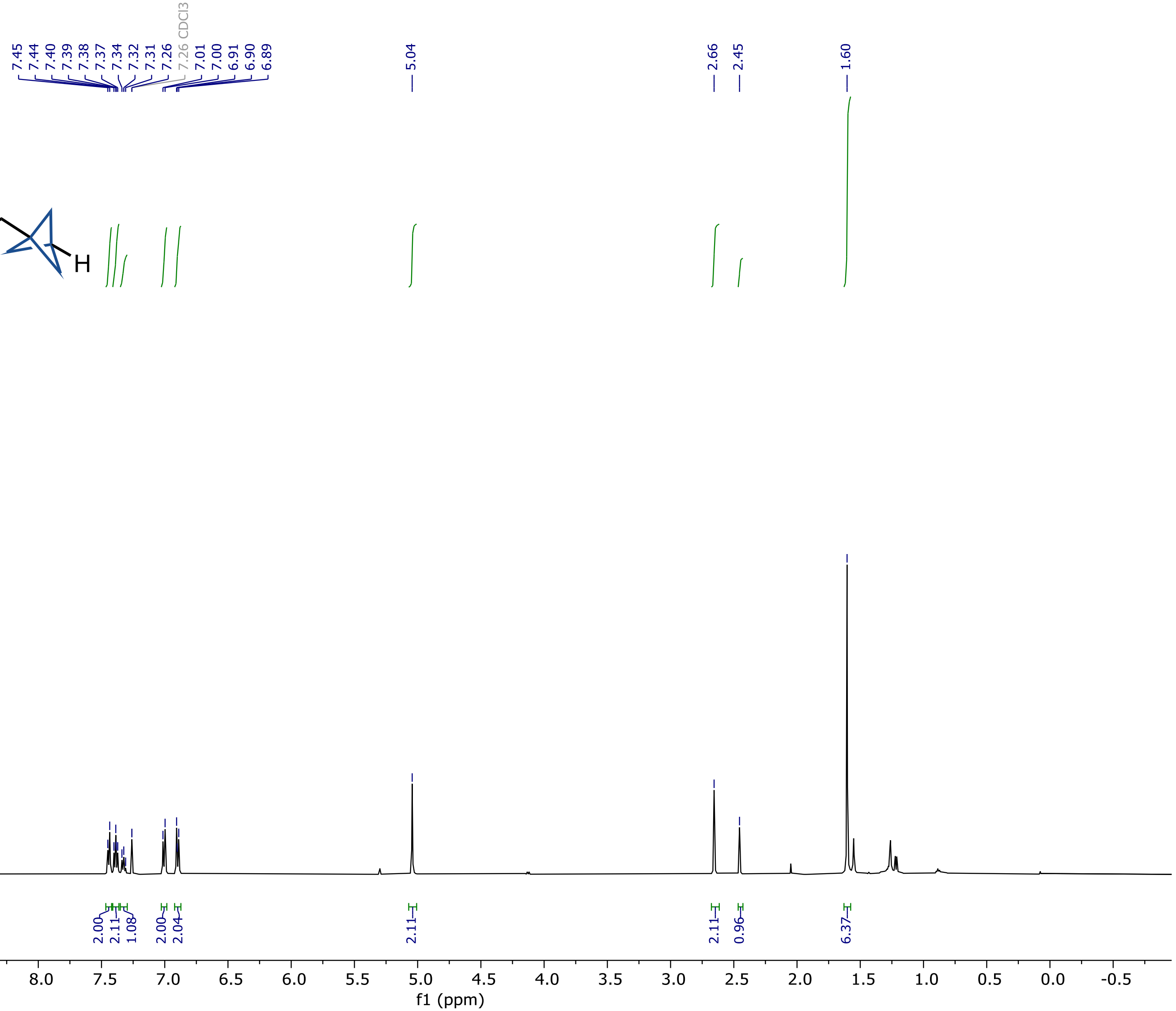

^{13}C NMR of intermediate 24

$CDCl_3$, 298 K

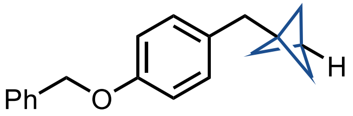

24

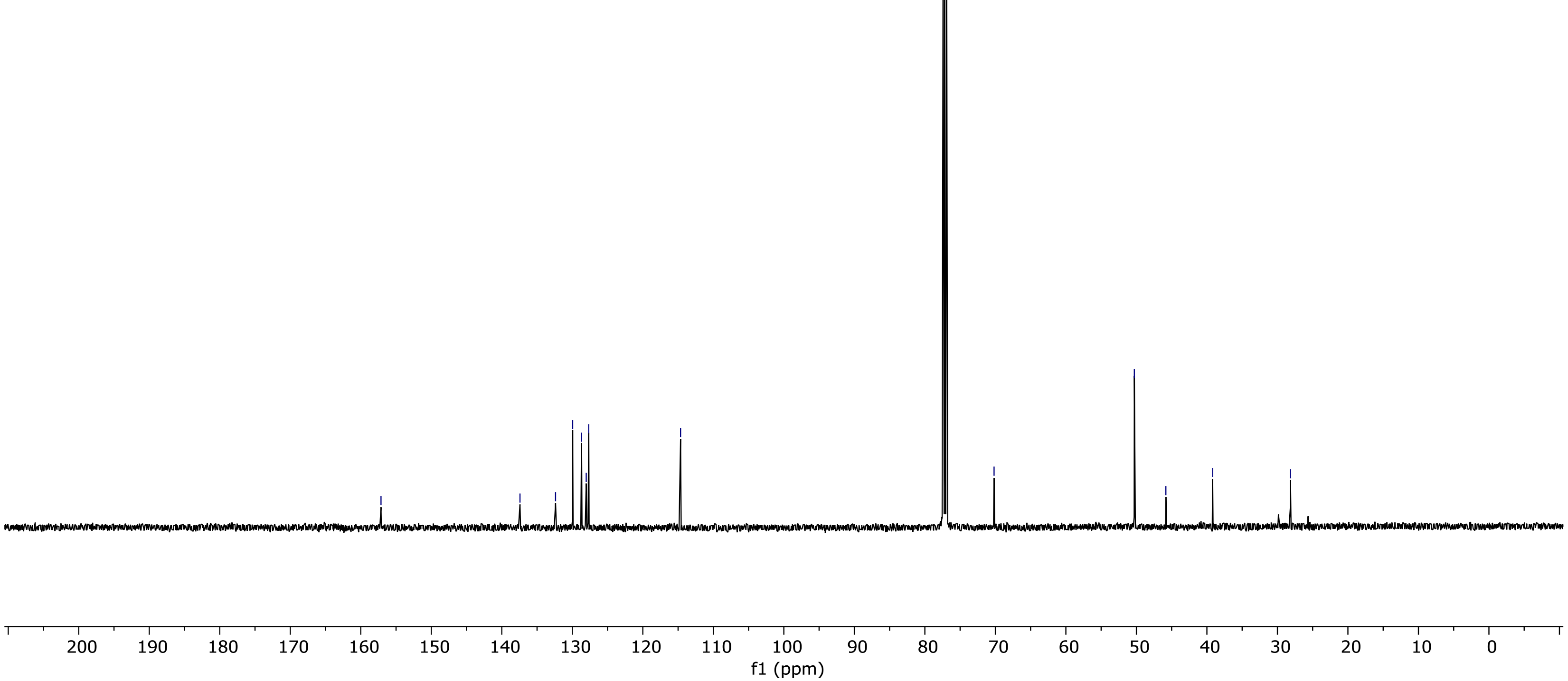

^{1}H NMR of BCP analog of tesmilifene 25

$CDCl_3$, 298 K

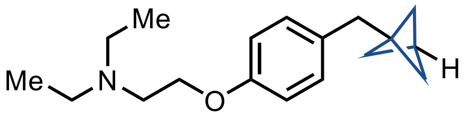

25

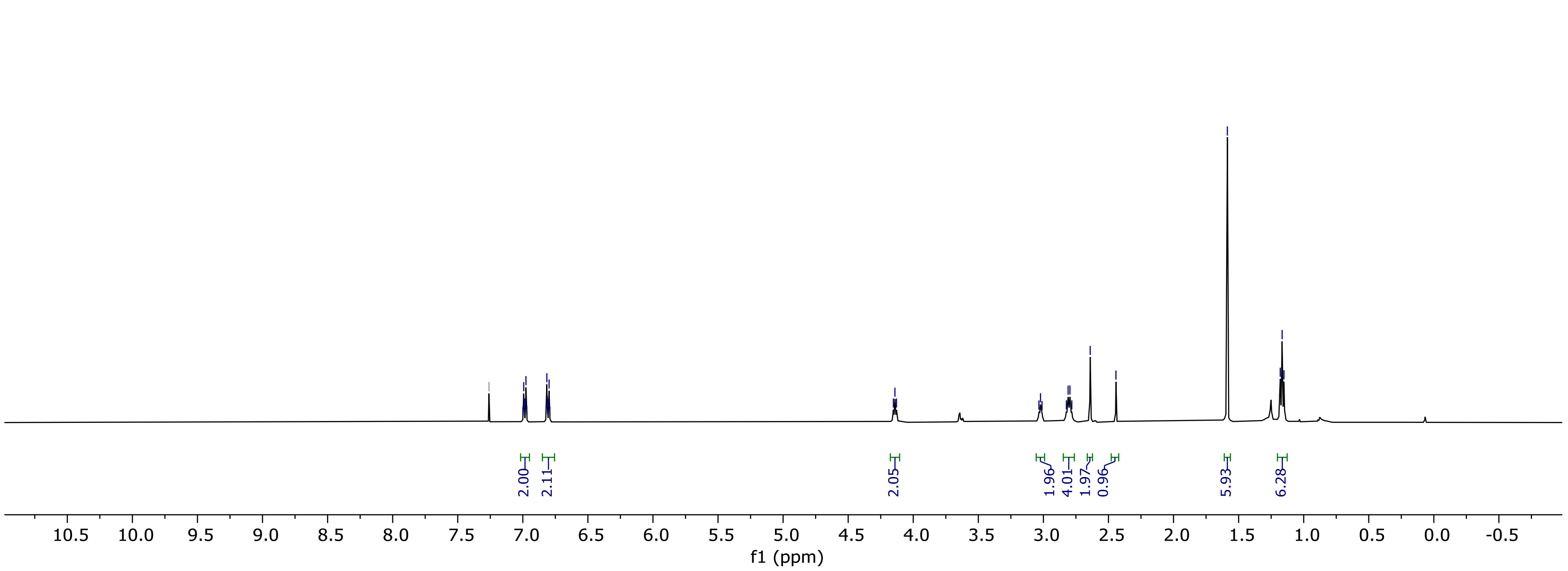

^{13}C NMR of BCP analog of tesmilifene 25

CDCl$_3$, 298 K

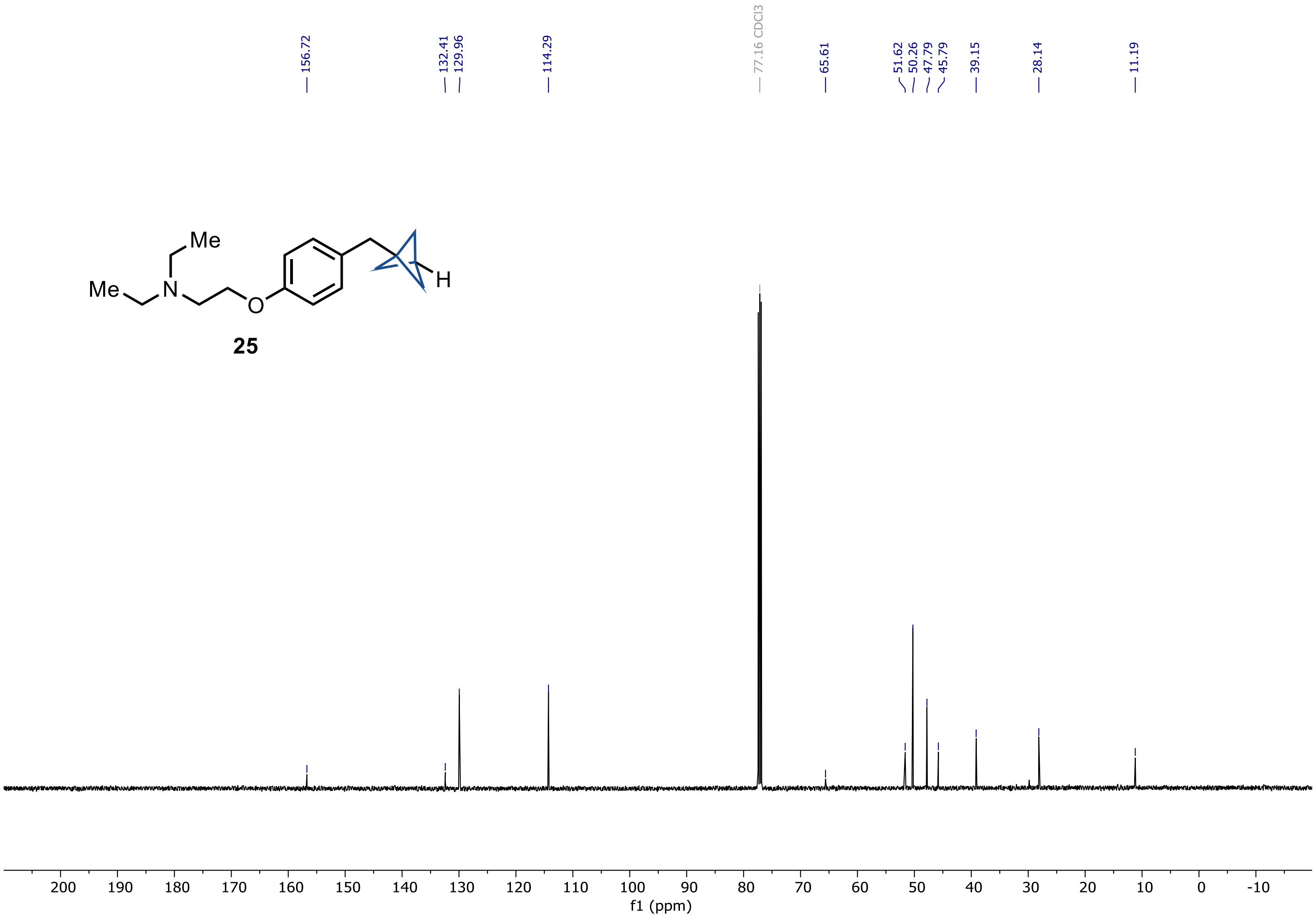

25

REFERENCES

1. G. R. Fulmer, A. J. M. Miller, N. H. Sherden, H. E. Gottlieb, A. Nudelman, B. M. Stoltz, J. E. Bercaw, K. I. Goldberg, *Organometallics* **2010**, *29*, 2176–2179.
2. R. K. Harris, E. D. Becker, S. M. Cabral de Menezes, R. Goodfellow, P. Granger, *Solid State Nucl. Magn. Reson.* **2002**, *22*, 458–483
3. E. M. Alvarez, Z. Bai, S. Pandit, N. Frank, L. Torkowski, T. Ritter, *Nat. Synth.* **2023**, *2*, 548–556.
4. H. Jia, A. P. Häring, F. Berger, L. Zhang, T. Ritter. *J. Am. Chem. Soc.* **2021**, *143*, 7623–7628.
5. C. Le, T. Q. Chen, T. Liang, P. Zhang, D. W. C. MacMillan, *Science* **2018**, *360 (6392)*, 1010–1014.
6. K. R. Abdellatif, M. A. Chowdhury, Y. Dong, C. Velázquez, D. Das, M. R. Suresh, E. E. Knaus, *Bioorg. Med. Chem.* **2008**, *16*, 9694–9698.
7. W. Xue, R. Shishido, M. Oestreich, *Angew. Chem. Int. Ed.* **2018**, *57*, 12141–12145.
